# Genomic and proteomic analysis of the Alkali-Tolerance Response (AlTR) in *Listeria monocytogenes *10403S

**DOI:** 10.1186/1471-2180-8-102

**Published:** 2008-06-24

**Authors:** Efstathios S Giotis, Arunachalam Muthaiyan, Ian S Blair, Brian J Wilkinson, David A McDowell

**Affiliations:** 1 Food Microbiology Research Group, University of Ulster, Northern Ireland, UK; 2 Microbiology Group, Illinois State University, Normal, IL, USA

## Abstract

**Background:**

Information regarding the Alkali-Tolerance Response (AlTR) in *Listeria monocytogenes *is very limited. Treatment of alkali-adapted cells with the protein synthesis inhibitor chloramphenicol has revealed that the AlTR is at least partially protein-dependent. In order to gain a more comprehensive perspective on the physiology and regulation of the AlTR, we compared differential gene expression and protein content of cells adapted at pH 9.5 and un-adapted cells (pH 7.0) using complementary DNA (cDNA) microarray and two-dimensional (2D) gel electrophoresis, (combined with mass spectrometry) respectively.

**Results:**

In this study, *L. monocytogenes *was shown to exhibit a significant AlTR following a 1-h exposure to mild alkali (pH 9.5), which is capable of protecting cells from subsequent lethal alkali stress (pH 12.0). Adaptive intracellular gene expression involved genes that are associated with virulence, the general stress response, cell division, and changes in cell wall structure and included many genes with unknown functions. The observed variability between results of cDNA arrays and 2D gel electrophoresis may be accounted for by posttranslational modifications. Interestingly, several alkali induced genes/proteins can provide a cross protective overlap to other types of stresses.

**Conclusion:**

Alkali pH provides therefore *L. monocytogenes *with nonspecific multiple-stress resistance that may be vital for survival in the human gastrointestinal tract as well as within food processing systems where alkali conditions prevail. This study showed strong evidence that the AlTR in *L. monocytogenes *functions as to minimize excess alkalisation and energy expenditures while mobilizing available carbon sources.

## Background

The resistance of *L. monocytogenes *to alkali stress is of particular concern especially when mild alkali treatments are used in the food industry [1-6] and it may account for the bacterium's persistence in such environments [1]. Even after ingestion, *Listeria*'s ability to tolerate high pH conditions, suggest that this organism is capable of resisting pH-related human defence mechanisms, e.g. a rise and fall of the vacuolar pH in the phagolysosomes [7], and alkali conditions in the presence of pancreatic secretions [8].

Bacteria have developed many sophisticated strategies to withstand hostile alkali conditions, and as a result the organisms become more resistant to further stress. It has been reported that *Listeria *adapts to sublethal concentrations of alkali detergents and subsequently resists previously lethal alkali, osmotic, ethanol or thermal based cleaning procedures [9-11]. Until now, information on how *Listeria *copes with sudden alkali shock is very limited. The ability of *Listeria *to induce an Alkali-Tolerance Response (AlTR) could be a significant factor in predicting the pathogen's fate in alkali foods/food processing systems and the pathogen's virulence within the human gastrointestinal system.

Knowledge concerning the mechanisms used by gram-positive bacteria for adaptation and growth at alkali pHs comes mainly from studies of alkaliphilic strains of *Bacillus *species, such as *Bacillus halodurans *C-125 and *Bacillus pseudofirmus *OF4. These organisms react to alkali stress with a specific response, resulting in the transiently enhanced expression of a subset of genes that increase metabolic acid production, changes cell surface properties and increase the expression and activity of monovalent-cation/proton antiporters [12-15]. These changes are essential to maintain a neutral cytoplasmic pH and therefore for growth under alkali conditions [14]. On the contrary, the ability of other bacteria such as *Enterococcus faecalis *to withstand and adapt to alkali stress is not obligatorily correlated to the maintenance of a neutral cytoplasmic pH [16,17]. Their adaptation to alkali conditions is achieved mainly due to *de novo *protein synthesis by induction of proteins/enzymes, capable of remaining active at high pH [17]. These proteins, either provide a specific protective function to alkali pH (Alkali-Shock Proteins, ASP) [18-20] or general stress proteins (GSP) that provide a rather unspecific protective function in cells, regardless of the type of stress (i.e. heat shock proteins and proteins involved in the SOS response) [16,21,22].

As a first step to understand the response of *Listeria *cells towards alkali stress, we attempted to identify the contribution of protein synthesis in the induction of AlTR. This study has also attempted to identify the gene expression profile of alkali-adapted cells using DNA microarray techniques coupled with two-dimensional (2D) gel electrophoresis. Microarrays are a very sensitive method that can give vast amounts of information on mRNA changes that occur under stress conditions. However, genes are not necessarily transcribed and even when some genes are transcribed to m-RNA they are not automatically translated [23]. Thus, the identified number of gene transcriptional changes does not necessarily reflect the precise number of functional protein molecules [23]. In addition, 2D gel electrophoresis is a useful tool since it could provide additional functional information such as evidence for post-translational modification, or the relative abundance of the protein product that are not available when only microarrays are used for gene expression profiling.

Hence, the employed technologies are complementary, allow systematic genome and proteome analysis and in combination are expected to generate a comprehensive amount of expression data that may add to the information on the regulatory events involved in alkali adaptation in *L. monocytogenes*.

## Results and Discussion

### Protein synthesis inhibition

Preliminary experiments have shown that *L. monocytogenes *10403S grows in a wide range of pH values (5.0 to 9.0). Nevertheless, pH 9.5 was necessary to induce maximal tolerance against pH 12.0 (data not shown).

**Figure 1:**
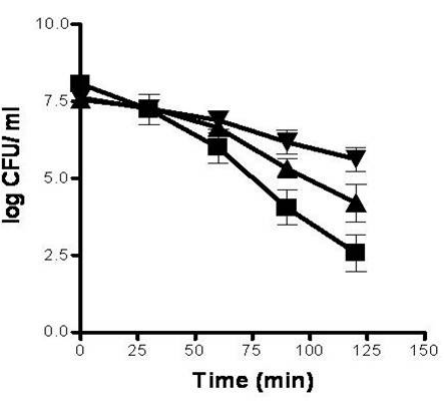
**Survival of *L. monocytogenes *mean populations in BHI (30°C) adjusted to pH 12.0 for 120 min.** Populations were either adapted at pH 9.5 with the presence of chloramphenicol (▲) or without chloramphenicol (▼) and not adapted at pH 9.5 (Control cultures) (■). Error bars indicate *s.e.m*.

We compared the relative survival of *Listeria *suspensions to lethal pH 12.0 with and without prior alkali adaptation to pH 9.5 (Figure 1). Chloramphenicol was used to determine whether *de novo *protein synthesis is required for the induction of AlTR. Chloramphenicol inhibits protein synthesis in bacterial cells by interacting with the 50S ribosomal subunit and inhibiting the peptidyltransferase reaction [24]. Un- adapted cells showed 1 and 3 orders of magnitude (logCFU/ml) less survival at lethal pH 12.0 than adapted cells (pH 9.5) with and without chloramphenicol respectively (Figure 1). Interestingly, when cells were adapted in the presence of chloramphenicol, a moderate AlTR was also induced. The phenotypic changes that take place during alkali adaptation show that activation or induction of proteins is necessary for the full induction of AlTR. Moreover, these results suggest also that there is an AITR dependent on protein synthesis and a *de novo *protein synthesis independent one.

Preliminary experiments showed that distinctive alterations in protein synthesis could be detected during the adaptation period at pH 9.5 by using one-dimensional sodium dodecyl sulfate-polyacrylamide gel electrophoresis (data not shown), suggesting also that protein synthesis might play a role in AlTR.

Unlike commonly used techniques (i.e. immunological methods) that are designed to examine individual adaptation mechanisms we have chosen to use broader methods of analysis to describe the molecular changes that take place during alkali adaptation. Microarrays and 2D gel electrophoresis allow a more comprehensive picture of the relationship between transcription, translation and the cellular physiological changes that occur within cells under stress.

### 2D electrophoresis and mass spectrometry

**Figure 2:**
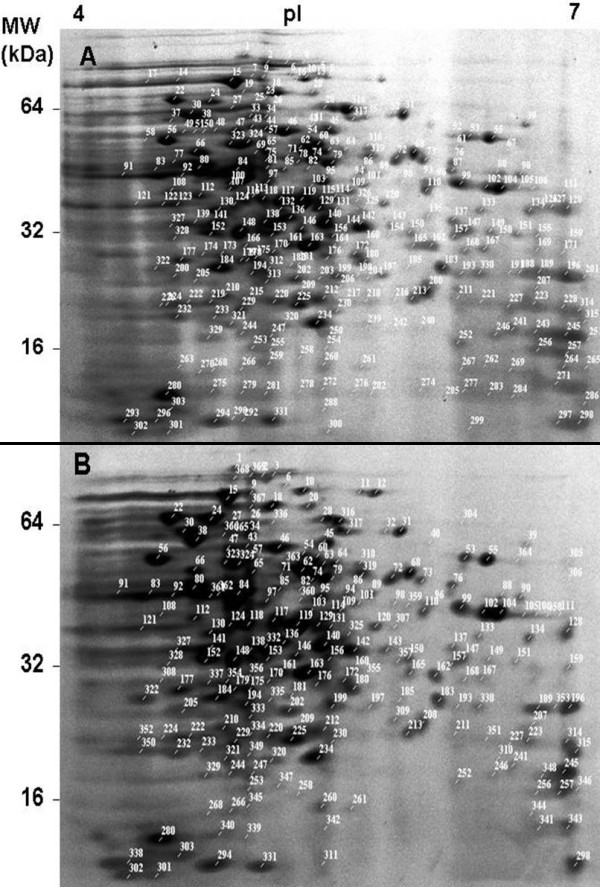
**Comparisons of 2D electrophoresis gels of proteins extracted from control (A) and adapted (B) cultures.** The reference numbers correspond to those listed in Additional file 1.

In this study cell protein extracts from *L. monocytogenes *adapted (pH 9.5 for 1 h) and control cultures (pH 7.4 for 1 h) were subjected to two-dimensional gel electrophoresis using initially a gradient ampholines strip with a pH range 3–10. Since there was no difference in protein composition below pH 4.0 and above pH 7.0, the analysis was then focused in the ampholines pH range 4.0–7.0 that could allow a better resolution of the protein spots. Approximately 331 spots could be resolved by the Progenesis software (*P *< 0.05) (Figure 2). The synthesis of 45 major protein spots was significantly upregulated and 45 protein spots were downregulated during the alkali adaptation at least 1.5 fold. 113 proteins were not expressed at all in stressed bacteria and 8 proteins were newly synthesised in comparison with the non-adapted cells. This analysis shows that the expression of most proteins was repressed to a varying level during alkali stress (Figure 2).

Identification of proteins was performed by in-gel trypsin digestion and MALDI-TOF. Among the spots analysed by mass spectrometry (10 initially) only 4 protein spots were identified (Table 1). Among the proteins identified as upregulated were two heat shock proteins GroEL and DnaK. It has been reported that DnaK chaperone [25] and possibly GroEL [26] are required for stress tolerance. Identified down regulated proteins included products of the genes *Atp*D (an ATP synthase), and *ddl *(D-alanine-D-alanine ligase), which is involved in cell wall formation. AtpD belongs to the *atp *operon that encodes the F_1_F_o _ATP synthase that imports H^+ ^during oxidative phosphorylation [27]. The downregulation of ATP synthase at high pH in *Listeria *is in disagreement with *E. coli *where high pH induced the proton importing ATP synthase [27]. These data may suggest a suppression of oxidative phosphorylation and cell wall biosynthesis at high pH. Full proteomic analysis results can be found as supplementary data (Additional file 1).

**Table 1:** Alkali adaptation affected proteins identified by 2D electrophoresis and Microarrays (MAs)

**Spot number**	**Gene name**	**pI**		**MW (KD)**		**Protein description**	**MAs Fold change**	**2D Fold change**
**Induced proteins**		Observed	Expected	Observed	Expected			

22	DnaK	4.57	4.30	65.99	66,01	Class I heat-shock protein (molecular chaperone) DnaK	1.68	159
38	GroEL	4.70	4.43	62.10	57,37	Heat-shock protein (molecular chaperone)	ND	1.66
**Repressed proteins**								
108	ddlA	4.50	4.20	49.192	40,67	D-alanine – D-alanine ligase	-1.88	-5.41
66	atpD	4.69	4.47	56.978	51,61	AtpD protein	ND	-2.17

^ND^-Not detectable.

### Transcriptional profiling

The microarray data discussed in this manuscript have been deposited in NCBIs Gene Expression Omnibus (GEO, [28]) and are accessible through GEO Series accession number GSE7966 [29].

*t *– test analysis (*P *< 0.005) of the transcript profile data for untreated bacterial cultures (pH 7.0) and bacterial cultures exposed to alkali condition (pH 9.5) indicated that the transcript levels increased or decreased for 355 genes in response to alkali condition (see Additional file 2). SAM analysis using one class mode (*Δ *= 1.01; False discovery rate (FDR) at 5%;*q *< 0.05) yielded 390 genes with either increase or decrease in expression in response to alkali exposure. The expression data for significantly regulated genes considered likely to be involved in alkali response processes are summarised in Table 2; complete results of the cDNA microarray analyses are available at additional file 2. Additional file 3 presents a comparative table between results of this study and results of microarray analyses of *L. monocytogenes *in different stress conditions (heat, cold, salt and pressure).

**Table 2:** List of selected up- and down- regulated genes during alkali adaptation.

**Locus ID**	**Symbol**	**Fold**	***P *value**	**Sub-functional category**	**Gene/protein name**
*lmo2673*	NA	5.38	**0.000**	**Adaptations to atypical conditions**	Conserved hypothetical protein
*lmo1074*	NA	-3.92	**0.007**	**Amino acids, peptides and amines**	Highly similar to teichoic acid translocation permease protein TagG
*lmo2250*	arpJ	-3.65	**0.007**		Similar to amino acid ABC transporter, permease protein
*lmo0847*	NA	-3.34	**0.008**		Similar to glutamine ABC transporter (binding and transport protein)
*lmo0997*	clpE	3.64	**0.013**		ATP-dependent protease
*LMOf2365_2469*	NA	3.44	**0.025**	**Anions**	Phosphate ABC transporter, ATP-binding protein
*LMOf2365_2470*	NA	4.19	**0.002**		Phosphate ABC transporter, permease protein
*lmo2495*	NA	6.03	**0.000**		Similar to phosphate ABC transporter
*LMOf6854_2559*	NA	8.01	**0.000**		Phosphate ABC transporter, permease protein
*lmo0097*	NA	-6.17	**0.004**	**Aromatic amino acid family**	Similar to PTS system mannose-specific, factor IIC
*lmo2202*	NA	-3.61	**0.001**	**Biosynthesis**	Similar to 3-oxoacyl- acyl-carrier protein synthase
*LMOf2365_2674*	NA	3.18	**0.055**		Putative Dihydroxyacetone kinase, Dak1 subunit
*LMOf6854_2815*	NA	5.21	**0.000**		Dihydroxyacetone kinase, Dak2 subunit, putative
*LMOf6854_2814*	NA	5.79	**0.000**		Dihydroxyacetone kinase, Dak1 subunit, putative
*lmo1389*	NA	-4.31	**0.006**	**Carbohydrates, organic alcohols & acids**	Similar to sugar ABC transporter, ATP-binding protein
*LMOf2365_1877*	NA	-7.45	**0.003**	**Cations and iron carrying compounds**	Manganese ABC transporter, ATP-binding protein
*lmo1848*	NA	-6.13	**0.003**		Similar metal cations ABC transporter
*lmo2087*	NA	4.03	**0.004**		Similar to unknown proteins
*lmo2507*	ftsE	-4.06	**0.002**	**Cell division**	Highly similar to cell-division ATP-binding protein FtsE
*lmo0582*	iap	-3.9	**0.004**		P60 extra cellular protein, invasion associated protein iap
*lmo1603*	NA	-5.43	**0.006**	**Degradation of proteins, peptides, and glycopeptides**	Similar to aminopeptidase
*lmo2230*	NA	5.04	**0.000**	**Detoxification**	Similar to arsenate reductase
*LMOf2365_2669*	NA	3.22	**0.048**	**DNA interactions**	Transcriptional regulator, TetR family
*lmo1398*	recA	3.12	**0.001**	**DNA replication, recombination, and repair**	Recombination protein recA
*lmo2489*	uvrB	3.42	**0.026**		Excinuclease ABC (subunit B)
*lmo2676*	NA	3.48	**0.022**		Similar to UV-damage repair protein
*lmo2717*	cydB	-3.3	**0.004**	**Electron transport**	Highly similar to cytochrome D ubiquinol oxidase subunit II
*lmo1067*	NA	-4.37	**0.005**	**General**	Similar to GTP-binding elongation factor
*lmo2494*	NA	5.35	**0.001**	**Glycolysis/gluconeogenesis**	Similar to negative regulator of phosphate regulon
*lmo0786*	NA	-4.02	**0.001**	**Other**	Similar to acyl-carrier phosphodiesterase and to NAD(P)H dehydrogenase
*lmo1870*	NA	4.22	**0.002**		Similar to alkali phosphatase
*lmo0820*	NA	4.7	**0.000**		Some similarity to acatyltransferases
*lmo1974*	NA	5.12	**0.001**		Similar to transcription regulators, (GntR family)
*LMOh7858_0838*	NA	3.24	**0.104**	**PTS**	PTS system, mannose/fructose/sorbose family, IIA component subfamily
*lmo2632*	rplC	-3.44	**0.003**	**Ribosomal proteins : synthesis and modification**	Ribosomal protein L3
*lmo0250*	rplJ	-3.36	**0.004**		Ribosomal protein L10
*lmo2631*	rplD	-3.26	**0.004**		Ribosomal protein L4
*lmo1252*	NA	5.69	**0.001**	**Role category not yet assigned**	Similar to *B. subtilis *YxkD protein
*lmo2695*	NA	4.55	**0.001**	**Sugars**	Similar to dihydroxyacetone kinase

Most of the modifications at the transcription level were related to energy metabolism, and transport and binding proteins. This study showed strong evidence that the AlTR mechanism in *L. monocytogenes *functions as to minimize excess alkalisation and energy expenditures, while mobilizing available carbon sources (increased metabolism of glycerol, upregulation of ABC transporters).

Operons encoding processes of glycolysis (*lmo2476*, *lmo2367*, *lmo1571*, *pfk*, and *lmo0268*), the pentose phosphate way (*lmo2712, lmo1978*) were significantly down regulated. However, other metabolic pathways such as the metabolism of glycerol (dihydroxyacetone kinase, *lmo1055*) were induced during alkali adaptation.

For most of the genes involved in processes associated with cellular growth, a general decline of expression was observed after alkali treatment, a finding confirmed by the proteomics study, consistent with the growth arrest observed at pH 9.5. Fatty acid and phospholipid metabolism (12 genes), aminoacid biosynthesis (21), protein synthesis (47 genes), cell wall biosynthesis (26 genes) and cellular processes (33 genes) were downregulated, as the genes involved in the respective processes were under expressed after an hour of alkali adaptation.

Our results suggest that alkali treatment alters phosphate uptake and utilisation system in this organism. Many genes involved in the phosphate uptake (phosphate- ABC transporters), phosphorylation/desphosphorylation of proteins (phosphatases, i.e. alkali phosphatase) were upregulated. On the other hand, the gene encoding for the negative regulator of the *pho *regulon (*lmo2494*) was also upregulated. This contradiction resembles the AlTR in *Bacillus subtilis*, when a sudden up shift of the external medium to pH 9.0 resulted in the upregulation of the phosphate deficiency response genes [30]. It appears that alkali stress mimics phosphate starvation and this in turn signals the induction of phosphatases. In *Listeria *this picture is reinforced by the over expression (4.22 fold) of alkali phosphatase (*lmo1870*) that is usually only produced by the bacteria during phosphate starvation and is more active at high pH [31,32]. To the question of what advantage the generation of free phosphate groups would confer upon the alkali tolerance in *L. monocytogenes *we suggest that upregulation of phosphatases may be an adaptation of cells to high pH stress. Transport of solutes through the cell membrane occurs mainly by a phosphorylation/dephosphorylation process [33]. Depending on the cellular needs, phosphorylation of certain molecules may prevent them from passing through the membrane. This is also supported by changes in genes involved in the PTS phosphorylation cascade at least at the transcriptional (*lmo1783*, *lmo1720*, *lmo2373*, *lmo0096*, *lmo0097*, *lmo0098 *and *lmo1255*) level. Nevertheless, it cannot be ruled out that this phosphorylation/dephosphorylation process is involved in other critical pH homeostasis cellular processes, or that the regulation of the related genes is a secondary effect of the broader bioenergetic changes that take place within alkali-stressed cells.

The second largest group affected by alkali adaptation were transport and binding proteins. At least 87 genes coding for transport and binding proteins were differentially regulated after alkali treatment. These included genes encoding transporters of carbohydrates, organic acids, cations, peptides, amino acids and metals (Additional file 2). Monovalent cation/proton antiporters, and especially Na^+^/H^+ ^antiporters, have been suggested to have a large variety of important physiological roles, including resistance to elevated levels of Na^+ ^in the medium which is toxic to the cytoplasm, pH homeostasis, osmoregulation and signalling [14,34-37]. Na^+ ^excretion is essential in bacteria for maintaining an internally directed Na^+ ^gradient, which serves as a driving force for many transport systems [36,38]. It was therefore expected that the expression of Na^+^/H^+ ^and other cation antiporters is significantly upregulated in *Listeria*, to minimise most likely, the increase of intracellular pH. Moreover this is consistent with alkali responses in other organisms [15,36,39,40].

38 genes that encode ATP-binding cassette (ABC) transporters were up- or down- regulated under the alkali conditions tested in this study. ABC transporters play an important role in various cellular physiological processes [41-45]. These transport systems have been previously reported as responsible for the uptake and mobilisation of hydrocarbons, oligopeptides and other solutes during growth under alkali pH conditions [15,41]. Previous studies in a range of organisms have shown that the imported peptides can contribute to cytoplasmic acidification following the activity of digestion by peptidases [15,41]. Free acidic amino acids can consequently become significant sources of protons [14,39,46].

Various heat shock and universal stress proteins were alkali-induced such as the products of *dnaK*, *grpE *and *lmo0292 *(similar to heat-shock protein HtrA, serine protease). Noteworthy, is also the significant upregulation (3.64 fold) of *clpE*. ClpE belongs to a Clp family of chaperone proteins that have an ATPase activity and stabilize and/or assist in the correct folding and assembly of denatured proteins. Furthermore, Nair *et al *have reported that *clpE *is required for prolonged survival of *Listeria *at 42°C and plays an important role in both cell division and virulence of the pathogen [47]. The DNA damage response genes *uvr*B, *rec*A and *tag *were alkali-induced, as was *lmo2676 *encoding the Ultra Violet (UV) damage repair protein. Similar results were obtained in *E. coli *by Schuldiner *et al*. where an alkali pH sensitive step was necessary for the activation of the SOS response whether directly or via arrest of DNA replication or induction of DNA damage [48]. The present study has shown a cross-protective overlap of the AITR with other stress responses such as high temperature, cell wall, salt and oxidative stress since some genes reported to play a protective role in these types of stresses were induced after alkali adaptation as well. Alkali pH induces therefore in *L. monocytogenes, a *nonspecific multiple-stress resistance that may be vital for survival in the human gastrointestinal tract as well as within food processing systems where similar alkali conditions as the ones used in this study prevail.

The repression of several genes involved in cellular septation and division (i.e. *ftsE*, *iap*) (Table 2) confirms the results of our previous study on alterations of alkali-stressed *Listeria *cells in one or more of the later steps of normal binary fission [49]. It is not clear if an abnormal cellular division is either part of a wider adaptation strategy employed by the microorganism to counteract alkalinity or just a consequence of inhibition of important stages of cell division under adverse pH conditions. Currently, the evidence could support either suggestion. It is possible that *L. monocytogenes *is limiting the high energy demanding processes of cellular septation and division in order to 'save energy', and 'invests' in more immediate responses to counteract stress.

Furthermore, approximately 30% of the identified genes encode proteins with hypothetical or unknown function. Most of them show no similarity to genes of other organisms, implying that they might play a distinctive role in alkali adaptation in *L. monocytogenes*.

The number of proteins identified as differentially regulated under alkali adaptation in the proteomic study is smaller than the number of genes identified using microarrays. The explanation for less than a 100% correlation between mRNA and protein results is probably due to posttranslational modifications of proteins or due to the inability of two-dimensional electrophoresis to detect low abundance proteins. Additionally, differences in the pI of the identified proteins by mass spectrometry might be explained as well as a result of a posttranslational modification of the activity of the enzymes (i.e. phosphorylation).

## Conclusion

Overall, by examining both transcriptional and translational changes we have obtained a clearer picture of how genes and proteins interact within the cell under alkali stress. The AITR in *L. monocytogenes *appears to be complex and seems to be a combination of different regulatory networks. Both proteomic and transcriptomic approaches have shown adaptive changes in gene expression involving genes that are associated with virulence, the general stress response, and cell wall structure. However, neither proteome nor transcriptome studies can identify proteins that are regulated at the level of enzymic activity. This is particularly important for instance for the antiporters that participate in alkali pH homeostasis [15]. Thus, further experimental work is needed using biochemical or other approaches in order to fully elucidate the mechanisms underlying the AlTR.

## Methods

### Bacterial strains and growth conditions

One hundred microlitres of an overnight grown *Listeria monocytogenes *10403S culture were inoculated into a 250 ml Erlenmeyer flask with 100 ml of Brain Heart Infusion (BHI) broth, and cultures were incubated at 30°C with shaking at 200 rpm. Cells were harvested by centrifugation when the optical density at 600 nm reached ~0.4, and then rapidly transferred to prewarmed (30°C) alkali-adjusted BHI pH 9.5 (adapted cultures) or BHI, pH 7.0 (Control cultures) for 60 min. The pH of BHI medium was adjusted to 9.5 with a glycine-NaOH-NaCl buffer.

### Protein synthesis inhibition tests

For protein synthesis inhibition tests, 3 replicate cultures were adapted for 60 min with/or without chloramphenicol (10 μg/ml). Cells were then harvested by centrifugation, and pellets were suspended in alkali-adjusted BHI medium to pH 12.0. Cell viability was measured by standard plate counting in duplicate on BHI agar plates by using samples taken before and after exposure to stress. The minimum inhibitory concentration for chloramphenicol was found using Etest (AB-Biodisk) [50].

### Extraction of cellular proteins for 2-D gel electrophoresis

Extraction was carried out as previously described [51] with the following modifications: After incubation at pH 9.5 or 7.0, cells were collected by centrifugation at 8,000 × *g *for 5 mins, washed twice in Tris HCl (pH 7.5) containing 5 mM EDTA and 5 mM MgCl_2_. The suspension was then sonicated on ice using a Misonix 3000 sonicator for a total process time of 5 min with 90 secs intervals between sessions, followed by centrifugation at 15,000 × g for 5 min at 4°C to remove unbroken cells and cell debris. Proteins were precipitated by addition of 4 volumes of ice-cold acetone, incubated at -20°C for 2 h and finally collected by centrifugation (15,000 × *g*) at 4°C for 20 min in order to avoid contamination by salts or nucleic acids. Protein concentrations were determined by the Bradford assay with bovine serum albumin as standard [52].

### Protein analysis using 2D-gel electrophoresis

For 2-D gel electrophoresis protein extracts were resuspended in sample rehydration buffer (8 M urea, 2% CHAPS, 0.5% (v/v) ZOOM^® ^Carrier Ampholytes (Invitrogen, Paisley, UK), 0.002% bromophenol blue, DTT 0.5 M). Appropriate protein preparations were incubated into linear pH gradient 4–7 (3–10 initially) ampholines strips (ZOOM Strip; Invitrogen) for 12 h at room temperature. Isoelectric focusing was performed using the ZOOM IPGRunner (Invitrogen, Paisley, UK) with a step voltage protocol (200 V for 20 min, 450 V for 15 min, 750 V for 15 min, 2000 V for 2 h) powered by the PowerPac 3000 (Biorad). Strips were then equilibrated for 30 min with NuPAGE LDS sample buffer and reducing agent followed by 30-min incubation with alkylating solution provided by the manufacturer. The SDS-PAGE was performed at 200 V for 45 min using precast NuPAGE Novex Bis-Tris ZOOM (Invitrogen, Paisley, UK) gels (4–12%). Mark 12 protein standards (Invitrogen, Paisley, UK) were used to calculate the molecular weight of the spots. The gels were fixed and stained with Sypro Ruby stain (Molecular Probes) according to the manufacturer's instructions. Gel images were captured (300 dpi resolution) by using a Nikon D100 digital camera and saved as TIFF files for subsequent image analysis. Spot detection was performed with the Progenesis PG200 software (v. 2006, Nonlinear Dynamics, Newcastle, UK). Each independent experiment was repeated in triplicate. An averaged reference gel was produced against which the other gel images were matched. Selected proteins for further analysis were identified in at least two of the gels. The individual protein spot volumes were expressed as normalised volumes relative to the total detected spot volume. Proteins were considered to be differentially expressed if the mean percentage spot normalised volume for an individual protein was at least 1.5-fold up- or down-regulated. Significance was determined by a Student's t test with a *P *value of 0.05 and data that did not meet the above criteria were eliminated by the analysis.

### Mass spectrometry

Proteins selected for mass spectrometry (MS) analysis were excised from the 2D gel. Spots of interest were excised from 2-D gels using spot-picker pens (1.5 mm diameter). Spots were destained and trypsin digested as previously described [53]. Digests were analysed on a MALDI-TOF Voyager-DE BioSpectrometry Workstation (PerSeptive Biosystems, Framingham, MA, USA). The spectra generated were mass calibrated and deisotoped. The masses were then used for protein identification using the MASCOT search engine [54]. The calculated Mowse score was -10 × Log (*P*), with *P*, the probability that the observed protein match was a random event. A protein is considered to be identified in this study, by a Mowse score greater than 74 (*P *< 0.05).

### Isolation of RNA

RNA was extracted from control and stressed cultures using the QIAGEN RNA extraction kit (Qiagen, Valencia, CA). Briefly, 5 ml of culture was mixed with bacterial RNA protect solution (Qiagen) to stabilise the transcripts. The mixture was then centrifuged to collect the cells. Pellets were resuspended in 1 ml of Trizol (Invitrogen) and cells were broken using the FastPrep system (Qbiogene) at speed 6.0 for 40 seconds. From the broken cell lysate, RNA was extracted as per the manufacturer's instructions. Residual DNA fragments were removed by DNase (Ambion) treatment and extracted RNA was purified using the RNeasy mini kit (Qiagen).

## Microarray analysis

cDNA was synthesized from the purified RNA by the Institute of Genomics Research (TIGR) microarray protocol. In this process 5 μg of the purified RNA were annealed with random hexamers as primers (Invitrogen) (70°C/5 min and snap frozen in ice/1 min), and extended overnight at 42°C with SuperScript II reverse transcriptase (Invitrogen) in the presence of 0.1 M dithiothreitol 12.5 mM DNTP/aa-UTP (Ambion, Austin, TX) mix. Residual RNA was removed and cDNA was purified with a QIAquick PCR purification kit (Qiagen). Purified aminoallyl-modified cDNA was then recovered, labelled with Cy3 or Cy5 mono-functional NHS ester cyanogen dyes (Amersham Pharmacia Biotech, Piscataway, NJ), and purified, using Qiagen PCR purification kit following the manufacturers instructions.

Purified labelled cDNA from alkali treated and control samples were hybridised on *L. monocytogenes *microarray slides (Version 1) consisting of 70-mer oligonucleotides representing Open Reading Frames (ORFs) from *L. monocytogenes *strains EGD-e, 4b F2365, 1/2a F6854 and 4b H7858, obtained from Pathogen Functional Genomic Research Centre [55]. The array information is available in NCBIs Gene Expression Omnibus (GEO, [28]) and is accessible through GEO platform accession number GPL5170 [56]. Hybridisation was done as previously described by Riordan *et al*. [57]. Hybridised slides were scanned using a GenePix 4000B microarray scanner (Axon Instruments, Union City, CA). TIFF images of the hybridised arrays were analyzed using TIGR-Spotfinder [58]. Spots were analyzed by adaptive quantitation, and the local background was subsequently subtracted. Spots with background-corrected signal intensity (median) in both channels less than two fold of background intensity (median) were rejected from further analysis. Data normalization was performed on the remaining spots by Lowess algorithm (block mode; smooth parameter: 0.33) using TIGR-MIDAS software [59]. The normalized log_2 _ratio of test/reference signal for each spot was recorded. Genes with less than three data points were considered unreliable, and their data points were discarded as well. The averaged log_2 _ratio for each remaining gene on the six replicate slides was ultimately calculated. Significant changes of gene expression were identified with significance analysis of microarrays (SAM) software [60,61] using one class mode (*Δ *= 1.01444) [the measurement is the log (test/reference)_2 _ratio from two labelled samples hybridized to an array]. SAM assigns a score to each gene on the basis of change in gene expression relative to the standard deviation of repeated measurements. For genes with scores greater than an adjustable threshold, SAM uses permutations of the repeated measurements to estimate the percentage of genes identified by chance, the false discovery rate (FDR). A *"q value" *assigned to each gene corresponds to the lowest false discovery rate at which the gene is called significant [62]. The differentially expressed genes identified by SAM were further filtered on the basis of previously defined criteria [63-66]. Also, to test for significant differences in expression between the control and treatment samples, we calculated the mean signal intensities for each set of samples (control samples in neutral pH, samples treated with alkali pH) and applied a two-tailed Student's *t *test, to evaluate the differences between the means at a significance level of 0.005 in order to minimize the expected false positive at 0.5%.

To examine how genes with transcript level changes are distributed with regard to their function, we further classified these genes using our in-house software 'Gene Sorter' according to the categories described in the comprehensive microbial resource of TIGR [67].

Several controls were employed to minimise the technical and biological variations and ensure that the quality of the data: i) each ORF was present in duplicate in each array, ii) array slides were prepared in duplicate for each experiment, and fluorophore dyes were swapped between replicates to account for dye bias, iii) three independent RNA batches from each condition were used. Only differences of ≥ 1.5 fold changes in the levels of gene expression, which also had *q *values of 0.05, were recorded as significant [68-70].

## Authors' contributions

ESG performed the experiments, the proteomic data analysis, interpreted the results and drafted the manuscript. AM performed the microarray data analysis. AM, DAMcD and BJW participated in the design of the study, in evaluation of the results and in revision of the manuscript. ISB discussed the results and critically read the manuscript. All authors have read and approved the final manuscript.

## Supplementary Material

Additional file 1 Complete 2D gel electrophoresis results of protein extracts of cells stressed at pH 9.5. Spot matching between 2D electrophoresis spots of protein extracts from stressed *Listeria monocytogenes *cells at pH 7 and pH 9.5. Table displays normalized volumes of spots, wide isoelectric point (pI), molecular weights (MW) and differences between normalized values of spots at pH 7.0 and pH 9.5. Click here for file

Additional file 2 DNA microarrays results of cells stressed at pH 9.5. Genes that were upregulated or down regulated at least 1.5-fold in *Listeria monocytogenes *cells stressed at pH 9.5 compared with *Listeria monocytogenes *stressed at pH 7.0. Genes are ordered based on functional category and minor category and within minor category based on absolute fold change. The list contains genes that were considered significant according to *t *– test analysis with differences of ≥ 1.5 fold changes in the levels of gene expression, and *q *values of 0.05. Click here for file

Additional file 3 Gene expression pattern of *Listeria monocytogenes *in different stress conditions. Genes that were up regulated or down regulated at least 1.5-fold in *Listeria monocytogenes *cells challenged at heat, salt, pressure and cold stress compared with *Listeria monocytogenes *stressed at pH pH 9.5. Genes are ordered based on functional category and minor category and within minor category based on absolute fold change. Click here for file
