# Supplementary material for: Genomic and proteomic analysis of the Alkali-Tolerance Response (AlTR) in Listeria monocytogenes 10403S
Source: BMC Microbiol. 2008 Jun 24;8:102. doi: 10.1186/1471-2180-8-102 (PMC2443805; doi:10.1186/1471-2180-8-102)
Supplement: Additional file 3 — Gene expression pattern of Listeria monocytogenes in different stress conditions. Genes that were up regulated or down regulated at least 1.5-fold in Listeria monocytogenes cells challenged at heat, salt, pressure and cold stress compared with Listeria monocytogenes stressed at pH pH 9.5. Genes are ordered based on functional category and minor category and within minor category based on absolute fold change. [file 1471-2180-8-102-S3.doc]

| Additional file 3. Gene Expression of Pattern of  *Listeria monocytogenes* in Different Stress Conditions | | | | | | | | |
| --- | --- | --- | --- | --- | --- | --- | --- | --- |
|  |  |  |  |  | | | | |
| **Gene Symbol** | **Locus ID** | **Gene/protein Name** | **Sub category** | **Gene Expression Change in Fold** | | | | |
|  |  |  |  | **Alkaline^a^** | **Heat^b^** | **Cold^c^** | **Salt^d^** | **Pressure^e^** |
| **Unclassified** | |  |  |  |  |  |  |  |
| *NA* | lmo1252 | similar to B. subtilis YxkD protein | Role category not yet assigned | 5.69 | 0 | 0 | 0 | 0.96 |
| *NA* | lmo1303 | similar to B. subtilis YneA protein | Role category not yet assigned | 3.22 | UP | 0 | 0 | 0.03 |
| *NA* | lmo2158 | similar to B. subtilis YwmG protein | Role category not yet assigned | 2.75 | UP | 5.1 | 17.8 | -0.58 |
| *NA* | lmo2220 | similar to S. aureus Cbf1 protein | Role category not yet assigned | 2.47 | 0 | 0 | 0 | -2.17 |
| *NA* | lmo2411 | similar to conserved hypothetical proteins | Role category not yet assigned | 2.37 | 0 | 0 | 0 | 0.49 |
| *NA* | lmo1966 | similar to unknown proteins | Role category not yet assigned | 2.22 | 0 | 0 | 0 | 0.8 |
| *NA* | lmo0992 | conserved hypothetical protein | Role category not yet assigned | 2.13 | 0 | 0 | 0 | -1.07 |
| *NA* | lmo0163 | unknown | Role category not yet assigned | 1.93 | 0 | 0 | 0 | 0.47 |
| *NA* | lmo0674 | unknown | Role category not yet assigned | 1.93 | UP | 0 | 0 | -0.88 |
| *NA* | lmo2207 | similar to unknown protein | Role category not yet assigned | 1.78 | UP | 0 | 0 | -0.01 |
| *NA* | lmo1291 | similar to acyltransferase (to B. subtilis YrhL protein) | Role category not yet assigned | 1.71 | 0 | 0 | 2 | 0.15 |
| *NA* | lmo0591 | similar to unknown membrane proteins | Role category not yet assigned | 1.68 | UP | 0 | 5.4 | -0.81 |
| *NA* | lmo2565 | conserved hypothetical protein | Role category not yet assigned | 1.55 | 0 | 0 | 0 | -1.01 |
| *NA* | lmo1887 | similar to conserved hypothetical proteins | Role category not yet assigned | 1.52 | 0 | 2.26 | 0 | 1.19 |
| *NA* | lmo2414 | similar to aminotransferase | Role category not yet assigned | 1.5 | 0 | -2.28 | 0 | -0.07 |
| *NA* | lmo2246 | similar to unknown proteins | Role category not yet assigned | 1.47 | 0 | 0 | 0 | 2.32 |
| *NA* | lmo2386 | similar to B. subtilis YuiD protein | Role category not yet assigned | 1.43 | UP | 0 | 2.3 | 0.02 |
| *NA* | lmo1941 | similar to unknown proteins | Role category not yet assigned | 1.36 | 0 | 0 | 0 | 1.54 |
| *NA* | lmo1009 | similar to B. subtilis YkuL protein | Role category not yet assigned | 1.31 | 0 | 0 | 0 | -2.66 |
| *NA* | lmo1790 | similar to unknown proteins | Role category not yet assigned | 1.3 | 0 | 0 | 0 | 1.49 |
| *NA* | lmo2403 | similar to B. subtilis YunD protein | Role category not yet assigned | 1.3 | 0 | 0 | 0 | -3.74 |
| *NA* | lmo1180 | similar to putative carboxysome structural protein | Role category not yet assigned | 1.26 | 0 | 0 | 0 | 0.31 |
| *NA* | lmo1336 | similar to 5-formyltetrahydrofolate cyclo-ligase | Role category not yet assigned | -4.9 | 0 | 0 | 0 | -0.05 |
| *tcsA* | lmo1388 | CD4+ T cell-stimulating antigen, lipoprotein | Role category not yet assigned | -4.45 | DOWN | 0 | 0 | 0.97 |
| *iap* | lmo0582 | P60 extracellular protein, invasion associated protein Iap | Role category not yet assigned | -3.9 | DOWN | 0 | 0 | 1.39 |
| *NA* | lmo1847 | similar to adhesion binding proteins and lipoproteins with multiple specificity for metal cations (ABC transporter) | Role category not yet assigned | -3.78 | 0 | 0 | 0 | -1 |
| *NA* | lmo1418 | weakly similar to pyrophosphatase | Role category not yet assigned | -3.71 | 0 | 0 | 0 | 0.83 |
| *NA* | LMOh7858_1619 | OB-fold nucleic acid binding domain protein | Role category not yet assigned | -3.19 | 0 | 0 | 0 | 0 |
| *NA* | lmo0282 | conserved hypothetical protein | Role category not yet assigned | -2.41 | 0 | 0 | 0 | 0.54 |
| *NA* | lmo1796 | similar to unknown protein | Role category not yet assigned | -2.3 | 0 | 0 | 0 | -0.52 |
| *NA* | lmo0285 | putative lipoprotein | Role category not yet assigned | -2.26 | 0 | 0 | 0 | -1.07 |
| *NA* | lmo0593 | similar to transport proteins (formate?) | Role category not yet assigned | -2.25 | UP | 0 | 1.5 | 0.95 |
| *NA* | lmo1814 | similar to unknown proteins | Role category not yet assigned | -2.19 | 0 | 0 | 0 | 0.37 |
| *NA* | lmo0661 | similar to unknown proteins | Role category not yet assigned | -2.17 | 0 | 0 | 0 | 1.42 |
| *bvrA* | lmo2788 | transcription antiterminator | Role category not yet assigned | -2.01 | 0 | 0 | 0 | 0.01 |
| *NA* | lmo1012 | similar to N-acyl-L-amino acid amidohydrolases | Role category not yet assigned | -1.98 | 0 | 0 | 0 | -1.91 |
| *NA* | lmo1909 | similar to unknown proteins | Role category not yet assigned | -1.69 | 0 | 0 | 0 | 0.52 |
| *NA* | lmo0664 | similar to acetyl transferase | Role category not yet assigned | -1.58 | DOWN | 0 | 0 | -1.31 |
| *NA* | lmo2142 | unknown | Role category not yet assigned | -1.52 | DOWN | 0 | -3.4 | -4.59 |
| *NA* | lmo1337 | similar to B. subtilis yqgP | Role category not yet assigned | -1.51 | 0 | 0 | 0 | 0.39 |
| *NA* | lmo2214 | similar to ABC transporter (membrane protein) | Role category not yet assigned | -1.51 | 0 | 0 | 0 | -0.32 |
| *NA* | lmo0111 | unknown | Role category not yet assigned | -1.46 | 0 | 0 | 0 | -1.29 |
| *NA* | lmo2051 | weakly similar to proteases | Role category not yet assigned | -1.42 | DOWN | 0 | 0 | -2.47 |
| *NA* | lmo0904 | unknown | Role category not yet assigned | -1.36 | UP | 0 | 0 | 0.79 |
| *NA* | lmo1001 | similar to B. subtilis protein YkvS | Role category not yet assigned | -1.31 | UP | 4.4 | 0 | 0.04 |
| *NA* | lmo0185 | similar to conserved hypothetical proteins | Role category not yet assigned | -1.29 | 0 | 0 | 0 | -0.97 |
| *NA* | lmo0386 | similar to B. subtilis IolD protein, to acetolactate synthase | Role category not yet assigned | 0 | UP | 0 | 1.3 | -1.33 |
| *NA* | lmo0555 | similar to di-tripeptide transporter | Role category not yet assigned | 0 | UP | 0 | 2.8 | -0.57 |
| *NA* | lmo0580 | weakly similar to carboxylesterase | Role category not yet assigned | 0 | UP | 0 | 2.2 | -3.57 |
| *NA* | lmo0265 | similar to succinyldiaminopimelate desuccinylase | Role category not yet assigned | 0 | UP | 0 | 1.1 | -1.4 |
| *NA* | lmo0607 | similar to ABC transporter, ATP-binding protein | Role category not yet assigned | 0 | UP | 0 | 2.1 | -2.21 |
| *NA* | lmo0608 | similar to ABC transporter, ATP-binding protein | Role category not yet assigned | 0 | UP | 0 | 3.3 | 0.99 |
| *NA* | lmo0760 | unknown | Role category not yet assigned | 0 | UP | 0 | 0 | -0.3 |
| *NA* | lmo0871 | similar to B. subtilis YtcD protein | Role category not yet assigned | 0 | UP | 0 | 0 | -1.78 |
| *NA* | lmo1220 | similar to unknown protein | Role category not yet assigned | 0 | UP | 0 | 0 | 0.37 |
| *cinA* | lmo1397 | similar to competence-damage inducible protein CinA | Role category not yet assigned | 0 | UP | 0 | 0 | -1.43 |
| *NA* | lmo1970 | similar to putative phosphotriesterase related proteins | Role category not yet assigned | 0 | UP | 0 | 0 | 0.83 |
| *NA* | lmo1998 | similar to opine catabolism protein | Role category not yet assigned | 0 | UP | 0 | 0 | 0.29 |
| *NA* | lmo1999 | weakly similar to glucosamine-fructose-6-phosphate aminotransferase | Role category not yet assigned | 0 | UP | 0 | 0 | 0.69 |
| *NA* | lmo2028 | similar to unknown proteins | Role category not yet assigned | 0 | UP | 0 | 0 | -0.42 |
| *NA* | lmo2029 | similar to unknown proteins | Role category not yet assigned | 0 | UP | 0 | 0 | 1.04 |
| *NA* | lmo2067 | similar to conjugated bile acid hydrolase | Role category not yet assigned | 0 | UP | 0 | 3.2 | 1.4 |
| *NA* | lmo2269 | unknown | Role category not yet assigned | 0 | UP | 2.21 | 2.5 | -0.97 |
| *NA* | lmo2298 | Protein gp4 [Bacteriophage A118] | Role category not yet assigned | 0 | UP | 0 | 0 | 1.17 |
| *NA* | lmo2323 | gp43 [Bacteriophage A118] | Role category not yet assigned | 0 | UP | 0 | 0 | -0.55 |
| *NA* | lmo2491 | unknown | Role category not yet assigned | 0 | UP | 0 | 0 | 0.53 |
| *NA* | lmo2602 | conserved hypothetical protein | Role category not yet assigned | 0 | UP | 0 | 1.4 | 1.36 |
| *NA* | lmo2670 | conserved hypothetical protein | Role category not yet assigned | 0 | UP | 0 | 2.3 | -0.03 |
| *NA* | lmo2671 | unknown | Role category not yet assigned | 0 | UP | 0 | 2.8 | -0.46 |
| *NA* | lmo0394 | similar to L. monocytogenes extracellular P60 protein | Role category not yet assigned | 0 | DOWN | 0 | -2.3 | 2.05 |
| *NA* | lmo0538 | similar to N-acyl-L-amino acid amidohydrolase | Role category not yet assigned | 0 | DOWN | 0 | 0 | 0.62 |
| *NA* | lmo1075 | similar to teichoic acid translocation ATP-binding protein TagH (ABC transporter) | Role category not yet assigned | 0 | DOWN | 0 | 0 | -1.54 |
| *NA* | lmo1430 | similar to unknown proteins | Role category not yet assigned | 0 | DOWN | 0 | 0 | 1.24 |
| *NA* | lmo1468 | similar to unknown proteins | Role category not yet assigned | 0 | DOWN | 0 | -2.5 | -0.92 |
| *NA* | lmo2102 | unknown | Role category not yet assigned | 0 | DOWN | 0 | 0 | -2.15 |
| *NA* | lmo2439 | unknown | Role category not yet assigned | 0 | DOWN | 0 | 0 | -3.03 |
| *NA* | lmo2522 | similar to hypothetical cell wall binding protein from B. subtilis | Role category not yet assigned | 0 | DOWN | 10.57 | -3.2 | 0.3 |
| *NA* | lmo2647 | similar to creatinine amidohydrolase | Role category not yet assigned | 0 | DOWN | 0 | 0 | 1.67 |
| *lmaA* | lmo0118 | antigen A | Role category not yet assigned | 0 | 0 | 2.2 | 0 | -0.95 |
| *NA* | lmo0189 | highly similar to B. subtilis Veg protein | Role category not yet assigned | 0 | 0 | 12.23 | 0 | -4.35 |
| *NA* | lmo0625 | unknown | Role category not yet assigned | 0 | 0 | 2.75 | 0 | -0.34 |
| *NA* | lmo2713 | secreted protein with 1 GW repeat | Role category not yet assigned | 0 | 0 | 2.5 | 0 | -2.32 |
| *NA* | lmo2766 | similar to hypothetical transcriptional regulator | Role category not yet assigned | 0 | 0 | 2.2 | 0 | 1.18 |
| *hly* | lmo0202 | listeriolysin O precursor | Role category not yet assigned | 0 | 0 | -20.74 | -1.1 | -0.59 |
| *NA* | lmo0354 | similar to fatty-acid--CoA ligase | Role category not yet assigned | 0 | 0 | -2.84 | 0 | 2.65 |
| *NA* | lmo0355 | similar to Flavocytochrome C Fumarate Reductase chain A | Role category not yet assigned | 0 | 0 | -9.22 | 0 | -0.69 |
| *NA* | lmo1013 | similar to conserved hypothetical proteins like to B. subtilis YkuT protein | Role category not yet assigned | 0 | 0 | -2.65 | 0 | -1.66 |
| *NA* | lmo1456 | similar to unknown proteins | Role category not yet assigned | 0 | 0 | -2.23 | 0 | 0.93 |
| *NA* | lmo2433 | similar to acetylesterase | Role category not yet assigned | 0 | 0 | -6.09 | 0 | -2.5 |
| *kat* | lmo2785 | catalase | Role category not yet assigned | 0 | 0 | -3.5 | 2.3 | 0.23 |
| *rsbV* | lmo0893 | anti-anti-sigma factor (antagonist of RsbW) | Role category not yet assigned | 0 | 0 | 0 | 2.2 | -1.77 |
| *rsbX* | lmo0896 | Indirect negative regulation of sigma B dependant gene expression (serine phosphatase) | Role category not yet assigned | 0 | 0 | 0 | 4.2 | -0.58 |
| *NA* | lmo1606 | similar to DNA translocase | Role category not yet assigned | 0 | 0 | 0 | 1.2 | 0.73 |
| *NA* | lmo0401 | highly similar to E. col YbgG protein, a putative sugar hydrolase | Role category not yet assigned | 0 | 0 | 0 | 1.1 | 0.82 |
| *dltA* | lmo0974 | D-alanine-activating enzyme (dae), D-alanine-D-alanyl carrier protein ligase (dcl) | Role category not yet assigned | 0 | 0 | 0 | -2.8 | -0.46 |
| *NA* | lmo0361 | similar to conserved hypothetical integral membrane protein | Role category not yet assigned | 0 | 0 | 0 | -1.3 | 0.14 |
| *NA* | lmo0362 | similar to conserved hypothetical protein | Role category not yet assigned | 0 | 0 | 0 | -3.6 | 0.94 |
| *NA* | lmo2182 | similar to ferrichrome ABC transporter (ATP-binding protein) | Role category not yet assigned | 0 | 0 | 0 | -2 | -1.12 |
| *NA* | lmo2180 | similar to unknown protein | Role category not yet assigned | 0 | 0 | 0 | -2.2 | -0.75 |
| *NA* | lmo2185 | unknown | Role category not yet assigned | 0 | 0 | 0 | -4.2 | 0.86 |
| *NA* | lmo2186 | unknown | Role category not yet assigned | 0 | 0 | 0 | -4.2 | -1.27 |
| *NA* | lmo0035 | similar to Glucosamine--fructose-6-phosphate aminotransferase (C-terminal domain) | Role category not yet assigned | 0 | 0 | 0 | 0 | 0.83 |
| *NA* | lmo0041 | conserved hypothetical protein, hypothetical regulator | Role category not yet assigned | 0 | 0 | 0 | 0 | -1.19 |
| *NA* | lmo0052 | highly similar to B. subtilis YybT protein | Role category not yet assigned | 0 | 0 | 0 | 0 | 2.88 |
| *NA* | lmo0102 | unknown | Role category not yet assigned | 0 | 0 | 0 | 0 | -0.88 |
| *NA* | lmo0107 | similar to ABC transporter, ATP-binding protein | Role category not yet assigned | 0 | 0 | 0 | 0 | -2.59 |
| *NA* | lmo0108 | similar to ABC transporter, ATP-binding protein | Role category not yet assigned | 0 | 0 | 0 | 0 | -1.05 |
| *NA* | lmo0130 | similar to 5-nucleotidase, putative peptidoglycan bound protein (LPXTG motif) | Role category not yet assigned | 0 | 0 | 0 | 0 | -1.02 |
| *NA* | lmo0139 | unknown | Role category not yet assigned | 0 | 0 | 0 | 0 | -0.09 |
| *NA* | lmo0175 | putative peptidoglycan bound protein (LPXTG motif) | Role category not yet assigned | 0 | 0 | 0 | 0 | -1.37 |
| *actA* | lmo0204 | actin-assembly inducing protein precursor | Role category not yet assigned | 0 | 0 | 0 | 0 | -0.32 |
| *NA* | lmo0208 | conserved hypothetical protein | Role category not yet assigned | 0 | 0 | 0 | 0 | -0.83 |
| *NA* | lmo0216 | highly similar to B. subtilis YabO protein | Role category not yet assigned | 0 | 0 | 0 | 0 | -1.31 |
| *NA* | lmo0230 | similar to B. subtilis YacH protein | Role category not yet assigned | 0 | 0 | 0 | 0 | 1.1 |
| *NA* | lmo0253 | similar to penicillinase antirepressor | Role category not yet assigned | 0 | 0 | 0 | 0 | -3.52 |
| *NA* | lmo0257 | similar to unknown protein | Role category not yet assigned | 0 | 0 | 0 | 0 | -0.99 |
| *NA* | lmo0267 | similar to other proteins | Role category not yet assigned | 0 | 0 | 0 | 0 | 0.23 |
| *NA* | lmo0289 | similar to B. subtilis YycH protein | Role category not yet assigned | 0 | 0 | 0 | 0 | -0.71 |
| *NA* | lmo0290 | similar to B. subtilis YycI protein | Role category not yet assigned | 0 | 0 | 0 | 0 | 0.98 |
| *NA* | lmo0326 | similar to transcriptional regulators | Role category not yet assigned | 0 | 0 | 0 | 0 | 0.16 |
| *NA* | lmo0336 | unknown | Role category not yet assigned | 0 | 0 | 0 | 0 | 0.11 |
| *NA* | lmo0367 | conserved hypothetical protein similar to B. subtilis YwbN protein | Role category not yet assigned | 0 | 0 | 0 | 0 | -0.32 |
| *NA* | lmo0368 | similar to different proteins | Role category not yet assigned | 0 | 0 | 0 | 0 | 1.59 |
| *NA* | lmo0383 | highly similar to B. subtilis methylmalonate-semialdehyde dehydrogenase IolA | Role category not yet assigned | 0 | 0 | 0 | 0 | 1.03 |
| *NA* | lmo0384 | similar to B. subtilis IolB protein | Role category not yet assigned | 0 | 0 | 0 | 0 | 0.45 |
| *NA* | lmo0385 | similar to B. subtilis IolC protein and to fructokinase | Role category not yet assigned | 0 | 0 | 0 | 0 | 0 |
| *NA* | lmo0429 | similar to sugar hydrolase | Role category not yet assigned | 0 | 0 | 0 | 0 | 2.02 |
| *NA* | lmo0431 | similar to acetyltransferase | Role category not yet assigned | 0 | 0 | 0 | 0 | -0.59 |
| *NA* | lmo0435 | putative peptidoglycan bound protein (LPXTG motif) | Role category not yet assigned | 0 | 0 | 0 | 0 | 1.15 |
| *NA* | lmo0452 | similar to unknown proteins | Role category not yet assigned | 0 | 0 | 0 | 0 | 0.93 |
| *NA* | lmo0453 | conserved hypothetical proteins | Role category not yet assigned | 0 | 0 | 0 | 0 | 1.04 |
| *NA* | lmo0476 | similar to oxetanocin A resistance protein oxrB | Role category not yet assigned | 0 | 0 | 0 | 0 | 0.2 |
| *NA* | lmo0512 | unknown | Role category not yet assigned | 0 | 0 | 0 | 0 | 2.56 |
| *NA* | lmo0522 | similar to transcription regulator | Role category not yet assigned | 0 | 0 | 0 | 0 | 1.97 |
| *NA* | lmo0533 | similar to unknown proteins | Role category not yet assigned | 0 | 0 | 0 | 0 | -0.07 |
| *NA* | lmo0553 | unknown | Role category not yet assigned | 0 | 0 | 0 | 0 | -0.09 |
| *NA* | lmo0562 | similar to phosphoribosyl-AMP cyclohydrolase (HisI1 protein) | Role category not yet assigned | 0 | 0 | 0 | 0 | -0.76 |
| *hisJ* | lmo0570 | similar histidinol phosphate phosphatase | Role category not yet assigned | 0 | 0 | 0 | 0 | 0.98 |
| *NA* | lmo0586 | unknown | Role category not yet assigned | 0 | 0 | 0 | 0 | 0.25 |
| *NA* | lmo0595 | similar to O-acetylhomoserine sulfhydrylase | Role category not yet assigned | 0 | 0 | 0 | 0 | -0.67 |
| *NA* | lmo0621 | conserved hypothetical protein | Role category not yet assigned | 0 | 0 | 0 | 0 | -0.51 |
| *NA* | lmo0732 | putative peptidoglycan bound protein (LPXTG motif) | Role category not yet assigned | 0 | 0 | 0 | 0 | 0.42 |
| *NA* | lmo0747 | unknown | Role category not yet assigned | 0 | 0 | 0 | 0 | -1.65 |
| *NA* | lmo0754 | weakly similar to a bile acid 7-alpha dehydratase | Role category not yet assigned | 0 | 0 | 0 | 0 | 0.63 |
| *NA* | lmo0755 | similar to unknown proteins | Role category not yet assigned | 0 | 0 | 0 | 0 | 0.07 |
| *NA* | lmo0757 | similar to hypothetical proteins | Role category not yet assigned | 0 | 0 | 0 | 0 | 0.05 |
| *NA* | lmo0768 | similar to sugar ABC transporter, periplasmic sugar-binding protein | Role category not yet assigned | 0 | 0 | 0 | 0 | -0.42 |
| *NA* | lmo0769 | similar to alpha-1,6-mannanase | Role category not yet assigned | 0 | 0 | 0 | 0 | 0.21 |
| *NA* | lmo0800 | similar to B. subtilis YqkB protein | Role category not yet assigned | 0 | 0 | 0 | 0 | 1.33 |
| *NA* | lmo0814 | similar to oxidoreductases | Role category not yet assigned | 0 | 0 | 0 | 0 | -1.83 |
| *nifJ* | lmo0829 | highly similar to pyruvate-flavodoxin oxidoreductase | Role category not yet assigned | 0 | 0 | 0 | 0 | -2.36 |
| *NA* | lmo0842 | putative peptidoglycan bound protein (LPXTG motif) | Role category not yet assigned | 0 | 0 | 0 | 0 | -0.31 |
| *NA* | lmo0912 | similar to transporters (formate) | Role category not yet assigned | 0 | 0 | 0 | 0 | 1.46 |
| *NA* | lmo0930 | conserved hypothetical protein, similar to B. subtilis YhfI protein | Role category not yet assigned | 0 | 0 | 0 | 0 | -1.75 |
| *NA* | lmo0945 | similar to C-terminal part of B. subtilis ComEC protein and to ComEA | Role category not yet assigned | 0 | 0 | 0 | 0 | -0.82 |
| *NA* | lmo0949 | conserved hypothetical membrane protein | Role category not yet assigned | 0 | 0 | 0 | 0 | -1 |
| *NA* | lmo0966 | unknown | Role category not yet assigned | 0 | 0 | 0 | 0 | -0.55 |
| *NA* | lmo0968 | similar to conserved hypothetical proteins like to B. subtilis YjbN protein | Role category not yet assigned | 0 | 0 | 0 | 0 | -1.45 |
| *NA* | lmo0991 | conserved hypothetical protein | Role category not yet assigned | 0 | 0 | 0 | 0 | -2.7 |
| *NA* | lmo1004 | conserved hypothetical protein | Role category not yet assigned | 0 | 0 | 0 | 0 | -1.48 |
| *NA* | lmo1018 | similar to E. coli copper homeostasis protein CutC | Role category not yet assigned | 0 | 0 | 0 | 0 | -1.43 |
| *NA* | lmo1050 | similar to B. subtilis YdfE protein | Role category not yet assigned | 0 | 0 | 0 | 0 | 1.77 |
| *NA* | lmo1077 | similar to teichoic acid biosynthesis protein B | Role category not yet assigned | 0 | 0 | 0 | 0 | -0.09 |
| *NA* | lmo1085 | similar to teichoic acid biosynthesis protein B | Role category not yet assigned | 0 | 0 | 0 | 0 | -0.42 |
| *tagB* | lmo1088 | similar to teichoic acid biosynthesis protein B precursor | Role category not yet assigned | 0 | 0 | 0 | 0 | 0.1 |
| *NA* | lmo1104 | highly similar to TN916 ORF14 and to L. monocytogenes P60 protein | Role category not yet assigned | 0 | 0 | 0 | 0 | -0.04 |
| *NA* | lmo1112 | highly similar to TN916 ORF21 | Role category not yet assigned | 0 | 0 | 0 | 0 | -0.17 |
| *NA* | lmo1115 | similar to fibrinogen-binding protein (LPXTG motif) | Role category not yet assigned | 0 | 0 | 0 | 0 | -0.61 |
| *NA* | lmo1143 | similar to Salmonella enterica PduT protein | Role category not yet assigned | 0 | 0 | 0 | 0 | 0.6 |
| *NA* | lmo1151 | similar to Salmonella typhimurium PduA protein | Role category not yet assigned | 0 | 0 | 0 | 0 | -0.73 |
| *NA* | lmo1152 | similar to Salmonella typhimurium PduB protein | Role category not yet assigned | 0 | 0 | 0 | 0 | 0.09 |
| *NA* | lmo1153 | highly similar to propanediol dehydratase, alpha subunit | Role category not yet assigned | 0 | 0 | 0 | 0 | -1.16 |
| *NA* | lmo1156 | similar to diol dehydratase-reactivating factor large subunit | Role category not yet assigned | 0 | 0 | 0 | 0 | -0.56 |
| *NA* | lmo1157 | similar to diol dehydratase-reactivating factor small chain | Role category not yet assigned | 0 | 0 | 0 | 0 | 0.72 |
| *NA* | lmo1158 | similar to Salmonella enterica PduK protein | Role category not yet assigned | 0 | 0 | 0 | 0 | -1.52 |
| *NA* | lmo1159 | similar to carboxysome structural protein | Role category not yet assigned | 0 | 0 | 0 | 0 | 0.64 |
| *NA* | lmo1160 | similar to Salmonella enterica PduL protein | Role category not yet assigned | 0 | 0 | 0 | 0 | 1.76 |
| *NA* | lmo1162 | unknown | Role category not yet assigned | 0 | 0 | 0 | 0 | 0.97 |
| *NA* | lmo1164 | hyghly similar to Salmonella enterica PduO protein | Role category not yet assigned | 0 | 0 | 0 | 0 | -0.33 |
| *NA* | lmo1170 | similar to Salmonella enterica PduX protein | Role category not yet assigned | 0 | 0 | 0 | 0 | 0.06 |
| *NA* | lmo1178 | similar to putative carboxysome structural protein | Role category not yet assigned | 0 | 0 | 0 | 0 | -0.14 |
| *NA* | lmo1185 | similar to Salmonella enterica PduT protein | Role category not yet assigned | 0 | 0 | 0 | 0 | -0.05 |
| *NA* | lmo1209 | similar to unknown protein | Role category not yet assigned | 0 | 0 | 0 | 0 | -0.45 |
| *NA* | lmo1232 | similar to MutS protein (MutS2) | Role category not yet assigned | 0 | 0 | 0 | 0 | 0.15 |
| *NA* | lmo1283 | similar to Lactococcus lactis LacX protein | Role category not yet assigned | 0 | 0 | 0 | 0 | 0.39 |
| *NA* | lmo1321 | conserved hypothetical protein, similar to B. subtilis YlxS protein | Role category not yet assigned | 0 | 0 | 0 | 0 | 1.13 |
| *NA* | lmo1341 | weakly similar to B. subtilis comG operon protein 7 (comGG) | Role category not yet assigned | 0 | 0 | 0 | 0 | -0.81 |
| *NA* | lmo1369 | similar to phosphotransbutyrylase | Role category not yet assigned | 0 | 0 | 0 | 0 | 0.18 |
| *NA* | lmo1399 | similar to unknown protein | Role category not yet assigned | 0 | 0 | 0 | 0 | 0.6 |
| *NA* | lmo1419 | conserved hypothetical protein | Role category not yet assigned | 0 | 0 | 0 | 0 | -1.13 |
| *NA* | lmo1443 | unknown | Role category not yet assigned | 0 | 0 | 0 | 0 | 1.55 |
| *NA* | lmo1466 | similar to unknown proteins | Role category not yet assigned | 0 | 0 | 0 | 0 | -0.21 |
| *NA* | lmo1510 | similar to unknown proteins | Role category not yet assigned | 0 | 0 | 0 | 0 | -0.06 |
| *NA* | lmo1575 | similar to unknown proteins | Role category not yet assigned | 0 | 0 | 0 | 0 | -0.42 |
| *NA* | lmo1586 | similar to unknown proteins | Role category not yet assigned | 0 | 0 | 0 | 0 | 1.04 |
| *NA* | lmo1595 | similar to unknown protein | Role category not yet assigned | 0 | 0 | 0 | 0 | -0.6 |
| *NA* | lmo1613 | similar to unknown proteins | Role category not yet assigned | 0 | 0 | 0 | 0 | 0.04 |
| *NA* | lmo1651 | similar to ABC transporter (ATP-binding protein) | Role category not yet assigned | 0 | 0 | 0 | 0 | 1.14 |
| *NA* | lmo1652 | similar to ABC transporter (ATP-binding protein) | Role category not yet assigned | 0 | 0 | 0 | 0 | -0.02 |
| *NA* | lmo1655 | similar to unknown proteins | Role category not yet assigned | 0 | 0 | 0 | 0 | 0.71 |
| *NA* | lmo1680 | similar to cystathionine gamma-synthase | Role category not yet assigned | 0 | 0 | 0 | 0 | 1.57 |
| *NA* | lmo1705 | similar to deoxyguanosine kinase/deoxyadenosine kinase(I) subunit | Role category not yet assigned | 0 | 0 | 0 | 0 | 0.41 |
| *NA* | lmo1714 | unknown | Role category not yet assigned | 0 | 0 | 0 | 0 | 1.14 |
| *NA* | lmo1777 | similar to unknown protein | Role category not yet assigned | 0 | 0 | 0 | 0 | -1.48 |
| *NA* | lmo1854 | similar to conserved hypothetical proteins | Role category not yet assigned | 0 | 0 | 0 | 0 | 1.18 |
| *NA* | lmo1862 | similar to hypothetical proteins | Role category not yet assigned | 0 | 0 | 0 | 0 | 1.17 |
| *dnaD* | lmo1895 | similar to chromosome replication initiation protein | Role category not yet assigned | 0 | 0 | 0 | 0 | 0.31 |
| *NA* | lmo1913 | similar to unknown proteins | Role category not yet assigned | 0 | 0 | 0 | 0 | 0.46 |
| *NA* | lmo1914 | similar to unknown proteins | Role category not yet assigned | 0 | 0 | 0 | 0 | 1.53 |
| *NA* | lmo1922 | similar to unknown proteins | Role category not yet assigned | 0 | 0 | 0 | 0 | 1.44 |
| *NA* | lmo1968 | similar to creatinine amidohydrolases | Role category not yet assigned | 0 | 0 | 0 | 0 | -0.29 |
| *NA* | lmo2015 | similar to alpha-mannosidase | Role category not yet assigned | 0 | 0 | 0 | 0 | -0.02 |
| *NA* | lmo2030 | similar to unknown proteins | Role category not yet assigned | 0 | 0 | 0 | 0 | 1.82 |
| *NA* | lmo2118 | similar to phosphoglucomutase | Role category not yet assigned | 0 | 0 | 0 | 0 | -2.06 |
| *NA* | lmo2125 | similar to maltose/maltodextrin ABC-transporter (binding protein) | Role category not yet assigned | 0 | 0 | 0 | 0 | -1.66 |
| *NA* | lmo2170 | similar to unknown proteins | Role category not yet assigned | 0 | 0 | 0 | 0 | -1.23 |
| *NA* | lmo2245 | similar to unknown proteins | Role category not yet assigned | 0 | 0 | 0 | 0 | -1.5 |
| *NA* | lmo2264 | similar to unknown proteins | Role category not yet assigned | 0 | 0 | 0 | 0 | -0.6 |
| *NA* | lmo2282 | protein gp21 [Bacteriophage A118] | Role category not yet assigned | 0 | 0 | 0 | 0 | -1.27 |
| *NA* | lmo2287 | putative tape-measure [Bacteriophage A118] | Role category not yet assigned | 0 | 0 | 0 | 0 | -0.53 |
| *NA* | lmo2294 | Protein gp9 [Bacteriophage A118] | Role category not yet assigned | 0 | 0 | 0 | 0 | 0.02 |
| *NA* | lmo2295 | Protein gp8 [Bacteriophage A118] | Role category not yet assigned | 0 | 0 | 0 | 0 | -1.15 |
| *NA* | lmo2300 | putative terminase large subunit from Bacteriophage A118 | Role category not yet assigned | 0 | 0 | 0 | 0 | 2.64 |
| *NA* | lmo2303 | Protein gp66 [Bacteriophage A118] | Role category not yet assigned | 0 | 0 | 0 | 0 | 0.03 |
| *NA* | lmo2304 | Bacteriophage A118 gp65 protein | Role category not yet assigned | 0 | 0 | 0 | 0 | -0.57 |
| *NA* | lmo2317 | similar to protein gp49 [Bacteriophage A118] | Role category not yet assigned | 0 | 0 | 0 | 0 | -0.36 |
| *NA* | lmo2322 | gp44 [Bacteriophage A118] | Role category not yet assigned | 0 | 0 | 0 | 0 | -1.11 |
| *NA* | lmo2324 | similar to anti-repressor [Bacteriophage A118] | Role category not yet assigned | 0 | 0 | 0 | 0 | -3.12 |
| *NA* | lmo2345 | conserved hypothetical protein | Role category not yet assigned | 0 | 0 | 0 | 0 | -0.46 |
| *NA* | lmo2348 | similar to amino acid ABC-transporter (permease) | Role category not yet assigned | 0 | 0 | 0 | 0 | -1.13 |
| *NA* | lmo2406 | similar to B. subtilis YunF protein | Role category not yet assigned | 0 | 0 | 0 | 0 | -1.02 |
| *NA* | lmo2417 | conserved lipoprotein (putative ABC transporter binding protein) | Role category not yet assigned | 0 | 0 | 0 | 0 | 1.07 |
| *NA* | lmo2423 | conserved hypothetical protein | Role category not yet assigned | 0 | 0 | 0 | 0 | -1.53 |
| *NA* | lmo2473 | conserved hypothetical protein | Role category not yet assigned | 0 | 0 | 0 | 0 | 0.46 |
| *NA* | lmo2479 | unknown | Role category not yet assigned | 0 | 0 | 0 | 0 | 0.52 |
| *NA* | lmo2508 | similar to conserved hypothetical proteins | Role category not yet assigned | 0 | 0 | 0 | 0 | 0.29 |
| *NA* | lmo2541 | similar to yeast translation initiation protein | Role category not yet assigned | 0 | 0 | 0 | 0 | -0.17 |
| *NA* | lmo2579 | conserved hypothetical protein | Role category not yet assigned | 0 | 0 | 0 | 0 | -0.11 |
| *NA* | lmo2587 | conserved hypothetical proteins | Role category not yet assigned | 0 | 0 | 0 | 0 | -0.82 |
| *NA* | lmo2640 | unknown | Role category not yet assigned | 0 | 0 | 0 | 0 | 1.27 |
| *NA* | lmo2648 | similar to Phosphotriesterase | Role category not yet assigned | 0 | 0 | 0 | 0 | 0.78 |
| *NA* | lmo2679 | similar to the two components sensor protein kdpD | Role category not yet assigned | 0 | 0 | 0 | 0 | 0.42 |
| *NA* | lmo2727 | similar to unknown proteins | Role category not yet assigned | 0 | 0 | 0 | 0 | 3.06 |
| *NA* | lmo2732 | unknown | Role category not yet assigned | 0 | 0 | 0 | 0 | 2.6 |
| *NA* | lmo2740 | unknown | Role category not yet assigned | 0 | 0 | 0 | 0 | 1.42 |
| *NA* | lmo2741 | similar to drug-efflux transporters | Role category not yet assigned | 0 | 0 | 0 | 0 | -0.17 |
| *NA* | lmo2742 | unknown | Role category not yet assigned | 0 | 0 | 0 | 0 | 0.56 |
| *NA* | lmo2745 | similar to ABC transporter (ATP-binding protein) | Role category not yet assigned | 0 | 0 | 0 | 0 | 2.34 |
| *NA* | lmo2751 | similar to ABC transporter, ATP-binding protein | Role category not yet assigned | 0 | 0 | 0 | 0 | -1.07 |
| *NA* | lmo2752 | similar to ABC transporter, ATP-binding protein | Role category not yet assigned | 0 | 0 | 0 | 0 | 0.85 |
| *NA* | lmo2759 | similar to unknown protein | Role category not yet assigned | 0 | 0 | 0 | 0 | -0.77 |
| *NA* | lmo2773 | similar to transcription antiterminator | Role category not yet assigned | 0 | 0 | 0 | 0 | 1.41 |
| *NA* | lmo2781 | similar to beta-glucosidase | Role category not yet assigned | 0 | 0 | 0 | 0 | -2.72 |
| *NA* | lmo2795 | similar to E. coli RpiR transcription regulator | Role category not yet assigned | 0 | 0 | 0 | 0 | 0.23 |
| *NA* | lmo2801 | similar to a putative N-acetylmannosamine-6-phosphate epimerase | Role category not yet assigned | 0 | 0 | 0 | 0 | -0.76 |
| *NA* | lmo2817 | similar to peptidases | Role category not yet assigned | 0 | 0 | 0 | 0 | -0.05 |
| *NA* | lmo2819 | similar to carboxypeptidase | Role category not yet assigned | 0 | 0 | 0 | 0 | 1.57 |
| **Amino acid biosynthesis** | | |  |  |  |  |  |  |
| *NA* | lmo2683 | similar to cellobiose phosphotransferase enzyme IIB component | Aromatic amino acid family | 2.72 | 0 | 0 | 0 | -1.77 |
| *NA* | lmo1720 | similar to phosphotransferase system (PTS) lichenan-specific enzyme IIB component | Aromatic amino acid family | 1.91 | 0 | 0 | 0 | 1.11 |
| *NA* | lmo2373 | similar to phosphotransferase system (PTS) beta-glucoside-specific enzyme IIB component | Aromatic amino acid family | 1.79 | 0 | 2.1 | 0 | -0.83 |
| *aroA* | LMOf2365_1952 | 3-phosphoshikimate 1-carboxyvinyltransferase | Aromatic amino acid family | 1.62 | 0 | 0 | 0 | 0 |
| *NA* | lmo2413 | similar to aminotransferase | Aspartate family | 1.57 | 0 | 0 | 0 | -1.4 |
| *NA* | lmo1435 | similar to dihydrodipicolinate synthase | Aspartate family | 1.49 | 0 | 0 | 0 | -0.48 |
| *tyrA* | LMOf6854_1983 | prephenate dehydrogenase | Aromatic amino acid family | 1.4 | 0 | 0 | 0 | 0 |
| *tyrA* | lmo1924 | similar to prephenate dehydrogenase | Aromatic amino acid family | 1.33 | 0 | 0 | 0 | 1.02 |
| *NA* | lmo0097 | similar to PTS system mannose-specific, factor IIC | Aromatic amino acid family | -6.17 | DOWN | -2.79 | 0 | -0.62 |
| *NA* | lmo0096 | similar to PTS system mannose-specific, factor IIAB | Aromatic amino acid family | -2.84 | DOWN | -2.74 | 0 | -0.9 |
| *hom* | LMOf6854_2608 | homoserine dehydrogenase | Aspartate family | -2.77 | 0 | 0 | 0 | 0 |
| *NA* | lmo0560 | similar to NADP-specific glutamate dehydrogenase | Glutamate family | -2.57 | 0 | 0 | -1.5 | -0.32 |
| *NA* | lmo1255 | similar to PTS system trehalose specific enzyme IIBC | Aromatic amino acid family | -2.48 | 0 | 0 | 0 | 1.46 |
| *glnA* | LMOf2365_1317 | glutamine synthetase, type I | Glutamate family | -2.47 | 0 | 0 | 0 | 0 |
| *NA* | lmo0098 | similar to PTS system mannose-specific, factor IID | Aromatic amino acid family | -2.36 | DOWN | -3.3 | 0 | -0.33 |
| *thrB* | lmo2545 | highly similar to homoserine kinase | Aspartate family | -2.34 | DOWN | 0 | 0 | -1.48 |
| *hom* | LMOf2365_2520 | homoserine dehydrogenase | Aspartate family | -2.21 | 0 | 0 | 0 | 0 |
| *glnA* | lmo1299 | highly similar to glutamine synthetases | Glutamate family | -2.2 | DOWN | -3.4 | 0 | 0.87 |
| *pheA* | LMOf6854_1584 | prephenate dehydratase | Aromatic amino acid family | -2.2 | 0 | 0 | 0 | 0 |
| *NA* | lmo1749 | similar to shikimate kinase | Aromatic amino acid family | -2.13 | 0 | 0 | 0 | 1.51 |
| *thrB* | LMOf6854_2606 | homoserine kinase | Aspartate family | -2.11 | 0 | 0 | 0 | 0 |
| *thrC* | lmo2546 | highly similar to threonine synthase | Aspartate family | -1.97 | DOWN | 0 | -2.1 | -1.68 |
| *ansB* | lmo1663 | similar to asparagine synthetase | Aspartate family | -1.93 | DOWN | 0 | 0 | 0.78 |
| *trpF* | lmo1629 | phosphoribosyl anthranilate isomerase | Aromatic amino acid family | -1.92 | UP | -2.79 | 0 | -0.38 |
| *NA* | LMOh7858_0595 | N-carbamoyl-L-amino acid amidohydrolase, putative | Other | -1.79 | 0 | 0 | 0 | 0 |
| *NA* | lmo2018 | similar to diaminopimelate epimerase | Aspartate family | -1.55 | DOWN | 0 | 0 | 0.27 |
| *NA* | lmo1734 | similar to glutamate synthase (large subunit) | Glutamate family | -1.52 | 0 | 0 | 0 | 0.22 |
| *NA* | lmo1437 | similar to aspartate-semialdehyde dehydrogenase | Aspartate family | -1.49 | 0 | 0 | 0 | -0.01 |
| *NA* | lmo2824 | similar to D-3-phosphoglycerate dehydrogenase | Serine family | -1.42 | 0 | 2.14 | 0 | 1.92 |
| *NA* | lmo1733 | similar to glutamate synthase (small subunit) | Glutamate family | -1.42 | 0 | 0 | 0 | 2.19 |
| *NA* | lmo0396 | similar to 1-pyrroline-5-carboxylate reductase (ProC) | Glutamate family | -1.39 | 0 | 0 | 0 | 1.64 |
| *aroF* | lmo1928 | similar to chorismate synthase | Aromatic amino acid family | -1.39 | 0 | 0 | 0 | -0.57 |
| *NA* | lmo0978 | similar to branched-chain amino acid aminotransferase | Pyruvate family | -1.38 | 0 | 0 | 0 | -1.55 |
| *NA* | lmo1490 | similar to shikimate 5-dehydrogenase (AroD) | Aromatic amino acid family | -1.36 | DOWN | 0 | 0 | 1.91 |
| *leuD* | LMOf2365_2013 | 3-isopropylmalate dehydratase, small subunit | Pyruvate family | -1.36 | 0 | 0 | 0 | 0 |
| *serC* | LMOf2365_2816 | phosphoserine aminotransferase | Serine family | -1.26 | 0 | 0 | 0 | 0 |
| *proB* | lmo1260 | gamma-glutamyl kinase | Glutamate family | -1.24 | 0 | 0 | 0 | 0.5 |
| *NA* | lmo0021 | similar to PTS system, fructose-specific IIA component | Aromatic amino acid family | 0 | UP | 0 | 0 | 0.51 |
| *NA* | lmo0781 | similar to mannose-specific phosphotransferase system (PTS) component IID | Aromatic amino acid family | 0 | UP | 0 | 2.9 | -0.54 |
| *NA* | lmo1513 | similar to iron-sulfur cofactor synthesis protein | Aspartate family | 0 | UP | 0 | 0 | -0.35 |
| *trpB* | lmo1628 | highly similar to tryptophan synthase (beta subunit) | Aromatic amino acid family | 0 | UP | -1.15 | 0 | -0.03 |
| *trpD* | lmo1631 | highly similar to anthranilate phosphoribosyltransferase | Aromatic amino acid family | 0 | UP | -2.39 | 0 | -0.13 |
| *NA* | lmo1971 | similar to pentitol PTS system enzyme II C component | Aromatic amino acid family | 0 | UP | 0 | 0 | 0.87 |
| *NA* | lmo1973 | similar to PTS system enzyme II A component | Aromatic amino acid family | 0 | UP | 0 | 0 | 0.8 |
| *leuD* | lmo1990 | similar to 3-isopropylmalate dehydratase (small subunit) | Pyruvate family | 0 | UP | 2.72 | 1.2 | -0.59 |
| *ilvA* | lmo1991 | similar to threonine dehydratase | Pyruvate family | 0 | UP | 2.54 | 1.2 | -0.44 |
| *NA* | lmo2000 | similar to PTS mannose-specific enzyme IID component | Aromatic amino acid family | 0 | UP | 0 | 0 | 1.38 |
| *NA* | lmo2001 | similar to PTS mannose-specific enzyme IIC component | Aromatic amino acid family | 0 | UP | 0 | 0 | -0.63 |
| *NA* | lmo2002 | similar to PTS mannose-specific enzyme IIB component | Aromatic amino acid family | 0 | UP | 0 | 0 | 0.12 |
| *fruA* | lmo2335 | highly similar to phosphotransferase system (PTS) fructose-specific enzyme IIABC component | Aromatic amino acid family | 0 | UP | 13.03 | 0 | 0.19 |
| *NA* | lmo2772 | similar to beta-glucoside-specific enzyme IIABC | Aromatic amino acid family | 0 | UP | 0 | 0 | 0.54 |
| *NA* | lmo2797 | similar to phosphotransferase system mannitol-specific enzyme IIA | Aromatic amino acid family | 0 | UP | 0 | 0 | -0.5 |
| *serC* | lmo2825 | highly similar to phosphoserine aminotransferase | Serine family | 0 | UP | 0 | 0 | -0.65 |
| *NA* | lmo1387 | similar to pyrroline-5-carboxylate reductase | Glutamate family | 0 | DOWN | 0 | 2 | 0.96 |
| *NA* | lmo1536 | similar to prephenate dehydratase PheA | Aromatic amino acid family | 0 | DOWN | 0 | 0 | -0.14 |
| *aroA* | lmo1600 | 3-deoxy-D-arabino-heptulosonate 7-phosphate synthase | Aromatic amino acid family | 0 | DOWN | 0 | -2 | -0.48 |
| *NA* | lmo2097 | similar to PTS system galactitol-specific enzyme IIB component | Aromatic amino acid family | 0 | DOWN | 0 | 0 | 2.62 |
| *NA* | lmo2524 | similar to hydroxymyristoyl-(acyl carrier protein) dehydratase | Pyruvate family | 0 | DOWN | 0 | 0 | 2.15 |
| *hom* | lmo2547 | highly similar to homoserine dehydrogenase | Aspartate family | 0 | DOWN | 0 | -2 | 0.07 |
| *NA* | lmo2650 | similar to hypothetical PTS enzyme IIB component | Aromatic amino acid family | 0 | DOWN | 0 | 0 | -0.26 |
| *ilvD* | lmo1983 | similar to dihydroxy-acid dehydratase | Pyruvate family | 0 | 0 | 1.6 | 0 | 1.93 |
| *ilvB* | lmo1984 | similar to acetolactate synthase (acetohydroxy-acid synthase) (large subunit) | Pyruvate family | 0 | 0 | 3.43 | 0 | 0.78 |
| *ilvN* | lmo1985 | similar to acetolactate synthase (acetohydroxy-acid synthase) (small subunit) | Pyruvate family | 0 | 0 | 4.39 | 1.1 | 0.12 |
| *ilvC* | lmo1986 | similar to ketol-acid reductoisomerase (acetohydroxy-acid isomeroreductase) | Pyruvate family | 0 | 0 | 3.55 | 0 | 2 |
| *leuA* | lmo1987 | similar to 2-isopropylmalate synthase | Pyruvate family | 0 | 0 | 1.5 | 0 | 0.88 |
| *leuB* | lmo1988 | similar to 3-isopropylmalate dehydrogenase | Pyruvate family | 0 | 0 | 2.38 | 1.2 | 1 |
| *leuC* | lmo1989 | similar to 3-isopropylmalate dehydratase (large subunit) | Pyruvate family | 0 | 0 | 3.5 | 1.1 | -0.73 |
| *cysK* | lmo0223 | highly similar to cysteine synthase | Serine family | 0 | 0 | -4.92 | 0 | 1.68 |
| *trpE* | lmo1633 | highly similar to anthranilate synthase alpha subunit | Aromatic amino acid family | 0 | 0 | -1.54 | 0 | 2.12 |
| *trpG* | lmo1632 | highly similar to anthranilate synthase beta subunit | Aromatic amino acid family | 0 | 0 | -2.35 | 0 | -0.92 |
| *trpC* | lmo1630 | highly similar to indol-3-glycerol phosphate synthases | Aromatic amino acid family | 0 | 0 | -2.68 | 0 | -0.79 |
| *trpA* | lmo1627 | highly similar to tryptophan synthase (alpha subunit) | Aromatic amino acid family | 0 | 0 | -1.81 | 0 | 0.3 |
| *alsS* | lmo2006 | similar to alpha-acetolactate synthase protein, AlsS | Pyruvate family | 0 | 0 | -2.21 | 0 | -0.66 |
| *NA* | lmo2652 | similar to transcriptional antiterminator | Aromatic amino acid family | 0 | 0 | -2.32 | 0 | 0.48 |
| *NA* | lmo0027 | similar to PTS system, beta-glucosides specific enzyme IIABC | Aromatic amino acid family | 0 | 0 | 0 | -2 | 0.58 |
| *NA* | lmo0503 | similar to PTS fructose-specific enzyme IIA component | Aromatic amino acid family | 0 | 0 | 0 | 1.4 | -0.58 |
| *NA* | lmo0022 | similar to PTS system, fructose-specific IIB component | Aromatic amino acid family | 0 | 0 | 0 | 0 | 1.08 |
| *NA* | lmo0023 | similar to PTS system, fructose-specific IIC component | Aromatic amino acid family | 0 | 0 | 0 | 0 | 2.84 |
| *NA* | lmo0024 | similar to PTS system, mannose-specific IID component | Aromatic amino acid family | 0 | 0 | 0 | 0 | 1.07 |
| *NA* | lmo0036 | similar to ornithine carbamoyltransferase | Glutamate family | 0 | 0 | 0 | 0 | 2.81 |
| *cysE* | lmo0238 | similar to serine O-acetyltransferase | Serine family | 0 | 0 | 0 | 0 | 0.72 |
| *NA* | lmo0299 | similar to PTS beta-glucoside-specific enzyme IIB component | Aromatic amino acid family | 0 | 0 | 0 | 0 | 1.47 |
| *NA* | lmo0301 | similar to PTS beta-glucoside-specific enzyme IIA component | Aromatic amino acid family | 0 | 0 | 0 | 0 | 0.34 |
| *NA* | lmo0490 | similar to shikimate 5-dehydrogenase | Aromatic amino acid family | 0 | 0 | 0 | 0 | 3.32 |
| *NA* | lmo0491 | similar to 3-dehydroquinate dehydratase | Aromatic amino acid family | 0 | 0 | 0 | 0 | 0.74 |
| *NA* | lmo0508 | similar to PTS system, Galactitol-specific IIC component | Aromatic amino acid family | 0 | 0 | 0 | 0 | -0.03 |
| *NA* | lmo0542 | similar to PTS system, glucitol/sorbitol-specific enzyme IIA component | Aromatic amino acid family | 0 | 0 | 0 | 0 | 3.44 |
| *NA* | lmo0543 | similar to PTS system, glucitol/sorbitol-specific enzyme IIBC component | Aromatic amino acid family | 0 | 0 | 0 | 0 | 0.48 |
| *NA* | lmo0544 | similar to PTS system, glucitol/sorbitol-specific enzyme II CII component | Aromatic amino acid family | 0 | 0 | 0 | 0 | -0.13 |
| *NA* | lmo0561 | similar to phosphorybosil-AMP-cyclohydrolase (HisI2 protein) | Histidine family | 0 | 0 | 0 | 0 | -1.52 |
| *hisF* | lmo0563 | highly similar to cyclase HisF | Histidine family | 0 | 0 | 0 | 0 | 0.26 |
| *hisA* | lmo0564 | highly similar to phosphoribosylformimino-5-aminoimidazole carboxamide ribotide isomerase | Histidine family | 0 | 0 | 0 | 0 | 2.61 |
| *hisH* | lmo0565 | similar to amidotransferases | Histidine family | 0 | 0 | 0 | 0 | 1.55 |
| *hisB* | lmo0566 | imidazoleglycerol-phosphate dehydratase | Histidine family | 0 | 0 | 0 | 0 | 0.34 |
| *hisD* | lmo0567 | highly similar to histidinol dehydrogenases | Histidine family | 0 | 0 | 0 | 0 | 1.19 |
| *hisG* | lmo0568 | similar to ATP phosphoribosyltransferase | Histidine family | 0 | 0 | 0 | 0 | -0.67 |
| *NA* | lmo0594 | similar to homoserine O-acetyltransferase | Aspartate family | 0 | 0 | 0 | 0 | -0.19 |
| *NA* | lmo0738 | similar to phosphotransferase system (PTS) beta-glucoside-specific enzyme IIABC component | Aromatic amino acid family | 0 | 0 | 0 | 0 | 0.9 |
| *NA* | lmo0875 | similar to PTS system, beta-glucoside enzyme IIB component | Aromatic amino acid family | 0 | 0 | 0 | 0 | -1.86 |
| *NA* | lmo0876 | similar to PTS system, Lichenan-specific enzyme IIC component | Aromatic amino acid family | 0 | 0 | 0 | 0 | 0.1 |
| *NA* | lmo0914 | similar to PTS system, IIB component | Aromatic amino acid family | 0 | 0 | 0 | 0 | -1 |
| *NA* | lmo0915 | similar to phosphotransferase system enzyme IIC | Aromatic amino acid family | 0 | 0 | 0 | 0 | -1.17 |
| *NA* | lmo1011 | similar to tetrahydrodipicolinate succinylase | Aspartate family | 0 | 0 | 0 | 0 | -1.71 |
| *NA* | lmo1035 | similar to phosphotransferase system (PTS) beta-glucoside-specific enzyme IIABC | Aromatic amino acid family | 0 | 0 | 0 | 0 | -0.55 |
| *NA* | lmo1095 | similar to PTS system, cellobiose-specific IIB component (cel A) | Aromatic amino acid family | 0 | 0 | 0 | 0 | -0.94 |
| *NA* | lmo1141 | similar to uroporphyrin-III C-methyltransferase | Aromatic amino acid family | 0 | 0 | 0 | 0 | -0.18 |
| *NA* | lmo1154 | similar to diol dehydrase (diol dehydratase) gamma subunit | Pyruvate family | 0 | 0 | 0 | 0 | -0.13 |
| *NA* | lmo1235 | similar to aspartokinase II alpha subunit | Aspartate family | 0 | 0 | 0 | 0 | -2.35 |
| *proA* | lmo1259 | gamma-glutamyl phosphate reductase | Glutamate family | 0 | 0 | 0 | 0 | 1.25 |
| *NA* | lmo1436 | similar to aspartokinase I (alpha and beta subunits) | Aspartate family | 0 | 0 | 0 | 0 | 0.92 |
| *argF* | lmo1587 | highly similar to ornithine carbamoyltransferase | Glutamate family | 0 | 0 | 0 | 0 | -0.2 |
| *argD* | lmo1588 | highly similar to N-acetylornithine aminotransferase | Glutamate family | 0 | 0 | 0 | 0 | 0.61 |
| *argB* | lmo1589 | highly similar to N-acetylglutamate 5-phosphotransferase | Glutamate family | 0 | 0 | 0 | 0 | -0.58 |
| *argJ* | lmo1590 | highly similar to ornithine acetyltransferase and amino-acid acetyltransferases | Glutamate family | 0 | 0 | 0 | 0 | 0.3 |
| *argC* | lmo1591 | similar to N-acetylglutamate gamma-semialdehyde dehydrogenases | Glutamate family | 0 | 0 | 0 | 0 | 0.26 |
| *NA* | lmo1593 | similar to iron-sulfur cofactor synthesis protein nifS | Aspartate family | 0 | 0 | 0 | 0 | -0.37 |
| *NA* | lmo1681 | similar to cobalamin-independent methionine synthase | Aspartate family | 0 | 0 | 0 | 0 | 1.02 |
| *NA* | lmo1719 | similar to phosphotransferase system (PTS) lichenan-specific enzyme IIA component | Aromatic amino acid family | 0 | 0 | 0 | 0 | 1.67 |
| *NA* | lmo1872 | similar to methyltransferases | Serine family | 0 | 0 | 0 | 0 | -1.52 |
| *aspB* | lmo1897 | similar to aspartate aminotransferases | Aspartate family | 0 | 0 | 0 | 0 | 1.38 |
| *dapB* | lmo1907 | similar to dihydrodipicolinate reductase | Aspartate family | 0 | 0 | 0 | 0 | -0.9 |
| *aroE* | lmo1923 | similar to 5-enolpyruvylshikimate-3-phosphate synthase | Aromatic amino acid family | 0 | 0 | 0 | 0 | 0.9 |
| *hisC* | lmo1925 | similar to histidinol-phosphate aminotransferase and tyrosine/phenylalanine aminotransferase | Histidine family | 0 | 0 | 0 | 0 | -2.78 |
| *NA* | lmo1926 | similar to chorismate mutase | Aromatic amino acid family | 0 | 0 | 0 | 0 | -0.05 |
| *aroB* | lmo1927 | similar to 3-dehydroquinate synthase | Aromatic amino acid family | 0 | 0 | 0 | 0 | 0.17 |
| *lysA* | lmo1952 | similar to diaminopimelate decarboxylase | Aspartate family | 0 | 0 | 0 | 0 | 1.69 |
| *argG* | lmo2090 | similar to argininosuccinate synthase | Glutamate family | 0 | 0 | 0 | 0 | -1.27 |
| *argH* | lmo2091 | similar to argininosuccinate lyase | Glutamate family | 0 | 0 | 0 | 0 | -0.73 |
| *NA* | lmo2096 | similar to PTS system galactitol-specific enzyme IIC component | Aromatic amino acid family | 0 | 0 | 0 | 0 | 0.32 |
| *NA* | lmo2098 | similar to PTS system galactitol-specific enzyme IIA component | Aromatic amino acid family | 0 | 0 | 0 | 0 | -1.94 |
| *NA* | lmo2122 | similar to maltodextrose utilization protein MalA | Pyruvate family | 0 | 0 | 0 | 0 | -1.62 |
| *NA* | lmo2135 | similar to PTS system, fructose-specific enzyme IIC component | Aromatic amino acid family | 0 | 0 | 0 | 0 | 0.08 |
| *NA* | lmo2236 | similar to oxidoreductase | Aromatic amino acid family | 0 | 0 | 0 | 0 | -0.5 |
| *NA* | lmo2252 | similar to aspartate aminotransferase | Aspartate family | 0 | 0 | 0 | 0 | 0.22 |
| *NA* | lmo2374 | similar to aspartate kinase | Aspartate family | 0 | 0 | 0 | 0 | -1.28 |
| *NA* | lmo2665 | similar to PTS system galactitol-specific enzyme IIC component | Aromatic amino acid family | 0 | 0 | 0 | 0 | 1.29 |
| *NA* | lmo2666 | similar to PTS system galactitol-specific enzyme IIB component | Aromatic amino acid family | 0 | 0 | 0 | 0 | 2.58 |
| *NA* | lmo2667 | similar to PTS system galactitol-specific enzyme IIA component | Aromatic amino acid family | 0 | 0 | 0 | 0 | 1.15 |
| *NA* | lmo2685 | similar to cellobiose phosphotransferase enzyme IIA component | Aromatic amino acid family | 0 | 0 | 0 | 0 | -0.29 |
| *NA* | lmo2750 | similar to para-aminobenzoate synthase component I | Aromatic amino acid family | 0 | 0 | 0 | 0 | -2.41 |
| *NA* | lmo2762 | similar to PTS cellobiose-specific enzyme IIB | Aromatic amino acid family | 0 | 0 | 0 | 0 | 1.16 |
| *NA* | lmo2780 | similar to cellobiose PTS enzyme IIA | Aromatic amino acid family | 0 | 0 | 0 | 0 | -0.47 |
| *NA* | lmo2782 | similar to PTS, cellobiose-specific IIB component | Aromatic amino acid family | 0 | 0 | 0 | 0 | 0.46 |
| *NA* | lmo2783 | similar to cellobiose phosphotransferase system enzyme IIC | Aromatic amino acid family | 0 | 0 | 0 | 0 | 0 |
| *bvrB* | lmo2787 | beta-glucoside-specific phosphotransferase enzyme II ABC component | Aromatic amino acid family | 0 | 0 | 0 | 0 | 0.64 |
| **Fatty acid and phospholipid metabolism** | | |  |  |  |  |  |  |
| *NA* | LMOf6854_2814 | dihydroxyacetone kinase, Dak1 subunit, putative | Biosynthesis | 5.79 | 0 | 0 | 0 | 0 |
| *NA* | LMOf6854_2815 | dihydroxyacetone kinase, Dak2 subunit, putative | Biosynthesis | 5.21 | 0 | 0 | 0 | 0 |
| *NA* | lmo2695 | similar to dihydroxyacetone kinase | Biosynthesis | 4.55 | 0 | 0 | 7 | -1.13 |
| *NA* | LMOf2365_2674 | putative dihydroxyacetone kinase, Dak1 subunit | Biosynthesis | 3.18 | 0 | 0 | 0 | 0 |
| *NA* | lmo1414 | similar to Acetyl-CoA:acetyltransferase | Other | 1.87 | 0 | 0 | 0 | -0.25 |
| *cdsA* | LMOf2365_1333 | phosphatidate cytidylyltransferase | Biosynthesis | 1.71 | 0 | 0 | 0 | 0 |
| *NA* | lmo1381 | unknown | Other | 1.7 | 0 | 0 | 0 | 1.85 |
| *NA* | LMOf6854_2150 | lipase | Degradation | 1.64 | 0 | 0 | 0 | 0 |
| *NA* | LMOf2365_1413 | putative 3-ketoacyl-acyl carrier protein reductase | Biosynthesis | 1.62 | 0 | 0 | 0 | 0 |
| *cdsA* | lmo1316 | similar to phosphatidate cytidylyltransferase (CDP-diglyceride synthase) | Biosynthesis | 1.6 | 0 | 0 | 0 | 0.68 |
| *NA* | lmo1357 | acetyl-CoA carboxylase subunit (biotin carboxylase subunit) | Biosynthesis | 1.34 | 0 | 0 | 0 | 0.22 |
| *NA* | lmo0786 | similar to acyl-carrier protein phosphodiesterase and to NAD(P)H dehydrogenase | Other | -4.02 | 0 | 0 | 0 | -1.46 |
| *NA* | lmo2202 | similar to 3-oxoacyl- acyl-carrier protein synthase | Biosynthesis | -3.61 | DOWN | 0 | 0 | -1.67 |
| *acpA* | lmo1806 | highly similar to acyl carrier proteins | Biosynthesis | -2.93 | DOWN | 0 | -2 | -3.11 |
| *NA* | lmo2201 | similar to 3-oxoacyl-acyl-carrier protein synthase | Biosynthesis | -2.72 | DOWN | 0 | 0 | -0.63 |
| *fabF* | LMOf2365_2234 | beta-ketoacyl-acyl carrier protein synthase II | Biosynthesis | -2.36 | 0 | 0 | 0 | 0 |
| *fabD* | lmo1808 | similar to malonyl CoA-acyl carrier protein transacylase | Biosynthesis | -2.26 | 0 | 0 | 0 | 1.18 |
| *NA* | lmo0616 | C-terminal domain similar to glycerophosphoryl diester phosphodiesterase | Degradation | -2.15 | 0 | 0 | 0 | 0.05 |
| *NA* | lmo0970 | similar to enoyl- acyl-carrier protein reductase | Biosynthesis | -1.87 | 0 | 0 | 0 | -1.52 |
| *NA* | lmo0494 | weakly similar to esterase | Degradation | -1.79 | 0 | 0 | 0 | 0.22 |
| *plsX* | lmo1809 | similar to plsX protein involved in fatty acid/phospholipid synthesis | Biosynthesis | -1.73 | 0 | 0 | 0 | 1.74 |
| *accA* | LMOf6854_1624 | acetyl-CoA carboxylase, carboxyl transferase, alpha subunit | Biosynthesis | -1.52 | 0 | 0 | 0 | 0 |
| *NA* | lmo0611 | similar to acyl-carrier protein phosphodiesterase and NAD(P)H dehydrogenase | Other | -1.51 | DOWN | 0 | 0 | 0.17 |
| *accA* | lmo1572 | highly similar to acetyl CoA carboxylase (alpha subunit) | Biosynthesis | -1.34 | 0 | 0 | 0 | -0.5 |
| *accA* | LMOf2365_1594 | acetyl-CoA carboxylase, carboxyl transferase, alpha subunit | Biosynthesis | -1.26 | 0 | 0 | 0 | 0 |
| *NA* | lmo0110 | similar to lipase | Degradation | 0 | UP | 0 | 0 | -2.32 |
| *plcA* | lmo0201 | phosphatidylinositol-specific phospholipase c | Degradation | 0 | UP | -8.34 | 0 | -1.13 |
| *NA* | lmo0348 | similar to dihydroxyacetone kinase | Biosynthesis | 0 | UP | 0 | 1.1 | 1.02 |
| *NA* | lmo1292 | similar to glycerophosphodiester phosphodiesterase | Degradation | 0 | UP | 0 | 0 | 0.05 |
| *NA* | lmo2471 | similar to NADH oxidase | Degradation | 0 | UP | 0 | 2.2 | 1.34 |
| *NA* | lmo1396 | similar to phosphatidylglycerophosphate synthase | Biosynthesis | 0 | DOWN | 0 | 0 | 0.32 |
| *NA* | lmo1415 | similar to hydroxy-3-methylglutaryl coenzyme A synthase | Biosynthesis | 0 | DOWN | 0 | 0 | 1.37 |
| *plcB* | lmo0205 | phospholipase C | Degradation | 0 | 0 | -5.32 | 0 | -0.93 |
| *NA* | lmo0347 | similar to dihydroxyacetone kinase | Biosynthesis | 0 | 0 | 0 | 1 | 1.19 |
| *NA* | lmo2696 | similar to hypothetical dihydroxyacetone kinase | Biosynthesis | 0 | 0 | 0 | 6.5 | 0.44 |
| *NA* | lmo1688 | similar to glucose 1-dehydrogenase | Biosynthesis | 0 | 0 | 0 | 1.3 | 0.51 |
| *NA* | lmo1688 | similar to glucose 1-dehydrogenase | Degradation | 0 | 0 | 0 | 1.3 | 0.51 |
| *NA* | lmo0008 | similar to cardiolipin synthase | Biosynthesis | 0 | 0 | 0 | 0 | 0.37 |
| *NA* | lmo0764 | similar to lipoate-protein ligase | Other | 0 | 0 | 0 | 0 | 0.19 |
| *NA* | lmo0885 | similar to holo-acyl-carrier protein synthase | Biosynthesis | 0 | 0 | 0 | 0 | 1.1 |
| *NA* | lmo0931 | similar to lipoate protein ligase A | Other | 0 | 0 | 0 | 0 | -0.5 |
| *NA* | lmo1161 | similar to ethanolamine utilization protein EutJ | Degradation | 0 | 0 | 0 | 0 | 0.29 |
| *NA* | lmo1165 | similar to ethanolamine utilization protein EutE | Degradation | 0 | 0 | 0 | 0 | -0.81 |
| *eutB* | lmo1175 | similar to ethanolamine ammonia-lyase, heavy chain | Biosynthesis | 0 | 0 | 0 | 0 | -2.05 |
| *eutB* | lmo1175 | similar to ethanolamine ammonia-lyase, heavy chain | Degradation | 0 | 0 | 0 | 0 | -2.05 |
| *eutC* | lmo1176 | similar to ethanolamine ammonia-lyase, light chain | Biosynthesis | 0 | 0 | 0 | 0 | -0.23 |
| *eutC* | lmo1176 | similar to ethanolamine ammonia-lyase, light chain | Degradation | 0 | 0 | 0 | 0 | -0.23 |
| *NA* | lmo1177 | similar to putative carboxysome structural protein (eutL) | Biosynthesis | 0 | 0 | 0 | 0 | 1.2 |
| *NA* | lmo1177 | similar to putative carboxysome structural protein (eutL) | Degradation | 0 | 0 | 0 | 0 | 1.2 |
| *NA* | lmo1186 | similar to ethanolamine utilization protein EutH - Escherichia coli | Biosynthesis | 0 | 0 | 0 | 0 | -0.18 |
| *NA* | lmo1186 | similar to ethanolamine utilization protein EutH - Escherichia coli | Degradation | 0 | 0 | 0 | 0 | -0.18 |
| *NA* | lmo1356 | similar to acetyl-CoA carboxylase subunit (biotin carboxyl carrier subunit) | Biosynthesis | 0 | 0 | 0 | 0 | 0.47 |
| *NA* | lmo1394 | similar to 3-ketoacyl-acyl carrier protein reductase | Biosynthesis | 0 | 0 | 0 | 0 | -2.8 |
| *NA* | lmo1394 | similar to 3-ketoacyl-acyl carrier protein reductase | Degradation | 0 | 0 | 0 | 0 | -2.8 |
| *NA* | lmo1464 | similar to diacylglycerol kinase | Biosynthesis | 0 | 0 | 0 | 0 | 0.3 |
| *accD* | lmo1573 | highly similar to acetyl-CoA carboxylase beta subunit | Biosynthesis | 0 | 0 | 0 | 0 | -0.05 |
| *NA* | lmo1647 | similar to 1-acylglycerol-3-phosphate O-acyltransferases | Biosynthesis | 0 | 0 | 0 | 0 | 0.72 |
| *NA* | lmo1729 | similar to beta-glucosidases | Degradation | 0 | 0 | 0 | 0 | -0.93 |
| *fabG* | lmo1807 | similar to 3-ketoacyl-acyl carrier protein reductase | Biosynthesis | 0 | 0 | 0 | 0 | 0.27 |
| *NA* | lmo1946 | similar to similar to acyl-CoA hydrolase | Degradation | 0 | 0 | 0 | 0 | -0.61 |
| *NA* | lmo2089 | similar to lipases | Degradation | 0 | 0 | 0 | 0 | 2.27 |
| *NA* | lmo2175 | similar to dehydrogenase | Biosynthesis | 0 | 0 | 0 | 0 | 0.32 |
| *NA* | lmo2175 | similar to dehydrogenase | Degradation | 0 | 0 | 0 | 0 | 0.32 |
| *NA* | lmo2450 | similar to carboxylesterase | Degradation | 0 | 0 | 0 | 0 | -1.74 |
| *NA* | lmo2452 | similar to carboxylesterase | Degradation | 0 | 0 | 0 | 0 | 0.24 |
| *NA* | lmo2503 | similar to cardiolipin synthase | Biosynthesis | 0 | 0 | 0 | 0 | -0.79 |
| *NA* | lmo2566 | unknown | Other | 0 | 0 | 0 | 0 | 0.59 |
| *NA* | lmo2815 | similar to reductases | Biosynthesis | 0 | 0 | 0 | 0 | -3.31 |
| *NA* | lmo2815 | similar to reductases | Degradation | 0 | 0 | 0 | 0 | -3.31 |
| **Central intermediary metabolism** | | |  |  |  |  |  |  |
| *NA* | lmo0820 | some similarity to acatyltransferases | Other | 4.7 | UP | 0 | 0 | -0.97 |
| *NA* | lmo1870 | similar to alkaline phosphatase | Other | 4.22 | 0 | 0 | 0 | -0.33 |
| *NA* | lmo1301 | conserved hypothetical protein | Other | 2.83 | 0 | 0 | 0 | 0.47 |
| *NA* | lmo0134 | similar to E. coli YjdJ protein | Other | 2.2 | UP | 0 | 6.7 | -0.72 |
| *NA* | lmo0727 | similar to L-glutamine-D-fructose-6-phosphate amidotransferase | Amino sugars | 2.04 | 0 | 0 | 0 | 0.87 |
| *NA* | LMOh7858_2560 | carbon-sulfur lyase | Sulfur metabolism | 2 | 0 | 0 | 0 | 0 |
| *NA* | lmo0956 | similar to N-acetylglucosamine-6P-phosphate deacetylase (EC 3.5.1.25) | Amino sugars | 1.96 | UP | 0 | 2.7 | -1.48 |
| *NA* | lmo2555 | weakly similar to human N-acetylglucosaminyl-phosphatidylinositol biosynthetic protein | Other | 1.89 | 0 | 0 | 0 | 0.16 |
| *NA* | lmo2263 | similar to unkown proteins | Other | 1.86 | 0 | 0 | 0 | -0.11 |
| *NA* | lmo1383 | similar to unknown protein | Other | 1.73 | 0 | 0 | 0 | -0.15 |
| *NA* | lmo0957 | similar to glucosamine-6-Phoasphate isomerase (EC 5.3.1.10) | Amino sugars | 1.68 | UP | 0 | 2.2 | -0.06 |
| *NA* | lmo0011 | similar to mevalonate diphosphate decarboxylase | Other | 1.67 | 0 | 0 | 0 | -1.6 |
| *NA* | lmo1087 | similar to glucitol dehydrogenase | Other | 1.64 | DOWN | 0 | 0 | -0.97 |
| *nagA-1* | LMOf6854_1003 | N-acetylglucosamine-6-phosphate deacetylase | Amino sugars | 1.59 | 0 | 0 | 0 | 0 |
| *NA* | lmo2413 | similar to aminotransferase | Other | 1.57 | 0 | 0 | 0 | -1.4 |
| *NA* | lmo0010 | similar to mevalonate kinase | Other | 1.41 | 0 | 0 | 0 | -0.89 |
| *pta* | LMOf2365_2135 | phosphate acetyltransferase | Other | 1.39 | 0 | 0 | 0 | 0 |
| *NA* | lmo0286 | similar to aminotransferase | Other | -3.34 | 0 | 0 | 0 | -0.07 |
| *NA* | lmo1494 | similar to 5-methylthioadenosine/S-adenosylhomocysteine nucleosidase | Other | -2.74 | 0 | 0 | 0 | 0.52 |
| *phnA* | LMOf2365_0386 | alkylphosphonate utilization operon protein PhnA | Phosphorus compounds | -2.73 | 0 | 0 | 0 | 0 |
| *phnA* | LMOf6854_0406 | alkylphosphonate utilization operon protein PhnA | Phosphorus compounds | -2.71 | 0 | 0 | 0 | 0 |
| *NA* | lmo1000 | similar to phytoene dehydrogenase | Other | -2.53 | 0 | 0 | 0 | -0.7 |
| *NA* | lmo2638 | similar to NADH dehydrogenase | Other | -2.53 | 0 | 0 | 0 | -0.02 |
| *NA* | lmo2550 | similar to glycosyl transferases | Other | -2.44 | 0 | 0 | 0 | -0.35 |
| *NA* | lmo0272 | conserved hypothetical protein similar to B. subtilis YxeH protein | Other | -2.44 | 0 | 0 | 0 | 2.27 |
| *NA* | lmo0485 | unknown | Other | -2.38 | 0 | 0 | 0 | 4.86 |
| *NA* | lmo0616 | C-terminal domain similar to glycerophosphoryl diester phosphodiesterase | Other | -2.15 | 0 | 0 | 0 | 0.05 |
| *ackA* | lmo1581 | highly similar to acetate kinase | Other | -2.12 | 0 | 0 | 0 | 0.69 |
| *NA* | lmo0277 | similar to oxidoreductase | Other | -1.98 | DOWN | 0 | 0 | -1.63 |
| *NA* | lmo1351 | unknown | Sulfur metabolism | -1.95 | DOWN | 0 | -2 | -0.24 |
| *NA* | lmo0271 | highly similar to phospho-beta-glucosidase | Other | -1.79 | 0 | 0 | 0 | -1.95 |
| *NA* | lmo0497 | similar to sugar transferase | Other | -1.78 | 0 | 0 | 0 | 1.05 |
| *NA* | lmo0635 | unknown | Other | -1.77 | 0 | 0 | 0 | -0.97 |
| *NA* | lmo1737 | similar to glycerol dehydrogenase | Other | -1.74 | 0 | 0 | 0 | 0.08 |
| *NA* | lmo0773 | similar to alcohol dehydrogenase | Other | -1.71 | 0 | 0 | 0 | 0.4 |
| *NA* | lmo1384 | similar to unknown protein | Sulfur metabolism | -1.61 | DOWN | 0 | 0 | 0.22 |
| *NA* | lmo1734 | similar to glutamate synthase (large subunit) | Nitrogen metabolism | -1.52 | 0 | 0 | 0 | 0.22 |
| *NA* | lmo0652 | similar to unknown proteins | Other | -1.47 | 0 | 0 | 0 | 3.08 |
| *NA* | lmo2577 | conserved hypothetical protein | Other | -1.42 | DOWN | 0 | 0 | -0.24 |
| *NA* | lmo1733 | similar to glutamate synthase (small subunit) | Nitrogen metabolism | -1.42 | 0 | 0 | 0 | 2.19 |
| *NA* | lmo2017 | similar to unknown proteins | Phosphorus compounds | -1.34 | DOWN | 0 | 0 | -0.04 |
| *NA* | lmo2017 | similar to unknown proteins | Other | -1.34 | DOWN | 0 | 0 | -0.04 |
| *NA* | lmo2389 | similar to NADH dehydrogenase | Other | -1.31 | 0 | 0 | 0 | -0.85 |
| *ppaC* | LMOf2365_1467 | inorganic pyrophosphatase, manganese-dependent | Phosphorus compounds | -1.3 | 0 | 0 | 0 | 0 |
| *NA* | lmo0554 | similar to NADH-dependent butanol dehydrogenase | Other | 0 | UP | 0 | 4 | 2.67 |
| *NA* | lmo0752 | weakly similar to a putative haloacetate dehalogenase | Other | 0 | UP | 0 | 0 | 0.95 |
| *NA* | lmo0877 | similar to B. subtilis NagB protein (glucosamine-6-phosphate isomerase) | Amino sugars | 0 | UP | 0 | 0 | -0.33 |
| *NA* | lmo1126 | similar to E. coli YjaB protein | Other | 0 | UP | 0 | 0 | -0.54 |
| *AckA2* | lmo1168 | similar to acetate kinase | Other | 0 | UP | 0 | 0 | 0.74 |
| *NA* | lmo1292 | similar to glycerophosphodiester phosphodiesterase | Other | 0 | UP | 0 | 0 | 0.05 |
| *NA* | lmo1513 | similar to iron-sulfur cofactor synthesis protein | Other | 0 | UP | 0 | 0 | -0.35 |
| *NA* | lmo2027 | putative cell surface protein, similar to internalin proteins | Other | 0 | UP | 0 | 0 | 0.35 |
| *NA* | lmo2108 | similar to N-acetylglucosamine-6-phosphate deacetylase | Amino sugars | 0 | UP | 0 | 0 | -1.5 |
| *NA* | lmo2434 | highly similar to glutamate decarboxylases | Other | 0 | UP | 0 | 4.1 | -0.66 |
| *NA* | lmo2453 | similar to lipolytic enzyme | Other | 0 | UP | -2.09 | 0 | -0.39 |
| *NA* | lmo2573 | similar to zinc-binding dehydrogenase | Other | 0 | UP | 0 | 4.9 | 0.19 |
| *NA* | lmo2592 | similar to oxidoreductase, aldo/keto reductase family | Other | 0 | UP | 0 | 0 | 0.47 |
| *NA* | lmo2663 | similar to polyol dehydrogenase | Other | 0 | UP | 0 | 0 | 0.66 |
| *NA* | lmo2664 | similar to sorbitol dehydrogenase | Other | 0 | UP | 0 | 0 | -2.58 |
| *NA* | lmo2800 | similar to dehydrogenase | Other | 0 | UP | 0 | 0 | 0.3 |
| *NA* | lmo0050 | similar to sensor histidine kinase (AgrC from Staphylococcus) | Nitrogen metabolism | 0 | DOWN | 0 | 0 | -0.73 |
| *NA* | lmo1005 | similar to 3-hydroxyisobutyrate dehydrogenase (B. subtilis YkwC protein) | Other | 0 | DOWN | 0 | 0 | -1.26 |
| *NA* | lmo1006 | similar to aminotransferases (to B. subtilis PatA protein) | Other | 0 | DOWN | 0 | 0 | -0.47 |
| *NA* | lmo1477 | similar to oxidoreductase | Other | 0 | DOWN | 0 | 0 | -0.61 |
| *NA* | lmo1498 | similar to O-methyltransferase | Other | 0 | DOWN | 0 | 0 | -1.2 |
| *metK* | lmo1664 | similar to S-methionine adenosyltransferase | Other | 0 | DOWN | 0 | 0 | 1.92 |
| *pta* | lmo2103 | similar to phosphotransacetylase | Other | 0 | DOWN | 0 | 0 | -1.89 |
| *NA* | lmo2700 | similar to aldo/keto reductase | Other | 0 | DOWN | 0 | 0 | -0.24 |
| *NA* | lmo0640 | similar to oxidoreductase | Other | 0 | 0 | 2.24 | 0 | 0.37 |
| *NA* | lmo1448 | conserved hypothetical protein | Phosphorus compounds | 0 | 0 | 2 | 0 | -0.78 |
| *NA* | lmo2363 | similar to glutamate decarboxylase | Polyamine biosynthesis | 0 | 0 | 2.63 | -2.2 | 1.39 |
| *NA* | lmo2363 | similar to glutamate decarboxylase | Other | 0 | 0 | 2.63 | -2.2 | 1.39 |
| *NA* | lmo2480 | similar to acetyltransferase | Other | 0 | 0 | 2.18 | 0 | 0.25 |
| *menE* | lmo1672 | similar to O-succinylbenzoic acid-CoA ligase | Other | 0 | 0 | 0 | 2.1 | 0.17 |
| *NA* | lmo1684 | similar to glycerate dehydrogenases | Other | 0 | 0 | 0 | 2 | 2.17 |
| *NA* | lmo2159 | similar to oxidoreductase | Other | 0 | 0 | 0 | -1.7 | -0.88 |
| *NA* | lmo2163 | similar to oxidoreductase | Other | 0 | 0 | 0 | -1.6 | -2.14 |
| *NA* | lmo0353 | similar to unknown proteins | Other | 0 | 0 | 0 | 2 | 0.39 |
| *NA* | lmo0395 | weakly similar to blasticidin S-acetyltransferase | Other | 0 | 0 | 0 | -2.3 | 0.66 |
| *NA* | lmo1961 | similar to oxidoreductases | Other | 0 | 0 | 0 | 1 | 0.68 |
| *NA* | lmo0370 | conserved hypothetical protein | Phosphorus compounds | 0 | 0 | 0 | -2.1 | -1.27 |
| *NA* | lmo0012 | similar to mevalonate kinases | Other | 0 | 0 | 0 | 0 | -2.75 |
| *NA* | lmo0018 | beta-glucosidase | Other | 0 | 0 | 0 | 0 | -1.86 |
| *NA* | lmo0030 | conserved hypothetical protein | Other | 0 | 0 | 0 | 0 | -1.66 |
| *NA* | lmo0067 | similar to dinitrogenase reductase ADP-ribosylation system | Other | 0 | 0 | 0 | 0 | -2.06 |
| *NA* | lmo0078 | similar to phosphoglycerate dehydrogenase | Other | 0 | 0 | 0 | 0 | 1.64 |
| *NA* | lmo0084 | similar to oxidoreductases | Other | 0 | 0 | 0 | 0 | -1.66 |
| *NA* | lmo0103 | similar to NADH oxidase | Other | 0 | 0 | 0 | 0 | -0.76 |
| *NA* | lmo0158 | conserved hypothetical protein | Other | 0 | 0 | 0 | 0 | -0.35 |
| *NA* | lmo0276 | conserved hypothetical protein | Other | 0 | 0 | 0 | 0 | -0.83 |
| *NA* | lmo0295 | similar to FMN-containing NADPH-linked nitro/flavin reductase | Other | 0 | 0 | 0 | 0 | -2.25 |
| *NA* | lmo0300 | similar to phospho-beta-glucosidase and phospho-beta-galactosidase | Other | 0 | 0 | 0 | 0 | -0.3 |
| *NA* | lmo0327 | similar to cell surface proteins (LPXTG motif) | Other | 0 | 0 | 0 | 0 | -1.43 |
| *NA* | lmo0339 | weakly similar to inorganic pyrophosphatase | Phosphorus compounds | 0 | 0 | 0 | 0 | 2.27 |
| *NA* | lmo0339 | weakly similar to inorganic pyrophosphatase | Other | 0 | 0 | 0 | 0 | 2.27 |
| *NA* | lmo0356 | similar to oxidoreductase | Other | 0 | 0 | 0 | 0 | 0.7 |
| *NA* | lmo0372 | similar to beta-glucosidase | Other | 0 | 0 | 0 | 0 | -0.77 |
| *NA* | lmo0420 | conserved hypothetical protein | Other | 0 | 0 | 0 | 0 | 0.99 |
| *NA* | lmo0432 | similar to oxidoreductase | Other | 0 | 0 | 0 | 0 | 0.76 |
| *NA* | lmo0447 | similar to glutamate decarboxylase | Other | 0 | 0 | 0 | 0 | -1.26 |
| *NA* | lmo0458 | similar to hydantoinase | Phosphorus compounds | 0 | 0 | 0 | 0 | 0.47 |
| *NA* | lmo0493 | similar to acylase | Other | 0 | 0 | 0 | 0 | 0.79 |
| *NA* | lmo0506 | similar to polyol (sorbitol) dehydrogenase | Other | 0 | 0 | 0 | 0 | 2.53 |
| *NA* | lmo0529 | conserved hypothetical protein similar to putative glucosaminyltransferase | Other | 0 | 0 | 0 | 0 | 0.28 |
| *NA* | lmo0536 | similar to 6-phospho-beta-glucosidase | Other | 0 | 0 | 0 | 0 | -2.72 |
| *NA* | lmo0549 | similar to internalin protein | Other | 0 | 0 | 0 | 0 | 2.7 |
| *NA* | lmo0574 | similar to beta-glucosidase | Other | 0 | 0 | 0 | 0 | 0.58 |
| *NA* | lmo0609 | similar to E. coli phage shock protein E | Sulfur metabolism | 0 | 0 | 0 | 0 |  |
| *NA* | lmo0613 | similar to oxidoreductase | Other | 0 | 0 | 0 | 0 | -0.5 |
| *NA* | lmo0614 | conserved hypothetical protein | Other | 0 | 0 | 0 | 0 | -0.48 |
| *NA* | lmo0663 | conserved hypothetical proteins | Other | 0 | 0 | 0 | 0 | -0.28 |
| *NA* | lmo0792 | similar to conserved hypothetical protein | Other | 0 | 0 | 0 | 0 | 1.59 |
| *NA* | lmo0811 | similar to carbonic anhydrase | Other | 0 | 0 | 0 | 0 | 0.07 |
| *NA* | lmo0823 | similar to oxydoreductases | Other | 0 | 0 | 0 | 0 | 0.11 |
| *NA* | lmo0845 | similar to B. subtilis YxjH and YxjG proteins | Other | 0 | 0 | 0 | 0 | -0.78 |
| *NA* | lmo0849 | similar to amidases | Other | 0 | 0 | 0 | 0 | -1.55 |
| *NA* | lmo0878 | similar to oxidoreductases | Other | 0 | 0 | 0 | 0 | -0.82 |
| *NA* | lmo0933 | similar to sugar transferase | Other | 0 | 0 | 0 | 0 | -1.64 |
| *NA* | lmo0938 | similar to protein-tyrosine-phosphatase | Other | 0 | 0 | 0 | 0 | 1.33 |
| *NA* | lmo1029 | similar to conserved hypothetical proteins | Other | 0 | 0 | 0 | 0 | -0.97 |
| *NA* | lmo1091 | similar to glysosyltransferases | Other | 0 | 0 | 0 | 0 | -0.43 |
| *NA* | lmo1144 | similar to Salmonelle enterica PduU protein | Other | 0 | 0 | 0 | 0 | -0.69 |
| *NA* | lmo1145 | similar to Salmonella enterica PduV protein | Other | 0 | 0 | 0 | 0 | 0.47 |
| *NA* | lmo1155 | similar to diol dehydrase (diol dehydratase) gamma subunit (pddC) | Other | 0 | 0 | 0 | 0 | -0.94 |
| *NA* | lmo1258 | unknown | Other | 0 | 0 | 0 | 0 | 1.37 |
| *NA* | lmo1289 | similar to internalin proteins, putative peptidoglycan bound protein (LPXTG motif) | Other | 0 | 0 | 0 | 0 | 0.56 |
| *NA* | lmo1308 | weakly similar to arginine N-methyltransferases | Other | 0 | 0 | 0 | 0 | 0.16 |
| *NA* | lmo1349 | similar to glycine dehydrogenase (decarboxylating) subunit 1 | Other | 0 | 0 | 0 | 0 | 0.05 |
| *NA* | lmo1350 | similar to glycine dehydrogenase (decarboxylating) subunit 2 | Other | 0 | 0 | 0 | 0 | 0.54 |
| *NA* | lmo1593 | similar to iron-sulfur cofactor synthesis protein nifS | Other | 0 | 0 | 0 | 0 | -0.37 |
| *NA* | lmo1623 | similar to unknown proteins | Phosphorus compounds | 0 | 0 | 0 | 0 | -0.82 |
| *NA* | lmo1623 | similar to unknown proteins | Other | 0 | 0 | 0 | 0 | -0.82 |
| *NA* | lmo1674 | similar to prolyl aminopetidases | Other | 0 | 0 | 0 | 0 | 0.64 |
| *NA* | lmo1702 | similar to glutathione transferase - fosfomycin resistance protein | Other | 0 | 0 | 0 | 0 | 1.08 |
| *NA* | lmo1726 | similar to hypothetical proteins | Other | 0 | 0 | 0 | 0 | 0.43 |
| *NA* | lmo1729 | similar to beta-glucosidases | Other | 0 | 0 | 0 | 0 | -0.93 |
| *NA* | lmo1736 | similar to unknown proteins | Other | 0 | 0 | 0 | 0 | 0.48 |
| *NA* | lmo1789 | weakly similar to Nad(P)h Oxidoreductase chain B | Other | 0 | 0 | 0 | 0 | 0.11 |
| *NA* | lmo1858 | similar to dehydogenases and hypothetical proteins | Other | 0 | 0 | 0 | 0 | -0.12 |
| *NA* | lmo1872 | similar to methyltransferases | Other | 0 | 0 | 0 | 0 | -1.52 |
| *NA* | lmo1910 | similar to oxidoreductases | Other | 0 | 0 | 0 | 0 | -0.78 |
| *NA* | lmo1976 | similar to oxidoreductase | Other | 0 | 0 | 0 | 0 | 0.2 |
| *NA* | lmo2026 | putative peptidoglycan bound protein (LPXTG motif) | Other | 0 | 0 | 0 | 0 | -0.65 |
| *NA* | lmo2111 | similar to FMN-containing NADPH-linked nitro/flavin reductase | Other | 0 | 0 | 0 | 0 | -0.38 |
| *NA* | lmo2117 | similar to unknown proteins | Other | 0 | 0 | 0 | 0 | -2.14 |
| *NA* | lmo2121 | similar to maltosephosphorylase | Other | 0 | 0 | 0 | 0 | -1.48 |
| *NA* | lmo2208 | similar to unknown protein | Other | 0 | 0 | 0 | 0 | 0.08 |
| *NA* | lmo2247 | similar to oxidoreductase | Other | 0 | 0 | 0 | 0 | 0.04 |
| *NA* | lmo2253 | similar to phosphoglucomutase | Other | 0 | 0 | 0 | 0 | -1.28 |
| *NA* | lmo2316 | similar to site-specific DNA-methyltransferase | Other | 0 | 0 | 0 | 0 | -0.01 |
| *NA* | lmo2350 | similar to B. subtilis YtmI protein | Other | 0 | 0 | 0 | 0 | -0.67 |
| *NA* | lmo2358 | similar to N-acetylglucosamine-6-phosphate isomerase | Amino sugars | 0 | 0 | 0 | 0 | 0.06 |
| *NA* | lmo2359 | conserved hypothetical protein | Other | 0 | 0 | 0 | 0 | -0.16 |
| *NA* | lmo2370 | similar to aminotransferase | Other | 0 | 0 | 0 | 0 | 0.48 |
| *NA* | lmo2390 | similar to hypothetical thioredoxine reductase | Other | 0 | 0 | 0 | 0 | -1.5 |
| *NA* | lmo2396 | similar to internalin proteins, putative peptidoglycan bound protein (LPXTG motif) | Other | 0 | 0 | 0 | 0 | -0.83 |
| *NA* | lmo2400 | similar to acetyltransferase | Other | 0 | 0 | 0 | 0 | -3.42 |
| *NA* | lmo2444 | similar to glycosidase | Other | 0 | 0 | 0 | 0 | 0.29 |
| *NA* | lmo2445 | similar to internalin | Other | 0 | 0 | 0 | 0 | 0.94 |
| *NA* | lmo2446 | similar to glycosidase | Other | 0 | 0 | 0 | 0 | 1.32 |
| *NA* | lmo2481 | similar to B. subtilis P-Ser-HPr phosphatase | Other | 0 | 0 | 0 | 0 | 0.84 |
| *NA* | lmo2517 | unknown | Other | 0 | 0 | 0 | 0 | 0.13 |
| *NA* | lmo2540 | similar to phosphatases | Other | 0 | 0 | 0 | 0 | -0.36 |
| *NA* | lmo2554 | similar to galactosyltransferase | Other | 0 | 0 | 0 | 0 | 0.13 |
| *NA* | lmo2658 | similar to spermidine/spermine N1-acetyl transferase | Other | 0 | 0 | 0 | 0 | 1.23 |
| *NA* | lmo2694 | similar to lysine decarboxylase | Polyamine biosynthesis | 0 | 0 | 0 | 0 | -1.97 |
| *NA* | lmo2694 | similar to lysine decarboxylase | Other | 0 | 0 | 0 | 0 | -1.97 |
| *NA* | lmo2699 | similar to conserved hypothetical protein | Other | 0 | 0 | 0 | 0 | 0.34 |
| *NA* | lmo2721 | similar to glucosamine-6-phosphate isomerase | Amino sugars | 0 | 0 | 0 | 0 | -0.84 |
| *NA* | lmo2761 | similar to beta-glucosidase | Other | 0 | 0 | 0 | 0 | 0.08 |
| *bvrC* | lmo2786 | bvrC | Nitrogen metabolism | 0 | 0 | 0 | 0 | 0.59 |
| *NA* | lmo2798 | similar to phosphatase | Other | 0 | 0 | 0 | 0 | 0.46 |
| *NA* | lmo2821 | similar to internalin, Unknown, putative peptidoglycan bound protein (LPXTG motif) | Other | 0 | 0 | 0 | 0 | 1.31 |
| *NA* | lmo2831 | similar to phosphoglucomutase | Other | 0 | 0 | 0 | 0 | -0.19 |
| *NA* | lmo2833 | similar to a maltose phosphorylase | Other | 0 | 0 | 0 | 0 | -0.89 |
| *NA* | lmo2834 | similar to oxidoreductases | Other | 0 | 0 | 0 | 0 | -3.04 |
| *NA* | lmo2836 | similar to alcohol dehydrogenase | Other | 0 | 0 | 0 | 0 | 0.32 |
| *NA* | lmo2844 | similar to unknown proteins | Other | 0 | 0 | 0 | 0 | 5.14 |
| **Energy metabolism** | | |  |  |  |  |  |  |
| *NA* | lmo2494 | similar to negative regulator of phosphate regulon | Glycolysis/gluconeogenesis | 5.35 | UP | 3.43 | 2.4 | 0.13 |
| *NA* | lmo2494 | similar to negative regulator of phosphate regulon | Photosynthesis | 5.35 | UP | 3.43 | 2.4 | 0.13 |
| *NA* | lmo2695 | similar to dihydroxyacetone kinase | Sugars | 4.55 | 0 | 0 | 7 | -1.13 |
| *NA* | lmo2683 | similar to cellobiose phosphotransferase enzyme IIB component | Pyruvate dehydrogenase | 2.72 | 0 | 0 | 0 | -1.77 |
| *NA* | lmo0539 | similar to tagatose-1,6-diphosphate aldolase | Biosynthesis and degradation of polysaccharides | 2.57 | UP | 0 | 18.8 | 0.13 |
| *NA* | lmo2205 | similar to phosphoglyceromutase 1 | Glycolysis/gluconeogenesis | 2.51 | UP | 0 | 4.3 | -0.63 |
| *NA* | lmo1538 | similar to glycerol kinase | Other | 2.45 | UP | 0 | 1.4 | 0.13 |
| *pdhC* | lmo1054 | highly similar to pyruvate dehydrogenase (dihydrolipoamide acetyltransferase E2 subunit) | Pyruvate dehydrogenase | 2.38 | 0 | 0 | 0 | 2.22 |
| *clpC* | lmo0232 | endopeptidase Clp ATP-binding chain C | ATP-proton motive force interconversion | 2.34 | 0 | 0 | 2.5 | 0.62 |
| *rpiB-4* | LMOf6854_2790 | ribose 5-phosphate isomerase B | Pentose phosphate pathway | 2.3 | 0 | 0 | 0 | 0 |
| *NA* | LMOf2365_0568 | putative tagatose 1,6-diphosphate aldolase | Biosynthesis and degradation of polysaccharides | 2.27 | 0 | 0 | 0 | 0 |
| *qoxD* | lmo0016 | highly similar to quinol oxidase aa3-600 chain IV | Electron transport | 2.26 | 0 | 2.24 | 0 | -2.94 |
| *rpiB-4* | LMOf2365_2654 | ribose 5-phosphate isomerase B | Pentose phosphate pathway | 2.22 | 0 | 0 | 0 | 0 |
| *PdhB* | lmo1053 | highly similar to pyruvate dehydrogenase (E1 beta subunit) | Pyruvate dehydrogenase | 2.1 | 0 | 0 | 3.2 | 1.86 |
| *NA* | lmo1514 | similar to unknown protein | ATP-proton motive force interconversion | 2.01 | 0 | 0 | 0 | 0.08 |
| *NA* | lmo1720 | similar to phosphotransferase system (PTS) lichenan-specific enzyme IIB component | Pyruvate dehydrogenase | 1.91 | 0 | 0 | 0 | 1.11 |
| *citZ* | lmo1567 | highly similar to citrate synthase subunit II | TCA cycle | 1.81 | UP | 0 | 0 | 0.13 |
| *NA* | LMOf2365_1075 | dihydrolipoamide acetyltransferase | Pyruvate dehydrogenase | 1.81 | 0 | 0 | 0 | 0 |
| *qoxC* | lmo0015 | AA3-600 quinol oxidase subunit III | Electron transport | 1.8 | 0 | 2.36 | 0 | -2.78 |
| *NA* | lmo2373 | similar to phosphotransferase system (PTS) beta-glucoside-specific enzyme IIB component | Pyruvate dehydrogenase | 1.79 | 0 | 2.1 | 0 | -0.83 |
| *NA* | lmo1383 | similar to unknown protein | Electron transport | 1.73 | 0 | 0 | 0 | -0.15 |
| *NA* | lmo1383 | similar to unknown protein | Fermentation | 1.73 | 0 | 0 | 0 | -0.15 |
| *NA* | lmo1383 | similar to unknown protein | Glycolysis/gluconeogenesis | 1.73 | 0 | 0 | 0 | -0.15 |
| *NA* | lmo1383 | similar to unknown protein | TCA cycle | 1.73 | 0 | 0 | 0 | -0.15 |
| *NA* | lmo1992 | similar to alpha-acetolactate decarboxylase | Fermentation | 1.67 | UP | 0 | 0 | -0.15 |
| *qoxB* | LMOf6854_0017 | quinol oxidase AA3, subunit I | Electron transport | 1.64 | 0 | 0 | 0 | 0 |
| *NA* | lmo1087 | similar to glucitol dehydrogenase | Fermentation | 1.64 | DOWN | 0 | 0 | -0.97 |
| *tkt* | lmo1305 | highly similar to transketolase | Pentose phosphate pathway | 1.59 | 0 | 0 | 0 | 1.51 |
| *NA* | lmo1373 | similar to branched-chain alpha-keto acid dehydrogenase E1 subunit (2-oxoisovalerate dehydrogenase beta subunit) | Pyruvate dehydrogenase | 1.59 | 0 | 0 | 0 | 0.12 |
| *PdhD* | lmo1055 | highly similar to dihydrolipoamide dehydrogenase, E3 subunit of pyruvate dehydrogenase complex | Pyruvate dehydrogenase | 1.51 | 0 | 0 | 0 | 0.49 |
| *pdhA* | LMOf2365_1073 | pyruvate dehydrogenase complex, E1 component, pyruvate dehydrogenase alpha subunit | Pyruvate dehydrogenase | 1.45 | 0 | 0 | 0 | 0 |
| *trxB* | lmo2478 | thioredoxin reductase | Electron transport | 1.44 | 0 | 0 | 0 | -1.89 |
| *pta* | LMOf2365_2135 | phosphate acetyltransferase | Fermentation | 1.39 | 0 | 0 | 0 | 0 |
| *NA* | lmo1467 | similar to phosphate starvation induced protein PhoH | Glycolysis/gluconeogenesis | 1.34 | 0 | 0 | 0 | 0.33 |
| *NA* | lmo0359 | similar to D-fructose-1,6-biphosphate aldolase | Glycolysis/gluconeogenesis | 1.33 | 0 | 0 | 0 | 0.53 |
| *NA* | lmo0736 | similar to ribose 5-phosphate isomerase | Pentose phosphate pathway | 1.31 | 0 | 0 | 0 | -0.76 |
| *garK-2* | LMOf6854_2949 | glycerate kinase 2 | Other | 1.28 | 0 | 0 | 0 | 0 |
| *NA* | lmo2564 | similar to 4-oxalocrotonate isomerase | Other | 1.22 | 0 | 0 | 0 | 1.63 |
| *NA* | lmo0097 | similar to PTS system mannose-specific, factor IIC | Pyruvate dehydrogenase | -6.17 | DOWN | -2.79 | 0 | -0.62 |
| *dat* | LMOf6854_1672 | D-amino acid aminotransferase | Amino acids and amines | -3.53 | 0 | 0 | 0 | 0 |
| *cydB* | lmo2717 | highly similar to cytochrome D ubiquinol oxidase subunit II | Electron transport | -3.3 | 0 | -2.82 | 0 | 1.15 |
| *cydA* | lmo2718 | highly similar to cytochrome D ubiquinol oxidase subunit I | Electron transport | -3.24 | 0 | -2.74 | -1.3 | 1.13 |
| *NA* | lmo1935 | similar to protein-tyrosine/serine phosphatase | Sugars | -2.88 | 0 | 0 | 0 | 0.32 |
| *NA* | lmo0096 | similar to PTS system mannose-specific, factor IIAB | Pyruvate dehydrogenase | -2.84 | DOWN | -2.74 | 0 | -0.9 |
| *pgi* | lmo2367 | glucose-6-phosphate isomerase | Glycolysis/gluconeogenesis | -2.77 | DOWN | 0 | 0 | 1.53 |
| *NA* | LMOf2365_2692 | putative gluconate kinase | Pentose phosphate pathway | -2.65 | 0 | 0 | 0 | 0 |
| *NA* | lmo0560 | similar to NADP-specific glutamate dehydrogenase | Amino acids and amines | -2.57 | 0 | 0 | -1.5 | -0.32 |
| *gpsA* | lmo1936 | similar to NAD(P)H-dependent glycerol-3-phosphate dehydrogenase | Other | -2.5 | DOWN | 2.2 | 0 | 1.39 |
| *NA* | lmo1255 | similar to PTS system trehalose specific enzyme IIBC | Pyruvate dehydrogenase | -2.48 | 0 | 0 | 0 | 1.46 |
| *NA* | lmo1978 | similar to glucose-6-phosphate 1-dehydrogenase | Pentose phosphate pathway | -2.41 | 0 | 0 | 0 | 1.08 |
| *NA* | lmo0485 | unknown | Electron transport | -2.38 | 0 | 0 | 0 | 4.86 |
| *NA* | lmo0098 | similar to PTS system mannose-specific, factor IID | Pyruvate dehydrogenase | -2.36 | DOWN | -3.3 | 0 | -0.33 |
| *pfk* | lmo1571 | highly similar to 6-phosphofructokinase | Glycolysis/gluconeogenesis | -2.3 | 0 | 0 | 0 | -0.16 |
| *galE* | lmo2477 | UDP-glucose 4-epimerase | Sugars | -2.3 | 0 | 0 | 0 | 0.48 |
| *NA* | lmo0537 | similar to N-carbamyl-L-amino acid amidohydrolase | Amino acids and amines | -2.29 | DOWN | 0 | 0 | -0.86 |
| *atpI* | LMOf2365_2509 | ATP synthase protein I | ATP-proton motive force interconversion | -2.27 | 0 | 0 | 0 | 0 |
| *pfk* | LMOf2365_1593 | 6-phosphofructokinase | Glycolysis/gluconeogenesis | -2.25 | 0 | 0 | 0 | 0 |
| *NA* | LMOf2365_0695 | carboxymuconolactone decarboxylase family protein | Other | -2.23 | 0 | 0 | 0 | 0 |
| *NA* | lmo2712 | highly similar to gluconate kinase | Sugars | -2.2 | 0 | 0 | 0 | 0.94 |
| *NA* | LMOf2365_0434 | polysaccharide deacetylase family protein | Biosynthesis and degradation of polysaccharides | -2.18 | 0 | 0 | 0 | 0 |
| *NA* | lmo0616 | C-terminal domain similar to glycerophosphoryl diester phosphodiesterase | Other | -2.15 | 0 | 0 | 0 | 0.05 |
| *daaA* | lmo1619 | D-Amino Acid Aminotransferase | Amino acids and amines | -2.15 | 0 | 0 | 0 | -0.03 |
| *NA* | lmo0813 | similar to fructokinases | Sugars | -2.13 | UP | 0 | 0 | -0.47 |
| *ackA* | lmo1581 | highly similar to acetate kinase | Fermentation | -2.12 | 0 | 0 | 0 | 0.69 |
| *NA* | lmo0268 | similar to phosphoglycerate mutase | Glycolysis/gluconeogenesis | -2.07 | 0 | 0 | -2 | -1.29 |
| *NA* | LMOf6854_0874 | pyruvate flavodoxin/ferredoxin oxidoreductase | Fermentation | -2.06 | 0 | 0 | 0 | 0 |
| *NA* | lmo0277 | similar to oxidoreductase | Electron transport | -1.98 | DOWN | 0 | 0 | -1.63 |
| *NA* | lmo0277 | similar to oxidoreductase | Fermentation | -1.98 | DOWN | 0 | 0 | -1.63 |
| *tpi* | lmo2457 | highly similar to triose phosphate isomerase | Glycolysis/gluconeogenesis | -1.91 | 0 | 0 | 0 | 0.09 |
| *NA* | lmo1871 | similar to phosphoglucomutases | Sugars | -1.83 | 0 | 0 | 0 | -0.04 |
| *NA* | lmo0678 | similar to flagellar biosynthetic protein FliR | Electron transport | -1.77 | 0 | 1.88 | 0 | 0.8 |
| *atpB* | lmo2535 | highly similar to H+-transporting ATP synthase chain a | ATP-proton motive force interconversion | -1.77 | 0 | 0 | 0 | 1.1 |
| *NA* | LMOf2365_2448 | phosphoglucomutase/phosphomannomutase family protein | Sugars | -1.75 | 0 | 0 | 0 | 0 |
| *pykA* | lmo1570 | highly similar to pyruvate kinases | Glycolysis/gluconeogenesis | -1.74 | 0 | 0 | 0 | -0.38 |
| *NA* | lmo2404 | similar to conserved hypothetical proteins | Photosynthesis | -1.74 | DOWN | 0 | 0 | 0.19 |
| *NA* | LMOh7858_0267 | B. subtilis YazC protein homolog lmo0240 [imported] | Electron transport | -1.74 | 0 | 0 | 0 | 0 |
| *pyc* | LMOf2365_1089 | pyruvate carboxylase | Glycolysis/gluconeogenesis | -1.72 | 0 | 0 | 0 | 0 |
| *NA* | lmo0773 | similar to alcohol dehydrogenase | Fermentation | -1.71 | 0 | 0 | 0 | 0.4 |
| *NA* | LMOf2365_0376 | fumarate reductase, flavoprotein subunit | TCA cycle | -1.71 | 0 | 0 | 0 | 0 |
| *ldh-2* | LMOf6854_1582 | L-lactate dehydrogenase | Aerobic | -1.71 | 0 | 0 | 0 | 0 |
| *ldh-2* | LMOf6854_1582 | L-lactate dehydrogenase | Anaerobic | -1.71 | 0 | 0 | 0 | 0 |
| *ldh-2* | LMOf6854_1582 | L-lactate dehydrogenase | Glycolysis/gluconeogenesis | -1.71 | 0 | 0 | 0 | 0 |
| *gap* | lmo2459 | highly similar to glyceraldehyde 3-phosphate dehydrogenase | Glycolysis/gluconeogenesis | -1.68 | 0 | 0 | 0 | 2.06 |
| *deoC* | LMOf6854_2055 | deoxyribose-phosphate aldolase | Other | -1.68 | 0 | 0 | 0 | 0 |
| *NA* | lmo1534 | similar to L-lactate dehydrogenase | Glycolysis/gluconeogenesis | -1.66 | 0 | 0 | 0 | 0.25 |
| *atpB* | LMOf6854_2597 | ATP synthase F0, A subunit | ATP-proton motive force interconversion | -1.66 | 0 | 0 | 0 | 0 |
| *NA* | lmo2476 | similar to aldose 1-epimerase (mutarotase) | Sugars | -1.64 | 0 | 0 | 0 | 0.9 |
| *pflB* | lmo1406 | pyruvate formate-lyase | Fermentation | -1.63 | 0 | 0 | 0 | 0.16 |
| *NA* | lmo2475 | similar to phosphomannomutase and phosphoglucomutase | Sugars | -1.63 | 0 | 0 | 0 | 0.46 |
| *NA* | lmo1813 | similar to phosphoglycerate dehydrogenase | Amino acids and amines | -1.63 | 0 | 0 | 0 | 0.99 |
| *NA* | LMOf6854_2509 | glycosyl hydrolase, family 31 | Biosynthesis and degradation of polysaccharides | -1.62 | 0 | 0 | 0 | 0 |
| *NA* | lmo0557 | similar to phosphoglycerate mutase | Glycolysis/gluconeogenesis | -1.52 | 0 | 0 | 0 | -1.31 |
| *atpH* | lmo2532 | highly similar to H+-transporting ATP synthase chain delta | ATP-proton motive force interconversion | -1.49 | 0 | 0 | 0 | -0.71 |
| *pyk* | LMOf2365_1592 | pyruvate kinase | Glycolysis/gluconeogenesis | -1.46 | 0 | 0 | 0 | 0 |
| *NA* | LMOf6854_1795 | alcohol dehydrogenase, iron-dependent | Fermentation | -1.46 | 0 | 0 | 0 | 0 |
| *atpF* | lmo2533 | highly similar to H+-transporting ATP synthase chain b | ATP-proton motive force interconversion | -1.43 | 0 | 0 | 0 | -0.61 |
| *NA* | lmo2110 | similar to mannnose-6 phospate isomerase | Sugars | -1.42 | 0 | 0 | 0 | -1.5 |
| *NA* | lmo1812 | similar to L-serine dehydratase | Amino acids and amines | -1.41 | 0 | 0 | 0 | 0.74 |
| *NA* | lmo1812 | similar to L-serine dehydratase | Glycolysis/gluconeogenesis | -1.41 | 0 | 0 | 0 | 0.74 |
| *NA* | LMOf2365_2395 | putative thioredoxin | Electron transport | -1.39 | 0 | 0 | 0 | 0 |
| *pycA* | lmo1072 | highly similar to pyruvate carboxylase | Glycolysis/gluconeogenesis | -1.39 | DOWN | 0 | 0 | 0.29 |
| *NA* | LMOf6854_0556 | maturase-related protein | Electron transport | -1.38 | 0 | 0 | 0 | 0 |
| *atpI* | lmo2536 | highly similar to ATP synthase subunit i | ATP-proton motive force interconversion | -1.37 | DOWN | 0 | 0 | -1.44 |
| *NA* | lmo0825 | similar to 3-hydroxy-3-methylglutaryl-coenzyme a reductase | Other | -1.25 | 0 | 0 | 0 | -0.58 |
| *NA* | lmo0556 | similar to phosphoglycerate mutase | Glycolysis/gluconeogenesis | -1.24 | 0 | 0 | 0 | 1.59 |
| *NA* | lmo1511 | similar to unknown proteins | Other | -1.23 | 0 | 0 | 0 | 0.62 |
| *NA* | lmo0021 | similar to PTS system, fructose-specific IIA component | Pyruvate dehydrogenase | 0 | UP | 0 | 0 | 0.51 |
| *NA* | lmo0039 | similar to carbamate kinase | Amino acids and amines | 0 | UP | 0 | 0 | 3.64 |
| *NA* | lmo0043 | similar to arginine deiminase | Amino acids and amines | 0 | UP | 0 | 4 | -0.95 |
| *NA* | lmo0089 | weakly similar to ATP synthase delta chain | ATP-proton motive force interconversion | 0 | UP | 0 | 0 | -0.16 |
| *NA* | lmo0105 | highly similar to chitinase B | ATP-proton motive force interconversion | 0 | UP | 0 | 0 | -0.76 |
| *ldh* | lmo0210 | similar to L-lactate dehydrogenase | Glycolysis/gluconeogenesis | 0 | UP | -3.98 | 2 | -0.37 |
| *NA* | lmo0406 | similar to B. subtilis YyaH protein | Other | 0 | UP | 0 | 0 | 0.63 |
| *NA* | lmo0411 | similar to phosphoenolpyruvate synthase (N-terminal part) | Glycolysis/gluconeogenesis | 0 | UP | 0 | 0 | 2.25 |
| *NA* | lmo0446 | similar to penicillin acylase and to conjugated bile acid hydrolase | Sugars | 0 | UP | 0 | 0 | -2.09 |
| *NA* | lmo0343 | similar to transaldolase | Pentose phosphate pathway | 0 | UP | 0 | 1 | -0.7 |
| *NA* | lmo0344 | similar to dehydrogenase/reductase | Biosynthesis and degradation of polysaccharides | 0 | UP | 0 | 1 | 1.2 |
| *NA* | lmo0345 | similar to sugar-phosphate isomerase | Pentose phosphate pathway | 0 | UP | 0 | 1 | 0.8 |
| *NA* | lmo0348 | similar to dihydroxyacetone kinase | Sugars | 0 | UP | 0 | 1.1 | 1.02 |
| *NA* | lmo0646 | similar to unknown proteins | Pyruvate dehydrogenase | 0 | UP | 0 | 0 | 1.34 |
| *NA* | lmo0646 | similar to unknown proteins | Other | 0 | UP | 0 | 0 | 1.34 |
| *NA* | lmo0722 | similar to pyruvate oxidase | Sugars | 0 | UP | 0 | 4.5 | 0.81 |
| *NA* | lmo0758 | unknown | Pyruvate dehydrogenase | 0 | UP | 0 | 0 | -0.26 |
| *NA* | lmo0758 | unknown | Other | 0 | UP | 0 | 0 | -0.26 |
| *NA* | lmo0759 | unknown | Pyruvate dehydrogenase | 0 | UP | 0 | 0 | 0.52 |
| *NA* | lmo0759 | unknown | Other | 0 | UP | 0 | 0 | 0.52 |
| *NA* | lmo0781 | similar to mannose-specific phosphotransferase system (PTS) component IID | Pyruvate dehydrogenase | 0 | UP | 0 | 2.9 | -0.54 |
| *NA* | lmo0862 | similar to oligo-1,6-glucosidase | Biosynthesis and degradation of polysaccharides | 0 | UP | 0 | 0 | -0.17 |
| *NA* | lmo0865 | similar to phosphomannomutase | Sugars | 0 | UP | 0 | 0 | 0.93 |
| *NA* | lmo0913 | similar to succinate semialdehyde dehydrogenase | Other | 0 | UP | 2.17 | 9.7 | -0.59 |
| *fri* | lmo0943 | non-heme iron-binding ferritin | Fermentation | 0 | UP | 0 | 1.9 | -1.98 |
| *AckA2* | lmo1168 | similar to acetate kinase | Fermentation | 0 | UP | 0 | 0 | 0.74 |
| *NA* | lmo1292 | similar to glycerophosphodiester phosphodiesterase | Other | 0 | UP | 0 | 0 | 0.05 |
| *glpD* | lmo1293 | similar to glycerol 3 phosphate dehydrogenase | Other | 0 | UP | 0 | 2.2 | -0.1 |
| *comGB* | lmo1346 | similar to B. subtilis comG operon protein 2 | Electron transport | 0 | UP | 0 | 0 | 0.22 |
| *NA* | lmo1376 | similar to 6-phosphogluconate dehydrogenase | Pentose phosphate pathway | 0 | UP | 0 | 0 | -1.09 |
| *NA* | lmo1971 | similar to pentitol PTS system enzyme II C component | Pyruvate dehydrogenase | 0 | UP | 0 | 0 | 0.87 |
| *NA* | lmo1973 | similar to PTS system enzyme II A component | Pyruvate dehydrogenase | 0 | UP | 0 | 0 | 0.8 |
| *NA* | lmo2000 | similar to PTS mannose-specific enzyme IID component | Pyruvate dehydrogenase | 0 | UP | 0 | 0 | 1.38 |
| *NA* | lmo2001 | similar to PTS mannose-specific enzyme IIC component | Pyruvate dehydrogenase | 0 | UP | 0 | 0 | -0.63 |
| *NA* | lmo2002 | similar to PTS mannose-specific enzyme IIB component | Pyruvate dehydrogenase | 0 | UP | 0 | 0 | 0.12 |
| *NA* | lmo2027 | putative cell surface protein, similar to internalin proteins | Amino acids and amines | 0 | UP | 0 | 0 | 0.35 |
| *NA* | lmo2266 | similar to unknown proteins | Amino acids and amines | 0 | UP | 0 | 0 | -1.07 |
| *fruA* | lmo2335 | highly similar to phosphotransferase system (PTS) fructose-specific enzyme IIABC component | Pyruvate dehydrogenase | 0 | UP | 13.03 | 0 | 0.19 |
| *fruB* | lmo2336 | fructose-1-phosphate kinase | Sugars | 0 | UP | 10.63 | -1.1 | -0.97 |
| *NA* | lmo2424 | similar to thioredoxin | Electron transport | 0 | UP | 0 | 0 | 0.59 |
| *NA* | lmo2434 | highly similar to glutamate decarboxylases | Amino acids and amines | 0 | UP | 0 | 4.1 | -0.66 |
| *NA* | lmo2437 | unknown | Pyruvate dehydrogenase | 0 | UP | 0 | 1.3 | 0.01 |
| *NA* | lmo2437 | unknown | Other | 0 | UP | 0 | 1.3 | 0.01 |
| *NA* | lmo2573 | similar to zinc-binding dehydrogenase | Fermentation | 0 | UP | 0 | 4.9 | 0.19 |
| *NA* | lmo2661 | similar to ribulose-5-phosphate 3-epimerase | Pentose phosphate pathway | 0 | UP | 0 | 0 | 2.35 |
| *NA* | lmo2663 | similar to polyol dehydrogenase | Electron transport | 0 | UP | 0 | 0 | 0.66 |
| *NA* | lmo2663 | similar to polyol dehydrogenase | Fermentation | 0 | UP | 0 | 0 | 0.66 |
| *NA* | lmo2663 | similar to polyol dehydrogenase | Glycolysis/gluconeogenesis | 0 | UP | 0 | 0 | 0.66 |
| *NA* | lmo2663 | similar to polyol dehydrogenase | TCA cycle | 0 | UP | 0 | 0 | 0.66 |
| *NA* | lmo2664 | similar to sorbitol dehydrogenase | Electron transport | 0 | UP | 0 | 0 | -2.58 |
| *NA* | lmo2664 | similar to sorbitol dehydrogenase | Fermentation | 0 | UP | 0 | 0 | -2.58 |
| *NA* | lmo2664 | similar to sorbitol dehydrogenase | Glycolysis/gluconeogenesis | 0 | UP | 0 | 0 | -2.58 |
| *NA* | lmo2664 | similar to sorbitol dehydrogenase | TCA cycle | 0 | UP | 0 | 0 | -2.58 |
| *NA* | lmo2674 | similar to ribose 5-phosphate epimerase | Pentose phosphate pathway | 0 | UP | 0 | 1.8 | 1.1 |
| *NA* | lmo2772 | similar to beta-glucoside-specific enzyme IIABC | Pyruvate dehydrogenase | 0 | UP | 0 | 0 | 0.54 |
| *NA* | lmo2797 | similar to phosphotransferase system mannitol-specific enzyme IIA | Pyruvate dehydrogenase | 0 | UP | 0 | 0 | -0.5 |
| *NA* | lmo2800 | similar to dehydrogenase | Electron transport | 0 | UP | 0 | 0 | 0.3 |
| *NA* | lmo2800 | similar to dehydrogenase | Fermentation | 0 | UP | 0 | 0 | 0.3 |
| *NA* | lmo2830 | similar to thioredoxin | Electron transport | 0 | UP | 0 | 0 | -1.21 |
| *NA* | lmo2847 | highly similar to rhamnulose-1-phosphate aldolase | Sugars | 0 | UP | 0 | 0 |  |
| *NA* | lmo2849 | similar to rhamnulokinase | Sugars | 0 | UP | 0 | 0 |  |
| *NA* | lmo1005 | similar to 3-hydroxyisobutyrate dehydrogenase (B. subtilis YkwC protein) | Amino acids and amines | 0 | DOWN | 0 | 0 | -1.26 |
| *NA* | lmo1005 | similar to 3-hydroxyisobutyrate dehydrogenase (B. subtilis YkwC protein) | Other | 0 | DOWN | 0 | 0 | -1.26 |
| *NA* | lmo1339 | similar to glucose kinase | Sugars | 0 | DOWN | 0 | 0 | 0.77 |
| *dra* | lmo1995 | similar to deoxyribose-phosphate aldolase | Other | 0 | DOWN | 0 | 0 | 0.46 |
| *NA* | lmo2097 | similar to PTS system galactitol-specific enzyme IIB component | Pyruvate dehydrogenase | 0 | DOWN | 0 | 0 | 2.62 |
| *pta* | lmo2103 | similar to phosphotransacetylase | Fermentation | 0 | DOWN | 0 | 0 | -1.89 |
| *fbaA* | lmo2556 | similar to fructose-1,6-bisphosphate aldolase | Glycolysis/gluconeogenesis | 0 | DOWN | 0 | 0 | -0.45 |
| *NA* | lmo2650 | similar to hypothetical PTS enzyme IIB component | Pyruvate dehydrogenase | 0 | DOWN | 0 | 0 | -0.26 |
| *qoxA* | lmo0013 | AA3-600 quinol oxidase subunit II | Electron transport | 0 | 0 | 1.4 | 0 | -4.13 |
| *NA* | lmo0640 | similar to oxidoreductase | Electron transport | 0 | 0 | 2.24 | 0 | 0.37 |
| *NA* | lmo0640 | similar to oxidoreductase | Fermentation | 0 | 0 | 2.24 | 0 | 0.37 |
| *NA* | lmo2363 | similar to glutamate decarboxylase | Amino acids and amines | 0 | 0 | 2.63 | -2.2 | 1.39 |
| *NA* | lmo1940 | similar to asparaginase | Amino acids and amines | 0 | 0 | -2.52 | 0 | -1.48 |
| *NA* | lmo2467 | similar to chitinase and chitin binding protein | Biosynthesis and degradation of polysaccharides | 0 | 0 | -2.07 | 0 | 0.44 |
| *NA* | lmo2652 | similar to transcriptional antiterminator | Pyruvate dehydrogenase | 0 | 0 | -2.32 | 0 | 0.48 |
| *NA* | lmo0314 | similar to unknown protein | Sugars | 0 | 0 | 0 | 2.1 | -1.52 |
| *NA* | lmo0342 | similar to transketolase | Pentose phosphate pathway | 0 | 0 | 0 | -1.1 | 0.92 |
| *NA* | lmo0346 | similar to triosephosphate isomerase | Glycolysis/gluconeogenesis | 0 | 0 | 0 | 1 | 2.22 |
| *NA* | lmo0347 | similar to dihydroxyacetone kinase | Sugars | 0 | 0 | 0 | 1 | 1.19 |
| *fbp* | lmo0830 | highly similar to fructose-1,6-bisphosphatase | Glycolysis/gluconeogenesis | 0 | 0 | 0 | 2 | -2.02 |
| *pdhA* | lmo1052 | highly similar to pyruvate dehydrogenase (E1 alpha subunit) | Pyruvate dehydrogenase | 0 | 0 | 0 | 1.8 | 1.61 |
| *NA* | lmo1348 | similar to aminomethyltransferase | Amino acids and amines | 0 | 0 | 0 | -1.3 | 0.16 |
| *NA* | lmo1883 | similar to chitinases | Biosynthesis and degradation of polysaccharides | 0 | 0 | 0 | 2.5 | -1.34 |
| *ctaB* | lmo2057 | highly similar to heme A farnesyltransferase | Electron transport | 0 | 0 | 0 | 2 | 0.14 |
| *NA* | lmo2159 | similar to oxidoreductase | Electron transport | 0 | 0 | 0 | -1.7 | -0.88 |
| *NA* | lmo2159 | similar to oxidoreductase | Fermentation | 0 | 0 | 0 | -1.7 | -0.88 |
| *NA* | lmo2163 | similar to oxidoreductase | Electron transport | 0 | 0 | 0 | -1.6 | -2.14 |
| *NA* | lmo2163 | similar to oxidoreductase | Fermentation | 0 | 0 | 0 | -1.6 | -2.14 |
| *pgm* | lmo2456 | highly similar to phosphoglycerate mutase | Glycolysis/gluconeogenesis | 0 | 0 | 0 | 2.5 | 0.29 |
| *pgk* | lmo2458 | highly similar to phosphoglycerate kinase | Glycolysis/gluconeogenesis | 0 | 0 | 0 | 1.2 | -1.38 |
| *NA* | lmo2696 | similar to hypothetical dihydroxyacetone kinase | Sugars | 0 | 0 | 0 | 6.5 | 0.44 |
| *NA* | lmo2724 | similar to unknown proteins | Pyruvate dehydrogenase | 0 | 0 | 0 | 3.7 | -0.64 |
| *NA* | lmo2724 | similar to unknown proteins | Other | 0 | 0 | 0 | 3.7 | -0.64 |
| *NA* | lmo0788 | unknown | Fermentation | 0 | 0 | 0 | 2 | 0.39 |
| *NA* | lmo0936 | similar to Nitroflavin-reductase | Electron transport | 0 | 0 | 0 | -2.1 | -1.75 |
| *trxA* | lmo1233 | thioredoxin | Electron transport | 0 | 0 | 0 | -2.3 | 0.36 |
| *NA* | lmo0183 | similar to alpha-glucosidase | Sugars | 0 | 0 | 0 | 1 | -1.09 |
| *NA* | lmo0184 | similar to oligo-1,6-glucosidase | Sugars | 0 | 0 | 0 | -1.1 | -0.49 |
| *NA* | lmo0319 | similar to phospho-beta-glucosidase | Sugars | 0 | 0 | 0 | -1.5 | 1.17 |
| *NA* | lmo0027 | similar to PTS system, beta-glucosides specific enzyme IIABC | Pyruvate dehydrogenase | 0 | 0 | 0 | -2 | 0.58 |
| *NA* | lmo0503 | similar to PTS fructose-specific enzyme IIA component | Pyruvate dehydrogenase | 0 | 0 | 0 | 1.4 | -0.58 |
| *NA* | lmo0022 | similar to PTS system, fructose-specific IIB component | Pyruvate dehydrogenase | 0 | 0 | 0 | 0 | 1.08 |
| *NA* | lmo0023 | similar to PTS system, fructose-specific IIC component | Pyruvate dehydrogenase | 0 | 0 | 0 | 0 | 2.84 |
| *NA* | lmo0024 | similar to PTS system, mannose-specific IID component | Pyruvate dehydrogenase | 0 | 0 | 0 | 0 | 1.07 |
| *NA* | lmo0031 | transcriptional regulator LacI family | Sugars | 0 | 0 | 0 | 0 | -1.75 |
| *NA* | lmo0032 | similar to xylose repressor | Sugars | 0 | 0 | 0 | 0 | -2.59 |
| *NA* | lmo0033 | similar to endoglucanase | Biosynthesis and degradation of polysaccharides | 0 | 0 | 0 | 0 | -0.37 |
| *NA* | lmo0088 | similar to ATP synthase C chain | ATP-proton motive force interconversion | 0 | 0 | 0 | 0 | 1.24 |
| *NA* | lmo0090 | similar to ATP synthase alpha chain | ATP-proton motive force interconversion | 0 | 0 | 0 | 0 | -0.51 |
| *NA* | lmo0091 | similar to ATP synthase gamma chain | ATP-proton motive force interconversion | 0 | 0 | 0 | 0 | -0.22 |
| *NA* | lmo0092 | similar to ATP synthase beta chain | ATP-proton motive force interconversion | 0 | 0 | 0 | 0 | 0.92 |
| *NA* | lmo0093 | similar to ATP synthase epsilon chain | ATP-proton motive force interconversion | 0 | 0 | 0 | 0 | 0.72 |
| *NA* | lmo0103 | similar to NADH oxidase | Electron transport | 0 | 0 | 0 | 0 | -0.76 |
| *NA* | lmo0182 | similar to alpha-xylosidase and alpha-glucosidase | Sugars | 0 | 0 | 0 | 0 | -0.07 |
| *NA* | lmo0191 | similar to a putative phospho-beta-glucosidase | Electron transport | 0 | 0 | 0 | 0 | -0.82 |
| *NA* | lmo0261 | similar to phospho-beta-glucosidase | Sugars | 0 | 0 | 0 | 0 | 0.05 |
| *NA* | lmo0295 | similar to FMN-containing NADPH-linked nitro/flavin reductase | Electron transport | 0 | 0 | 0 | 0 | -2.25 |
| *NA* | lmo0299 | similar to PTS beta-glucoside-specific enzyme IIB component | Pyruvate dehydrogenase | 0 | 0 | 0 | 0 | 1.47 |
| *NA* | lmo0301 | similar to PTS beta-glucoside-specific enzyme IIA component | Pyruvate dehydrogenase | 0 | 0 | 0 | 0 | 0.34 |
| *NA* | lmo0305 | similar to low specificity L-allo-threonine aldolase | Amino acids and amines | 0 | 0 | 0 | 0 | 1.91 |
| *NA* | lmo0327 | similar to cell surface proteins (LPXTG motif) | Amino acids and amines | 0 | 0 | 0 | 0 | -1.43 |
| *NA* | lmo0356 | similar to oxidoreductase | Electron transport | 0 | 0 | 0 | 0 | 0.7 |
| *NA* | lmo0356 | similar to oxidoreductase | Fermentation | 0 | 0 | 0 | 0 | 0.7 |
| *NA* | lmo0447 | similar to glutamate decarboxylase | Amino acids and amines | 0 | 0 | 0 | 0 | -1.26 |
| *NA* | lmo0454 | conserved hypothetical protein similar to B. subtilis YeaC | ATP-proton motive force interconversion | 0 | 0 | 0 | 0 | 0.35 |
| *NA* | lmo0489 | similar to NADH:flavin oxidoreductase | Electron transport | 0 | 0 | 0 | 0 | 0.04 |
| *NA* | lmo0498 | similar to ribose 5-phosphate isomerase | Pentose phosphate pathway | 0 | 0 | 0 | 0 | 0.07 |
| *NA* | lmo0499 | similar to ribulose-5-phosphate 3 epimerase | Pentose phosphate pathway | 0 | 0 | 0 | 0 | -0.48 |
| *NA* | lmo0500 | similar to transaldolase | Pentose phosphate pathway | 0 | 0 | 0 | 0 | -0.19 |
| *NA* | lmo0502 | similar to putative sugar-phosphate isomerase | Sugars | 0 | 0 | 0 | 0 | 1.24 |
| *NA* | lmo0505 | similar to ribulose-5-phosphate 3-epimerase | Pentose phosphate pathway | 0 | 0 | 0 | 0 | -2.07 |
| *NA* | lmo0506 | similar to polyol (sorbitol) dehydrogenase | Electron transport | 0 | 0 | 0 | 0 | 2.53 |
| *NA* | lmo0506 | similar to polyol (sorbitol) dehydrogenase | Fermentation | 0 | 0 | 0 | 0 | 2.53 |
| *NA* | lmo0506 | similar to polyol (sorbitol) dehydrogenase | Glycolysis/gluconeogenesis | 0 | 0 | 0 | 0 | 2.53 |
| *NA* | lmo0506 | similar to polyol (sorbitol) dehydrogenase | TCA cycle | 0 | 0 | 0 | 0 | 2.53 |
| *NA* | lmo0508 | similar to PTS system, Galactitol-specific IIC component | Pyruvate dehydrogenase | 0 | 0 | 0 | 0 | -0.03 |
| *NA* | lmo0517 | similar to phosphoglycerate mutase | Glycolysis/gluconeogenesis | 0 | 0 | 0 | 0 | 0.03 |
| *NA* | lmo0521 | similar to 6-phospho-beta-glucosidase | Sugars | 0 | 0 | 0 | 0 | 0.64 |
| *NA* | lmo0542 | similar to PTS system, glucitol/sorbitol-specific enzyme IIA component | Pyruvate dehydrogenase | 0 | 0 | 0 | 0 | 3.44 |
| *NA* | lmo0543 | similar to PTS system, glucitol/sorbitol-specific enzyme IIBC component | Pyruvate dehydrogenase | 0 | 0 | 0 | 0 | 0.48 |
| *NA* | lmo0544 | similar to PTS system, glucitol/sorbitol-specific enzyme II CII component | Pyruvate dehydrogenase | 0 | 0 | 0 | 0 | -0.13 |
| *NA* | lmo0549 | similar to internalin protein | Amino acids and amines | 0 | 0 | 0 | 0 | 2.7 |
| *NA* | lmo0613 | similar to oxidoreductase | Electron transport | 0 | 0 | 0 | 0 | -0.5 |
| *NA* | lmo0613 | similar to oxidoreductase | Fermentation | 0 | 0 | 0 | 0 | -0.5 |
| *NA* | lmo0627 | peptidoglycan bound protein (LPXTG motif) similar to adhesin | Electron transport | 0 | 0 | 0 | 0 | -0.9 |
| *NA* | lmo0643 | similar to putative transaldolase | Pentose phosphate pathway | 0 | 0 | 0 | 0 | -1.01 |
| *NA* | lmo0735 | similar to Ribulose-5-Phosphate 3-Epimerase | Pentose phosphate pathway | 0 | 0 | 0 | 0 | -0.09 |
| *NA* | lmo0738 | similar to phosphotransferase system (PTS) beta-glucoside-specific enzyme IIABC component | Pyruvate dehydrogenase | 0 | 0 | 0 | 0 | 0.9 |
| *NA* | lmo0739 | similar to 6-phospho-beta-glucosidase | Sugars | 0 | 0 | 0 | 0 | 1.34 |
| *NA* | lmo0739 | similar to 6-phospho-beta-glucosidase | Biosynthesis and degradation of polysaccharides | 0 | 0 | 0 | 0 | 1.34 |
| *NA* | lmo0811 | similar to carbonic anhydrase | Photosynthesis | 0 | 0 | 0 | 0 | 0.07 |
| *NA* | lmo0817 | similar to E. coli PhnB protein | Pyruvate dehydrogenase | 0 | 0 | 0 | 0 | -2.06 |
| *NA* | lmo0817 | similar to E. coli PhnB protein | Other | 0 | 0 | 0 | 0 | -2.06 |
| *uhpT* | lmo0837 | highly similar to hexose phosphate transport protein | Glycolysis/gluconeogenesis | 0 | 0 | 0 | 0 | -0.13 |
| *NA* | lmo0875 | similar to PTS system, beta-glucoside enzyme IIB component | Pyruvate dehydrogenase | 0 | 0 | 0 | 0 | -1.86 |
| *NA* | lmo0876 | similar to PTS system, Lichenan-specific enzyme IIC component | Pyruvate dehydrogenase | 0 | 0 | 0 | 0 | 0.1 |
| *NA* | lmo0907 | similar to phosphoglycerate mutase | Glycolysis/gluconeogenesis | 0 | 0 | 0 | 0 | 0 |
| *NA* | lmo0914 | similar to PTS system, IIB component | Pyruvate dehydrogenase | 0 | 0 | 0 | 0 | -1 |
| *NA* | lmo0915 | similar to phosphotransferase system enzyme IIC | Pyruvate dehydrogenase | 0 | 0 | 0 | 0 | -1.17 |
| *NA* | lmo0917 | similar to beta-glucosidase | Sugars | 0 | 0 | 0 | 0 | -2.39 |
| *NA* | lmo0934 | similar to B. subtilis YhbA protein | Electron transport | 0 | 0 | 0 | 0 | -1.04 |
| *NA* | lmo0948 | similar to transcription regulator | Pyruvate dehydrogenase | 0 | 0 | 0 | 0 | -0.69 |
| *NA* | lmo0975 | similar to ribose 5-phosphate isomerase | Pentose phosphate pathway | 0 | 0 | 0 | 0 | -5.82 |
| *NA* | lmo1023 | similar to a bacterial K(+)-uptake system | ATP-proton motive force interconversion | 0 | 0 | 0 | 0 | -1.16 |
| *NA* | lmo1032 | similar to transketolase | Pentose phosphate pathway | 0 | 0 | 0 | 0 | -0.61 |
| *NA* | lmo1033 | similar to transketolase | Pentose phosphate pathway | 0 | 0 | 0 | 0 | -0.44 |
| *NA* | lmo1034 | similar to glycerol kinase | Other | 0 | 0 | 0 | 0 | 0.56 |
| *NA* | lmo1035 | similar to phosphotransferase system (PTS) beta-glucoside-specific enzyme IIABC | Pyruvate dehydrogenase | 0 | 0 | 0 | 0 | -0.55 |
| *NA* | lmo1057 | similar to L-lactate dehydrogenase | Glycolysis/gluconeogenesis | 0 | 0 | 0 | 0 | 0.21 |
| *NA* | lmo1095 | similar to PTS system, cellobiose-specific IIB component (cel A) | Pyruvate dehydrogenase | 0 | 0 | 0 | 0 | -0.94 |
| *NA* | lmo1131 | similar to ABC transporters, ATP-binding proteins | Electron transport | 0 | 0 | 0 | 0 | -0.57 |
| *NA* | lmo1141 | similar to uroporphyrin-III C-methyltransferase | Pyruvate dehydrogenase | 0 | 0 | 0 | 0 | -0.18 |
| *NA* | lmo1142 | similar to Salmonella enterica PduS protein | Electron transport | 0 | 0 | 0 | 0 | 0.26 |
| *NA* | lmo1149 | similar to alpha-ribazole-5-phosphatase | Glycolysis/gluconeogenesis | 0 | 0 | 0 | 0 | -0.99 |
| *NA* | lmo1155 | similar to diol dehydrase (diol dehydratase) gamma subunit (pddC) | Photosynthesis | 0 | 0 | 0 | 0 | -0.94 |
| *NA* | lmo1163 | similar to carbon dioxide concentrating mechanism protein | Photosynthesis | 0 | 0 | 0 | 0 | 0.25 |
| *NA* | lmo1166 | similar to NADPH-dependent butanol dehydrogenase | Fermentation | 0 | 0 | 0 | 0 | 1.01 |
| *pduQ* | lmo1171 | similar to NADPH-dependent butanol dehydrogenase | Fermentation | 0 | 0 | 0 | 0 | 0.85 |
| *NA* | lmo1179 | similar to acetaldehyde dehydrogenase / alcohol dehydrogenase | Fermentation | 0 | 0 | 0 | 0 | 0.61 |
| *NA* | lmo1184 | similar to carbon dioxide concentrating mechanism protein | Photosynthesis | 0 | 0 | 0 | 0 | 0.69 |
| *NA* | lmo1244 | weakly similar to phosphoglycerate mutase 1 | Glycolysis/gluconeogenesis | 0 | 0 | 0 | 0 | -0.07 |
| *NA* | lmo1254 | similar to alpha,alpha-phosphotrehalase | Biosynthesis and degradation of polysaccharides | 0 | 0 | 0 | 0 | 0.55 |
| *NA* | lmo1289 | similar to internalin proteins, putative peptidoglycan bound protein (LPXTG motif) | Amino acids and amines | 0 | 0 | 0 | 0 | 0.56 |
| *NA* | lmo1309 | similar to E. coli YbdM protein | Electron transport | 0 | 0 | 0 | 0 | -0.21 |
| *NA* | lmo1349 | similar to glycine dehydrogenase (decarboxylating) subunit 1 | Amino acids and amines | 0 | 0 | 0 | 0 | 0.05 |
| *NA* | lmo1350 | similar to glycine dehydrogenase (decarboxylating) subunit 2 | Amino acids and amines | 0 | 0 | 0 | 0 | 0.54 |
| *NA* | lmo1371 | similar to branched-chain alpha-keto acid dehydrogenase E3 subunit | Pyruvate dehydrogenase | 0 | 0 | 0 | 0 | 1.21 |
| *NA* | lmo1374 | similar to branched-chain alpha-keto acid dehydrogenase E2 subunit (lipoamide acyltransferase) | Pyruvate dehydrogenase | 0 | 0 | 0 | 0 | 1.01 |
| *NA* | lmo1374 | similar to branched-chain alpha-keto acid dehydrogenase E2 subunit (lipoamide acyltransferase) | TCA cycle | 0 | 0 | 0 | 0 | 1.01 |
| *NA* | lmo1405 | similar to putative anti-terminator regulatory protein | Sugars | 0 | 0 | 0 | 0 | 0.79 |
| *citC* | lmo1566 | highly similar to isocitrate dehyrogenases | TCA cycle | 0 | 0 | 0 | 0 | 0.1 |
| *NA* | lmo1579 | similar to alanine dehydrogenase | Amino acids and amines | 0 | 0 | 0 | 0 | 1.06 |
| *NA* | lmo1609 | similar to thioredoxin | Electron transport | 0 | 0 | 0 | 0 | 0.14 |
| *NA* | lmo1634 | similar to Alcohol-acetaldehyde dehydrogenase | Fermentation | 0 | 0 | 0 | 0 | -0.29 |
| *citB* | lmo1641 | highly similar to aconitate hydratases | TCA cycle | 0 | 0 | 0 | 0 | 0.85 |
| *NA* | lmo1667 | similar to L-lactate dehydrogenases | Glycolysis/gluconeogenesis | 0 | 0 | 0 | 0 | 1.5 |
| *NA* | lmo1678 | similar to 5-methyltetrahydrofolate-homocysteine methyltransferase (metH) | Methanogenesis | 0 | 0 | 0 | 0 | -1.29 |
| *NA* | lmo1679 | similar to cystathionine beta-lyase | Amino acids and amines | 0 | 0 | 0 | 0 | 0.97 |
| *NA* | lmo1719 | similar to phosphotransferase system (PTS) lichenan-specific enzyme IIA component | Pyruvate dehydrogenase | 0 | 0 | 0 | 0 | 1.67 |
| *NA* | lmo1726 | similar to hypothetical proteins | Electron transport | 0 | 0 | 0 | 0 | 0.43 |
| *NA* | lmo1726 | similar to hypothetical proteins | Fermentation | 0 | 0 | 0 | 0 | 0.43 |
| *NA* | lmo1729 | similar to beta-glucosidases | Biosynthesis and degradation of polysaccharides | 0 | 0 | 0 | 0 | -0.93 |
| *NA* | lmo1789 | weakly similar to Nad(P)h Oxidoreductase chain B | Electron transport | 0 | 0 | 0 | 0 | 0.11 |
| *NA* | lmo1789 | weakly similar to Nad(P)h Oxidoreductase chain B | Fermentation | 0 | 0 | 0 | 0 | 0.11 |
| *NA* | lmo1789 | weakly similar to Nad(P)h Oxidoreductase chain B | Glycolysis/gluconeogenesis | 0 | 0 | 0 | 0 | 0.11 |
| *NA* | lmo1789 | weakly similar to Nad(P)h Oxidoreductase chain B | TCA cycle | 0 | 0 | 0 | 0 | 0.11 |
| *NA* | lmo1800 | similar to protein-tyrosine phosphatase | Sugars | 0 | 0 | 0 | 0 | -0.43 |
| *NA* | lmo1818 | similar to ribulose-5-phosphate 3-epimerase | Pentose phosphate pathway | 0 | 0 | 0 | 0 | -0.46 |
| *NA* | lmo1858 | similar to dehydogenases and hypothetical proteins | Electron transport | 0 | 0 | 0 | 0 | -0.12 |
| *NA* | lmo1858 | similar to dehydogenases and hypothetical proteins | Fermentation | 0 | 0 | 0 | 0 | -0.12 |
| *NA* | lmo1867 | similar to pyruvate phosphate dikinase | Glycolysis/gluconeogenesis | 0 | 0 | 0 | 0 | -0.74 |
| *NA* | lmo1867 | similar to pyruvate phosphate dikinase | Photosynthesis | 0 | 0 | 0 | 0 | -0.74 |
| *NA* | lmo1868 | similar to conserved hypothetical proteins | Pyruvate dehydrogenase | 0 | 0 | 0 | 0 | -0.77 |
| *NA* | lmo1868 | similar to conserved hypothetical proteins | Other | 0 | 0 | 0 | 0 | -0.77 |
| *NA* | lmo1906 | similar to methylglyoxal synthase | Other | 0 | 0 | 0 | 0 | -0.7 |
| *NA* | lmo1910 | similar to oxidoreductases | Electron transport | 0 | 0 | 0 | 0 | -0.78 |
| *NA* | lmo1910 | similar to oxidoreductases | Fermentation | 0 | 0 | 0 | 0 | -0.78 |
| *NA* | lmo1915 | similar to malolactic enzyme (malate dehydrogenase) | TCA cycle | 0 | 0 | 0 | 0 | 0.72 |
| *NA* | lmo1915 | similar to malolactic enzyme (malate dehydrogenase) | Other | 0 | 0 | 0 | 0 | 0.72 |
| *pflA* | lmo1917 | similar to pyruvate formate-lyase | Fermentation | 0 | 0 | 0 | 0 | -0.84 |
| *NA* | lmo1944 | similar to ferredoxin | Electron transport | 0 | 0 | 0 | 0 | 1.74 |
| *NA* | lmo1969 | similar to 2-keto-3-deoxygluconate-6-phosphate aldolase | Entner-Doudoroff | 0 | 0 | 0 | 0 | 1.88 |
| *NA* | lmo2026 | putative peptidoglycan bound protein (LPXTG motif) | Amino acids and amines | 0 | 0 | 0 | 0 | -0.65 |
| *NA* | lmo2094 | similar to L-fuculose-phosphate aldolase | Sugars | 0 | 0 | 0 | 0 | 1.7 |
| *NA* | lmo2095 | similar to 1-phosphofructokinase | Glycolysis/gluconeogenesis | 0 | 0 | 0 | 0 | 0.87 |
| *NA* | lmo2096 | similar to PTS system galactitol-specific enzyme IIC component | Pyruvate dehydrogenase | 0 | 0 | 0 | 0 | 0.32 |
| *NA* | lmo2098 | similar to PTS system galactitol-specific enzyme IIA component | Pyruvate dehydrogenase | 0 | 0 | 0 | 0 | -1.94 |
| *NA* | lmo2111 | similar to FMN-containing NADPH-linked nitro/flavin reductase | Electron transport | 0 | 0 | 0 | 0 | -0.38 |
| *NA* | lmo2122 | similar to maltodextrose utilization protein MalA | TCA cycle | 0 | 0 | 0 | 0 | -1.62 |
| *NA* | lmo2123 | similar to maltodextrin ABC-transport system (permease) | Sugars | 0 | 0 | 0 | 0 | -2.07 |
| *NA* | lmo2123 | similar to maltodextrin ABC-transport system (permease) | Biosynthesis and degradation of polysaccharides | 0 | 0 | 0 | 0 | -2.07 |
| *NA* | lmo2124 | similar to maltodextrin ABC-transport system (permease) | Sugars | 0 | 0 | 0 | 0 | -2.11 |
| *NA* | lmo2124 | similar to maltodextrin ABC-transport system (permease) | Biosynthesis and degradation of polysaccharides | 0 | 0 | 0 | 0 | -2.11 |
| *NA* | lmo2126 | similar to maltogenic amylase | Biosynthesis and degradation of polysaccharides | 0 | 0 | 0 | 0 | -2.53 |
| *NA* | lmo2133 | similar to fructose-1,6-biphosphate aldolase type II | Glycolysis/gluconeogenesis | 0 | 0 | 0 | 0 | 0.66 |
| *NA* | lmo2134 | similar to fructose-1,6-biphosphate aldolase type II | Sugars | 0 | 0 | 0 | 0 | -1.13 |
| *NA* | lmo2135 | similar to PTS system, fructose-specific enzyme IIC component | Pyruvate dehydrogenase | 0 | 0 | 0 | 0 | 0.08 |
| *NA* | lmo2138 | similar to transcription regulator | Sugars | 0 | 0 | 0 | 0 | -0.45 |
| *NA* | lmo2143 | weakly similar to mannose-6-phosphate isomerase | Sugars | 0 | 0 | 0 | 0 | -4.88 |
| *NA* | lmo2152 | similar to thioredoxin | Electron transport | 0 | 0 | 0 | 0 | -0.8 |
| *NA* | lmo2153 | similar to flavodoxin | Electron transport | 0 | 0 | 0 | 0 | 1.21 |
| *NA* | lmo2168 | similar to glyoxalase I | Other | 0 | 0 | 0 | 0 | 0.37 |
| *NA* | lmo2172 | similar to propionate CoA-transferase | Fermentation | 0 | 0 | 0 | 0 | -1.41 |
| *citG* | lmo2225 | similar to fumarate hydratase | TCA cycle | 0 | 0 | 0 | 0 | -1.07 |
| *NA* | lmo2288 | Protein gp15 [Bacteriophage A118] | Biosynthesis and degradation of polysaccharides | 0 | 0 | 0 | 0 | -0.87 |
| *NA* | lmo2341 | similar to carbohydrate kinases | Sugars | 0 | 0 | 0 | 0 | -0.84 |
| *NA* | lmo2374 | similar to aspartate kinase | Amino acids and amines | 0 | 0 | 0 | 0 | -1.28 |
| *NA* | lmo2396 | similar to internalin proteins, putative peptidoglycan bound protein (LPXTG motif) | Amino acids and amines | 0 | 0 | 0 | 0 | -0.83 |
| *NA* | lmo2425 | similar to glycine cleavage system protein H | Amino acids and amines | 0 | 0 | 0 | 0 | -0.76 |
| *NA* | lmo2436 | similar to transcription antiterminator | Sugars | 0 | 0 | 0 | 0 | 0.79 |
| *NA* | lmo2445 | similar to internalin | Amino acids and amines | 0 | 0 | 0 | 0 | 0.94 |
| *eno* | lmo2455 | highly similar to enolase | Glycolysis/gluconeogenesis | 0 | 0 | 0 | 0 | 0.34 |
| *NA* | lmo2520 | similar to B. subtilis O-succinylbenzoate-CoA synthase (MenC) | Other | 0 | 0 | 0 | 0 | 1.14 |
| *atpC* | lmo2528 | highly similar to H+-transporting ATP synthase chain epsilon | ATP-proton motive force interconversion | 0 | 0 | 0 | 0 | 2.56 |
| *atpD* | lmo2529 | highly similar to H+-transporting ATP synthase chain beta | ATP-proton motive force interconversion | 0 | 0 | 0 | 0 | 1.01 |
| *atpG* | lmo2530 | highly similar to H+-transporting ATP synthase chain gamma | ATP-proton motive force interconversion | 0 | 0 | 0 | 0 | -0.22 |
| *atpA* | lmo2531 | highly similar to H+-transporting ATP synthase chain alpha | ATP-proton motive force interconversion | 0 | 0 | 0 | 0 | 0.79 |
| *atpE* | lmo2534 | highly similar to H+-transporting ATP synthase chain c | ATP-proton motive force interconversion | 0 | 0 | 0 | 0 | -0.02 |
| *NA* | lmo2584 | similar to formate dehydrogenase associated protein | Anaerobic | 0 | 0 | 0 | 0 | 0.04 |
| *NA* | lmo2584 | similar to formate dehydrogenase associated protein | Electron transport | 0 | 0 | 0 | 0 | 0.04 |
| *NA* | lmo2586 | similar to formate dehydrogenase alpha chain | Fermentation | 0 | 0 | 0 | 0 | -1.71 |
| *NA* | lmo2586 | similar to formate dehydrogenase alpha chain | Glycolysis/gluconeogenesis | 0 | 0 | 0 | 0 | -1.71 |
| *NA* | lmo2586 | similar to formate dehydrogenase alpha chain | Pentose phosphate pathway | 0 | 0 | 0 | 0 | -1.71 |
| *NA* | lmo2586 | similar to formate dehydrogenase alpha chain | TCA cycle | 0 | 0 | 0 | 0 | -1.71 |
| *NA* | lmo2586 | similar to formate dehydrogenase alpha chain | Methanogenesis | 0 | 0 | 0 | 0 | -1.71 |
| *NA* | lmo2659 | similar to ribulose-phosphate 3-epimerase | Pentose phosphate pathway | 0 | 0 | 0 | 0 | 1.65 |
| *NA* | lmo2660 | similar to transketolase | Pentose phosphate pathway | 0 | 0 | 0 | 0 | 0.83 |
| *NA* | lmo2662 | similar to ribose 5-phosphate epimerase | Pentose phosphate pathway | 0 | 0 | 0 | 0 | 1.71 |
| *NA* | lmo2665 | similar to PTS system galactitol-specific enzyme IIC component | Pyruvate dehydrogenase | 0 | 0 | 0 | 0 | 1.29 |
| *NA* | lmo2666 | similar to PTS system galactitol-specific enzyme IIB component | Pyruvate dehydrogenase | 0 | 0 | 0 | 0 | 2.58 |
| *NA* | lmo2667 | similar to PTS system galactitol-specific enzyme IIA component | Pyruvate dehydrogenase | 0 | 0 | 0 | 0 | 1.15 |
| *NA* | lmo2677 | similar to hydrolase (esterase) | Other | 0 | 0 | 0 | 0 | 0.2 |
| *kdpB* | lmo2681 | potassium-transporting atpase b chain | ATP-proton motive force interconversion | 0 | 0 | 0 | 0 | 1.05 |
| *NA* | lmo2685 | similar to cellobiose phosphotransferase enzyme IIA component | Pyruvate dehydrogenase | 0 | 0 | 0 | 0 | -0.29 |
| *NA* | lmo2694 | similar to lysine decarboxylase | Amino acids and amines | 0 | 0 | 0 | 0 | -1.97 |
| *NA* | lmo2720 | similar to acetate-CoA ligase | Fermentation | 0 | 0 | 0 | 0 | 1.3 |
| *NA* | lmo2720 | similar to acetate-CoA ligase | Sugars | 0 | 0 | 0 | 0 | 1.3 |
| *NA* | lmo2730 | similar to phosphatase | Sugars | 0 | 0 | 0 | 0 | 1.15 |
| *NA* | lmo2735 | similar to Sucrose phosphorylase | Biosynthesis and degradation of polysaccharides | 0 | 0 | 0 | 0 | 0.64 |
| *NA* | lmo2743 | similar to transaldolase | Pentose phosphate pathway | 0 | 0 | 0 | 0 | 0.31 |
| *NA* | lmo2762 | similar to PTS cellobiose-specific enzyme IIB | Pyruvate dehydrogenase | 0 | 0 | 0 | 0 | 1.16 |
| *NA* | lmo2771 | similar to beta-glucosidase | Sugars | 0 | 0 | 0 | 0 | -3.42 |
| *NA* | lmo2771 | similar to beta-glucosidase | Biosynthesis and degradation of polysaccharides | 0 | 0 | 0 | 0 | -3.42 |
| *NA* | lmo2780 | similar to cellobiose PTS enzyme IIA | Pyruvate dehydrogenase | 0 | 0 | 0 | 0 | -0.47 |
| *NA* | lmo2782 | similar to PTS, cellobiose-specific IIB component | Pyruvate dehydrogenase | 0 | 0 | 0 | 0 | 0.46 |
| *NA* | lmo2783 | similar to cellobiose phosphotransferase system enzyme IIC | Pyruvate dehydrogenase | 0 | 0 | 0 | 0 | 0 |
| *bvrB* | lmo2787 | beta-glucoside-specific phosphotransferase enzyme II ABC component | Pyruvate dehydrogenase | 0 | 0 | 0 | 0 | 0.64 |
| *NA* | lmo2794 | highly similar to B. subtilis DNA-binding protein Spo0J-like homolog YyaA | Electron transport | 0 | 0 | 0 | 0 | -0.18 |
| *NA* | lmo2821 | similar to internalin, Unknown, putative peptidoglycan bound protein (LPXTG motif) | Amino acids and amines | 0 | 0 | 0 | 0 | 1.31 |
| *NA* | lmo2834 | similar to oxidoreductases | Electron transport | 0 | 0 | 0 | 0 | -3.04 |
| *NA* | lmo2834 | similar to oxidoreductases | Fermentation | 0 | 0 | 0 | 0 | -3.04 |
| *NA* | lmo2836 | similar to alcohol dehydrogenase | Electron transport | 0 | 0 | 0 | 0 | 0.32 |
| *NA* | lmo2836 | similar to alcohol dehydrogenase | Fermentation | 0 | 0 | 0 | 0 | 0.32 |
| *NA* | lmo2836 | similar to alcohol dehydrogenase | Glycolysis/gluconeogenesis | 0 | 0 | 0 | 0 | 0.32 |
| *NA* | lmo2836 | similar to alcohol dehydrogenase | TCA cycle | 0 | 0 | 0 | 0 | 0.32 |
| *NA* | lmo2840 | similar to Sucrose phosphorylase | Biosynthesis and degradation of polysaccharides | 0 | 0 | 0 | 0 | -1.03 |
| *NA* | lmo2841 | weakly similar to sucrose phosphorylase | Biosynthesis and degradation of polysaccharides | 0 | 0 | 0 | 0 | -0.59 |
| **Transport and binding proteins** | | |  |  |  |  |  |  |
| *NA* | LMOf6854_2559 | phosphate ABC transporter, permease protein | Anions | 8.01 | 0 | 0 | 0 | 0 |
| *NA* | lmo2495 | similar to phosphate ABC transporter (ATP-binding protein) | Anions | 6.03 | UP | 0 | 0 | -0.42 |
| *NA* | LMOf2365_2470 | phosphate ABC transporter, permease protein | Anions | 4.19 | 0 | 0 | 0 | 0 |
| *NA* | lmo2087 | similar to unknown proteins | Cations and iron carrying compounds | 4.03 | 0 | 0 | 0 | 0 |
| *clpE* | lmo0997 | ATP-dependent protease | Amino acids, peptides and amines | 3.64 | UP | 0 | 0 | -0.88 |
| *NA* | LMOf2365_2469 | phosphate ABC transporter, ATP-binding protein | Anions | 3.44 | 0 | 0 | 0 | 0 |
| *NA* | LMOh7858_0838 | PTS system, mannose/fructose/sorbose family, IIA component subfamily | Carbohydrates, organic alcohols, and acids | 3.24 | 0 | 0 | 0 | 0 |
| *NA* | lmo2384 | similar to proteins involved in resistance to cholate and to NA(+) and in pH homeostasis | Cations and iron carrying compounds | 2.92 | 0 | 0 | 0 | 0.4 |
| *NA* | LMOf2365_2663 | PTS system, beta-glucoside-specific, IIB component | Carbohydrates, organic alcohols, and acids | 2.9 | 0 | 0 | 0 | 0 |
| *NA* | LMOf6854_2439 | Na+/H+ antiporter component A, putative | Cations and iron carrying compounds | 2.86 | 0 | 0 | 0 | 0 |
| *NA* | lmo2381 | similar to proteins involved in resistance to cholate and to NA(+) and in pH homeostasis | Cations and iron carrying compounds | 2.86 | 0 | 0 | 0 | 0.12 |
| *NA* | LMOf2365_2357 | putative Na+/H+ antiporter component G | Cations and iron carrying compounds | 2.85 | 0 | 0 | 0 | 0 |
| *NA* | LMOf2365_2355 | putative Na+/H+ antiporter component E | Cations and iron carrying compounds | 2.78 | 0 | 0 | 0 | 0 |
| *NA* | lmo2683 | similar to cellobiose phosphotransferase enzyme IIB component | Carbohydrates, organic alcohols, and acids | 2.72 | 0 | 0 | 0 | -1.77 |
| *NA* | LMOh7858_2533 | Na+/H+ antiporter component G, putative | Cations and iron carrying compounds | 2.69 | 0 | 0 | 0 | 0 |
| *NA* | lmo2378 | similar to proteins involved in resistance to cholate and to NA(+) and in pH homeostasis | Cations and iron carrying compounds | 2.61 | 0 | 0 | 0 | -1.37 |
| *NA* | lmo0783 | similar to mannose-specific phosphotransferase system (PTS) component IIB | Carbohydrates, organic alcohols, and acids | 2.55 | UP | 0 | 8.9 | 1.01 |
| *NA* | lmo2382 | similar to proteins involved in resistance to cholate and to NA(+) and in pH homeostasis | Cations and iron carrying compounds | 2.39 | 0 | 0 | 0 | -0.28 |
| *NA* | lmo2383 | similar to proteins involved in resistance to cholate and to NA(+) and in pH homeostasis | Cations and iron carrying compounds | 2.37 | 0 | 0 | 0 | 0.33 |
| *clpC* | lmo0232 | endopeptidase Clp ATP-binding chain C | Cations and iron carrying compounds | 2.34 | 0 | 0 | 2.5 | 0.62 |
| *NA* | lmo0841 | similar to cation (calcium) transporting ATPase | Cations and iron carrying compounds | 2.34 | 0 | 0 | 0 | -0.29 |
| *NA* | lmo1539 | similar to glycerol uptake facilitator | Other | 2.3 | UP | 0 | 1.8 | 0.37 |
| *opuCB* | lmo1427 | similar to glycine betaine/carnitine/choline ABC transporter (membrane protein) | Amino acids, peptides and amines | 2.21 | UP | 3.08 | 3 | -0.41 |
| *NA* | LMOf2365_2471 | phosphate ABC transporter, permease protein | Anions | 2.09 | 0 | 0 | 0 | 0 |
| *NA* | lmo1422 | similar to glycine betaine/carnitine/choline ABC transporter (membrane protein) | Amino acids, peptides and amines | 2.09 | 0 | 0 | 2.9 | 0 |
| *NA* | lmo1514 | similar to unknown protein | Cations and iron carrying compounds | 2.01 | 0 | 0 | 0 | 0.08 |
| *NA* | lmo1720 | similar to phosphotransferase system (PTS) lichenan-specific enzyme IIB component | Carbohydrates, organic alcohols, and acids | 1.91 | 0 | 0 | 0 | 1.11 |
| *NA* | lmo2634 | similar to B. subtilis YbaF protein | Cations and iron carrying compounds | 1.88 | 0 | 0 | 0 | -0.04 |
| *NA* | lmo0993 | similar to Na+-transporting ATP synthase subunit J | Amino acids, peptides and amines | 1.88 | DOWN | 0 | 0 | -0.58 |
| *NA* | lmo0993 | similar to Na+-transporting ATP synthase subunit J | Cations and iron carrying compounds | 1.88 | DOWN | 0 | 0 | -0.58 |
| *NA* | lmo0809 | similar to spermidine/putrescine ABC transporter, permease protein | Amino acids, peptides and amines | 1.87 | 0 | 0 | 0 | 0.02 |
| *NA* | LMOf2365_2394 | cation efflux family protein | Cations and iron carrying compounds | 1.85 | 0 | 0 | 0 | 0 |
| *opuCC* | lmo1426 | similar to glycine betaine/carnitine/choline ABC transporter (osmoprotectant-binding protein) | Amino acids, peptides and amines | 1.85 | UP | 1.41 | 2.7 | 1.24 |
| *NA* | lmo1372 | similar to branched-chain alpha-keto acid dehydrogenase E1 subunit (2-oxoisovalerate dehydrogenase alpha subunit) | Amino acids, peptides and amines | 1.82 | 0 | 0 | 0 | -0.19 |
| *NA* | lmo1382 | unknown | Amino acids, peptides and amines | 1.79 | 0 | 0 | 0 | 2.65 |
| *NA* | lmo1382 | unknown | Carbohydrates, organic alcohols, and acids | 1.79 | 0 | 0 | 0 | 2.65 |
| *NA* | lmo1382 | unknown | Cations and iron carrying compounds | 1.79 | 0 | 0 | 0 | 2.65 |
| *NA* | lmo2373 | similar to phosphotransferase system (PTS) beta-glucoside-specific enzyme IIB component | Carbohydrates, organic alcohols, and acids | 1.79 | 0 | 2.1 | 0 | -0.83 |
| *NA* | LMOh7858_1060 | cation transport protein | Cations and iron carrying compounds | 1.73 | 0 | 0 | 0 | 0 |
| *NA* | lmo0559 | putative membrane protein | Amino acids, peptides and amines | 1.25 | 0 | 0 | 0 | 0.72 |
| *NA* | LMOf2365_1877 | manganese ABC transporter, ATP-binding protein | Cations and iron carrying compounds | -7.45 | 0 | 0 | 0 | 0 |
| *NA* | lmo0097 | similar to PTS system mannose-specific, factor IIC | Carbohydrates, organic alcohols, and acids | -6.17 | DOWN | -2.79 | 0 | -0.62 |
| *NA* | lmo1848 | similar metal cations ABC transporter (permease protein) | Cations and iron carrying compounds | -6.13 | 0 | 0 | 2.6 | 2.48 |
| *NA* | LMOf2365_1876 | manganese ABC transporter, permease protein | Cations and iron carrying compounds | -5.5 | 0 | 0 | 0 | 0 |
| *NA* | LMOh7858_1973 | manganese ABC transporter, permease protein | Cations and iron carrying compounds | -4.35 | 0 | 0 | 0 | 0 |
| *NA* | lmo1389 | similar to sugar ABC transporter, ATP-binding protein | Carbohydrates, organic alcohols, and acids | -4.31 | 0 | 0 | 0 | -0.52 |
| *uraA* | LMOf6854_1899 | uracil permease | Nucleosides, purines and pyrimidines | -4.23 | 0 | 0 | 0 | 0 |
| *NA* | lmo1074 | highly similar to teichoic acid translocation permease protein TagG | Amino acids, peptides and amines | -3.92 | 0 | 0 | 0 | -0.12 |
| *arpJ* | lmo2250 | similar to amino acid ABC transporter, permease protein | Amino acids, peptides and amines | -3.65 | 0 | 0 | 0 | -0.05 |
| *NA* | lmo0847 | similar to Glutamine ABC transporter (binding and transport protein) | Amino acids, peptides and amines | -3.34 | 0 | -2.87 | 0 | -0.82 |
| *NA* | lmo1761 | similar to putative sodium-dependent transporter | Amino acids, peptides and amines | -3.14 | DOWN | 0 | 0 | 1.44 |
| *NA* | lmo1424 | similar to manganese transport proteins NRAMP | Amino acids, peptides and amines | -3.01 | 0 | 0 | 0 | 1.95 |
| *NA* | lmo0981 | similar to efflux transporter | Other | -3 | 0 | 0 | 0 | -1.12 |
| *NA* | lmo1391 | similar to sugar ABC transporter, permease protein | Carbohydrates, organic alcohols, and acids | -2.84 | DOWN | 0 | 0 | 0.57 |
| *NA* | lmo0096 | similar to PTS system mannose-specific, factor IIAB | Carbohydrates, organic alcohols, and acids | -2.84 | DOWN | -2.74 | 0 | -0.9 |
| *uraA* | LMOf2365_1867 | uracil permease | Nucleosides, purines and pyrimidines | -2.76 | 0 | 0 | 0 | 0 |
| *NA* | lmo0605 | conserved hypothetical membrane protein | Cations and iron carrying compounds | -2.75 | 0 | 0 | 0 | 0.66 |
| *NA* | lmo0135 | similar to oligopeptide ABC transport system substrate-binding proteins | Amino acids, peptides and amines | -2.7 | 0 | 0 | 0 | 1.26 |
| *NA* | lmo1390 | similar to ABC transporter (permease proteins) | Carbohydrates, organic alcohols, and acids | -2.69 | DOWN | 0 | 0 | -2.37 |
| *NA* | LMOf2365_1002 | drug resistance transporter, EmrB/QacA family | Other | -2.52 | 0 | 0 | 0 | 0 |
| *NA* | lmo1255 | similar to PTS system trehalose specific enzyme IIBC | Carbohydrates, organic alcohols, and acids | -2.48 | 0 | 0 | 0 | 1.46 |
| *NA* | lmo2469 | similar to amino acid transporter | Amino acids, peptides and amines | -2.46 | 0 | 0 | 0 | 1.03 |
| *NA* | LMOf2365_0548 | drug resistance transporter, EmrB/QacA family | Other | -2.36 | 0 | 0 | 0 | 0 |
| *NA* | lmo0098 | similar to PTS system mannose-specific, factor IID | Carbohydrates, organic alcohols, and acids | -2.36 | DOWN | -3.3 | 0 | -0.33 |
| *NA* | lmo1846 | similar to conserved hypothetical proteins | Cations and iron carrying compounds | -2.23 | 0 | 0 | 0 | 1.58 |
| *NA* | LMOf6854_0560 | drug resistance transporter, EmrB/QacA family | Other | -2.22 | 0 | 0 | 0 | 0 |
| *lmrB* | LMOf6854_0559 | drug resistance transporter, EmrB/QacA subfamily | Other | -2.16 | 0 | 0 | 0 | 0 |
| *NA* | lmo0848 | similar to amino acid ABC transporter, ATP-binding protein | Amino acids, peptides and amines | -2.05 | 0 | 0 | 0 | -1.23 |
| *NA* | LMOf6854_2021 | iron compound ABC transporter, ATP-binding protein | Cations and iron carrying compounds | -2 | 0 | 0 | 0 | 0 |
| *NA* | lmo0519 | similar to multidrug resistance protein | Amino acids, peptides and amines | -1.98 | DOWN | 0 | 0 | 1.75 |
| *treB* | LMOf6854_1298 | PTS system, trehalose-specific, IIBC component | Carbohydrates, organic alcohols, and acids | -1.97 | 0 | 0 | 0 | 0 |
| *NA* | lmo2355 | Similar to multidrug resistance protein | Amino acids, peptides and amines | -1.92 | 0 | 0 | 0 | -1.31 |
| *NA* | LMOf6854_1148 | PTS system, IIB component, putative | Carbohydrates, organic alcohols, and acids | -1.86 | 0 | 0 | 0 | 0 |
| *NA* | lmo2249 | similar to low-affinity inorganic phosphate transporter | Amino acids, peptides and amines | -1.82 | DOWN | 0 | 0 | -1.49 |
| *NA* | lmo2195 | similar to oligopeptide ABC transporter (permease) | Amino acids, peptides and amines | -1.72 | DOWN | 0 | 0 | -0.72 |
| *zurM* | lmo1446 | metal (zinc) transport protein (ABC transporter, permease protein) | Amino acids, peptides and amines | -1.72 | 0 | 0 | 0 | 1.81 |
| *NA* | lmo2251 | similar to amino acid ABC transporter (ATP-binding protein) | Amino acids, peptides and amines | -1.7 | 0 | 0 | 0 | -0.22 |
| *NA* | lmo0136 | similar to oligopeptide ABC transporter, permease protein | Amino acids, peptides and amines | -1.65 | 0 | -5.48 | 0 | -0.19 |
| *NA* | lmo2845 | similar to transmembrane efflux proteins | Other | -1.65 | 0 | 0 | 0 | -0.91 |
| *sirB* | LMOf6854_2019 | ferrichrome ABC transporter, permease protein | Cations and iron carrying compounds | -1.63 | 0 | 0 | 0 | 0 |
| *NA* | LMOf2365_2333 | amino acid antiporter | Amino acids, peptides and amines | -1.63 | 0 | 0 | 0 | 0 |
| *NA* | lmo2483 | HPr-P(Ser) kinase/phosphatase | Carbohydrates, organic alcohols, and acids | -1.6 | 0 | 0 | 0 | 1.35 |
| *gbuB* | lmo1015 | highly similar to glycine betaine ABC transporters (permease) | Amino acids, peptides and amines | -1.58 | DOWN | 0 | 0 | -2.52 |
| *NA* | lmo2193 | similar to oligopeptide ABC transporter (ATP-binding protein) | Amino acids, peptides and amines | -1.58 | DOWN | 0 | 0 | -2.18 |
| *NA* | lmo1003 | phosphotransferase system enzyme I | Carbohydrates, organic alcohols, and acids | -1.57 | DOWN | 0 | 0 | -2.47 |
| *NA* | lmo2725 | conserved hypothetical proteins | Cations and iron carrying compounds | -1.53 | 0 | 0 | 0 | 2.36 |
| *NA* | LMOf6854_2316 | amino acid ABC transporter, permease protein, His/Glu/Gln/Arg/opine family | Amino acids, peptides and amines | -1.52 | 0 | 0 | 0 | 0 |
| *NA* | LMOh7858_2016 | Fibronectin type III domain protein | Other | -1.49 | 0 | 0 | 0 | 0 |
| *NA* | LMOh7858_0160 | oligopeptide ABC transporter, oligopeptide-binding protein | Amino acids, peptides and amines | -1.48 | 0 | 0 | 0 | 0 |
| *NA* | lmo1370 | similar to branched-chain fatty-acid kinase | Amino acids, peptides and amines | -1.47 | 0 | 0 | 0 | 0.88 |
| *NA* | lmo0524 | similar to putative sulfate transporter | Anions | -1.42 | 0 | 0 | 0 | 1.47 |
| *NA* | lmo2347 | similar to amino acid ABC transporter (permease) | Amino acids, peptides and amines | -1.39 | 0 | 0 | 0 | -2.87 |
| *NA* | lmo2369 | similar to B. subtilis general stress protein 13 containing a ribosomal S1 protein domain | Amino acids, peptides and amines | -1.33 | 0 | 0 | 0 | -0.03 |
| *NA* | lmo2369 | similar to B. subtilis general stress protein 13 containing a ribosomal S1 protein domain | Carbohydrates, organic alcohols, and acids | -1.33 | 0 | 0 | 0 | -0.03 |
| *NA* | lmo2369 | similar to B. subtilis general stress protein 13 containing a ribosomal S1 protein domain | Cations and iron carrying compounds | -1.33 | 0 | 0 | 0 | -0.03 |
| *NA* | LMOf6854_1754 | cation efflux family protein | Cations and iron carrying compounds | -1.32 | 0 | 0 | 0 | 0 |
| *NA* | lmo1019 | similar to B. subtilis YitL protein | Amino acids, peptides and amines | -1.3 | 0 | 0 | 0 | 1.09 |
| *NA* | lmo1019 | similar to B. subtilis YitL protein | Carbohydrates, organic alcohols, and acids | -1.3 | 0 | 0 | 0 | 1.09 |
| *NA* | lmo1019 | similar to B. subtilis YitL protein | Cations and iron carrying compounds | -1.3 | 0 | 0 | 0 | 1.09 |
| *NA* | lmo1884 | similar to xanthine permeases | Nucleosides, purines and pyrimidines | -1.28 | 0 | 0 | 0 | 1.05 |
| *NA* | lmo0021 | similar to PTS system, fructose-specific IIA component | Carbohydrates, organic alcohols, and acids | 0 | UP | 0 | 0 | 0.51 |
| *NA* | lmo0037 | similar to amino acid transporter | Amino acids, peptides and amines | 0 | UP | 0 | 0 | 3.97 |
| *NA* | lmo0405 | similar to phosphate transport protein | Amino acids, peptides and amines | 0 | UP | 0 | 2 | 1.32 |
| *NA* | lmo0781 | similar to mannose-specific phosphotransferase system (PTS) component IID | Carbohydrates, organic alcohols, and acids | 0 | UP | 0 | 2.9 | -0.54 |
| *NA* | lmo0782 | similar to mannose-specific phosphotransferase system (PTS) component IIC | Carbohydrates, organic alcohols, and acids | 0 | UP | 0 | 10.3 | -0.04 |
| *NA* | lmo0859 | similar to putative sugar ABC transporter, periplasmic sugar-binding protein | Carbohydrates, organic alcohols, and acids | 0 | UP | 0 | 0 | 0.29 |
| *NA* | lmo0860 | similar to sugar ABC transporter, permease protein | Carbohydrates, organic alcohols, and acids | 0 | UP | 0 | 0 | -1.25 |
| *NA* | lmo0897 | similar to transport proteins | Amino acids, peptides and amines | 0 | UP | 0 | 0 | -0.78 |
| *NA* | lmo0979 | similar to daunorubicin resistance ATP-binding proteins | Amino acids, peptides and amines | 0 | UP | 0 | 0 | -1.04 |
| *NA* | lmo0990 | conserved hypothetical protein | Cations and iron carrying compounds | 0 | UP | 0 | 0 | -2.26 |
| *glpF* | lmo1167 | similar to glycerol uptake facilitator protein | Other | 0 | UP | 0 | 0 | -0.17 |
| *NA* | lmo1300 | similar to arsenic efflux pump protein | Anions | 0 | UP | -2.42 | 0 | 1.95 |
| *NA* | lmo1300 | similar to arsenic efflux pump protein | Cations and iron carrying compounds | 0 | UP | -2.42 | 0 | 1.95 |
| *NA* | lmo1421 | similar to glycine betaine/carnitine/choline ABC transporter (ATP-binding protein) | Amino acids, peptides and amines | 0 | UP | 0 | 3.1 | -0.48 |
| *opuCD* | lmo1425 | similar to betaine/carnitine/choline ABC transporter (membrane p) | Amino acids, peptides and amines | 0 | UP | 2.7 | 7 | 1.02 |
| *opuCA* | lmo1428 | similar to glycine betaine/carnitine/choline ABC transporter (ATP-binding protein) | Amino acids, peptides and amines | 0 | UP | 4.41 | 10.1 | -0.18 |
| *NA* | lmo1971 | similar to pentitol PTS system enzyme II C component | Carbohydrates, organic alcohols, and acids | 0 | UP | 0 | 0 | 0.87 |
| *NA* | lmo1973 | similar to PTS system enzyme II A component | Carbohydrates, organic alcohols, and acids | 0 | UP | 0 | 0 | 0.8 |
| *NA* | lmo1997 | similar to PTS mannose-specific enzyme IIA component | Carbohydrates, organic alcohols, and acids | 0 | UP | 0 | 0 | -0.99 |
| *NA* | lmo2000 | similar to PTS mannose-specific enzyme IID component | Carbohydrates, organic alcohols, and acids | 0 | UP | 0 | 0 | 1.38 |
| *NA* | lmo2001 | similar to PTS mannose-specific enzyme IIC component | Carbohydrates, organic alcohols, and acids | 0 | UP | 0 | 0 | -0.63 |
| *NA* | lmo2002 | similar to PTS mannose-specific enzyme IIB component | Carbohydrates, organic alcohols, and acids | 0 | UP | 0 | 0 | 0.12 |
| *NA* | lmo2027 | putative cell surface protein, similar to internalin proteins | Amino acids, peptides and amines | 0 | UP | 0 | 0 | 0.35 |
| *NA* | lmo2084 | unknown | Carbohydrates, organic alcohols, and acids | 0 | UP | 0 | 0 | 0.58 |
| *betL* | lmo2092 | glycine betaine transporter BetL | Amino acids, peptides and amines | 0 | UP | 0 | 0 | -0.11 |
| *NA* | lmo2136 | similar to PTS system, fructose-specific enzyme IIB component | Carbohydrates, organic alcohols, and acids | 0 | UP | 0 | 0 | 0.38 |
| *fruA* | lmo2335 | highly similar to phosphotransferase system (PTS) fructose-specific enzyme IIABC component | Carbohydrates, organic alcohols, and acids | 0 | UP | 13.03 | 0 | 0.19 |
| *NA* | lmo2496 | similar to phosphate ABC transporter (ATP-binding protein) | Anions | 0 | UP | 0 | 0 | -0.02 |
| *NA* | lmo2497 | similar to phosphate ABC transporter (permease protein) | Anions | 0 | UP | 0 | 0 | 0.52 |
| *NA* | lmo2498 | similar to phosphate ABC transporter (permease protein) | Anions | 0 | UP | 0 | 0 | 0.25 |
| *NA* | lmo2575 | similar to cation transport protein (efflux) | Cations and iron carrying compounds | 0 | UP | 0 | 1.1 | -0.21 |
| *NA* | lmo2588 | similar to drug-export proteins | Amino acids, peptides and amines | 0 | UP | 0 | 0 | 2.57 |
| *NA* | lmo2772 | similar to beta-glucoside-specific enzyme IIABC | Carbohydrates, organic alcohols, and acids | 0 | UP | 0 | 0 | 0.54 |
| *NA* | lmo2797 | similar to phosphotransferase system mannitol-specific enzyme IIA | Carbohydrates, organic alcohols, and acids | 0 | UP | 0 | 0 | -0.5 |
| *NA* | lmo2799 | similar to phosphotransferase system mannitol-specific enzyme IIBC | Carbohydrates, organic alcohols, and acids | 0 | UP | 0 | 0 | -1.05 |
| *ptsH* | lmo1002 | PTS phosphocarrier protein Hpr (histidine containing protein) | Carbohydrates, organic alcohols, and acids | 0 | DOWN | 0 | 0 | 1.5 |
| *gbuA* | lmo1014 | highly similar to glycine betaine ABC transporter (ATP-binding protein) | Amino acids, peptides and amines | 0 | DOWN | 0 | 2.1 | -2.25 |
| *gbuC* | lmo1016 | highly similar to glycine betaine ABC transporters (glycine betaine-binding protein) | Amino acids, peptides and amines | 0 | DOWN | 2.18 | 0 | -0.84 |
| *NA* | lmo1740 | similar to amino acid (glutamine) ABC transporter, permease protein | Amino acids, peptides and amines | 0 | DOWN | 0 | 0 | 1.06 |
| *NA* | lmo2097 | similar to PTS system galactitol-specific enzyme IIB component | Carbohydrates, organic alcohols, and acids | 0 | DOWN | 0 | 0 | 2.62 |
| *NA* | lmo2105 | similar to ferrous iron transport protein B | Cations and iron carrying compounds | 0 | DOWN | 0 | 0 | -0.12 |
| *NA* | lmo2650 | similar to hypothetical PTS enzyme IIB component | Carbohydrates, organic alcohols, and acids | 0 | DOWN | 0 | 0 | -0.26 |
| *NA* | lmo2651 | similar to mannitol-specific PTS enzyme IIA component | Carbohydrates, organic alcohols, and acids | 0 | DOWN | 0 | 0 | 0.8 |
| *NA* | lmo0986 | similar to antibiotic ABC transporter, ATP-binding protein | Other | 0 | 0 | 1.88 | 0 | 0.4 |
| *NA* | lmo0987 | similar to Streptococcus agalactiae CylB protein | Amino acids, peptides and amines | 0 | 0 | 7.55 | 0 | -0.95 |
| *NA* | lmo1845 | similar to conserved hypothetical proteins | Nucleosides, purines and pyrimidines | 0 | 0 | 2.22 | 0 | -0.07 |
| *NA* | lmo2362 | similar to amino acid antiporter (acid resistance) | Amino acids, peptides and amines | 0 | 0 | 2.31 | -1.4 | 0.41 |
| *NA* | lmo0645 | similar to amino acid transporter | Amino acids, peptides and amines | 0 | 0 | -2.09 | 0 | -0.81 |
| *NA* | lmo2652 | similar to transcriptional antiterminator | Carbohydrates, organic alcohols, and acids | 0 | 0 | -2.32 | 0 | 0.48 |
| *NA* | lmo0137 | similar to oligopeptide ABC transporter, permease protein | Amino acids, peptides and amines | 0 | 0 | 0 | 2.2 | -1.83 |
| *NA* | lmo0398 | similar to phosphotransferase system enzyme IIA | Carbohydrates, organic alcohols, and acids | 0 | 0 | 0 | -1.2 | 2.49 |
| *NA* | lmo0399 | similar to fructose-specific phosphotransferase enzyme IIB | Carbohydrates, organic alcohols, and acids | 0 | 0 | 0 | 1.1 | 0.67 |
| *NA* | lmo0400 | similar to fructose-specific phosphotransferase enzyme IIC | Carbohydrates, organic alcohols, and acids | 0 | 0 | 0 | 1 | 0.97 |
| *NA* | lmo0784 | similar to mannose-specific phosphotransferase system (PTS) component IIA | Carbohydrates, organic alcohols, and acids | 0 | 0 | 0 | 8.5 | 0.08 |
| *NA* | lmo0341 | unknown | Other | 0 | 0 | 0 | -1.3 | 0.81 |
| *NA* | lmo0027 | similar to PTS system, beta-glucosides specific enzyme IIABC | Carbohydrates, organic alcohols, and acids | 0 | 0 | 0 | -2 | 0.58 |
| *NA* | lmo0180 | similar to sugar ABC transporter, permease protein | Carbohydrates, organic alcohols, and acids | 0 | 0 | 0 | -1.1 | -0.58 |
| *NA* | lmo0503 | similar to PTS fructose-specific enzyme IIA component | Carbohydrates, organic alcohols, and acids | 0 | 0 | 0 | 1.4 | -0.58 |
| *NA* | lmo0541 | similar to ABC transporter (binding protein) | Cations and iron carrying compounds | 0 | 0 | 0 | -2.6 | 2.39 |
| *NA* | lmo0861 | similar to sugar ABC transporter, permease protein - | Carbohydrates, organic alcohols, and acids | 0 | 0 | 0 | 1.1 | -1.19 |
| *NA* | lmo1285 | conserved hypothetical protein, similar to B. subtilis YneT protein | Amino acids, peptides and amines | 0 | 0 | 0 | -2.1 | -0.67 |
| *NA* | lmo1285 | conserved hypothetical protein, similar to B. subtilis YneT protein | Carbohydrates, organic alcohols, and acids | 0 | 0 | 0 | -2.1 | -0.67 |
| *NA* | lmo1285 | conserved hypothetical protein, similar to B. subtilis YneT protein | Cations and iron carrying compounds | 0 | 0 | 0 | -2.1 | -0.67 |
| *NA* | lmo0003 | conserved hypothetical protein | Cations and iron carrying compounds | 0 | 0 | 0 | 0 | 1.29 |
| *NA* | lmo0022 | similar to PTS system, fructose-specific IIB component | Carbohydrates, organic alcohols, and acids | 0 | 0 | 0 | 0 | 1.08 |
| *NA* | lmo0023 | similar to PTS system, fructose-specific IIC component | Carbohydrates, organic alcohols, and acids | 0 | 0 | 0 | 0 | 2.84 |
| *NA* | lmo0024 | similar to PTS system, mannose-specific IID component | Carbohydrates, organic alcohols, and acids | 0 | 0 | 0 | 0 | 1.07 |
| *NA* | lmo0026 | similar to E. coli copper homeostasis protein CutC | Cations and iron carrying compounds | 0 | 0 | 0 | 0 | -0.56 |
| *NA* | lmo0034 | similar to PTS system, cellobiose-specific IIC component | Carbohydrates, organic alcohols, and acids | 0 | 0 | 0 | 0 | -1.53 |
| *NA* | lmo0155 | similar to high-affinity zinc ABC transporter (membrane protein) | Amino acids, peptides and amines | 0 | 0 | 0 | 0 | -0.35 |
| *NA* | lmo0155 | similar to high-affinity zinc ABC transporter (membrane protein) | Cations and iron carrying compounds | 0 | 0 | 0 | 0 | -0.35 |
| *NA* | lmo0179 | similar to sugar ABC transporters, permease proteins | Carbohydrates, organic alcohols, and acids | 0 | 0 | 0 | 0 | 0.46 |
| *NA* | lmo0278 | similar to sugar ABC transporter, ATP-binding protein | Carbohydrates, organic alcohols, and acids | 0 | 0 | 0 | 0 | -0.84 |
| *NA* | lmo0298 | similar to PTS beta-glucoside-specific enzyme IIC component | Carbohydrates, organic alcohols, and acids | 0 | 0 | 0 | 0 | 0.45 |
| *NA* | lmo0299 | similar to PTS beta-glucoside-specific enzyme IIB component | Carbohydrates, organic alcohols, and acids | 0 | 0 | 0 | 0 | 1.47 |
| *NA* | lmo0301 | similar to PTS beta-glucoside-specific enzyme IIA component | Carbohydrates, organic alcohols, and acids | 0 | 0 | 0 | 0 | 0.34 |
| *NA* | lmo0327 | similar to cell surface proteins (LPXTG motif) | Amino acids, peptides and amines | 0 | 0 | 0 | 0 | -1.43 |
| *NA* | lmo0357 | similar to PTS system, enzyme IIA component | Carbohydrates, organic alcohols, and acids | 0 | 0 | 0 | 0 | -5.42 |
| *NA* | lmo0358 | similar to PTS system, fructose-specific enzyme IIBC component | Carbohydrates, organic alcohols, and acids | 0 | 0 | 0 | 0 | 2 |
| *NA* | lmo0373 | similar to PTS betaglucoside-specific enzyme IIC component | Carbohydrates, organic alcohols, and acids | 0 | 0 | 0 | 0 | 0.73 |
| *NA* | lmo0374 | similar to PTS betaglucoside-specific enzyme IIB component | Carbohydrates, organic alcohols, and acids | 0 | 0 | 0 | 0 | 0.9 |
| *NA* | lmo0424 | similar to Staphylococcus xylosus glucose uptake protein | Carbohydrates, organic alcohols, and acids | 0 | 0 | 0 | 0 | 1.13 |
| *NA* | lmo0426 | similar to PTS fructose-specific enzyme IIA component | Carbohydrates, organic alcohols, and acids | 0 | 0 | 0 | 0 | 0.84 |
| *NA* | lmo0428 | similar to PTS fructose-specific enzyme IIC component | Carbohydrates, organic alcohols, and acids | 0 | 0 | 0 | 0 | 0.42 |
| *NA* | lmo0454 | conserved hypothetical protein similar to B. subtilis YeaC | Cations and iron carrying compounds | 0 | 0 | 0 | 0 | 0.35 |
| *NA* | lmo0456 | similar to permeases | Nucleosides, purines and pyrimidines | 0 | 0 | 0 | 0 | -0.16 |
| *NA* | lmo0507 | similar to PTS system, Galactitol-specific IIB component | Carbohydrates, organic alcohols, and acids | 0 | 0 | 0 | 0 | 0.76 |
| *NA* | lmo0508 | similar to PTS system, Galactitol-specific IIC component | Carbohydrates, organic alcohols, and acids | 0 | 0 | 0 | 0 | -0.03 |
| *NA* | lmo0542 | similar to PTS system, glucitol/sorbitol-specific enzyme IIA component | Carbohydrates, organic alcohols, and acids | 0 | 0 | 0 | 0 | 3.44 |
| *NA* | lmo0543 | similar to PTS system, glucitol/sorbitol-specific enzyme IIBC component | Carbohydrates, organic alcohols, and acids | 0 | 0 | 0 | 0 | 0.48 |
| *NA* | lmo0544 | similar to PTS system, glucitol/sorbitol-specific enzyme II CII component | Carbohydrates, organic alcohols, and acids | 0 | 0 | 0 | 0 | -0.13 |
| *NA* | lmo0549 | similar to internalin protein | Amino acids, peptides and amines | 0 | 0 | 0 | 0 | 2.7 |
| *NA* | lmo0573 | conserved hypothetical protein | Nucleosides, purines and pyrimidines | 0 | 0 | 0 | 0 | 0.79 |
| *NA* | lmo0631 | similar to PTS system, fructose-specific IIA component | Carbohydrates, organic alcohols, and acids | 0 | 0 | 0 | 0 | 1.56 |
| *NA* | lmo0632 | similar to PTS system, fructose-specific IIC component | Carbohydrates, organic alcohols, and acids | 0 | 0 | 0 | 0 | 1.34 |
| *NA* | lmo0633 | similar to PTS system, fructose-specific IIB component | Carbohydrates, organic alcohols, and acids | 0 | 0 | 0 | 0 | 0.47 |
| *NA* | lmo0641 | similar to heavy metal-transporting ATPase | Cations and iron carrying compounds | 0 | 0 | 0 | 0 | -3.78 |
| *NA* | lmo0648 | similar to membrane proteins | Amino acids, peptides and amines | 0 | 0 | 0 | 0 | -0.24 |
| *NA* | lmo0668 | similar to putative ABC transporter, permease protein | Amino acids, peptides and amines | 0 | 0 | 0 | 0 | -0.67 |
| *NA* | lmo0721 | putative fibronectin-binding protein | Other | 0 | 0 | 0 | 0 | 1.37 |
| *NA* | lmo0738 | similar to phosphotransferase system (PTS) beta-glucoside-specific enzyme IIABC component | Carbohydrates, organic alcohols, and acids | 0 | 0 | 0 | 0 | 0.9 |
| *NA* | lmo0766 | similar to putative sugar ABC transporter, permease protein | Carbohydrates, organic alcohols, and acids | 0 | 0 | 0 | 0 | -0.81 |
| *NA* | lmo0767 | similar to ABC transporter, permease protein | Carbohydrates, organic alcohols, and acids | 0 | 0 | 0 | 0 | -3.01 |
| *NA* | lmo0787 | similar to amino acid transporter | Amino acids, peptides and amines | 0 | 0 | 0 | 0 | -0.38 |
| *NA* | lmo0798 | similar to lysine-specific permease | Amino acids, peptides and amines | 0 | 0 | 0 | 0 | 0.29 |
| *NA* | lmo0803 | similar to putative Na+/H+ antiporter | Cations and iron carrying compounds | 0 | 0 | 0 | 0 | -1.18 |
| *NA* | lmo0807 | similar to spermidine/putrescine ABC transporter, ATP-binding protein | Amino acids, peptides and amines | 0 | 0 | 0 | 0 | 1.26 |
| *NA* | lmo0808 | similar to spermidine/putrescine ABC transporter, permease protein | Amino acids, peptides and amines | 0 | 0 | 0 | 0 | 2.57 |
| *NA* | lmo0810 | similar to spermidine/putrescine-binding protein | Amino acids, peptides and amines | 0 | 0 | 0 | 0 | 1.14 |
| *NA* | lmo0818 | similar to cation transporting ATPase | Cations and iron carrying compounds | 0 | 0 | 0 | 0 | 0.52 |
| *NA* | lmo0826 | similar to transport protein | Anions | 0 | 0 | 0 | 0 | 0.62 |
| *NA* | lmo0826 | similar to transport protein | Carbohydrates, organic alcohols, and acids | 0 | 0 | 0 | 0 | 0.62 |
| *NA* | lmo0874 | similar to PTS system enzyme IIA component | Carbohydrates, organic alcohols, and acids | 0 | 0 | 0 | 0 | -1.09 |
| *NA* | lmo0875 | similar to PTS system, beta-glucoside enzyme IIB component | Carbohydrates, organic alcohols, and acids | 0 | 0 | 0 | 0 | -1.86 |
| *NA* | lmo0876 | similar to PTS system, Lichenan-specific enzyme IIC component | Carbohydrates, organic alcohols, and acids | 0 | 0 | 0 | 0 | 0.1 |
| *NA* | lmo0898 | conserved hypothetical protein | Amino acids, peptides and amines | 0 | 0 | 0 | 0 | 0.67 |
| *NA* | lmo0898 | conserved hypothetical protein | Carbohydrates, organic alcohols, and acids | 0 | 0 | 0 | 0 | 0.67 |
| *NA* | lmo0898 | conserved hypothetical protein | Cations and iron carrying compounds | 0 | 0 | 0 | 0 | 0.67 |
| *NA* | lmo0901 | similar to PTS system, cellobiose-specific IIC component | Carbohydrates, organic alcohols, and acids | 0 | 0 | 0 | 0 | 0.39 |
| *NA* | lmo0914 | similar to PTS system, IIB component | Carbohydrates, organic alcohols, and acids | 0 | 0 | 0 | 0 | -1 |
| *NA* | lmo0915 | similar to phosphotransferase system enzyme IIC | Carbohydrates, organic alcohols, and acids | 0 | 0 | 0 | 0 | -1.17 |
| *NA* | lmo0916 | similar to phosphotransferase system enzyme IIA | Carbohydrates, organic alcohols, and acids | 0 | 0 | 0 | 0 | -1.72 |
| *NA* | lmo0925 | putative membrane protein | Amino acids, peptides and amines | 0 | 0 | 0 | 0 | -1.21 |
| *NA* | lmo0947 | hypothetical transport protein | Other | 0 | 0 | 0 | 0 | -0.94 |
| *NA* | lmo0980 | similar to ABC transporter transmembrane component | Amino acids, peptides and amines | 0 | 0 | 0 | 0 | -0.62 |
| *NA* | lmo1017 | similar to phosphotransferase system glucose-specific enzyme IIA | Carbohydrates, organic alcohols, and acids | 0 | 0 | 0 | 0 | -0.02 |
| *NA* | lmo1023 | similar to a bacterial K(+)-uptake system | Cations and iron carrying compounds | 0 | 0 | 0 | 0 | -1.16 |
| *NA* | lmo1035 | similar to phosphotransferase system (PTS) beta-glucoside-specific enzyme IIABC | Carbohydrates, organic alcohols, and acids | 0 | 0 | 0 | 0 | -0.55 |
| *NA* | lmo1039 | similar to ABC transporter, ATP-binding protein | Anions | 0 | 0 | 0 | 0 | -1.6 |
| *NA* | lmo1041 | similar to molybdate ABC transporter binding protein | Cations and iron carrying compounds | 0 | 0 | 0 | 0 | -1.87 |
| *NA* | lmo1064 | similar to membrane and transport proteins | Cations and iron carrying compounds | 0 | 0 | 0 | 0 | 0.68 |
| *NA* | lmo1073 | similar to metal binding protein (ABC transporter) | Cations and iron carrying compounds | 0 | 0 | 0 | 0 | -0.18 |
| *NA* | lmo1073 | similar to metal binding protein (ABC transporter) | Other | 0 | 0 | 0 | 0 | -0.18 |
| *NA* | lmo1095 | similar to PTS system, cellobiose-specific IIB component (cel A) | Carbohydrates, organic alcohols, and acids | 0 | 0 | 0 | 0 | -0.94 |
| *cadA* | lmo1100 | cadmium resistance protein | Cations and iron carrying compounds | 0 | 0 | 0 | 0 | -1.6 |
| *NA* | lmo1141 | similar to uroporphyrin-III C-methyltransferase | Carbohydrates, organic alcohols, and acids | 0 | 0 | 0 | 0 | -0.18 |
| *NA* | lmo1205 | similar to putative cobalt transport protein CbiN | Cations and iron carrying compounds | 0 | 0 | 0 | 0 | 0.59 |
| *cbiQ* | lmo1206 | similar to cobalt transport protein Q | Cations and iron carrying compounds | 0 | 0 | 0 | 0 | 0.23 |
| *NA* | lmo1207 | similar to cobalt transport ATP-binding protein CbiO | Cations and iron carrying compounds | 0 | 0 | 0 | 0 | 0.44 |
| *NA* | lmo1224 | similar to different proteins | Amino acids, peptides and amines | 0 | 0 | 0 | 0 | -1.22 |
| *NA* | lmo1289 | similar to internalin proteins, putative peptidoglycan bound protein (LPXTG motif) | Amino acids, peptides and amines | 0 | 0 | 0 | 0 | 0.56 |
| *NA* | lmo1409 | similar to multidrug-efflux transporter | Other | 0 | 0 | 0 | 0 | 0.6 |
| *NA* | lmo1516 | similar to ammonium transporter NrgA | Cations and iron carrying compounds | 0 | 0 | 0 | 0 | 1.02 |
| *pheT* | lmo1607 | similar phenylalanyl-tRNA synthetase (beta subunit) | Amino acids, peptides and amines | 0 | 0 | 0 | 0 | -0.9 |
| *pheT* | lmo1607 | similar phenylalanyl-tRNA synthetase (beta subunit) | Carbohydrates, organic alcohols, and acids | 0 | 0 | 0 | 0 | -0.9 |
| *pheT* | lmo1607 | similar phenylalanyl-tRNA synthetase (beta subunit) | Cations and iron carrying compounds | 0 | 0 | 0 | 0 | -0.9 |
| *NA* | lmo1617 | similar to multidrug-efflux transporter | Amino acids, peptides and amines | 0 | 0 | 0 | 0 | 0.18 |
| *NA* | lmo1719 | similar to phosphotransferase system (PTS) lichenan-specific enzyme IIA component | Carbohydrates, organic alcohols, and acids | 0 | 0 | 0 | 0 | 1.67 |
| *NA* | lmo1730 | similar to sugar ABC transporter binding protein | Carbohydrates, organic alcohols, and acids | 0 | 0 | 0 | 0 | 0.13 |
| *NA* | lmo1731 | similar to sugar ABC transporter, permease protein | Carbohydrates, organic alcohols, and acids | 0 | 0 | 0 | 0 | 1.63 |
| *NA* | lmo1732 | similar to sugar ABC transporter, permease protein | Carbohydrates, organic alcohols, and acids | 0 | 0 | 0 | 0 | -1.25 |
| *NA* | lmo1738 | similar to amino acid ABC transporter (binding protein) | Amino acids, peptides and amines | 0 | 0 | 0 | 0 | 0.43 |
| *NA* | lmo1739 | similar to amino acid (glutamine) ABC transporter (ATP-binding protein) | Amino acids, peptides and amines | 0 | 0 | 0 | 0 | -0.16 |
| *pyrP* | lmo1839 | highly similar to uracil permease | Nucleosides, purines and pyrimidines | 0 | 0 | 0 | 0 | 0.91 |
| *NA* | lmo1852 | similar to putative mercuric ion binding proteins | Cations and iron carrying compounds | 0 | 0 | 0 | 0 | 1.55 |
| *NA* | lmo1853 | similar to heavy metal-transporting ATPases | Cations and iron carrying compounds | 0 | 0 | 0 | 0 | 0.39 |
| *NA* | lmo1889 | similar to conserved hypothetical proteins | Amino acids, peptides and amines | 0 | 0 | 0 | 0 | 0.04 |
| *NA* | lmo1889 | similar to conserved hypothetical proteins | Cations and iron carrying compounds | 0 | 0 | 0 | 0 | 0.04 |
| *fhuG* | lmo1957 | similar to ferrichrome ABC transporter (permease) | Amino acids, peptides and amines | 0 | 0 | 0 | 0 | -1.55 |
| *fhuB* | lmo1958 | similar to ferrichrome ABC transporter (permease) | Amino acids, peptides and amines | 0 | 0 | 0 | 0 | 2.93 |
| *fhuB* | lmo1958 | similar to ferrichrome ABC transporter (permease) | Cations and iron carrying compounds | 0 | 0 | 0 | 0 | 2.93 |
| *fhuC* | lmo1960 | similar to ferrichrome ABC transporter (ATP-binding protein) | Cations and iron carrying compounds | 0 | 0 | 0 | 0 | 1.12 |
| *NA* | lmo2026 | putative peptidoglycan bound protein (LPXTG motif) | Amino acids, peptides and amines | 0 | 0 | 0 | 0 | -0.65 |
| *NA* | lmo2044 | similar to transporter binding proteins | Amino acids, peptides and amines | 0 | 0 | 0 | 0 | -2.56 |
| *NA* | lmo2059 | similar to potassium channel subunit | Cations and iron carrying compounds | 0 | 0 | 0 | 0 | 1.32 |
| *NA* | lmo2096 | similar to PTS system galactitol-specific enzyme IIC component | Carbohydrates, organic alcohols, and acids | 0 | 0 | 0 | 0 | 0.32 |
| *NA* | lmo2098 | similar to PTS system galactitol-specific enzyme IIA component | Carbohydrates, organic alcohols, and acids | 0 | 0 | 0 | 0 | -1.94 |
| *NA* | lmo2135 | similar to PTS system, fructose-specific enzyme IIC component | Carbohydrates, organic alcohols, and acids | 0 | 0 | 0 | 0 | 0.08 |
| *NA* | lmo2137 | similar to PTS system, fructose-specific enzyme IIA component | Carbohydrates, organic alcohols, and acids | 0 | 0 | 0 | 0 | -1.49 |
| *NA* | lmo2171 | similar to antiporter proteins | Carbohydrates, organic alcohols, and acids | 0 | 0 | 0 | 0 | -0.6 |
| *NA* | lmo2183 | similar to ferrichrome ABC transporter (permease) | Amino acids, peptides and amines | 0 | 0 | 0 | 0 | -4.39 |
| *NA* | lmo2192 | similar to oligopeptide ABC transporter (ATP-binding protein) | Amino acids, peptides and amines | 0 | 0 | 0 | 0 | -0.3 |
| *NA* | lmo2237 | similar to transport system permease protein | Other | 0 | 0 | 0 | 0 | -0.37 |
| *NA* | lmo2254 | similar to unknown proteins | Nucleosides, purines and pyrimidines | 0 | 0 | 0 | 0 | -1.56 |
| *NA* | lmo2259 | similar to phosphotransferase system (PTS) beta-glucoside-specific enzyme IIA | Carbohydrates, organic alcohols, and acids | 0 | 0 | 0 | 0 | -0.22 |
| *NA* | lmo2346 | similar to amino acid ABC-transporter, ATP-binding protein | Amino acids, peptides and amines | 0 | 0 | 0 | 0 | -1.44 |
| *NA* | lmo2353 | similar to putative Na+/H+ antiporter | Cations and iron carrying compounds | 0 | 0 | 0 | 0 | 0.23 |
| *NA* | lmo2371 | similar to putative ABC-transporter transmembrane subunit | Amino acids, peptides and amines | 0 | 0 | 0 | 0 | 0.59 |
| *NA* | lmo2377 | similar to multi-drug resistance efflux pump | Other | 0 | 0 | 0 | 0 | -1.39 |
| *NA* | lmo2379 | similar to proteins involved in resistance to cholate and to NA(+) and in pH homeostasis | Cations and iron carrying compounds | 0 | 0 | 0 | 0 | 0.2 |
| *NA* | lmo2380 | similar to proteins involved in resistance to cholate and to NA(+) and in pH homeostasis | Cations and iron carrying compounds | 0 | 0 | 0 | 0 | -2.25 |
| *NA* | lmo2396 | similar to internalin proteins, putative peptidoglycan bound protein (LPXTG motif) | Amino acids, peptides and amines | 0 | 0 | 0 | 0 | -0.83 |
| *NA* | lmo2429 | similar to B. subtilis ferrichrome ABC transporter (ATP-binding protein) FhuC | Cations and iron carrying compounds | 0 | 0 | 0 | 0 | -0.75 |
| *NA* | lmo2430 | similar to B. subtilis ferrichrome ABC transporter (permease) FhuG | Amino acids, peptides and amines | 0 | 0 | 0 | 0 | -3.08 |
| *NA* | lmo2445 | similar to internalin | Amino acids, peptides and amines | 0 | 0 | 0 | 0 | 0.94 |
| *NA* | lmo2470 | similar to internalin proteins | Cations and iron carrying compounds | 0 | 0 | 0 | 0 | 1.53 |
| *NA* | lmo2499 | similar to phosphate ABC transporter (binding protein) | Anions | 0 | 0 | 0 | 0 | -0.61 |
| *NA* | lmo2581 | conserved hypothetical protein | Amino acids, peptides and amines | 0 | 0 | 0 | 0 | 1.41 |
| *NA* | lmo2599 | highly similar to B. subtilis YbaF protein | Cations and iron carrying compounds | 0 | 0 | 0 | 0 | -1.78 |
| *NA* | lmo2601 | similar to ABC transporter (ATP-binding protein) | Cations and iron carrying compounds | 0 | 0 | 0 | 0 | -0.12 |
| *NA* | lmo2665 | similar to PTS system galactitol-specific enzyme IIC component | Carbohydrates, organic alcohols, and acids | 0 | 0 | 0 | 0 | 1.29 |
| *NA* | lmo2666 | similar to PTS system galactitol-specific enzyme IIB component | Carbohydrates, organic alcohols, and acids | 0 | 0 | 0 | 0 | 2.58 |
| *NA* | lmo2667 | similar to PTS system galactitol-specific enzyme IIA component | Carbohydrates, organic alcohols, and acids | 0 | 0 | 0 | 0 | 1.15 |
| *kdpC* | lmo2680 | potassium-transporting atpase c chain | Cations and iron carrying compounds | 0 | 0 | 0 | 0 | 0.1 |
| *kdpB* | lmo2681 | potassium-transporting atpase b chain | Cations and iron carrying compounds | 0 | 0 | 0 | 0 | 1.05 |
| *kdpA* | lmo2682 | highly similar to potassium-transporting atpase a chain | Cations and iron carrying compounds | 0 | 0 | 0 | 0 | -0.51 |
| *NA* | lmo2684 | similar to cellobiose phosphotransferase enzyme IIC component | Carbohydrates, organic alcohols, and acids | 0 | 0 | 0 | 0 | 0.35 |
| *NA* | lmo2685 | similar to cellobiose phosphotransferase enzyme IIA component | Carbohydrates, organic alcohols, and acids | 0 | 0 | 0 | 0 | -0.29 |
| *NA* | lmo2689 | highly similar to Mg2+ transport ATPase | Cations and iron carrying compounds | 0 | 0 | 0 | 0 | -1.4 |
| *NA* | lmo2708 | similar to PTS system, cellobiose-specific enzyme IIC | Carbohydrates, organic alcohols, and acids | 0 | 0 | 0 | 0 | -1.37 |
| *NA* | lmo2722 | similar to merR-family transcriptional regulator | Other | 0 | 0 | 0 | 0 | -1.83 |
| *NA* | lmo2733 | similar to PTS system, fructose-specific IIABC component | Carbohydrates, organic alcohols, and acids | 0 | 0 | 0 | 0 | 2.91 |
| *NA* | lmo2762 | similar to PTS cellobiose-specific enzyme IIB | Carbohydrates, organic alcohols, and acids | 0 | 0 | 0 | 0 | 1.16 |
| *NA* | lmo2763 | similar to PTS cellobiose-specific enzyme IIC | Carbohydrates, organic alcohols, and acids | 0 | 0 | 0 | 0 | -0.16 |
| *NA* | lmo2765 | similar to PTS cellobiose-specific enzyme IIA | Carbohydrates, organic alcohols, and acids | 0 | 0 | 0 | 0 | 0.67 |
| *NA* | lmo2777 | similar to efflux protein | Amino acids, peptides and amines | 0 | 0 | 0 | 0 | -0.53 |
| *NA* | lmo2780 | similar to cellobiose PTS enzyme IIA | Carbohydrates, organic alcohols, and acids | 0 | 0 | 0 | 0 | -0.47 |
| *NA* | lmo2782 | similar to PTS, cellobiose-specific IIB component | Carbohydrates, organic alcohols, and acids | 0 | 0 | 0 | 0 | 0.46 |
| *NA* | lmo2783 | similar to cellobiose phosphotransferase system enzyme IIC | Carbohydrates, organic alcohols, and acids | 0 | 0 | 0 | 0 | 0 |
| *bvrB* | lmo2787 | beta-glucoside-specific phosphotransferase enzyme II ABC component | Carbohydrates, organic alcohols, and acids | 0 | 0 | 0 | 0 | 0.64 |
| *NA* | lmo2818 | similar to transmembrane efflux protein | Amino acids, peptides and amines | 0 | 0 | 0 | 0 | 0.87 |
| *NA* | lmo2821 | similar to internalin, Unknown, putative peptidoglycan bound protein (LPXTG motif) | Amino acids, peptides and amines | 0 | 0 | 0 | 0 | 1.31 |
| **DNA metabolism** | | |  |  |  |  |  |  |
| *NA* | lmo2676 | similar to UV-damage repair protein | DNA replication, recombination, and repair | 3.48 | UP | 0 | 0 | 1.93 |
| *uvrB* | lmo2489 | excinuclease ABC (subunit B) | DNA replication, recombination, and repair | 3.42 | UP | 0 | 0 | -0.96 |
| *recA* | lmo1398 | Recombination protein recA | DNA replication, recombination, and repair | 3.12 | UP | 0 | 0 | 0.57 |
| *gyrA* | lmo0007 | DNA gyrase subunit A | DNA replication, recombination, and repair | 2.81 | 0 | 0 | 0 | 0.66 |
| *NA* | lmo1302 | highly similar to SOS response regulator lexA, transcription repressor protein | DNA replication, recombination, and repair | 2.59 | 0 | 0 | 0 | 0.75 |
| *recF* | LMOh7858_0006 | DNA replication and repair protein RecF | DNA replication, recombination, and repair | 2.46 | 0 | 0 | 0 | 0 |
| *tag* | LMOf2365_1661 | DNA-3-methyladenine glycosylase I | DNA replication, recombination, and repair | 2.22 | 0 | 0 | 0 | 0 |
| *mutM* | lmo1564 | highly similar to formamidopyrimidine-DNA glycosylases | DNA replication, recombination, and repair | 2.13 | 0 | 0 | 0 | 0.78 |
| *NA* | lmo2242 | similar to O6-methylguanine-DNA methyltransferase | DNA replication, recombination, and repair | 2.03 | UP | 0 | 0 | -0.32 |
| *dnaI* | lmo1560 | primosome component (helicase loader) DnaI | DNA replication, recombination, and repair | 2.01 | 0 | 0 | 0 | -0.6 |
| *RecF* | lmo0005 | RecF protein | DNA replication, recombination, and repair | 1.95 | 0 | 0 | 0 | -0.18 |
| *polA* | lmo1565 | DNA polymerase I | DNA replication, recombination, and repair | 1.92 | 0 | 0 | 0 | 1.8 |
| *dnaI* | LMOf2365_1582 | primosomal protein DnaI | DNA replication, recombination, and repair | 1.91 | 0 | 0 | 0 | 0 |
| *NA* | lmo0287 | similar to two-component response regulator | DNA replication, recombination, and repair | 1.89 | 0 | 2.49 | 0 | -0.01 |
| *gyrB* | LMOf2365_0006 | DNA gyrase, B subunit | DNA replication, recombination, and repair | 1.88 | 0 | 0 | 0 | 0 |
| *polC* | LMOf6854_1362 | DNA polymerase III, alpha subunit, Gram-positive type, frameshift | DNA replication, recombination, and repair | 1.77 | 0 | 0 | 0 | 0 |
| *NA* | lmo1825 | similar to pantothenate metabolism flavoprotein homolog | DNA replication, recombination, and repair | 1.52 | 0 | 0 | 0 | 0.4 |
| *recS* | lmo1942 | similar to ATP-dependent DNA helicase | DNA replication, recombination, and repair | 1.49 | 0 | 0 | 0 | 0.82 |
| *ruvB* | LMOf6854_1579 | holliday junction DNA helicase RuvB | DNA replication, recombination, and repair | 1.46 | 0 | 0 | 0 | 0 |
| *ssb* | LMOf6854_2682 | single-strand binding protein subfamily | DNA replication, recombination, and repair | -2.48 | 0 | 0 | 0 | 0 |
| *codV* | lmo1277 | similar to integrase/recombinase | DNA replication, recombination, and repair | -2.04 | 0 | 0 | 0 | -0.66 |
| *ssb* | lmo0045 | highly similar to single-strand binding protein (SSB) | DNA replication, recombination, and repair | -2.01 | 0 | 0 | 0 | 0.62 |
| *NA* | lmo1449 | similar to endonuclease IV | DNA replication, recombination, and repair | -1.92 | DOWN | 2.88 | 0 | -0.6 |
| *NA* | lmo1449 | similar to endonuclease IV | Degradation of DNA | -1.92 | DOWN | 2.88 | 0 | -0.6 |
| *NA* | lmo1722 | similar to ATP-dependent RNA helicases | DNA replication, recombination, and repair | -1.74 | 0 | 2.4 | 0 | 0.97 |
| *NA* | LMOf2365_0517 | MutT/nudix family protein | DNA replication, recombination, and repair | -1.73 | 0 | 0 | 0 | 0 |
| *NA* | LMOf2365_1910 | 5-3 exonuclease family protein | DNA replication, recombination, and repair | -1.68 | 0 | 0 | 0 | 0 |
| *NA* | LMOf2365_1910 | 5-3 exonuclease family protein | Degradation of DNA | -1.68 | 0 | 0 | 0 | 0 |
| *NA* | lmo1697 | similar to putative transmembrane proteins | DNA replication, recombination, and repair | -1.47 | DOWN | 0 | 0 | 0.68 |
| *NA* | lmo0233 | similar to DNA repair protein Sms | DNA replication, recombination, and repair | 0 | UP | 0 | 0 | 0.87 |
| *NA* | lmo0658 | conserved hypothetical protein | DNA replication, recombination, and repair | 0 | UP | 0 | 0 | -0.72 |
| *NA* | lmo1256 | unknown | DNA replication, recombination, and repair | 0 | UP | 0 | 0 | 1.36 |
| *recN* | lmo1368 | DNA repair and genetic recombination | DNA replication, recombination, and repair | 0 | UP | 0 | 0 | 0.55 |
| *NA* | lmo1412 | modulates DNA topology | DNA replication, recombination, and repair | 0 | UP | 0 | 0 | -0.78 |
| *NA* | lmo2222 | similar to unknown proteins | DNA replication, recombination, and repair | 0 | UP | -2.1 | 0 | -2.36 |
| *NA* | lmo2231 | similar to unknown proteins | DNA replication, recombination, and repair | 0 | UP | 0 | 2.6 | -1.03 |
| *NA* | lmo2267 | similar to ATP-dependent deoxyribonuclease (subunit A) | Degradation of DNA | 0 | UP | 0 | 0 | -2.26 |
| *int* | lmo2332 | putative integrase [Bacteriophage A118] | DNA replication, recombination, and repair | 0 | UP | -3.31 | 0 | -1.55 |
| *int* | lmo2332 | putative integrase [Bacteriophage A118] | Restriction/modification | 0 | UP | -3.31 | 0 | -1.55 |
| *NA* | lmo2368 | unknown | DNA replication, recombination, and repair | 0 | UP | 0 | 0 | -2.8 |
| *uvrA* | lmo2488 | excinuclease ABC (subunit A) | DNA replication, recombination, and repair | 0 | UP | 0 | 0 | 0.91 |
| *NA* | lmo1525 | similar to single-stranded-DNA-specific exonuclease (RecJ) | DNA replication, recombination, and repair | 0 | DOWN | 0 | 0 | -0.12 |
| *recU* | lmo1891 | similar to DNA repair and homologous recombination protein | DNA replication, recombination, and repair | 0 | 0 | 2.26 | 0 | -0.84 |
| *NA* | lmo2719 | conserved hypothetical proteins | DNA metabolism, Other | 0 | 0 | -2.39 | 0 | -0.91 |
| *NA* | lmo0996 | similar to methylated-DNA-protein-cystein methyltransferase | DNA replication, recombination, and repair | 0 | 0 | 0 | 2 | -1.26 |
| *dnaX* | lmo2704 | highly similar to DNA polymerase III (gamma and tau subunits) | DNA replication, recombination, and repair | 0 | 0 | 0 | -2 | -1.1 |
| *dnaA* | lmo0001 | Chromosomal replication initiation protein DnaA | DNA replication, recombination, and repair | 0 | 0 | 0 | 0 | 2.53 |
| *dnaN* | lmo0002 | DNA polymerase III, beta chain | DNA replication, recombination, and repair | 0 | 0 | 0 | 0 | 1.27 |
| *gyrB* | lmo0006 | DNA gyrase subunit B | DNA replication, recombination, and repair | 0 | 0 | 0 | 0 | -0.73 |
| *dnaC* | lmo0054 | highly similar to replicative DNA helicases | DNA replication, recombination, and repair | 0 | 0 | 0 | 0 | -4.57 |
| *NA* | lmo0157 | similar to ATP dependent helicase | DNA replication, recombination, and repair | 0 | 0 | 0 | 0 | -0.15 |
| *NA* | lmo0162 | similar to B. subtilis DNA polymerase III (delta subunit) | DNA replication, recombination, and repair | 0 | 0 | 0 | 0 | -0.62 |
| *mfd* | lmo0214 | transcription-repair coupling factor | DNA replication, recombination, and repair | 0 | 0 | 0 | 0 | -0.81 |
| *NA* | lmo0390 | similar to uracil-DNA glycosylase | DNA replication, recombination, and repair | 0 | 0 | 0 | 0 | 2.56 |
| *NA* | lmo0423 | similar to RNA polymerase ECF-type sigma factor | DNA replication, recombination, and repair | 0 | 0 | 0 | 0 | 2.04 |
| *NA* | lmo0466 | unknown | DNA replication, recombination, and repair | 0 | 0 | 0 | 0 | -0.63 |
| *NA* | lmo0470 | weakly similar to site-specific DNA-methyltransferase | Restriction/modification | 0 | 0 | 0 | 0 | -0.32 |
| *NA* | lmo0487 | unknown | DNA replication, recombination, and repair | 0 | 0 | 0 | 0 | 3.17 |
| *NA* | lmo0571 | similar to methyltransferase | DNA replication, recombination, and repair | 0 | 0 | 0 | 0 | 1.18 |
| *NA* | lmo0588 | similar to DNA photolyase | DNA replication, recombination, and repair | 0 | 0 | 0 | 0 | -0.23 |
| *NA* | lmo0763 | similar to unknown proteins | DNA replication, recombination, and repair | 0 | 0 | 0 | 0 | 0.75 |
| *NA* | lmo0846 | similar to excinuclease ABC, chain C (UvrC) | DNA replication, recombination, and repair | 0 | 0 | 0 | 0 | -0.43 |
| *NA* | lmo0928 | similar to 3-methyladenine DNA glycosylase | DNA replication, recombination, and repair | 0 | 0 | 0 | 0 | -0.14 |
| *NA* | lmo1119 | similar to methylases | DNA replication, recombination, and repair | 0 | 0 | 0 | 0 | -0.99 |
| *NA* | lmo1227 | similar to uracil-DNA glycosylase | DNA replication, recombination, and repair | 0 | 0 | 0 | 0 | 0.43 |
| *rnhC* | lmo1228 | similar to B. subtilis ribonuclease HIII | DNA replication, recombination, and repair | 0 | 0 | 0 | 0 | 0.16 |
| *NA* | lmo1231 | similar to DNA polymerase beta, to B. subtilis YshC protein | DNA replication, recombination, and repair | 0 | 0 | 0 | 0 | 0.48 |
| *uvrC* | lmo1234 | highly similar to excinuclease ABC subunit C | DNA replication, recombination, and repair | 0 | 0 | 0 | 0 | 1.7 |
| *NA* | lmo1248 | weakly similar to 8-oxo-dGTPase (mutT) | DNA replication, recombination, and repair | 0 | 0 | 0 | 0 | 0.08 |
| *topA* | lmo1275 | highly similar to DNA topoisomerase I TopA | DNA replication, recombination, and repair | 0 | 0 | 0 | 0 | 0.34 |
| *parE* | lmo1286 | highly similar to DNA gyrase-like protein (subunit B) | DNA replication, recombination, and repair | 0 | 0 | 0 | 0 | -0.22 |
| *parC* | lmo1287 | highly similar to DNA gyrase-like protein (subunit A) | DNA replication, recombination, and repair | 0 | 0 | 0 | 0 | -0.27 |
| *polC* | lmo1320 | highly similar to DNA polymerase III (alpha subunit) | DNA replication, recombination, and repair | 0 | 0 | 0 | 0 | 0.85 |
| *NA* | lmo1361 | similar to exodeoxyribonuclease VII (large subunit) | Degradation of DNA | 0 | 0 | 0 | 0 | 3.03 |
| *NA* | lmo1362 | similar to exodeoxyribonuclease small subunit | Degradation of DNA | 0 | 0 | 0 | 0 | 0.83 |
| *mutS* | lmo1403 | DNA mismatch repair (recognition) | DNA replication, recombination, and repair | 0 | 0 | 0 | 0 | 0.12 |
| *mutL* | lmo1404 | DNA mismatch repair protein | DNA replication, recombination, and repair | 0 | 0 | 0 | 0 | -1.83 |
| *dnaG* | lmo1455 | DNA primase | DNA replication, recombination, and repair | 0 | 0 | 0 | 0 | 1.05 |
| *NA* | lmo1460 | similar to B. subtilis RecO protein involved in DNA repair and homologous recombination | DNA replication, recombination, and repair | 0 | 0 | 0 | 0 | 1.83 |
| *comEB* | lmo1483 | similar to B. subtilis ComEB protein | DNA metabolism, Other | 0 | 0 | 0 | 0 | -2.18 |
| *NA* | lmo1509 | similar to exodeoxyribonuclease V | DNA replication, recombination, and repair | 0 | 0 | 0 | 0 | -0.58 |
| *ruvB* | lmo1532 | highly similar to Holliday junction DNA helicase RuvB | DNA replication, recombination, and repair | 0 | 0 | 0 | 0 | 2.32 |
| *ruvA* | lmo1533 | highly similar to Holliday junction DNA helicase (ruvA) | DNA replication, recombination, and repair | 0 | 0 | 0 | 0 | -0.12 |
| *dnaB* | lmo1561 | chromosome replication initiation / membrane attachment protein DnaB | DNA replication, recombination, and repair | 0 | 0 | 0 | 0 | 1.53 |
| *dnaE* | lmo1574 | highly similar to DNA polymerase III (alpha subunit) DnaE | DNA replication, recombination, and repair | 0 | 0 | 0 | 0 | -0.66 |
| *NA* | lmo1621 | weakly similar to E. coli MutT protein (dGTP pyrophosphohydrolase | DNA replication, recombination, and repair | 0 | 0 | 0 | 0 | 1.4 |
| *NA* | lmo1639 | similar to dna-3-methyladenine glycosidase | DNA replication, recombination, and repair | 0 | 0 | 0 | 0 | 2.86 |
| *NA* | lmo1644 | similar to SNF2-type helicase | DNA replication, recombination, and repair | 0 | 0 | 0 | 0 | -0.84 |
| *NA* | lmo1645 | similar to ATP-dependent dsDNA exonuclease SbcC | DNA replication, recombination, and repair | 0 | 0 | 0 | 0 | -0.3 |
| *NA* | lmo1645 | similar to ATP-dependent dsDNA exonuclease SbcC | Degradation of DNA | 0 | 0 | 0 | 0 | -0.3 |
| *NA* | lmo1646 | similar to putative exonucleases SbcD | DNA replication, recombination, and repair | 0 | 0 | 0 | 0 | 0.96 |
| *NA* | lmo1646 | similar to putative exonucleases SbcD | Degradation of DNA | 0 | 0 | 0 | 0 | 0.96 |
| *NA* | lmo1669 | some similarity to hypothetical proteins | DNA replication, recombination, and repair | 0 | 0 | 0 | 0 | 0.67 |
| *NA* | lmo1689 | similar to A/G-specific adenine glycosylase | DNA replication, recombination, and repair | 0 | 0 | 0 | 0 | 0.63 |
| *NA* | lmo1758 | similar to DNA ligase | DNA replication, recombination, and repair | 0 | 0 | 0 | 0 | 1.38 |
| *pcrA* | lmo1759 | ATP-dependent DNA helicase | DNA replication, recombination, and repair | 0 | 0 | 0 | 0 | 1.04 |
| *NA* | lmo1782 | similar to 3-exo-deoxyribonuclease exoA | DNA replication, recombination, and repair | 0 | 0 | 0 | 0 | -4.43 |
| *NA* | lmo1811 | similar to ATP-dependent DNA helicase recG | DNA replication, recombination, and repair | 0 | 0 | 0 | 0 | 0.22 |
| *priA* | lmo1824 | similar to primosomal replication factor Y | DNA replication, recombination, and repair | 0 | 0 | 0 | 0 | -1.37 |
| *NA* | lmo1872 | similar to methyltransferases | DNA replication, recombination, and repair | 0 | 0 | 0 | 0 | -1.52 |
| *NA* | lmo1872 | similar to methyltransferases | Restriction/modification | 0 | 0 | 0 | 0 | -1.52 |
| *NA* | lmo1881 | similar to 5-3 exonuclease | DNA replication, recombination, and repair | 0 | 0 | 0 | 0 | 0.22 |
| *nth* | lmo1894 | probable endonuclease III (DNA repair) | DNA replication, recombination, and repair | 0 | 0 | 0 | 0 | 0 |
| *dinG* | lmo1899 | similar to ATP-dependent helicases | DNA replication, recombination, and repair | 0 | 0 | 0 | 0 | 0.94 |
| *NA* | lmo2050 | similar to excinuclease ABC (subunit A) | DNA replication, recombination, and repair | 0 | 0 | 0 | 0 | 3.08 |
| *NA* | lmo2164 | similar to transcriptional regulator (AraC/XylS family) | DNA replication, recombination, and repair | 0 | 0 | 0 | 0 | -0.86 |
| *addB* | lmo2268 | similar to ATP-dependent deoxyribonuclease (subunit B) | Degradation of DNA | 0 | 0 | 0 | 0 | -2.72 |
| *NA* | lmo2308 | similar to single-stranded DNA-binding protein | DNA replication, recombination, and repair | 0 | 0 | 0 | 0 | 0.47 |
| *NA* | lmo2523 | similar to single-strand DNA-binding protein | DNA replication, recombination, and repair | 0 | 0 | 0 | 0 | 2.01 |
| *recR* | lmo2702 | highly similar to recombination protein recR | DNA replication, recombination, and repair | 0 | 0 | 0 | 0 | 0.67 |
| *topB* | lmo2756 | similar to DNA topoisomerase III | DNA replication, recombination, and repair | 0 | 0 | 0 | 0 | 0.2 |
| *NA* | lmo2757 | similar to ATP-dependent DNA helicases | DNA replication, recombination, and repair | 0 | 0 | 0 | 0 | 0.42 |
| **Transcription** | |  |  |  |  |  |  |  |
| *NA* | lmo2449 | similar to exoribonuclease RNase-R | Degradation of RNA | 2.32 | UP | 0 | 0 | 0.94 |
| *NA* | lmo2449 | similar to exoribonuclease RNase-R | RNA processing | 2.32 | UP | 0 | 0 | 0.94 |
| *rnhB* | lmo1273 | similar to ribonuclease H rnh | Degradation of RNA | 1.84 | 0 | 0 | 0 | 1.64 |
| *sigB* | lmo0895 | RNA polymerase sigma-37 factor (sigma-B) | Transcription factors | 1.57 | 0 | 0 | 0 | -2.07 |
| *NA* | lmo1793 | similar to putative 16S rRNA processing protein RimM | RNA processing | 1.54 | 0 | 0 | 0 | 0.5 |
| *rpoD* | lmo1454 | RNA polymerase sigma factor RpoD | Transcription factors | 1.5 | UP | 0 | 1.1 | 0.36 |
| *pnpA* | lmo1331 | polynucleotide phosphorylase (PNPase) | Degradation of RNA | 1.38 | 0 | 0 | 0 | 2.1 |
| *NA* | LMOf6854_1900 | pyrimidine operon regulatory protein PyrR | Transcription factors | -5.7 | 0 | 0 | 0 | 0 |
| *rncS* | lmo1805 | similar to ribonuclease III | RNA processing | -2.44 | 0 | 0 | 0 | 1.99 |
| *NA* | lmo1496 | similar to transcription elongation factor GreA | Transcription factors | -1.75 | 0 | 0 | 0 | 1.12 |
| *NA* | lmo1722 | similar to ATP-dependent RNA helicases | Other | -1.74 | 0 | 2.4 | 0 | 0.97 |
| *NA* | lmo1450 | similar to ATP-dependent RNA helicase, DEAD-box family (deaD) | Transcription factors | -1.58 | 0 | 2.18 | 0 | -0.16 |
| *NA* | lmo1450 | similar to ATP-dependent RNA helicase, DEAD-box family (deaD) | RNA processing | -1.58 | 0 | 2.18 | 0 | -0.16 |
| *NA* | lmo1450 | similar to ATP-dependent RNA helicase, DEAD-box family (deaD) | Other | -1.58 | 0 | 2.18 | 0 | -0.16 |
| *NA* | LMOf6854_1591 | ribonuclease, Rne/Rng family | Degradation of RNA | -1.48 | 0 | 0 | 0 | 0 |
| *rho* | lmo2551 | highly similar to transcription terminator factor rho | Transcription factors | -1.45 | 0 | 0 | 0 | 0.94 |
| *rnpA* | lmo2855 | ribonuclease P protein component | RNA processing | -1.43 | 0 | 0 | 0 | 0 |
| *rpoA* | lmo2606 | highly similar to RNA polymerase (alpha subunit) | DNA-dependent RNA polymerase | -1.41 | 0 | 0 | 0 | -1.7 |
| *NA* | lmo1246 | similar to ATP-dependent RNA helicase (DEAD motif) | Transcription factors | -1.33 | 0 | 0 | 0 | 0.01 |
| *NA* | lmo2275 | Portein gp28 [Bacteriophage A118] | Transcription factors | 0 | UP | 0 | 0 | -0.94 |
| *sigL* | lmo2461 | RNA polymerase sigma-54 factor (sigma-L) | Transcription factors | 0 | UP | 0 | 0 | 1.45 |
| *NA* | lmo2560 | similar to B. subtilis RNA polymerase delta subunit | DNA-dependent RNA polymerase | 0 | DOWN | 0 | 0 | 1.45 |
| *NA* | lmo0866 | similar to ATP-dependent RNA helicase | Transcription factors | 0 | 0 | 2.36 | 0 | 0.79 |
| *NA* | lmo0866 | similar to ATP-dependent RNA helicase | RNA processing | 0 | 0 | 2.36 | 0 | 0.79 |
| *NA* | lmo0866 | similar to ATP-dependent RNA helicase | Other | 0 | 0 | 2.36 | 0 | 0.79 |
| *rsbW* | lmo0894 | sigma-B activity negative regulator RsbW | Transcription factors | 0 | 0 | 0 | 2.4 | -1.06 |
| *NA* | lmo0218 | polyribonucleotide nucleotidyltransferase domain present | Degradation of RNA | 0 | 0 | 0 | 0 | -2.85 |
| *sigH* | lmo0243 | RNA polymerase sigma-30 factor (sigma-H) | Transcription factors | 0 | 0 | 0 | 0 | -2.55 |
| *nusG* | lmo0246 | transcription antitermination factor | Transcription factors | 0 | 0 | 0 | 0 | -0.02 |
| *rpoB* | lmo0258 | RNA polymerase (beta subunit) | DNA-dependent RNA polymerase | 0 | 0 | 0 | 0 | -0.55 |
| *rpoC* | lmo0259 | RNA polymerase (beta subunit) | DNA-dependent RNA polymerase | 0 | 0 | 0 | 0 | -0.13 |
| *NA* | lmo0423 | similar to RNA polymerase ECF-type sigma factor | DNA-dependent RNA polymerase | 0 | 0 | 0 | 0 | 2.04 |
| *NA* | lmo0423 | similar to RNA polymerase ECF-type sigma factor | Other | 0 | 0 | 0 | 0 | 2.04 |
| *NA* | lmo0844 | conserved hypothetical protein | Degradation of RNA | 0 | 0 | 0 | 0 | 0.65 |
| *rsbS* | lmo0890 | highly similar to negative regulation of sigma-B activity | Transcription factors | 0 | 0 | 0 | 0 | -0.03 |
| *rsbT* | lmo0891 | highly similar to positive regulation of sigma-B activity | Transcription factors | 0 | 0 | 0 | 0 | -0.72 |
| *rsbU* | lmo0892 | highly similar to serine phosphatase RsbU | Transcription factors | 0 | 0 | 0 | 0 | 1.9 |
| *nusA* | lmo1322 | highly similar to N utilization substance protein A (NusA protein) | Transcription factors | 0 | 0 | 0 | 0 | 1.2 |
| *rbfA* | lmo1327 | highly similar to ribosome-binding factor A | RNA processing | 0 | 0 | 0 | 0 | 1.04 |
| *NA* | lmo1359 | similar to transcription termination protein (NusB) | Transcription factors | 0 | 0 | 0 | 0 | -0.51 |
| *NA* | lmo1644 | similar to SNF2-type helicase | Other | 0 | 0 | 0 | 0 | -0.84 |
| *NA* | lmo1706 | similar to transport proteins | RNA processing | 0 | 0 | 0 | 0 | -1.87 |
| *NA* | lmo1880 | similar to similar to RNase HI | Degradation of RNA | 0 | 0 | 0 | 0 | 0.09 |
| *cca* | lmo1905 | similar to tRNA CCA-adding enzyme | RNA processing | 0 | 0 | 0 | 0 | -6.09 |
| *phoP* | lmo2501 | two-component response phosphate regulator | Other | 0 | 0 | 0 | 0 | -1.67 |
| **Protein synthesis** | |  |  |  |  |  |  |  |
| *NA* | lmo0695 | unknown | tRNA aminoacylation | 2.31 | 0 | 0 | -2.1 | 1.68 |
| *ctc* | lmo0211 | similar to B. subtilis general stress protein | Ribosomal proteins: synthesis and modification | 2.22 | UP | 3.9 | 1.4 | -3.78 |
| *NA* | lmo2448 | conserved hypothetical protein | Other | 2 | UP | 0 | 0 | 0.64 |
| *proS* | lmo1319 | prolyl-tRNA synthetase | tRNA aminoacylation | 1.89 | 0 | 0 | 0 | 2.22 |
| *NA* | lmo2555 | weakly similar to human N-acetylglucosaminyl-phosphatidylinositol biosynthetic protein | tRNA aminoacylation | 1.89 | 0 | 0 | 0 | 0.16 |
| *fmt* | lmo1823 | similar to methionyl-tRNA formyltransferase | tRNA aminoacylation | 1.72 | 0 | 0 | 0 | 2.24 |
| *trmD* | lmo1792 | similar to E. coli tRNA (guanine-N1) methyltransferase | tRNA and rRNA base modification | 1.67 | 0 | 0 | 0 | 1.97 |
| *NA* | LMOf6854_1809 | RNA methyltransferase, TrmA family | tRNA and rRNA base modification | 1.64 | 0 | 0 | 0 | 0 |
| *efp* | lmo1355 | highly similar to elongation factor P (EF-P) | Translation factors | 1.27 | 0 | 0 | 0 | 1.94 |
| *hisS* | LMOf6854_1567 | histidyl-tRNA synthetase | tRNA aminoacylation | -4.2 | 0 | 0 | 0 | 0 |
| *aspS* | lmo1519 | aspartyl-tRNA synthetase | tRNA aminoacylation | -3.52 | 0 | 0 | 0 | 1.55 |
| *rplC* | lmo2632 | ribosomal protein L3 | Ribosomal proteins: synthesis and modification | -3.44 | DOWN | 0 | -2 | 0.96 |
| *valS* | lmo1552 | valyl-tRNA synthetase | tRNA aminoacylation | -3.43 | DOWN | 0 | 0 | 0.43 |
| *rplJ* | lmo0250 | ribosomal protein L10 | Ribosomal proteins: synthesis and modification | -3.36 | DOWN | 0 | 0 | -0.42 |
| *rpmG-2* | LMOf2365_1352 | ribosomal protein L33 | Ribosomal proteins: synthesis and modification | -3.3 | 0 | 0 | 0 | 0 |
| *rplD* | lmo2631 | ribosomal protein L4 | Ribosomal proteins: synthesis and modification | -3.26 | DOWN | 0 | 0 | -1.96 |
| *rpsG* | lmo2655 | ribosomal protein S7 | Ribosomal proteins: synthesis and modification | -2.95 | 0 | 0 | 0 | 0.25 |
| *NA* | LMOh7858_0268 | RNA methyltransferase, TrmH family, group 3 | tRNA and rRNA base modification | -2.93 | 0 | 0 | 0 | 0 |
| *rplW* | lmo2630 | ribosomal protein L23 | Ribosomal proteins: synthesis and modification | -2.81 | 0 | 0 | -2.2 | 0.9 |
| *rpmD* | lmo2614 | ribosomal protein L30 | Ribosomal proteins: synthesis and modification | -2.69 | 0 | 0 | -2 | 1.44 |
| *rplL* | lmo0251 | ribosomal protein L12 | Ribosomal proteins: synthesis and modification | -2.64 | DOWN | 0 | 0 | -0.51 |
| *rpsP* | lmo1797 | ribosomal protein S16 | Ribosomal proteins: synthesis and modification | -2.58 | DOWN | 0 | -3.9 | 0.3 |
| *NA* | lmo2550 | similar to glycosyl transferases | tRNA aminoacylation | -2.44 | 0 | 0 | 0 | -0.35 |
| *leuS* | lmo1660 | leucyl-tRNA synthetase | tRNA aminoacylation | -2.43 | 0 | 0 | 0 | 1.48 |
| *rpmF* | lmo0486 | ribosomal protein L32 | Ribosomal proteins: synthesis and modification | -2.43 | 0 | 3.33 | -2.8 | 3.48 |
| *rpmF* | lmo2047 | ribosomal protein L32 | Ribosomal proteins: synthesis and modification | -2.39 | DOWN | 0 | -2.7 | -0.81 |
| *rpsR* | lmo0046 | ribosomal protein S18 | Ribosomal proteins: synthesis and modification | -2.35 | 0 | 0 | 0 | -1.44 |
| *ileS* | LMOf6854_2081 | isoleucyl-tRNA synthetase | tRNA aminoacylation | -2.28 | 0 | 0 | 0 | 0 |
| *rplF* | lmo2617 | ribosomal protein L6 | Ribosomal proteins: synthesis and modification | -2.27 | DOWN | 0 | 0 | 1.95 |
| *rpsF* | lmo0044 | ribosomal protein S6 | Ribosomal proteins: synthesis and modification | -2.21 | DOWN | 0 | 0 | -0.74 |
| *lysS* | lmo0228 | lysyl-tRNA synthetase | tRNA aminoacylation | -2.19 | DOWN | 0 | 0 | 0.22 |
| *rplO* | lmo2613 | ribosomal protein L15 | Ribosomal proteins: synthesis and modification | -2.1 | DOWN | 0 | 0 | 1.16 |
| *trpS* | lmo2198 | tryptophanyl-tRNA synthetase | tRNA aminoacylation | -2.05 | DOWN | 0 | 0 | -1.31 |
| *rpmC* | lmo2624 | ribosomal protein L29 | Ribosomal proteins: synthesis and modification | -2.03 | 0 | 0 | -3.3 | 1.38 |
| *NA* | lmo0511 | conserved hypothetical protein | tRNA aminoacylation | -2 | 0 | 0 | 0 | 0.52 |
| *rplR* | lmo2616 | ribosomal protein L18 | Ribosomal proteins: synthesis and modification | -1.98 | 0 | 0 | 0 | 1.62 |
| *thrS* | lmo1559 | threonyl-tRNA synthetase | tRNA aminoacylation | -1.97 | DOWN | 0 | 0 | -0.28 |
| *miaA* | lmo1294 | similar to tRNA isopentenylpyrophosphate transferase | tRNA and rRNA base modification | -1.93 | 0 | 0 | -2.4 | -0.84 |
| *NA* | lmo1938 | similar to similar to ribosomal protein S1 like protein | Ribosomal proteins: synthesis and modification | -1.92 | DOWN | 0 | 0 | 1.12 |
| *rpsB* | lmo1658 | 30S ribosomal protein S2 | Ribosomal proteins: synthesis and modification | -1.9 | 0 | 0 | 0 | 0.1 |
| *NA* | lmo1530 | similar to tRNA-guanine transglycosylase Tgt | tRNA and rRNA base modification | -1.83 | DOWN | 0 | 0 | 0.13 |
| *frr* | lmo1314 | highly similar to ribosome recycling factors | Translation factors | -1.82 | 0 | 0 | 0 | -0.81 |
| *rplU* | lmo1542 | ribosomal protein L21 | Ribosomal proteins: synthesis and modification | -1.8 | DOWN | 0 | 0 | 2.1 |
| *thrS* | LMOf2365_1580 | threonyl-tRNA synthetase | tRNA aminoacylation | -1.78 | 0 | 0 | 0 | 0 |
| *aspS* | LMOf2365_1538 | aspartyl-tRNA synthetase | tRNA aminoacylation | -1.77 | 0 | 0 | 0 | 0 |
| *rpmA* | lmo1540 | ribosomal protein L27 | Ribosomal proteins: synthesis and modification | -1.76 | DOWN | 0 | 0 | -1.13 |
| *pheS* | lmo1221 | phenylalany-tRNA synthetase alpha subunit | tRNA aminoacylation | -1.76 | 0 | -2.13 | 0 | -0.29 |
| *NA* | LMOh7858_0267 | B. subtilis YazC protein homolog lmo0240 [imported] | Ribosomal proteins: synthesis and modification | -1.74 | 0 | 0 | 0 | 0 |
| *NA* | lmo1722 | similar to ATP-dependent RNA helicases | Translation factors | -1.74 | 0 | 2.4 | 0 | 0.97 |
| *prf1* | LMOf6854_2604 | peptide chain release factor 1 | Translation factors | -1.74 | 0 | 0 | 0 | 0 |
| *infA* | lmo2610 | highly similar to initiation factor IF-I | Translation factors | -1.71 | 0 | 0 | 0 | 1.15 |
| *rpmJ* | lmo2609 | ribosomal protein L36 | Ribosomal proteins: synthesis and modification | -1.7 | 0 | 0 | 0 | 0.24 |
| *rpsK* | lmo2607 | ribosomal protein S11 | Ribosomal proteins: synthesis and modification | -1.67 | 0 | 0 | 0 | -0.12 |
| *metS* | lmo0177 | methionyl-tRNA synthetase | tRNA aminoacylation | -1.64 | DOWN | 0 | 0 | -1.3 |
| *rpsM* | lmo2608 | ribosomal protein S13 | Ribosomal proteins: synthesis and modification | -1.63 | 0 | 0 | 0 | 1.28 |
| *hisS* | lmo1520 | histidyl-tRNA synthetase | tRNA aminoacylation | -1.61 | DOWN | 0 | 0 | -0.27 |
| *NA* | lmo0244 | similar to ribosomal protein L33 type II | Ribosomal proteins: synthesis and modification | -1.54 | 0 | 0 | 0 | -2.41 |
| *infC* | LMOf6854_1844 | translation initiation factor IF-3 | Translation factors | -1.48 | 0 | 0 | 0 | 0 |
| *rplK* | lmo0248 | ribosomal protein L11 | Ribosomal proteins: synthesis and modification | -1.47 | DOWN | 0 | 0 | -1.08 |
| *NA* | LMOh7858_0250 | dihydrouridine synthase family protein | tRNA and rRNA base modification | -1.46 | 0 | 0 | 0 | 0 |
| *NA* | lmo1882 | similar to ribosomal protein S14 | Ribosomal proteins: synthesis and modification | -1.42 | 0 | 0 | 0 | 0.63 |
| *NA* | lmo2811 | similar to GTPase | tRNA and rRNA base modification | -1.37 | 0 | 0 | 0 | -0.71 |
| *argS* | lmo2561 | arginyl tRNA synthetase | tRNA aminoacylation | -1.36 | 0 | 0 | 0 | -0.18 |
| *rpsN-1* | LMOf2365_1911 | ribosomal protein S14 | Ribosomal proteins: synthesis and modification | -1.36 | 0 | 0 | 0 | 0 |
| *NA* | lmo1324 | conserved hypothetical protein, similar to B. subtilis YlxQ protein | Ribosomal proteins: synthesis and modification | -1.22 | 0 | 0 | 0 | 1.01 |
| *NA* | lmo2027 | putative cell surface protein, similar to internalin proteins | tRNA aminoacylation | 0 | UP | 0 | 0 | 0.35 |
| *NA* | lmo2511 | similar to conserved hypothetical proteins like to B. subtilis YvyD protein | Ribosomal proteins: synthesis and modification | 0 | UP | 0 | 1.3 | 0.58 |
| *ksgA* | lmo0188 | dimethyladenosine transferase (16S rRNA dimethylase) | tRNA and rRNA base modification | 0 | DOWN | 0 | 0 | -1.25 |
| *rplA* | lmo0249 | ribosomal protein L1 | Ribosomal proteins: synthesis and modification | 0 | DOWN | 0 | 0 | -0.2 |
| *glyS* | lmo1458 | similar to glycyl-tRNA synthetase beta chain | tRNA aminoacylation | 0 | DOWN | -2.21 | 0 | 1.17 |
| *glyQ* | lmo1459 | similar to glycyl-tRNA synthetase alpha chain | tRNA aminoacylation | 0 | DOWN | 0 | 0 | 0.66 |
| *rpsU* | lmo1469 | 30S ribosomal protein S21 | Ribosomal proteins: synthesis and modification | 0 | DOWN | 0 | -1.2 | 0.89 |
| *rpsT* | lmo1480 | ribosomal protein S20 | Ribosomal proteins: synthesis and modification | 0 | DOWN | 0 | -2.8 | -3.98 |
| *alaS* | lmo1504 | alanyl-tRNA synthetase | tRNA aminoacylation | 0 | DOWN | -2.37 | 0 | -0.15 |
| *rpsD* | lmo1596 | ribosomal protein S4 | Ribosomal proteins: synthesis and modification | 0 | DOWN | 0 | 0 | -1.73 |
| *tyrS* | lmo1598 | tyrosyl-tRNA synthetase | tRNA aminoacylation | 0 | DOWN | -2.27 | 0 | 0.82 |
| *infC* | lmo1785 | translation initiation factor IF-3 | Translation factors | 0 | DOWN | 0 | 0 | 3.21 |
| *rplS* | lmo1787 | ribosomal protein L19 | Ribosomal proteins: synthesis and modification | 0 | DOWN | 0 | 0 | 0.81 |
| *rpmE* | lmo2548 | ribosomal protein L31 | Ribosomal proteins: synthesis and modification | 0 | DOWN | 0 | 0 | 0.44 |
| *rpsI* | lmo2596 | ribosomal protein S9 | Ribosomal proteins: synthesis and modification | 0 | DOWN | 0 | 0 | 1.09 |
| *rplM* | lmo2597 | ribosomal protein L13 | Ribosomal proteins: synthesis and modification | 0 | DOWN | 0 | 1.5 | 0.21 |
| *rpsE* | lmo2615 | ribosomal protein S5 | Ribosomal proteins: synthesis and modification | 0 | DOWN | 0 | 0 | 1.18 |
| *rpsH* | lmo2618 | ribosomal protein S8 | Ribosomal proteins: synthesis and modification | 0 | DOWN | 0 | -2.5 | 2.07 |
| *rpsN* | lmo2619 | ribosomal protein S14 | Ribosomal proteins: synthesis and modification | 0 | DOWN | 0 | 0 | -1.21 |
| *rplX* | lmo2621 | ribosomal protein L24 | Ribosomal proteins: synthesis and modification | 0 | DOWN | 0 | -2.3 | 2.61 |
| *rplN* | lmo2622 | ribosomal protein L14 | Ribosomal proteins: synthesis and modification | 0 | DOWN | 0 | -2.3 | 2.27 |
| *rplP* | lmo2625 | ribosomal protein L16 | Ribosomal proteins: synthesis and modification | 0 | DOWN | 0 | 0 | 0.03 |
| *rpsC* | lmo2626 | ribosomal protein S3 | Ribosomal proteins: synthesis and modification | 0 | DOWN | 0 | 0 | -3.02 |
| *rplV* | lmo2627 | ribosomal protein L22 | Ribosomal proteins: synthesis and modification | 0 | DOWN | 0 | -2.1 | 2 |
| *rpsS* | lmo2628 | ribosomal protein S19 | Ribosomal proteins: synthesis and modification | 0 | DOWN | 0 | -3.3 | 1.08 |
| *rplB* | lmo2629 | ribosomal protein L2 | Ribosomal proteins: synthesis and modification | 0 | DOWN | 0 | 0 | 2.33 |
| *rpsJ* | lmo2633 | ribosomal protein S10 | Ribosomal proteins: synthesis and modification | 0 | DOWN | 0 | 0 | 0.25 |
| *NA* | lmo0988 | similar to peptide chain release factor 3 (RF-3) | Translation factors | 0 | 0 | 2.19 | 0 | -1.26 |
| *pheT* | lmo1222 | phenylalanyl-tRNA synthetase beta subunit | tRNA aminoacylation | 0 | 0 | -2.31 | 0 | -0.36 |
| *ansB* | lmo1896 | similar to asparaginyl-tRNA synthetases | tRNA aminoacylation | 0 | 0 | -2.55 | 0 | 0.78 |
| *NA* | lmo1698 | similar to ribosomal-protein-alanine N-acetyltransferase | Ribosomal proteins: synthesis and modification | 0 | 0 | 0 | 1.5 | 0.73 |
| *rpsO* | lmo1330 | ribosomal protein S15 | Ribosomal proteins: synthesis and modification | 0 | 0 | 0 | -2 | -0.06 |
| *rplT* | lmo1783 | ribosomal protein L20 | Ribosomal proteins: synthesis and modification | 0 | 0 | 0 | -2.4 | 3.38 |
| *rpmI* | lmo1784 | ribosomal protein L35 | Ribosomal proteins: synthesis and modification | 0 | 0 | 0 | -2 | 1.11 |
| *rplE* | lmo2620 | ribosomal protein L5 | Ribosomal proteins: synthesis and modification | 0 | 0 | 0 | -2.9 | 1.01 |
| *rpsQ* | lmo2623 | ribosomal protein S17 | Ribosomal proteins: synthesis and modification | 0 | 0 | 0 | -2.2 | 2.39 |
| *rpmH* | lmo2856 | ribosomal protein L34 | Ribosomal proteins: synthesis and modification | 0 | 0 | 0 | -2.7 | 0 |
| *rplI* | lmo0053 | 50S ribosomal protein L9 | Ribosomal proteins: synthesis and modification | 0 | 0 | 0 | 0 | -2.19 |
| *pth* | lmo0213 | similar to peptidyl-tRNA hydrolase | Other | 0 | 0 | 0 | 0 | -1.34 |
| *gltX* | lmo0237 | highly similar to glutamyl-tRNA synthetase | tRNA aminoacylation | 0 | 0 | 0 | 0 | 4.57 |
| *cysS* | lmo0239 | cysteinyl-tRNA synthetase | tRNA aminoacylation | 0 | 0 | 0 | 0 | 2.59 |
| *NA* | lmo0240 | highly similar to B. subtilis YazC protein | tRNA aminoacylation | 0 | 0 | 0 | 0 | 1.78 |
| *NA* | lmo0241 | similar to conserved hypothetical proteins like to B. subtilis YacO protein | tRNA and rRNA base modification | 0 | 0 | 0 | 0 | 0.42 |
| *NA* | lmo0327 | similar to cell surface proteins (LPXTG motif) | tRNA aminoacylation | 0 | 0 | 0 | 0 | -1.43 |
| *NA* | lmo0549 | similar to internalin protein | tRNA aminoacylation | 0 | 0 | 0 | 0 | 2.7 |
| *hisZ* | lmo0569 | histidyl-tRNA synthetase | tRNA aminoacylation | 0 | 0 | 0 | 0 | 0.85 |
| *NA* | lmo0716 | similar to H+-transporting ATP synthase alpha chain FliI, flagellar-specific, - | tRNA aminoacylation | 0 | 0 | 0 | 0 | 0.02 |
| *NA* | lmo0933 | similar to sugar transferase | tRNA aminoacylation | 0 | 0 | 0 | 0 | -1.64 |
| *NA* | lmo0935 | similar to B. subtilis CspR protein, rRNA methylase homolog | tRNA and rRNA base modification | 0 | 0 | 0 | 0 | -1.56 |
| *NA* | lmo0969 | similar to ribosomal large subunit pseudouridine synthetase | tRNA and rRNA base modification | 0 | 0 | 0 | 0 | -1.73 |
| *NA* | lmo1218 | similar to rRNA methylase | tRNA and rRNA base modification | 0 | 0 | 0 | 0 | -0.18 |
| *NA* | lmo1289 | similar to internalin proteins, putative peptidoglycan bound protein (LPXTG motif) | tRNA aminoacylation | 0 | 0 | 0 | 0 | 0.56 |
| *infB* | lmo1325 | highly similar to translation initiation factor IF-2 | Translation factors | 0 | 0 | 0 | 0 | 1 |
| *truB* | lmo1328 | highly similar to tRNA pseudouridine 55 synthase | tRNA and rRNA base modification | 0 | 0 | 0 | 0 | 2.23 |
| *NA* | lmo1471 | similar to ribosomal protein L11 methyltransferase | Ribosomal proteins: synthesis and modification | 0 | 0 | 0 | 0 | 0.55 |
| *NA* | lmo1512 | similar to putative tRNA (5-methylaminomethyl-2-thiouridylate)-methyltransferase | tRNA and rRNA base modification | 0 | 0 | 0 | 0 | -0.19 |
| *NA* | lmo1531 | similar to S-adenosylmethionine:tRNA ribosyltransferase-isomerase | tRNA and rRNA base modification | 0 | 0 | 0 | 0 | 0.65 |
| *NA* | lmo1644 | similar to SNF2-type helicase | Translation factors | 0 | 0 | 0 | 0 | -0.84 |
| *tsf* | lmo1657 | translation elongation factor | Translation factors | 0 | 0 | 0 | 0 | -0.16 |
| *NA* | lmo1703 | similar to similar to RNA methyltransferases | tRNA and rRNA base modification | 0 | 0 | 0 | 0 | -1.26 |
| *NA* | lmo1751 | similar to hypothetical RNA methyltransferase | tRNA and rRNA base modification | 0 | 0 | 0 | 0 | 0.88 |
| *gatB* | lmo1754 | glutamyl-tRNA(Gln) amidotransferase (subunit B) | tRNA aminoacylation | 0 | 0 | 0 | 0 | 0.11 |
| *gatA* | lmo1755 | glutamyl-tRNA(Gln) amidotransferase (subunit A) | tRNA aminoacylation | 0 | 0 | 0 | 0 | 0.17 |
| *gatC* | lmo1756 | glutamyl-tRNA(Gln) amidotransferase (subunit C) | tRNA aminoacylation | 0 | 0 | 0 | 0 | 0.35 |
| *NA* | lmo1795 | similar to unknown proteins | tRNA aminoacylation | 0 | 0 | 0 | 0 | -0.8 |
| *rpmB* | lmo1816 | ribosomal protein L28 | Ribosomal proteins: synthesis and modification | 0 | 0 | 0 | 0 | 0.97 |
| *NA* | lmo1822 | similar to RNA-binding Sun protein | tRNA and rRNA base modification | 0 | 0 | 0 | 0 | -0.79 |
| *NA* | lmo1843 | similar to conserved hypothetical proteins | tRNA and rRNA base modification | 0 | 0 | 0 | 0 | 1.2 |
| *NA* | lmo1872 | similar to methyltransferases | tRNA and rRNA base modification | 0 | 0 | 0 | 0 | -1.52 |
| *NA* | lmo1949 | similar to unknown proteins | tRNA and rRNA base modification | 0 | 0 | 0 | 0 | -1.04 |
| *ileS* | lmo2019 | isoleucyl-tRNA synthetase | tRNA aminoacylation | 0 | 0 | 0 | 0 | -5.67 |
| *NA* | lmo2026 | putative peptidoglycan bound protein (LPXTG motif) | tRNA aminoacylation | 0 | 0 | 0 | 0 | -0.65 |
| *NA* | lmo2076 | similar to ribosomal protein alanine acetyltransferase | Ribosomal proteins: synthesis and modification | 0 | 0 | 0 | 0 | 0.2 |
| *NA* | lmo2121 | similar to maltosephosphorylase | tRNA aminoacylation | 0 | 0 | 0 | 0 | -1.48 |
| *NA* | lmo2244 | similar to putative ribosomal large subunit pseudouridine synthase | tRNA and rRNA base modification | 0 | 0 | 0 | 0 | -1.13 |
| *NA* | lmo2342 | similar to 16S pseudouridylate synthase | tRNA and rRNA base modification | 0 | 0 | 0 | 0 | -0.93 |
| *NA* | lmo2396 | similar to internalin proteins, putative peptidoglycan bound protein (LPXTG motif) | tRNA aminoacylation | 0 | 0 | 0 | 0 | -0.83 |
| *NA* | lmo2445 | similar to internalin | tRNA aminoacylation | 0 | 0 | 0 | 0 | 0.94 |
| *prfB* | lmo2509 | highly similar to peptide chain release factor 2 | Translation factors | 0 | 0 | 0 | 0 | -0.68 |
| *prf1* | lmo2543 | highly similar to peptide chain release factor 1 | Translation factors | 0 | 0 | 0 | 0 | 2.23 |
| *truA* | lmo2598 | highly similar to pseudouridylate synthase I | tRNA and rRNA base modification | 0 | 0 | 0 | 0 | 1.36 |
| *rplQ* | lmo2605 | ribosomal protein L17 | Ribosomal proteins: synthesis and modification | 0 | 0 | 0 | 0 | 1.55 |
| *tufA* | lmo2653 | highly similar to translation elongation factor EF-Tu | Translation factors | 0 | 0 | 0 | 0 | -0.05 |
| *fus* | lmo2654 | highly similar to translation elongation factor G | Translation factors | 0 | 0 | 0 | 0 | -0.32 |
| *rpsL* | lmo2656 | ribosomal protein S12 | Ribosomal proteins: synthesis and modification | 0 | 0 | 0 | 0 | 0.9 |
| *serS* | lmo2747 | seryl-trna synthetase | tRNA aminoacylation | 0 | 0 | 0 | 0 | -0.1 |
| *NA* | lmo2821 | similar to internalin, Unknown, putative peptidoglycan bound protein (LPXTG motif) | tRNA aminoacylation | 0 | 0 | 0 | 0 | 1.31 |
| *NA* | lmo2833 | similar to a maltose phosphorylase | tRNA aminoacylation | 0 | 0 | 0 | 0 | -0.89 |
|  | Protein fate |  |  |  |  |  |  |  |
| *NA* | lmo2199 | similar to unknown protein | Protein and peptide secretion and trafficking | 2.77 | UP | 2.89 | 0 | -0.45 |
| *sepA* | lmo2157 | sepA | Protein and peptide secretion and trafficking | 2.73 | UP | 0 | 13.9 | 2.23 |
| *secA* | lmo2510 | translocase binding subunit (ATPase) | Protein and peptide secretion and trafficking | 2.59 | 0 | 0 | 0 | -0.29 |
| *NA* | lmo0292 | similar to heat-shock protein htrA serine protease | Degradation of proteins, peptides, and glycopeptides | 2.31 | 0 | 0 | 0 | 0.65 |
| *NA* | lmo0655 | similar to phosphoprotein phosphatases | Protein modification and repair | 2.26 | UP | 0 | 2.5 | -1.63 |
| *NA* | LMOf2365_2289 | intracellular protease, PfpI family | Degradation of proteins, peptides, and glycopeptides | 2.15 | 0 | 0 | 0 | 0 |
| *NA* | lmo0215 | conserved membrane-spanning protein | Protein and peptide secretion and trafficking | 2.04 | 0 | 0 | 0 | 0.77 |
| *NA* | lmo0215 | conserved membrane-spanning protein | Degradation of proteins, peptides, and glycopeptides | 2.04 | 0 | 0 | 0 | 0.77 |
| *NA* | lmo2515 | similar to B. subtilis two-component response regulator DegU | Degradation of proteins, peptides, and glycopeptides | 1.94 | 0 | 0 | 0 | -3.28 |
| *sugE-2* | LMOf2365_0871 | sugE protein | Protein folding and stabilization | 1.84 | 0 | 0 | 0 | 0 |
| *NA* | lmo1821 | similar to putative phosphoprotein phosphatase | Protein modification and repair | 1.84 | 0 | 0 | 0 | 0.14 |
| *NA* | lmo0853 | similar to E. coli SugE protein (transmembrane chaperone) | Protein folding and stabilization | 1.83 | 0 | 0 | 0 | -0.34 |
| *NA* | lmo0854 | similar to E. coli SugE protein (transmembrane chaperone) | Protein folding and stabilization | 1.82 | 0 | 0 | 0 | -0.61 |
| *dnaJ* | LMOf2365_1491 | chaperone protein DnaJ | Protein folding and stabilization | 1.8 | 0 | 0 | 0 | 0 |
| *NA* | lmo1393 | similar to putative protease | Degradation of proteins, peptides, and glycopeptides | 1.78 | 0 | 0 | 0 | -3.56 |
| *NA* | lmo0583 | similar to preprotein translocase SecA subunit | Protein and peptide secretion and trafficking | 1.68 | 0 | 0 | 0 | -2.35 |
| *dnaK* | lmo1473 | class I heat-shock protein (molecular chaperone) DnaK | Protein folding and stabilization | 1.68 | UP | -4.32 | 0 | 0.85 |
| *grpE* | lmo1474 | heat shock protein GrpE | Protein folding and stabilization | 1.6 | UP | -3.4 | 0 | -0.41 |
| *NA* | lmo1271 | similar to signal peptidase I | Protein and peptide secretion and trafficking | 1.54 | 0 | 0 | 0 | 1.48 |
| *clpB* | lmo2206 | similar to endopeptidase Clp ATP-binding chain B (ClpB) | Degradation of proteins, peptides, and glycopeptides | 1.43 | UP | 0 | 0 | -0.29 |
| *NA* | lmo1803 | similar to FtsY of E. coli and SRP receptor alpha-subunit | Protein and peptide secretion and trafficking | 1.26 | 0 | 0 | 0 | -0.19 |
| *birA* | LMOf2365_1933 | birA bifunctional protein | Protein modification and repair | 1.25 | 0 | 0 | 0 | 0 |
| *NA* | LMOf6854_1656 | aminopeptidase, putative | Degradation of proteins, peptides, and glycopeptides | -5.8 | 0 | 0 | 0 | 0 |
| *NA* | lmo1603 | similar to aminopeptidase | Degradation of proteins, peptides, and glycopeptides | -5.43 | DOWN | 0 | 0 | 1.58 |
| *spl* | lmo2505 | peptidoglycan lytic protein P45 | Degradation of proteins, peptides, and glycopeptides | -2.48 | DOWN | 0 | 0 | -0.21 |
| *secY* | lmo2612 | highly similar to preprotein translocase subunit | Protein and peptide secretion and trafficking | -2.48 | 0 | 0 | 0 | 1.17 |
| *gtcA* | lmo2549 | wall teichoic acid glycosylation protein GtcA | Protein modification and repair | -2.33 | 0 | 0 | 0 | 0.57 |
| *NA* | lmo1217 | similar to endo-1,4-beta-glucanase and to aminopeptidase | Degradation of proteins, peptides, and glycopeptides | -2.32 | 0 | 0 | 0 | 1.05 |
| *NA* | lmo0960 | similar to proteases | Degradation of proteins, peptides, and glycopeptides | -2.08 | 0 | 0 | -2.2 | -0.44 |
| *NA* | lmo1625 | similar to putative transporters | Protein and peptide secretion and trafficking | -2.06 | DOWN | -1.5 | 0 | -1.48 |
| *NA* | lmo1625 | similar to putative transporters | Degradation of proteins, peptides, and glycopeptides | -2.06 | DOWN | -1.5 | 0 | -1.48 |
| *secE* | lmo0245 | highly similar to preprotein translocase subunit | Protein and peptide secretion and trafficking | -1.98 | 0 | 0 | -2.4 | -1.14 |
| *NA* | lmo1585 | similar to proteases | Degradation of proteins, peptides, and glycopeptides | -1.87 | DOWN | 0 | 0 | 2.35 |
| *NA* | lmo0494 | weakly similar to esterase | Degradation of proteins, peptides, and glycopeptides | -1.79 | 0 | 0 | 0 | 0.22 |
| *NA* | lmo0678 | similar to flagellar biosynthetic protein FliR | Degradation of proteins, peptides, and glycopeptides | -1.77 | 0 | 1.88 | 0 | 0.8 |
| *NA* | lmo1620 | similar to Xaa-His dipeptidase | Degradation of proteins, peptides, and glycopeptides | -1.77 | 0 | 0 | 0 | 0.21 |
| *ffh* | lmo1801 | similar to signal recognition particle protein Ffh | Protein and peptide secretion and trafficking | -1.61 | 0 | 0 | 0 | -0.5 |
| *NA* | lmo1624 | similar to putative transporters | Protein and peptide secretion and trafficking | -1.54 | 0 | 0 | 0 | 0.73 |
| *NA* | lmo1624 | similar to putative transporters | Degradation of proteins, peptides, and glycopeptides | -1.54 | 0 | 0 | 0 | 0.73 |
| *lgt* | lmo2482 | highly similar to prolipoprotein diacylglyceryl transferase | Protein modification and repair | -1.49 | 0 | 0 | 0 | 0.5 |
| *NA* | LMOf2365_1735 | aminopeptidase | Degradation of proteins, peptides, and glycopeptides | -1.47 | 0 | 0 | 0 | 0 |
| *NA* | lmo2462 | similar to dipeptidases | Degradation of proteins, peptides, and glycopeptides | -1.38 | UP | 0 | 0 | -0.57 |
| *NA* | lmo0387 | similar to B. subtilis YhdG protein | Degradation of proteins, peptides, and glycopeptides | 0 | UP | -2.18 | 0 | 1.06 |
| *NA* | lmo1138 | similar to ATP-dependent Clp protease proteolytic component | Degradation of proteins, peptides, and glycopeptides | 0 | UP | 0 | 0 | -0.97 |
| *clpY* | lmo1279 | highly similar to ATP-dependent Clp protease-like proteins | Protein folding and stabilization | 0 | UP | 0 | 0 | 0.38 |
| *comGB* | lmo1346 | similar to B. subtilis comG operon protein 2 | Degradation of proteins, peptides, and glycopeptides | 0 | UP | 0 | 0 | 0.22 |
| *NA* | lmo1375 | similar to aminotripeptidase | Degradation of proteins, peptides, and glycopeptides | 0 | UP | 0 | 2.9 | 2.39 |
| *dnaJ* | lmo1472 | heat shock protein DnaJ | Protein folding and stabilization | 0 | UP | -2.44 | 0 | 0.05 |
| *groEL* | lmo2068 | class I heat-shock protein (chaperonin) GroEL | Protein folding and stabilization | 0 | UP | -5.73 | 0 | -0.6 |
| *groES* | lmo2069 | class I heat-shock protein (chaperonin) GroES | Protein folding and stabilization | 0 | UP | -5.82 | 0 | 0.8 |
| *ltrC* | lmo2398 | low temperature requirement C protein, also similar to B. subtilis YutG protein | Protein and peptide secretion and trafficking | 0 | UP | 0 | 7 | -0.96 |
| *ltrC* | lmo2398 | low temperature requirement C protein, also similar to B. subtilis YutG protein | Degradation of proteins, peptides, and glycopeptides | 0 | UP | 0 | 7 | -0.96 |
| *clpP* | lmo2468 | ATP-dependent Clp protease proteolytic subunit | Degradation of proteins, peptides, and glycopeptides | 0 | UP | 0 | 0 | -0.49 |
| *NA* | lmo0186 | similar to B. subtilis YabE protein | Degradation of proteins, peptides, and glycopeptides | 0 | DOWN | 0 | 0 | -1.27 |
| *NA* | lmo1529 | similar to unknown proteins | Protein and peptide secretion and trafficking | 0 | DOWN | 0 | 0 | 0.8 |
| *NA* | lmo2504 | similar to cell wall binding proteins | Degradation of proteins, peptides, and glycopeptides | 0 | DOWN | 0 | -2.7 | 0 |
| *NA* | lmo0680 | similar to flagella-associated protein flhA | Protein and peptide secretion and trafficking | 0 | 0 | 3.39 | 0 | 0.41 |
| *NA* | lmo0681 | similar to flagellar biosynthesis protein FlhF | Protein and peptide secretion and trafficking | 0 | 0 | 3.18 | 0 | 1.1 |
| *NA* | lmo1864 | similar to hemolysinIII proteins, putative integral membrane protein | Degradation of proteins, peptides, and glycopeptides | 0 | 0 | 4.09 | 0 | -1.11 |
| *pflC* | lmo1407 | pyruvate-formate lyase activating enzyme | Protein modification and repair | 0 | 0 | -2.05 | 0 | 0.59 |
| *NA* | lmo2451 | similar to preprotein translocase subunit SecG | Protein and peptide secretion and trafficking | 0 | 0 | 0 | -2.3 | 0.66 |
| *clpQ* | lmo1278 | highly similar to beta-type subunit of the 20S proteasome | Protein folding and stabilization | 0 | 0 | 0 | -1.1 | -1.18 |
| *NA* | lmo0062 | unknown | Degradation of proteins, peptides, and glycopeptides | 0 | 0 | 0 | 0 | 2.08 |
| *NA* | lmo0153 | similar to a probable high-affinity zinc ABC transporter (Zn(II)-binding lipoprotein) | Degradation of proteins, peptides, and glycopeptides | 0 | 0 | 0 | 0 | -1.32 |
| *NA* | lmo0191 | similar to a putative phospho-beta-glucosidase | Protein folding and stabilization | 0 | 0 | 0 | 0 | -0.82 |
| *mpl* | lmo0203 | Zinc metalloproteinase precursor | Degradation of proteins, peptides, and glycopeptides | 0 | 0 | 0 | 0 | -1.56 |
| *NA* | lmo0222 | conserved hypothetical protein | Protein folding and stabilization | 0 | 0 | 0 | 0 | 1.48 |
| *ltrA* | lmo0389 | low temperature requirement protein A | Protein and peptide secretion and trafficking | 0 | 0 | 0 | 0 | 3.25 |
| *ltrA* | lmo0389 | low temperature requirement protein A | Degradation of proteins, peptides, and glycopeptides | 0 | 0 | 0 | 0 | 3.25 |
| *NA* | lmo0764 | similar to lipoate-protein ligase | Protein modification and repair | 0 | 0 | 0 | 0 | 0.19 |
| *NA* | lmo0845 | similar to B. subtilis YxjH and YxjG proteins | Protein modification and repair | 0 | 0 | 0 | 0 | -0.78 |
| *NA* | lmo0931 | similar to lipoate protein ligase A | Protein modification and repair | 0 | 0 | 0 | 0 | -0.5 |
| *NA* | lmo0942 | similar to heat shock protein HtpG | Protein folding and stabilization | 0 | 0 | 0 | 0 | -0.92 |
| *NA* | lmo0961 | similar to proteases | Degradation of proteins, peptides, and glycopeptides | 0 | 0 | 0 | 0 | 0.34 |
| *NA* | lmo0963 | similar to putative heat shock protein HtpX, Listeria epitope LemB | Protein folding and stabilization | 0 | 0 | 0 | 0 | -0.34 |
| *NA* | lmo1051 | similar to formylmethionine deformylase and to B. subtilis YkrB protein | Protein modification and repair | 0 | 0 | 0 | 0 | 0.88 |
| *NA* | lmo1097 | similar to integrases | Degradation of proteins, peptides, and glycopeptides | 0 | 0 | 0 | 0 | 0 |
| *NA* | lmo1101 | similar to lipoprotein signal peptidase | Protein and peptide secretion and trafficking | 0 | 0 | 0 | 0 | -2.73 |
| *eutA* | lmo1174 | similar to ethanolamine utilization protein EutA (putative chaperonin) | Protein folding and stabilization | 0 | 0 | 0 | 0 | -1.79 |
| *tig* | lmo1267 | trigger factor (prolyl isomerase) | Protein and peptide secretion and trafficking | 0 | 0 | 0 | 0 | 2.51 |
| *tig* | lmo1267 | trigger factor (prolyl isomerase) | Protein folding and stabilization | 0 | 0 | 0 | 0 | 2.51 |
| *clpX* | lmo1268 | ATP-dependent Clp protease ATP-binding subunit ClpX | Degradation of proteins, peptides, and glycopeptides | 0 | 0 | 0 | 0 | 1.05 |
| *NA* | lmo1269 | similar to type-I signal peptidase | Protein and peptide secretion and trafficking | 0 | 0 | 0 | 0 | 0.81 |
| *NA* | lmo1270 | similar to signal peptidase I | Protein and peptide secretion and trafficking | 0 | 0 | 0 | 0 | -0.14 |
| *NA* | lmo1318 | conserved hypothetical protein similar to B. subtilis YluC protein | Degradation of proteins, peptides, and glycopeptides | 0 | 0 | 0 | 0 | -1.76 |
| *NA* | lmo1354 | similar to aminopeptidase P | Degradation of proteins, peptides, and glycopeptides | 0 | 0 | 0 | 0 | 1.66 |
| *NA* | lmo1493 | similar to oligopeptidase | Degradation of proteins, peptides, and glycopeptides | 0 | 0 | 0 | 0 | 0.92 |
| *comC* | lmo1550 | similar to B. subtilis late competence protein ComC (type IV prepilin peptidase) | Degradation of proteins, peptides, and glycopeptides | 0 | 0 | 0 | 0 | 0.67 |
| *NA* | lmo1578 | similar to X-Pro dipeptidase | Degradation of proteins, peptides, and glycopeptides | 0 | 0 | 0 | 0 | 0.48 |
| *NA* | lmo1611 | similar to aminopeptidase | Degradation of proteins, peptides, and glycopeptides | 0 | 0 | 0 | 0 | 0.03 |
| *NA* | lmo1666 | peptidoglycan linked protein (LPxTG) | Degradation of proteins, peptides, and glycopeptides | 0 | 0 | 0 | 0 | 1.25 |
| *NA* | lmo1671 | similar to ABC transporter and adhesion proteins | Degradation of proteins, peptides, and glycopeptides | 0 | 0 | 0 | 0 | 0.11 |
| *NA* | lmo1709 | similar to methionine aminopeptidases | Protein modification and repair | 0 | 0 | 0 | 0 | 0.64 |
| *NA* | lmo1711 | highly similar to aminopeptidases | Degradation of proteins, peptides, and glycopeptides | 0 | 0 | 0 | 0 | 2 |
| *NA* | lmo1780 | similar to aminotripeptidase (peptidase T) | Degradation of proteins, peptides, and glycopeptides | 0 | 0 | 0 | 0 | 0.8 |
| *lsp* | lmo1844 | highly similar to signal peptidase II | Protein and peptide secretion and trafficking | 0 | 0 | 0 | 0 | -0.67 |
| *NA* | lmo1851 | similar to carboxy-terminal processing proteinase | Degradation of proteins, peptides, and glycopeptides | 0 | 0 | 0 | 0 | 0.04 |
| *NA* | lmo1860 | similar to peptidyl methionine sulfoxide reductases | Protein modification and repair | 0 | 0 | 0 | 0 | 1.18 |
| *NA* | lmo1886 | similar to probable thermostable carboxypeptidases | Degradation of proteins, peptides, and glycopeptides | 0 | 0 | 0 | 0 | -0.06 |
| *NA* | lmo2075 | similar to glycoprotein endopeptidase | Degradation of proteins, peptides, and glycopeptides | 0 | 0 | 0 | 0 | 2.18 |
| *NA* | lmo2077 | similar to glycoprotease | Degradation of proteins, peptides, and glycopeptides | 0 | 0 | 0 | 0 | 1.07 |
| *NA* | lmo2188 | similar to oligoendopeptidase | Degradation of proteins, peptides, and glycopeptides | 0 | 0 | 0 | 0 | -1.09 |
| *NA* | lmo2280 | protein gp23 [Bacteriophage A118] | Protein and peptide secretion and trafficking | 0 | 0 | 0 | 0 | 0.08 |
| *pepC* | lmo2338 | aminopeptidase C | Degradation of proteins, peptides, and glycopeptides | 0 | 0 | 0 | 0 | -0.74 |
| *NA* | lmo2376 | similar to peptidyl-prolyl cis-trans isomerase | Protein folding and stabilization | 0 | 0 | 0 | 0 | -0.96 |
| *NA* | lmo2566 | unknown | Protein modification and repair | 0 | 0 | 0 | 0 | 0.59 |
| **Regulatory functions** | | |  |  |  |  |  |  |
| *NA* | lmo1974 | similar to transcription regulators, (GntR family) | Other | 5.12 | UP | -2.27 | 0 | 1.49 |
| *NA* | LMOf2365_2818 | transcriptional regulator, MarR family | DNA interactions | 4.25 | 0 | 0 | 0 | 0 |
| *NA* | LMOf2365_2669 | transcriptional regulator, TetR family | DNA interactions | 3.22 | 0 | 0 | 0 | 0 |
| *NA* | lmo2690 | similar to transcription regulator, TetR family | DNA interactions | 3 | 0 | 0 | 0 | -5.13 |
| *NA* | lmo2088 | similar to transcription regulators | Other | 2.99 | 0 | 0 | 0 | -0.48 |
| *NA* | lmo2200 | similar to transcription regulator | Other | 2.77 | UP | 3.03 | 0 | -0.63 |
| *NA* | lmo1302 | highly similar to SOS response regulator lexA, transcription repressor protein | DNA interactions | 2.59 | 0 | 0 | 0 | 0.75 |
| *NA* | lmo0651 | similar to transcription regulator | DNA interactions | 2.5 | 0 | 0 | 0 | 0.4 |
| *NA* | lmo0655 | similar to phosphoprotein phosphatases | Other | 2.26 | UP | 0 | 2.5 | -1.63 |
| *NA* | lmo2714 | peptidoglycan anchored protein (LPXTG motif) | Other | 2.21 | 0 | 0 | 0 | 1.13 |
| *NA* | lmo0360 | similar to transcriptional regulator (DeoR family) | Other | 2.11 | 0 | 0 | 0 | -0.37 |
| *NA* | lmo2514 | similar to B. subtilis YviA (DegV) protein | Other | 1.98 | 0 | 0 | 0 | -0.05 |
| *NA* | lmo2515 | similar to B. subtilis two-component response regulator DegU | Other | 1.94 | 0 | 0 | 0 | -3.28 |
| *NA* | lmo0352 | highly similar to regulatory proteins (DeoR family) | Other | 1.92 | UP | 0 | 0 | 0.75 |
| *NA* | lmo0958 | similar to transcription regulator (GntR family) | Other | 1.74 | UP | 0 | 2 | 0.66 |
| *NA* | lmo0852 | similar to transcription regulator TetR/AcrR family | DNA interactions | 1.67 | 0 | 0 | 0 | 1.08 |
| *fur* | lmo1956 | similar to transcriptional regulator (Fur family) | Other | 1.6 | 0 | 0 | 0 | 0.27 |
| *NA* | lmo0443 | similar to B. subtilis transcription regulator LytR | Other | 1.55 | DOWN | 0 | -2 | -1.35 |
| *NA* | LMOf2365_1907 | iron-dependent repressor family protein | DNA interactions | 1.51 | 0 | 0 | 0 | 0 |
| *NA* | lmo1253 | similar to transcription regulator GntR family | Other | 1.49 | 0 | 0 | 0 | 1.91 |
| *NA* | lmo0858 | similar to transcription regulator lacI family | Other | 1.45 | UP | 0 | 0 | 0.1 |
| *NA* | lmo1189 | similar to transcriptional regulator | Other | 1.44 | UP | 0 | -2 | -1.36 |
| *NA* | LMOf2365_2307 | transcriptional regulator, DeoR family | DNA interactions | 1.38 | 0 | 0 | 0 | 0 |
| *NA* | LMOf6854_0460 | transcriptional antiterminator, bglG family, putative | DNA interactions | 1.3 | 0 | 0 | 0 | 0 |
| *NA* | LMOf6854_0460 | transcriptional antiterminator, bglG family, putative | RNA interactions | 1.3 | 0 | 0 | 0 | 0 |
| *birA* | LMOf2365_1933 | birA bifunctional protein | DNA interactions | 1.25 | 0 | 0 | 0 | 0 |
| *ccpA* | lmo1599 | catabolite control protein A | Other | 1.22 | 0 | 0 | 0 | 0.75 |
| *NA* | LMOf6854_1937 | iron-dependent repressor family protein | DNA interactions | 1.22 | 0 | 0 | 0 | 0 |
| *NA* | LMOf2365_1868 | pyrimidine operon regulatory protein PyrR | RNA interactions | -4.22 | 0 | 0 | 0 | 0 |
| *NA* | lmo2128 | similar to transcription regulator, LacI family | Other | -2.49 | 0 | 0 | 0 | -1.7 |
| *NA* | lmo2352 | similar to LysR family transcription regulator | Other | -2.36 | 0 | 0 | 2 | 1.1 |
| *NA* | LMOf6854_0303 | sensory box histidine kinase | Protein interactions | -1.98 | 0 | 0 | 0 | 0 |
| *NA* | lmo2243 | similar to methylphosphotriester-DNA alkyltransferase and transcriptional regulator | Other | -1.9 | 0 | 0 | 0 | -0.05 |
| *NA* | LMOf2365_1964 | putative protein-tyrosine phosphatase | Protein interactions | -1.82 | 0 | 0 | 0 | 0 |
| *NA* | lmo1262 | similar to transcriptional regulator (phage-related) | Other | -1.8 | 0 | 0 | 0 | 0.62 |
| *NA* | lmo0192 | similar to PurR, transcription repressor of purine operon of B. subtilis | Other | -1.79 | 0 | 0 | 0 | 0.45 |
| *NA* | LMOh7858_0267 | B. subtilis YazC protein homolog lmo0240 [imported] | Other | -1.74 | 0 | 0 | 0 | 0 |
| *NA* | lmo1850 | similar to transcriptional regulator (MarR family) | Other | -1.7 | 0 | 0 | 0 | 0.41 |
| *NA* | LMOf6854_2845 | transcriptional regulator, MarR family | DNA interactions | -1.69 | 0 | 0 | 0 | 0 |
| *NA* | lmo1225 | similar to transcriptional regulator (MarR family). | Other | -1.67 | 0 | 0 | 0 | -0.68 |
| *lisR* | lmo1377 | two-component response regulator | Other | -1.61 | 0 | 0 | 0 | -0.74 |
| *NA* | lmo2483 | HPr-P(Ser) kinase/phosphatase | Protein interactions | -1.6 | 0 | 0 | 0 | 1.35 |
| *NA* | lmo2366 | similar to transcription regulator DeoR family | Other | -1.59 | 0 | 0 | 0 | -0.67 |
| *NA* | lmo0483 | unknown | Other | -1.56 | 0 | 0 | 0 | 1.23 |
| *kdpD* | LMOf6854_2795 | sensor histidine kinase KdpD | Protein interactions | -1.54 | 0 | 0 | 0 | 0 |
| *NA* | LMOf2365_0518 | transcriptional regulator, LysR family | DNA interactions | -1.48 | 0 | 0 | 0 | 0 |
| *NA* | lmo1411 | unknown | Other | -1.45 | 0 | 0 | 0 | -0.46 |
| *NA* | LMOf6854_2056 | transcriptional regulator, putative | DNA interactions | -1.36 | 0 | 0 | 0 | 0 |
| *NA* | lmo0207 | hypothetical lipoprotein | Other | -1.32 | 0 | 0 | 0 | -1.84 |
| *NA* | lmo0888 | similar to B. subtilis YdcE protein | Other | -1.3 | 0 | 0 | 0 | -1.22 |
| *NA* | lmo2421 | similar to two-component sensor histidine kinase | Other | -1.19 | 0 | 0 | 0 | -0.55 |
| *NA* | lmo0109 | similar to transcriptional regulatory proteins, AraC family | Other | 0 | UP | 0 | 0 | -0.39 |
| *prfA* | lmo0200 | listeriolysin positive regulatory protein | Other | 0 | UP | -2.56 | 0 | 0.12 |
| *NA* | lmo0520 | similar to transcription regulator | Other | 0 | UP | 0 | 0 | 2.7 |
| *NA* | lmo0606 | similar to transcription regulator MarR family | DNA interactions | 0 | UP | 0 | 1.5 | -1.64 |
| *NA* | lmo0770 | similar to transcriptional regulator (LacI family) | Other | 0 | UP | 0 | 0 | 1.48 |
| *NA* | lmo0806 | similar to transcription regulator | Other | 0 | UP | 0 | 0 | 3.01 |
| *NA* | lmo0822 | similar to transcriptional regulators | Other | 0 | UP | -2.17 | 0 | -0.52 |
| *NA* | lmo0902 | similar to transcription regulator (GntR family) | Other | 0 | UP | 0 | 0 | 0.56 |
| *NA* | lmo0989 | similar to regulatory proteins (MarR family) | Other | 0 | UP | 0 | 0 | -0.2 |
| *NA* | lmo1295 | similar to host factor-1 protein | Other | 0 | UP | 0 | 2.5 | 0.44 |
| *NA* | lmo1367 | similar to arginine repressor | Other | 0 | UP | 0 | 0 | 0.27 |
| *hrcA* | lmo1475 | transcription repressor of class I heat-shock gene HrcA | DNA interactions | 0 | UP | -3.62 | 2.8 | 1.92 |
| *NA* | lmo2003 | similar to transcription regulator GntR family | Other | 0 | UP | 0 | 0 | 2.2 |
| *NA* | lmo2004 | similar to transcription regulator GntR family | Other | 0 | UP | 0 | 0 | -0.11 |
| *NA* | lmo2027 | putative cell surface protein, similar to internalin proteins | Other | 0 | UP | 0 | 0 | 0.35 |
| *NA* | lmo2085 | putative peptidoglycan bound protein (LPXTG motif) | Other | 0 | UP | 0 | 5 | 0.17 |
| *NA* | lmo2107 | similar to transcriptional regulator (DeoR family) | Other | 0 | UP | 0 | 0 | -1.55 |
| *NA* | lmo2329 | similar to a putative repressor protein [Bacteriophage A118] | Other | 0 | UP | 0 | 0 | -0.9 |
| *NA* | lmo2337 | similar to regulatory protein DeoR family | Other | 0 | UP | 6.35 | 0 | -1.44 |
| *NA* | lmo2493 | similar to transcription regulator ArsR family | Other | 0 | UP | 0 | 0 | -1.12 |
| *NA* | lmo2672 | weakly similar to transcription regulator | Other | 0 | UP | 0 | 0 | 0.68 |
| *NA* | lmo2678 | similar to two components response regulator | Other | 0 | UP | 0 | 0 | 0.17 |
| *NA* | lmo2784 | similar to lichenan operon transcription antiterminator licR | Other | 0 | UP | 0 | 0 | 0.48 |
| *NA* | lmo2842 | similar to transcriptional regulator | Other | 0 | UP | 0 | 0 | -3.74 |
| *NA* | lmo0048 | similar to Staphylococcus two-component sensor histidine kinase AgrB | Other | 0 | DOWN | 0 | 0 | -0.07 |
| *NA* | lmo0051 | similar to 2-components response regulator protein (AgrA from Staphylococcus) | Other | 0 | DOWN | 0 | 0 | -1.09 |
| *NA* | lmo1026 | similar to B. subtilis LytR protein | Other | 0 | DOWN | 0 | 0 | -0.74 |
| *NA* | lmo1618 | similar to transcription regulator MarR family | Other | 0 | DOWN | 0 | 0 | 0.39 |
| *NA* | lmo1911 | similar to unknown proteins (hypothetical sensory transduction histidine kinase) | Other | 0 | DOWN | 0 | 0 | -0.77 |
| *NA* | lmo2518 | similar to B. subtilis putative transcriptional regulator LytR | Other | 0 | DOWN | -2.15 | 0 | -0.32 |
| *NA* | lmo2145 | similar to unknown protein | Other | 0 | 0 | 2.58 | 0 | -0.63 |
| *NA* | lmo2460 | similar to B. subtilis CggR hypothetical transcriptional regulator | Other | 0 | 0 | 2.62 | 1 | 0.24 |
| *glnR* | lmo1298 | similar to glutamine synthetase repressor | Other | 0 | 0 | -3.54 | 0 | -0.27 |
| *NA* | lmo0402 | similar to transcriptional antiterminator (BglG family) | Other | 0 | 0 | 0 | 1.1 | 2.16 |
| *NA* | lmo0445 | similar to transcription regulator | Other | 0 | 0 | 0 | 2.5 | 0.41 |
| *NA* | lmo2739 | similar to regulatory proteins of the SIR2 family | Other | 0 | 0 | 0 | 2.2 | -0.64 |
| *NA* | lmo0788 | unknown | Other | 0 | 0 | 0 | 2 | 0.39 |
| *NA* | lmo0020 | similar to transcriptional regulator (GntR family) | Other | 0 | 0 | 0 | 0 | 1.46 |
| *NA* | lmo0076 | similar to E. coli Ada protein (O6-methylguanine-DNA methyltransferase) | Other | 0 | 0 | 0 | 0 | 1.04 |
| *NA* | lmo0083 | similar to transcription regulator (merR family) | Other | 0 | 0 | 0 | 0 | -0.55 |
| *NA* | lmo0101 | similar to transcription regulator | Other | 0 | 0 | 0 | 0 | -0.49 |
| *NA* | lmo0131 | conserved hypothetical protein | Other | 0 | 0 | 0 | 0 | -2.01 |
| *NA* | lmo0160 | putative peptidoglycan bound protein (LPXTG motif) | Other | 0 | 0 | 0 | 0 | -0.76 |
| *NA* | lmo0161 | unknown | Protein interactions | 0 | 0 | 0 | 0 | -1.33 |
| *NA* | lmo0168 | similar to B. subtilis transcription regulatory protein AbrB | Other | 0 | 0 | 0 | 0 | -1.86 |
| *NA* | lmo0178 | similar to xylose repressor | Other | 0 | 0 | 0 | 0 | -1.04 |
| *NA* | lmo0191 | similar to a putative phospho-beta-glucosidase | Other | 0 | 0 | 0 | 0 | -0.82 |
| *NA* | lmo0221 | conserved hypothetical protein | Other | 0 | 0 | 0 | 0 | 1.69 |
| *NA* | lmo0255 | similar to unknown protein | Other | 0 | 0 | 0 | 0 | -0.79 |
| *NA* | lmo0266 | similar to transcriptional regulators | Other | 0 | 0 | 0 | 0 | -1.57 |
| *NA* | lmo0288 | similar to two-component sensor histidine kinase | Other | 0 | 0 | 0 | 0 | 0.1 |
| *NA* | lmo0294 | similar to transcription regulator LysR-gltR family | Other | 0 | 0 | 0 | 0 | -1.77 |
| *NA* | lmo0297 | similar to transcriptional antiterminator (BglG family) | Other | 0 | 0 | 0 | 0 | -0.8 |
| *NA* | lmo0315 | similar to thiamin biosynthesis protein | Other | 0 | 0 | 0 | 0 | -3.25 |
| *NA* | lmo0325 | similar to transcriptional regulators | Other | 0 | 0 | 0 | 0 | 1.45 |
| *NA* | lmo0327 | similar to cell surface proteins (LPXTG motif) | Other | 0 | 0 | 0 | 0 | -1.43 |
| *NA* | lmo0371 | similar to transcription regulator (GntR family) | Other | 0 | 0 | 0 | 0 | 0.73 |
| *NA* | lmo0416 | similar to putative transcription regulator | Other | 0 | 0 | 0 | 0 | 0.71 |
| *NA* | lmo0425 | similar to transcription antiterminator BglG family | Other | 0 | 0 | 0 | 0 | -3.42 |
| *NA* | lmo0427 | similar to PTS fructose-specific enzyme IIB component | Other | 0 | 0 | 0 | 0 | 2.12 |
| *NA* | lmo0430 | similar to transcriptional regulator (LysR family) | Other | 0 | 0 | 0 | 0 | 0.95 |
| *NA* | lmo0480 | similar to putative transcriptional regulator | Other | 0 | 0 | 0 | 0 | 0.63 |
| *NA* | lmo0488 | similar to transcriptional regulator (LysR family) | Other | 0 | 0 | 0 | 0 | 2.11 |
| *NA* | lmo0492 | similar to transcriptional regulator (LysR family) | Other | 0 | 0 | 0 | 0 | 1.07 |
| *NA* | lmo0501 | similar to transcription antiterminator BglG family | Other | 0 | 0 | 0 | 0 | 1.27 |
| *NA* | lmo0513 | weakly similar to transcription regulator | Other | 0 | 0 | 0 | 0 | 0.15 |
| *NA* | lmo0526 | similar to transcription regulator (TipA from Streptomyces coelicolor) | Other | 0 | 0 | 0 | 0 | -1.12 |
| *NA* | lmo0535 | similar to transcription regulator (LacI family) | Other | 0 | 0 | 0 | 0 | 1.25 |
| *NA* | lmo0547 | similar to B. subtilis DeoR transcriptional regulator | Other | 0 | 0 | 0 | 0 | -1.11 |
| *NA* | lmo0549 | similar to internalin protein | Other | 0 | 0 | 0 | 0 | 2.7 |
| *NA* | lmo0575 | similar to transcription regulator GntR family | Other | 0 | 0 | 0 | 0 | -1 |
| *NA* | lmo0612 | similar to transcription regulator MarR family | Other | 0 | 0 | 0 | 0 | 1.05 |
| *NA* | lmo0630 | similar to transcription antiterminator BglG family | Other | 0 | 0 | 0 | 0 | 2.04 |
| *NA* | lmo0639 | similar to a transcription regulator (surface protein PAg negative regulator par) | Other | 0 | 0 | 0 | 0 | 0.33 |
| *NA* | lmo0649 | similar to transcription regulators | Other | 0 | 0 | 0 | 0 | -0.01 |
| *NA* | lmo0659 | similar to transcription regulator (Rgg type) | Other | 0 | 0 | 0 | 0 | -0.73 |
| *cheY* | lmo0691 | Chemotaxis response regulator CheY | Other | 0 | 0 | 0 | 0 | 0.2 |
| *NA* | lmo0725 | putative peptidoglycan bound protein (LPXTG motif) | Other | 0 | 0 | 0 | 0 | 0.04 |
| *NA* | lmo0733 | similar to transcription regulator | Other | 0 | 0 | 0 | 0 | 0.23 |
| *NA* | lmo0734 | similar to transcriptional regulator (LacI family) | Other | 0 | 0 | 0 | 0 | -0.28 |
| *NA* | lmo0740 | similar to putative transcription regulator | Other | 0 | 0 | 0 | 0 | -0.24 |
| *NA* | lmo0741 | similar to transcriptional regulator (GntR family) | Other | 0 | 0 | 0 | 0 | 0.74 |
| *NA* | lmo0749 | unknown | Other | 0 | 0 | 0 | 0 | -6.19 |
| *NA* | lmo0753 | similar to transcription regulator Crp/Fnr family | Other | 0 | 0 | 0 | 0 | -0.75 |
| *NA* | lmo0772 | similar to transcription regulator | DNA interactions | 0 | 0 | 0 | 0 | 0.88 |
| *NA* | lmo0785 | similar to transcriptional regulator (NifA/NtrC family) | Other | 0 | 0 | 0 | 0 | -0.98 |
| *NA* | lmo0797 | unknown | DNA interactions | 0 | 0 | 0 | 0 | -0.47 |
| *NA* | lmo0799 | unknown | Other | 0 | 0 | 0 | 0 | -0.37 |
| *NA* | lmo0802 | weakly similar to GTP-pyrophosphokinase | Other | 0 | 0 | 0 | 0 | -0.72 |
| *NA* | lmo0812 | unknown | Other | 0 | 0 | 0 | 0 | -2.62 |
| *NA* | lmo0815 | similar to transcription regulators | Other | 0 | 0 | 0 | 0 | -2.74 |
| *NA* | lmo0833 | similar to transcriptional regulator | Other | 0 | 0 | 0 | 0 | -1.19 |
| *NA* | lmo0835 | putative peptidoglycan bound protein (LPXTG motif) | Other | 0 | 0 | 0 | 0 | 0.91 |
| *NA* | lmo0840 | unknown | Other | 0 | 0 | 0 | 0 | -0.59 |
| *NA* | lmo0873 | Similar to transcriptional regulator (antiterminator) | Other | 0 | 0 | 0 | 0 | 1.14 |
| *NA* | lmo0887 | similar to B. subtilis YdcD protein | DNA interactions | 0 | 0 | 0 | 0 | 0.48 |
| *RsbR* | lmo0889 | highly similar to positive regulator of sigma-B activity | Other | 0 | 0 | 0 | 0 | -0.12 |
| *NA* | lmo0899 | similar to B. subtilis YdcK protein | Other | 0 | 0 | 0 | 0 | 0.9 |
| *NA* | lmo0909 | similar to transcription regulator, GntR family | Other | 0 | 0 | 0 | 0 | -1.72 |
| *NA* | lmo0918 | similar to transcription antiterminator BglG family | Other | 0 | 0 | 0 | 0 | -0.65 |
| *NA* | lmo0926 | similar to transcription regulator (TetR/AcrR family) | DNA interactions | 0 | 0 | 0 | 0 | 0.51 |
| *NA* | lmo0967 | similar to B. subtilis YjbM protein | Other | 0 | 0 | 0 | 0 | -1.1 |
| *NA* | lmo1010 | similar to transcription regulator (LysR family). | Other | 0 | 0 | 0 | 0 | -1.35 |
| *NA* | lmo1021 | similar to two-component sensor histidine kinase in particular B. subtilis YvqE protein | Other | 0 | 0 | 0 | 0 | 0.58 |
| *NA* | lmo1022 | similar to two-component response regulator, in particular B. subtilis YvqC protein | Other | 0 | 0 | 0 | 0 | -1.38 |
| *NA* | lmo1030 | similar to transcriptional regulator, LacI family | Other | 0 | 0 | 0 | 0 | -1.43 |
| *NA* | lmo1060 | similar to transcription response regulator | Other | 0 | 0 | 0 | 0 | 0.85 |
| *NA* | lmo1061 | similar to two-component sensor histidine kinase | Other | 0 | 0 | 0 | 0 | 0.87 |
| *NA* | lmo1102 | similar to cadmium efflux system accessory proteins | Other | 0 | 0 | 0 | 0 | -0.56 |
| *NA* | lmo1116 | similar to regulatory proteins | DNA interactions | 0 | 0 | 0 | 0 | -1.92 |
| *NA* | lmo1134 | similar to regulatory proteins | Other | 0 | 0 | 0 | 0 | -1.1 |
| *NA* | lmo1150 | Regulatory protein similar to Salmonella typhimurium PocR protein | Other | 0 | 0 | 0 | 0 | -0.79 |
| *NA* | lmo1172 | similar to similar to two-component response regulator | Other | 0 | 0 | 0 | 0 | 1.13 |
| *NA* | lmo1173 | similar to two-component sensor histidine kinase | Other | 0 | 0 | 0 | 0 | -0.87 |
| *NA* | lmo1251 | similar to regulator of the Fnr CRP family (including PrfA) | Other | 0 | 0 | 0 | 0 | 0.26 |
| *NA* | lmo1263 | similar to transcriptional regulator | DNA interactions | 0 | 0 | 0 | 0 | 1.59 |
| *codY* | lmo1280 | highly similar to B. subtilis CodY protein | Other | 0 | 0 | 0 | 0 | 0.58 |
| *NA* | lmo1289 | similar to internalin proteins, putative peptidoglycan bound protein (LPXTG motif) | Other | 0 | 0 | 0 | 0 | 0.56 |
| *NA* | lmo1311 | unknown | Other | 0 | 0 | 0 | 0 | -0.38 |
| *lisK* | lmo1378 | two-component sensor histidine kinase | Other | 0 | 0 | 0 | 0 | 0.41 |
| *zurR* | lmo1445 | transcriptional regulator ZurR (ferric uptake regulation) | Other | 0 | 0 | 0 | 0 | -0.44 |
| *NA* | lmo1462 | similar to GTP binding proteins | RNA interactions | 0 | 0 | 0 | 0 | -0.76 |
| *NA* | lmo1478 | similar to transcriptional regulator (MerR family) | Other | 0 | 0 | 0 | 0 | -0.89 |
| *NA* | lmo1507 | similar to two-component response regulators | Other | 0 | 0 | 0 | 0 | -0.15 |
| *NA* | lmo1508 | similar to two-component sensor histidine kinase | Other | 0 | 0 | 0 | 0 | -0.3 |
| *NA* | lmo1517 | similar to nitrogen regulatory PII protein | Protein interactions | 0 | 0 | 0 | 0 | 2.38 |
| *relA* | lmo1523 | similar to (p)ppGpp synthetase | Other | 0 | 0 | 0 | 0 | 1.85 |
| *NA* | lmo1642 | similar to putative sigma factor regulator | Other | 0 | 0 | 0 | 0 | 1.83 |
| *NA* | lmo1683 | similar to transcription regulators (Fur family), PerR in B. subtilis | Other | 0 | 0 | 0 | 0 | 0.4 |
| *NA* | lmo1693 | similar to hypothetical proteins | Other | 0 | 0 | 0 | 0 | 1.93 |
| *NA* | lmo1716 | similar to putative transcription regulators | DNA interactions | 0 | 0 | 0 | 0 | -1.55 |
| *NA* | lmo1721 | similar to transcriptional regulator (NifA/NtrC family) | Other | 0 | 0 | 0 | 0 | 1.89 |
| *NA* | lmo1725 | similar to transcriptional regulator (GntR family) | Other | 0 | 0 | 0 | 0 | 1.62 |
| *NA* | lmo1727 | similar to transcription regulators (LacI family) | Other | 0 | 0 | 0 | 0 | -0.17 |
| *gltC* | lmo1735 | transcription activator of glutamate synthase operon GltC | Other | 0 | 0 | 0 | 0 | -0.35 |
| *NA* | lmo1741 | similar to two-component sensor histidine kinase | Other | 0 | 0 | 0 | 0 | 1 |
| *NA* | lmo1745 | similar to two-component response regulator | Other | 0 | 0 | 0 | 0 | 3.16 |
| *NA* | lmo1788 | similar to transcription regulator | Other | 0 | 0 | 0 | 0 | 0.23 |
| *NA* | lmo1820 | similar to putative serine/threonine-specific protein kinase | Other | 0 | 0 | 0 | 0 | -0.26 |
| *NA* | lmo1842 | similar to unknown proteins | Other | 0 | 0 | 0 | 0 | 0.61 |
| *NA* | lmo1863 | similar to hypothetical proteins | Other | 0 | 0 | 0 | 0 | 0.83 |
| *NA* | lmo1878 | similar o transcriptional regulators | Other | 0 | 0 | 0 | 0 | 0.06 |
| *birA* | lmo1904 | similar to transcriptional regulator and biotin acetyl-CoA-carboxylase synthetase | Other | 0 | 0 | 0 | 0 | -0.41 |
| *resE* | lmo1947 | similar to two-component sensor histidine kinase (ResE) | Other | 0 | 0 | 0 | 0 | -1.59 |
| *resD* | lmo1948 | similar to two-component response regulator (ResD) | Other | 0 | 0 | 0 | 0 | -0.33 |
| *NA* | lmo1962 | similar to transcription regulators (TetR family) | Other | 0 | 0 | 0 | 0 | -0.28 |
| *NA* | lmo1994 | similar to transcription regulators (LacI family) | Other | 0 | 0 | 0 | 0 | -0.9 |
| *NA* | lmo1996 | similar to transcription repressor of dra/nupC/pdp operon DeoR | Other | 0 | 0 | 0 | 0 | -0.9 |
| *NA* | lmo2010 | similar to two-component response regulator | Other | 0 | 0 | 0 | 0 | 1.35 |
| *NA* | lmo2011 | similar to two-component sensor histidine kinase | Other | 0 | 0 | 0 | 0 | 0.65 |
| *NA* | lmo2021 | similar to unknown protein | Other | 0 | 0 | 0 | 0 | -0.14 |
| *NA* | lmo2026 | putative peptidoglycan bound protein (LPXTG motif) | Other | 0 | 0 | 0 | 0 | -0.65 |
| *NA* | lmo2099 | similar to transcription antiterminator | Other | 0 | 0 | 0 | 0 | -1.87 |
| *NA* | lmo2100 | similar to transcriptional regulator (GntR family) and to aminotransferase (MocR-like) | Other | 0 | 0 | 0 | 0 | -2.54 |
| *NA* | lmo2144 | similar to transcription regulator GntR family | Other | 0 | 0 | 0 | 0 | -1.85 |
| *NA* | lmo2146 | similar to transcription regulator LysR family | Other | 0 | 0 | 0 | 0 | -0.58 |
| *NA* | lmo2173 | similar to sigma-54-dependent transcriptional activator | Other | 0 | 0 | 0 | 0 | -1.32 |
| *NA* | lmo2176 | similar to transcriptional regulator (tetR family) | Other | 0 | 0 | 0 | 0 | -0.43 |
| *NA* | lmo2178 | putative peptidoglycan bound protein (LPXTG motif) | Other | 0 | 0 | 0 | 0 | 1.19 |
| *NA* | lmo2179 | putative peptidoglycan bound protein (LPXTG motif) | Other | 0 | 0 | 0 | 0 | -0.36 |
| *NA* | lmo2233 | similar to transcriptional regulators (LysR family) | Other | 0 | 0 | 0 | 0 | -0.5 |
| *NA* | lmo2241 | similar to transcriptional regulators (GntR family) | Other | 0 | 0 | 0 | 0 | 0.07 |
| *comK* | lmo2270 | similar to competence transcription factor ComK, N terminal part | DNA interactions | 0 | 0 | 0 | 0 | -1.02 |
| *NA* | lmo2328 | similar to transcription regulator | Other | 0 | 0 | 0 | 0 | 0.28 |
| *NA* | lmo2334 | similar to transcriptional regulator | DNA interactions | 0 | 0 | 0 | 0 | 0.74 |
| *NA* | lmo2365 | similar to S. pyogenes RofA regulatory protein | Other | 0 | 0 | 0 | 0 | 0.33 |
| *NA* | lmo2396 | similar to internalin proteins, putative peptidoglycan bound protein (LPXTG motif) | Other | 0 | 0 | 0 | 0 | -0.83 |
| *NA* | lmo2408 | similar to repressor protein | Other | 0 | 0 | 0 | 0 | 0.15 |
| *NA* | lmo2422 | similar to two-component response regulator | Other | 0 | 0 | 0 | 0 | -1.34 |
| *NA* | lmo2445 | similar to internalin | Other | 0 | 0 | 0 | 0 | 0.94 |
| *NA* | lmo2447 | similar to transcription regulator | DNA interactions | 0 | 0 | 0 | 0 | -0.51 |
| *NA* | lmo2464 | similar to transcription regulator | Other | 0 | 0 | 0 | 0 | 0.39 |
| *phoR* | lmo2500 | two-component sensor histidine kinase | Other | 0 | 0 | 0 | 0 | -0.33 |
| *phoP* | lmo2501 | two-component response phosphate regulator | Other | 0 | 0 | 0 | 0 | -1.67 |
| *comFA* | lmo2513 | similar to late competence protein comFA | Other | 0 | 0 | 0 | 0 | 0.14 |
| *NA* | lmo2582 | similar to two-component sensor histidine kinase | Other | 0 | 0 | 0 | 0 | -2.63 |
| *NA* | lmo2583 | similar to two-component response regulator | Other | 0 | 0 | 0 | 0 | -0.14 |
| *NA* | lmo2593 | similar to transcription regulators (MerR family) | Other | 0 | 0 | 0 | 0 | 1.53 |
| *NA* | lmo2668 | similar to transcriptional antiterminator (BglG family) | Other | 0 | 0 | 0 | 0 | 2.26 |
| *NA* | lmo2698 | similar to putative transcription regulator | Other | 0 | 0 | 0 | 0 | 0.99 |
| *NA* | lmo2726 | similar to transcription regulators | Other | 0 | 0 | 0 | 0 | -0.55 |
| *NA* | lmo2728 | similar to transcription regulator MerR family | Other | 0 | 0 | 0 | 0 | 0.74 |
| *NA* | lmo2737 | similar to transcriptional regulator (LacI family) | Other | 0 | 0 | 0 | 0 | 0.38 |
| *NA* | lmo2744 | weakly similar to transcription regulators CRP/FNR family | Other | 0 | 0 | 0 | 0 | 1.19 |
| *NA* | lmo2764 | similar to xylose operon regulatory protein and to glucose kinase | Other | 0 | 0 | 0 | 0 | -0.5 |
| *parA* | lmo2791 | Partition protein, ParA homolog | Other | 0 | 0 | 0 | 0 | -1.24 |
| *NA* | lmo2792 | unknown | DNA interactions | 0 | 0 | 0 | 0 | 0.27 |
| *NA* | lmo2814 | similar to transcriptional regulator | DNA interactions | 0 | 0 | 0 | 0 | 0.21 |
| *NA* | lmo2820 | amino-terminal domain similar to transcription regulators | Other | 0 | 0 | 0 | 0 | -0.07 |
| *NA* | lmo2821 | similar to internalin, Unknown, putative peptidoglycan bound protein (LPXTG motif) | Other | 0 | 0 | 0 | 0 | 1.31 |
| **Signal transduction** | | |  |  |  |  |  |  |
| *NA* | LMOh7858_0838 | PTS system, mannose/fructose/sorbose family, IIA component subfamily | PTS | 3.24 | 0 | 0 | 0 | 0 |
| *NA* | LMOf2365_2663 | PTS system, beta-glucoside-specific, IIB component | PTS | 2.9 | 0 | 0 | 0 | 0 |
| *NA* | LMOf6854_0303 | sensory box histidine kinase | Two-component systems | -1.98 | 0 | 0 | 0 | 0 |
| *treB* | LMOf6854_1298 | PTS system, trehalose-specific, IIBC component | PTS | -1.97 | 0 | 0 | 0 | 0 |
| *NA* | LMOf6854_1148 | PTS system, IIB component, putative | PTS | -1.86 | 0 | 0 | 0 | 0 |
| *kdpD* | LMOf6854_2795 | sensor histidine kinase KdpD | Two-component systems | -1.54 | 0 | 0 | 0 | 0 |
| *NA* | lmo0034 | similar to PTS system, cellobiose-specific IIC component | PTS | 0 | 0 | 0 | 0 | -1.53 |
| *NA* | lmo0373 | similar to PTS betaglucoside-specific enzyme IIC component | PTS | 0 | 0 | 0 | 0 | 0.73 |
| *NA* | lmo0901 | similar to PTS system, cellobiose-specific IIC component | PTS | 0 | 0 | 0 | 0 | 0.39 |
| *NA* | lmo2684 | similar to cellobiose phosphotransferase enzyme IIC component | PTS | 0 | 0 | 0 | 0 | 0.35 |
| *NA* | lmo2708 | similar to PTS system, cellobiose-specific enzyme IIC | PTS | 0 | 0 | 0 | 0 | -1.37 |
| *NA* | lmo2763 | similar to PTS cellobiose-specific enzyme IIC | PTS | 0 | 0 | 0 | 0 | -0.16 |
| **Cell envelope** | | |  |  |  |  |  |  |
| *NA* | lmo0695 | unknown | Other | 2.31 | 0 | 0 | -2.1 | 1.68 |
| *NA* | lmo1382 | unknown | Biosynthesis and degradation of murein sacculus and peptidoglycan | 1.79 | 0 | 0 | 0 | 2.65 |
| *NA* | lmo0789 | similar to conserved hypothetical proteins | Biosynthesis and degradation of surface polysaccharides and lipopolysaccharides | 1.77 | 0 | 0 | 0 | 1.01 |
| *murE* | lmo2038 | similar to UDP-N-acetylmuramoylalanyl-D-glutamate-2,6-diaminopimelate ligase | Biosynthesis and degradation of murein sacculus and peptidoglycan | 1.65 | 0 | 0 | 0 | -2.35 |
| *NA* | lmo1712 | similar to multidrug resistance protein, integral membrane protein | Other | 1.5 | 0 | 0 | 0 | 1.98 |
| *pbpB* | lmo2039 | similar to penicillin-binding protein 2B | Biosynthesis and degradation of murein sacculus and peptidoglycan | 1.49 | DOWN | 0 | 0 | 1.09 |
| *murG* | lmo2035 | similar to peptidoglycan synthesis enzymes, putative phospho-N-acetylmuramoyl-pentapeptide-transferase | Biosynthesis and degradation of murein sacculus and peptidoglycan | 1.42 | 0 | 0 | 0 | -0.67 |
| *NA* | lmo1081 | similar to glucose-1-phosphate thymidyl transferase | Biosynthesis and degradation of surface polysaccharides and lipopolysaccharides | 1.36 | 0 | 0 | 0 | -1.3 |
| *NA* | lmo1695 | similar to putative membrane proteins | Other | 1.35 | 0 | 0 | 0 | 2.09 |
| *mreC* | lmo1547 | similar to cell-shape determining protein MreC | Biosynthesis and degradation of murein sacculus and peptidoglycan | 1.25 | DOWN | 0 | 0 | 0.55 |
| *NA* | lmo2691 | similar to autolysin, N-acetylmuramidase | Biosynthesis and degradation of murein sacculus and peptidoglycan | -3.23 | DOWN | 0 | -2.2 | -0.41 |
| *NA* | LMOf6854_1250 | membrane protein, putative | Other | -3.05 | 0 | 0 | 0 | 0 |
| *NA* | LMOf2365_2498 | mbl protein | Biosynthesis and degradation of murein sacculus and peptidoglycan | -2.7 | 0 | 0 | 0 | 0 |
| *NA* | lmo1076 | similar to autolysin (EC 3.5.1.28) (N-acetylmuramoyl-L-alanine amidase) | Biosynthesis and degradation of murein sacculus and peptidoglycan | -2.68 | DOWN | 0 | 0 | -0.13 |
| *NA* | lmo2130 | similar to unknown protein | Other | -2.54 | UP | 0 | 0 | -0.48 |
| *NA* | lmo0688 | similar to unknown protein | Biosynthesis and degradation of surface polysaccharides and lipopolysaccharides | -2.53 | 0 | 2.77 | -3.3 | 0.25 |
| *NA* | lmo0685 | similar to motility protein (flagellar motor rotation) MotA | Surface structures | -2.5 | 0 | 3.11 | 0 | 1.39 |
| *NA* | lmo2637 | conserved lipoprotein | Other | -2.48 | 0 | 0 | 0 | -1.37 |
| *NA* | LMOf2365_0634 | putative membrane protein | Other | -2.33 | 0 | 0 | 0 | 0 |
| *gtcA* | lmo2549 | wall teichoic acid glycosylation protein GtcA | Biosynthesis and degradation of surface polysaccharides and lipopolysaccharides | -2.33 | 0 | 0 | 0 | 0.57 |
| *NA* | lmo0415 | similar to endo-1,4-beta-xylanase | Biosynthesis and degradation of murein sacculus and peptidoglycan | -2.31 | DOWN | 0 | -3.4 | 1.51 |
| *NA* | LMOf2365_1219 | putative membrane protein | Other | -2.31 | 0 | 0 | 0 | 0 |
| *mbl* | lmo2525 | similar to MreB-like protein | Biosynthesis and degradation of murein sacculus and peptidoglycan | -2.26 | 0 | 0 | -2 | 1.73 |
| *NA* | LMOh7858_2554 | membrane protein, putative | Other | -2.23 | 0 | 0 | 0 | 0 |
| *murF* | lmo0856 | UDP-N-acetylmuramoylalanyl-D-glutamyl-2,6-diamino pimelate-D-alanyl-D-alanyl ligase | Biosynthesis and degradation of murein sacculus and peptidoglycan | -2.07 | 0 | 0 | 0 | 0.06 |
| *NA* | lmo1521 | similar to N-acetylmuramoyl-L-alanine amidase | Biosynthesis and degradation of murein sacculus and peptidoglycan | -2.05 | 0 | 0 | 0 | -0.65 |
| *NA* | lmo1855 | similar to similar to D-alanyl-D-alanine carboxypeptidases | Biosynthesis and degradation of murein sacculus and peptidoglycan | -1.99 | DOWN | 0 | 0 | -1.89 |
| *murI* | LMOf6854_1277 | glutamate racemase | Biosynthesis and degradation of murein sacculus and peptidoglycan | -1.93 | 0 | 0 | 0 | 0 |
| *ddlA* | lmo0855 | D-alanine--D-alanine ligase | Biosynthesis and degradation of murein sacculus and peptidoglycan | -1.88 | 0 | 0 | 0 | -2.32 |
| *NA* | lmo0497 | similar to sugar transferase | Biosynthesis and degradation of surface polysaccharides and lipopolysaccharides | -1.78 | 0 | 0 | 0 | 1.05 |
| *NA* | lmo0077 | conserved hypothetical protein | Biosynthesis and degradation of murein sacculus and peptidoglycan | -1.76 | 0 | 0 | 0 | 1.43 |
| *NA* | lmo1079 | similar to B. subtilis YfhO protein | Other | -1.71 | 0 | 0 | 0 | 0.24 |
| *NA* | LMOf6854_1679 | polysaccharide biosynthesis family protein | Biosynthesis and degradation of surface polysaccharides and lipopolysaccharides | -1.65 | 0 | 0 | 0 | 0 |
| *NA* | LMOf2365_1738 | cell shape-determining protein | Biosynthesis and degradation of murein sacculus and peptidoglycan | -1.51 | 0 | 0 | 0 | 0 |
| *NA* | LMOh7858_2016 | Fibronectin type III domain protein | Biosynthesis and degradation of surface polysaccharides and lipopolysaccharides | -1.49 | 0 | 0 | 0 | 0 |
| *NA* | LMOh7858_2016 | Fibronectin type III domain protein | Other | -1.49 | 0 | 0 | 0 | 0 |
| *NA* | LMOh7858_1514 | membrane protein, putative | Other | -1.49 | 0 | 0 | 0 | 0 |
| *NA* | lmo0908 | similar to membrane proteins | Other | -1.46 | 0 | 0 | 0 | -0.52 |
| *NA* | lmo1527 | similar to protein-export membrane protein SecDF | Other | -1.45 | DOWN | 0 | 0 | 0.65 |
| *NA* | lmo2854 | highly similar to B. subtilis SpoIIIJ protein | Other | -1.41 | 0 | 0 | 0 | 0 |
| *NA* | lmo0518 | similar to unknown protein | Other | -1.41 | 0 | 2.19 | 0 | 1.24 |
| *NA* | LMOh7858_1793 | adhesion lipoprotein | Other | -1.36 | 0 | 0 | 0 | 0 |
| *NA* | lmo2369 | similar to B. subtilis general stress protein 13 containing a ribosomal S1 protein domain | Biosynthesis and degradation of murein sacculus and peptidoglycan | -1.33 | 0 | 0 | 0 | -0.03 |
| *NA* | lmo0959 | similar to undacaprenyl-phosphate N-acetylglucosaminyltransferase | Biosynthesis and degradation of surface polysaccharides and lipopolysaccharides | -1.31 | 0 | 0 | 0 | -0.44 |
| *NA* | lmo1019 | similar to B. subtilis YitL protein | Biosynthesis and degradation of murein sacculus and peptidoglycan | -1.3 | 0 | 0 | 0 | 1.09 |
| *murA* | lmo2526 | UDP-N-acetylglucosamine 1-carboxyvinyltransferase | Biosynthesis and degradation of murein sacculus and peptidoglycan | -1.24 | 0 | 0 | 0 | -0.23 |
| *NA* | lmo1083 | similar to dTDP-D-glucose 4,6-dehydratase | Biosynthesis and degradation of surface polysaccharides and lipopolysaccharides | -1.22 | DOWN | 0 | 0 | -0.5 |
| *NA* | lmo0584 | conserved hypothetical membrane protein | Other | 0 | UP | 0 | 0 | 0.86 |
| *NA* | lmo0610 | similar to internalin proteins, putative peptidoglycan bound protein (LPXTG motif) | Other | 0 | UP | 0 | 1.6 | -0.15 |
| *NA* | lmo0880 | similar to wall associated protein precursor (LPXTG motif) | Other | 0 | UP | 0 | 12.3 | -0.69 |
| *NA* | lmo1413 | putative peptidoglycan bound protein (LPXTG motif) | Other | 0 | UP | 0 | 0 | 0.15 |
| *dnaJ* | lmo1472 | heat shock protein DnaJ | Surface structures | 0 | UP | -2.44 | 0 | 0.05 |
| *NA* | lmo2463 | similar to transport protein | Other | 0 | UP | 0 | 1.7 | 1.08 |
| *NA* | lmo2484 | similar to B. subtilis YvlD protein | Other | 0 | UP | 0 | 6.2 | 0.35 |
| *NA* | lmo2590 | similar to ATP binding proteins | Biosynthesis and degradation of surface polysaccharides and lipopolysaccharides | 0 | UP | 0 | 0 | 0.45 |
| *NA* | lmo1084 | similar to DTDP-L-rhamnose synthetase | Biosynthesis and degradation of surface polysaccharides and lipopolysaccharides | 0 | DOWN | 0 | 0 | -0.97 |
| *tagD* | lmo1089 | highly similar to glycerol-3-phosphate cytidylyltransferase (gct), CDP-glycerol pyrophosphorylase (teichoic acid biosynthesis protein D) | Other | 0 | DOWN | 0 | 0 | -0.22 |
| *NA* | lmo1090 | similar to glycosyltransferases | Biosynthesis and degradation of surface polysaccharides and lipopolysaccharides | 0 | DOWN | 0 | 0 | -0.96 |
| *racE* | lmo1237 | similar to glutamate racemase | Biosynthesis and degradation of murein sacculus and peptidoglycan | 0 | DOWN | -2.14 | 0 | 0.17 |
| *NA* | lmo1420 | weakly similar to UDP-N-acetylglucosaminyl-3-enolpyruvate reductase | Biosynthesis and degradation of murein sacculus and peptidoglycan | 0 | DOWN | 0 | 0 | 0.33 |
| *mreD* | lmo1546 | similar to cell-shape determining protein MreD | Biosynthesis and degradation of murein sacculus and peptidoglycan | 0 | DOWN | 0 | 0 | 1.53 |
| *mreB* | lmo1548 | similar to cell-shape determining protein MreB | Biosynthesis and degradation of murein sacculus and peptidoglycan | 0 | DOWN | 0 | -2.2 | 1.14 |
| *NA* | lmo1604 | similar to 2-cys peroxiredoxin | Other | 0 | DOWN | -2.41 | -2.1 | 1.48 |
| *NA* | lmo2537 | similar to UDP-N-acetylglucosamine 2-epimerase | Biosynthesis and degradation of surface polysaccharides and lipopolysaccharides | 0 | DOWN | 0 | 0 | -1.01 |
| *NA* | lmo2636 | conserved hypothetical lipoprotein | Biosynthesis and degradation of surface polysaccharides and lipopolysaccharides | 0 | DOWN | 0 | 0 | 0.45 |
| *NA* | lmo2754 | similar to D-alanyl-D-alanine carboxypeptidase (penicillin-binding protein 5) | Biosynthesis and degradation of murein sacculus and peptidoglycan | 0 | DOWN | 0 | 0 | 0.7 |
| *NA* | lmo0129 | similar to autolysin: N-acetylmuramoyl-L-alanine amidase | Biosynthesis and degradation of murein sacculus and peptidoglycan | 0 | 0 | 1.27 | 0 | -0.76 |
| *NA* | lmo0540 | similar to penicillin-binding protein | Biosynthesis and degradation of murein sacculus and peptidoglycan | 0 | 0 | 2.34 | 0 | -0.16 |
| *NA* | lmo1713 | similar to cell-shape determining proteins | Biosynthesis and degradation of murein sacculus and peptidoglycan | 0 | 0 | 2.48 | 0 | 1.83 |
| *NA* | lmo0495 | similar to transmembrane protein | Other | 0 | 0 | 0 | 2 | 3.61 |
| *NA* | lmo0481 | similar to unknown proteins | Biosynthesis and degradation of surface polysaccharides and lipopolysaccharides | 0 | 0 | 0 | 1.4 | 0.35 |
| *NA* | lmo0441 | similar to penicillin-binding protein (D-alanyl-D-alanine carboxypeptidase) | Biosynthesis and degradation of murein sacculus and peptidoglycan | 0 | 0 | 0 | -2.1 | 0.35 |
| *NA* | lmo1216 | similar to N-acetylmuramoyl-L-alanine amidase (autolysin) | Biosynthesis and degradation of murein sacculus and peptidoglycan | 0 | 0 | 0 | -2.4 | 1.83 |
| *NA* | lmo0366 | conserved hypothetical protein, putative lipoprotein | Other | 0 | 0 | 0 | -3.7 | 1.23 |
| *NA* | lmo1285 | conserved hypothetical protein, similar to B. subtilis YneT protein | Biosynthesis and degradation of murein sacculus and peptidoglycan | 0 | 0 | 0 | -2.1 | -0.67 |
| *NA* | lmo0153 | similar to a probable high-affinity zinc ABC transporter (Zn(II)-binding lipoprotein) | Surface structures | 0 | 0 | 0 | 0 | -1.32 |
| *NA* | lmo0153 | similar to a probable high-affinity zinc ABC transporter (Zn(II)-binding lipoprotein) | Other | 0 | 0 | 0 | 0 | -1.32 |
| *NA* | lmo0159 | putative peptidoglycan bound protein (LPXTG motif) | Other | 0 | 0 | 0 | 0 | -1.51 |
| *NA* | lmo0195 | similar to membrane protein (putative ABC transporter component) | Other | 0 | 0 | 0 | 0 | -2.7 |
| *gcaD* | lmo0198 | highly similar to UDP-N-acetylglucosamine pyrophosphorylase | Biosynthesis and degradation of surface polysaccharides and lipopolysaccharides | 0 | 0 | 0 | 0 | 0.57 |
| *NA* | lmo0320 | similar to surface protein (peptidoglycan bound, LPXTG motif) | Surface structures | 0 | 0 | 0 | 0 | 0.46 |
| *NA* | lmo0320 | similar to surface protein (peptidoglycan bound, LPXTG motif) | Other | 0 | 0 | 0 | 0 | 0.46 |
| *NA* | lmo0463 | unknown | Other | 0 | 0 | 0 | 0 | 0.1 |
| *NA* | lmo0514 | similar to internalin protein, putative peptidoglycan bound protein (LPXTG motif) | Other | 0 | 0 | 0 | 0 | 1.57 |
| *NA* | lmo0529 | conserved hypothetical protein similar to putative glucosaminyltransferase | Biosynthesis and degradation of surface polysaccharides and lipopolysaccharides | 0 | 0 | 0 | 0 | 0.28 |
| *NA* | lmo0550 | pepdidoglycan bound protein (LPXTG motif) | Other | 0 | 0 | 0 | 0 | 1.2 |
| *NA* | lmo0577 | unknown | Other | 0 | 0 | 0 | 0 | 0.01 |
| *NA* | lmo0603 | unknown | Other | 0 | 0 | 0 | 0 | 1.75 |
| *NA* | lmo0644 | similar to conserved hypothetical proteins | Other | 0 | 0 | 0 | 0 | -0.69 |
| *dal* | lmo0886 | similar to alanine racemase | Biosynthesis and degradation of murein sacculus and peptidoglycan | 0 | 0 | 0 | 0 | -0.05 |
| *NA* | lmo0898 | conserved hypothetical protein | Biosynthesis and degradation of murein sacculus and peptidoglycan | 0 | 0 | 0 | 0 | 0.67 |
| *NA* | lmo0927 | hypothetical transmembrane protein | Other | 0 | 0 | 0 | 0 | -0.84 |
| *dltD* | lmo0971 | DltD protein for D-alanine esterification of lipoteichoic acid and wall teichoic acid | Other | 0 | 0 | 0 | 0 | -0.67 |
| *dltC* | lmo0972 | D-alanyl carrier protein | Biosynthesis and degradation of murein sacculus and peptidoglycan | 0 | 0 | 0 | 0 | -0.36 |
| *dltB* | lmo0973 | DltB protein for D-alanine esterification of lipoteichoic acid and wall teichoic acid | Other | 0 | 0 | 0 | 0 | -0.58 |
| *NA* | lmo1044 | similar to molybdopterin converting factor, subunit 2 | Biosynthesis and degradation of surface polysaccharides and lipopolysaccharides | 0 | 0 | 0 | 0 | -1.42 |
| *NA* | lmo1062 | similar to ABC transporters (permease protein) | Other | 0 | 0 | 0 | 0 | 1.62 |
| *NA* | lmo1078 | similar to putative UDP-glucose pyrophosphorylases | Biosynthesis and degradation of surface polysaccharides and lipopolysaccharides | 0 | 0 | 0 | 0 | 0.23 |
| *NA* | lmo1080 | similar to B. subtilis minor teichoic acids biosynthesis protein GgaB | Biosynthesis and degradation of surface polysaccharides and lipopolysaccharides | 0 | 0 | 0 | 0 | 0 |
| *NA* | lmo1082 | similar to dTDP-sugar epimerase | Biosynthesis and degradation of surface polysaccharides and lipopolysaccharides | 0 | 0 | 0 | 0 | -1.89 |
| *NA* | lmo1091 | similar to glysosyltransferases | Biosynthesis and degradation of surface polysaccharides and lipopolysaccharides | 0 | 0 | 0 | 0 | -0.43 |
| *NA* | lmo1215 | similar to N-acetylmuramoyl-L-alanine amidase (autolysin) | Biosynthesis and degradation of murein sacculus and peptidoglycan | 0 | 0 | 0 | 0 | 0.41 |
| *NA* | lmo1226 | similar to transporter, (to B. subtilis YdgH protein) | Other | 0 | 0 | 0 | 0 | -0.52 |
| *NA* | lmo1290 | similar to internalin proteins, putative peptidoglycan bound protein (LPXTG motif) | Other | 0 | 0 | 0 | 0 | 0.6 |
| *NA* | lmo1379 | similar to B. subtilis SpoIIIJ protein | Other | 0 | 0 | 0 | 0 | -0.02 |
| *NA* | lmo1438 | similar to penicillin-binding protein | Biosynthesis and degradation of murein sacculus and peptidoglycan | 0 | 0 | 0 | 0 | 1.42 |
| *NA* | lmo1506 | similar to transporter | Other | 0 | 0 | 0 | 0 | -0.64 |
| *murC* | lmo1605 | similar to UDP-N-acetyl muramate-alanine ligases | Biosynthesis and degradation of murein sacculus and peptidoglycan | 0 | 0 | 0 | 0 | 0.24 |
| *pheT* | lmo1607 | similar phenylalanyl-tRNA synthetase (beta subunit) | Biosynthesis and degradation of murein sacculus and peptidoglycan | 0 | 0 | 0 | 0 | -0.9 |
| *NA* | lmo1647 | similar to 1-acylglycerol-3-phosphate O-acyltransferases | Biosynthesis and degradation of surface polysaccharides and lipopolysaccharides | 0 | 0 | 0 | 0 | 0.72 |
| *NA* | lmo1671 | similar to ABC transporter and adhesion proteins | Surface structures | 0 | 0 | 0 | 0 | 0.11 |
| *NA* | lmo1671 | similar to ABC transporter and adhesion proteins | Other | 0 | 0 | 0 | 0 | 0.11 |
| *NA* | lmo1799 | putative peptidoglycan bound protein (LPXTG motif) | Other | 0 | 0 | 0 | 0 | -1.18 |
| *NA* | lmo1829 | similar to fibronectin binding proteins | Surface structures | 0 | 0 | 0 | 0 | -4.56 |
| *pbpA* | lmo1892 | similar to penicillin-binding protein 2A | Biosynthesis and degradation of murein sacculus and peptidoglycan | 0 | 0 | 0 | 0 | 1.88 |
| *murD* | lmo2036 | similar to UDP-N-acetylmuramoylalanine D-glutamate ligase | Biosynthesis and degradation of murein sacculus and peptidoglycan | 0 | 0 | 0 | 0 | 0.84 |
| *mraY* | lmo2037 | similar to phospho-N-acetylmuramoyl-pentapeptide transferase | Biosynthesis and degradation of murein sacculus and peptidoglycan | 0 | 0 | 0 | 0 | -0.55 |
| *NA* | lmo2043 | similar to integral membrane proteins | Other | 0 | 0 | 0 | 0 | 2.26 |
| *NA* | lmo2052 | similar to phosphopantetheine adenylyltransferase | Biosynthesis and degradation of surface polysaccharides and lipopolysaccharides | 0 | 0 | 0 | 0 | -1.21 |
| *NA* | lmo2147 | similar to unknown proteins | Other | 0 | 0 | 0 | 0 | -1.39 |
| *NA* | lmo2203 | similar to N-acetylmuramoyl-L-alanine amidase and to internalin B | Biosynthesis and degradation of murein sacculus and peptidoglycan | 0 | 0 | 0 | 0 | -0.78 |
| *NA* | lmo2229 | similar to penicillin-binding protein | Biosynthesis and degradation of murein sacculus and peptidoglycan | 0 | 0 | 0 | 0 | -0.95 |
| *lysA* | lmo2278 | L-alanoyl-D-glutamate peptidase | Biosynthesis and degradation of murein sacculus and peptidoglycan | 0 | 0 | 0 | 0 | -0.37 |
| *NA* | lmo2291 | major tail shaft protein [Bacteriophage A118] | Other | 0 | 0 | 0 | 0 | -0.41 |
| *NA* | lmo2312 | unknown | Biosynthesis and degradation of murein sacculus and peptidoglycan | 0 | 0 | 0 | 0 | 0.79 |
| *NA* | lmo2519 | similar to B. subtilis TagO teichoic acid linkage unit synthesis protein | Biosynthesis and degradation of surface polysaccharides and lipopolysaccharides | 0 | 0 | 0 | 0 | 2.3 |
| *NA* | lmo2521 | similar to B. subtilis TagA protein involved in polyglycerol phosphate biosynthesis | Other | 0 | 0 | 0 | 0 | 0.59 |
| *murZ* | lmo2552 | highly similar to UDP-N-acetylglucosamine 1-carboxyvinyltransferase | Biosynthesis and degradation of murein sacculus and peptidoglycan | 0 | 0 | 0 | 0 | -0.46 |
| *NA* | lmo2554 | similar to galactosyltransferase | Biosynthesis and degradation of surface polysaccharides and lipopolysaccharides | 0 | 0 | 0 | 0 | 0.13 |
| *NA* | lmo2576 | peptidoglycan anchored protein (LPXTG motif) | Other | 0 | 0 | 0 | 0 | -0.8 |
| *NA* | lmo2812 | similar to D-alanyl-D-alanine carboxypeptidase | Biosynthesis and degradation of murein sacculus and peptidoglycan | 0 | 0 | 0 | 0 | -1.75 |
| **Cellular processes** | | |  |  |  |  |  |  |
| *NA* | lmo2673 | conserved hypothetical protein | Adaptations to atypical conditions | 5.38 | UP | 0 | 7.4 | -2.37 |
| *NA* | lmo2230 | similar to arsenate reductase | Detoxification | 5.04 | UP | 2.3 | 33.9 | -1.16 |
| *NA* | lmo2230 | similar to arsenate reductase | Toxin production and resistance | 5.04 | UP | 2.3 | 33.9 | -1.16 |
| *NA* | LMOf6854_2028 | tellurite resistance protein, putative | Toxin production and resistance | 2.99 | 0 | 0 | 0 | 0 |
| *NA* | lmo0515 | conserved hypothetical protein | Adaptations to atypical conditions | 2.72 | UP | 0 | 2.6 | -0.52 |
| *mecA* | lmo2190 | competence negative regulator mecA | DNA transformation | 2.69 | UP | 0 | 0 | -1.36 |
| *NA* | LMOh7858_0844 | phenazine biosynthesis protein, PhzF family | Toxin production and resistance | 2.36 | 0 | 0 | 0 | 0 |
| *NA* | LMOf2365_1997 | putative tellurite resistance protein | Toxin production and resistance | 2.32 | 0 | 0 | 0 | 0 |
| *kaT* | LMOf6854_2903 | catalase | Detoxification | 2.21 | 0 | 0 | 0 | 0 |
| *fliM* | LMOf2365_0735 | flagellar motor switch protein FliM | Chemotaxis and motility | 2.2 | 0 | 0 | 0 | 0 |
| *NA* | lmo2827 | similar to transcriptional regulator (MarR family) | Other | 2.15 | UP | 0 | 0 | 1.92 |
| *NA* | lmo0152 | similar to oligopeptide ABC transporter-binding protein | DNA transformation | 2.06 | 0 | -6.2 | 0 | -1.81 |
| *NA* | lmo0215 | conserved membrane-spanning protein | Adaptations to atypical conditions | 2.04 | 0 | 0 | 0 | 0.77 |
| *sugE-2* | LMOf2365_0871 | sugE protein | Toxin production and resistance | 1.84 | 0 | 0 | 0 | 0 |
| *NA* | lmo1638 | similar to unknown proteins | Toxin production and resistance | 1.84 | 0 | 0 | 0 | 0.58 |
| *NA* | lmo1580 | similar to unknown protein | Adaptations to atypical conditions | 1.82 | UP | 0 | 1.9 | 1.02 |
| *NA* | lmo0854 | similar to E. coli SugE protein (transmembrane chaperone) | Toxin production and resistance | 1.82 | 0 | 0 | 0 | -0.61 |
| *cheA* | lmo0692 | two-component sensor histidine kinase CheA | Chemotaxis and motility | 1.78 | 0 | 0 | 0 | 1.99 |
| *NA* | LMOh7858_0763 | flagellar motor switch protein, putative | Chemotaxis and motility | 1.77 | 0 | 0 | 0 | 0 |
| *fliD* | LMOf2365_0743 | flagellar hook-associated protein 2 | Chemotaxis and motility | 1.7 | 0 | 0 | 0 | 0 |
| *NA* | lmo2738 | conserved hypothetical protein similar to hypothetical hemolysin | Toxin production and resistance | 1.64 | 0 | 0 | 0 | 1.32 |
| *NA* | lmo0669 | similar to oxidoreductase | Adaptations to atypical conditions | 1.59 | UP | 0 | 34.4 | -4.02 |
| *NA* | lmo0711 | similar to flagellar basal-body rod protein flgC | Chemotaxis and motility | 1.54 | 0 | 0 | -2.7 | -0.03 |
| *NA* | LMOf2365_2066 | cell division protein FtsQ | Cell division | 1.51 | 0 | 0 | 0 | 0 |
| *NA* | lmo0698 | weakly similar to flagellar switch protein | Chemotaxis and motility | 1.47 | 0 | 0 | -2.1 | 0.94 |
| *NA* | lmo0234 | highly similar to B. subtilis YacL protein | DNA transformation | 1.4 | 0 | 0 | 0 | 0.74 |
| *NA* | lmo0693 | similar to flagellar motor switch protein fliY C-terminal part | Chemotaxis and motility | 1.39 | 0 | 0 | 0 | 2.72 |
| *NA* | lmo0723 | similar to metyl-accepting chemotaxis protein | Toxin production and resistance | 1.38 | UP | 0 | 0 | 1.61 |
| *NA* | lmo1366 | conserved hypothetical protein | Toxin production and resistance | 1.37 | 0 | 0 | 0 | 1.7 |
| *flgB* | LMOf6854_0757 | flagellar basal-body rod protein FlgB | Chemotaxis and motility | 1.26 | 0 | 0 | 0 | 0 |
| *NA* | lmo0706 | similar to flagellar hook-associated protein 3 FlgL | Chemotaxis and motility | 1.26 | 0 | 0 | -2 | 0.45 |
| *iap* | LMOf2365_0611 | protein P60 | Pathogenesis | -4.49 | 0 | 0 | 0 | 0 |
| *ftsE* | lmo2507 | highly similar to the cell-division ATP-binding protein FtsE | Cell division | -4.06 | DOWN | 0 | 0 | -0.27 |
| *NA* | lmo1700 | unknown | Toxin production and resistance | -3.53 | 0 | 0 | -5.3 | 0.7 |
| *iap* | LMOf6854_0623 | protein P60 | Pathogenesis | -3.25 | 0 | 0 | 0 | 0 |
| *NA* | lmo2196 | similar to pheromone ABC transporter (binding protein) | DNA transformation | -3.23 | DOWN | -2.17 | 0 | -0.5 |
| *cspB* | lmo2016 | similar to major cold-shock protein | Adaptations to atypical conditions | -3.04 | DOWN | -36.11 | 0 | -0.08 |
| *ftsX* | lmo2506 | highly similar to cell-division protein FtsX | Cell division | -2.9 | DOWN | 0 | 0 | 1.05 |
| *NA* | lmo1699 | some similarities to methyl-accepting chemotaxis proteins | Chemotaxis and motility | -2.85 | 0 | 0 | -4.7 | 1.63 |
| *NA* | LMOf2365_1002 | drug resistance transporter, EmrB/QacA family | Toxin production and resistance | -2.52 | 0 | 0 | 0 | 0 |
| *NA* | lmo2569 | similar to dipeptide ABC transporter (dipeptide-binding protein) | DNA transformation | -2.51 | 0 | 0 | 0 | -0.34 |
| *NA* | lmo0685 | similar to motility protein (flagellar motor rotation) MotA | Chemotaxis and motility | -2.5 | 0 | 3.11 | 0 | 1.39 |
| *NA* | LMOf2365_0548 | drug resistance transporter, EmrB/QacA family | Toxin production and resistance | -2.36 | 0 | 0 | 0 | 0 |
| *NA* | lmo0683 | similar to chemotactic methyltransferase CheR | Chemotaxis and motility | -2.23 | 0 | 3.48 | 0 | 3.28 |
| *NA* | LMOf6854_0560 | drug resistance transporter, EmrB/QacA family | Toxin production and resistance | -2.22 | 0 | 0 | 0 | 0 |
| *lmrB* | LMOf6854_0559 | drug resistance transporter, EmrB/QacA subfamily | Toxin production and resistance | -2.16 | 0 | 0 | 0 | 0 |
| *NA* | lmo0689 | similar to CheA activity-modulating chemotaxis protein CheV | Chemotaxis and motility | -2.1 | UP | 2.03 | 0 | -2.99 |
| *NA* | lmo1625 | similar to putative transporters | Adaptations to atypical conditions | -2.06 | DOWN | -1.5 | 0 | -1.48 |
| *NA* | LMOf6854_1767 | aminoglycoside N3-acetyltransferase | Toxin production and resistance | -1.92 | 0 | 0 | 0 | 0 |
| *NA* | lmo0682 | similar to flagellar hook-basal body protein FlgG | Chemotaxis and motility | -1.91 | 0 | 2.71 | 0 | 1.96 |
| *NA* | LMOf2365_1723 | methyl-accepting chemotaxis protein | Chemotaxis and motility | -1.9 | 0 | 0 | 0 | 0 |
| *NA* | LMOf2365_1628 | FtsK/SpoIIIE family protein | Cell division | -1.89 | 0 | 0 | 0 | 0 |
| *NA* | lmo0676 | similar to flagellar biosynthesic protein FliP | Chemotaxis and motility | -1.78 | UP | 1.83 | 0 | -0.1 |
| *NA* | lmo0497 | similar to sugar transferase | Toxin production and resistance | -1.78 | 0 | 0 | 0 | 1.05 |
| *NA* | lmo0678 | similar to flagellar biosynthetic protein FliR | Toxin production and resistance | -1.77 | 0 | 1.88 | 0 | 0.8 |
| *NA* | lmo0333 | similar to internalin proteins, putative peptidoglycan bound protein (LPXTG motif) | Pathogenesis | -1.73 | 0 | 0 | 0 | -0.51 |
| *fliP* | LMOf2365_0712 | flagellar biosynthesis protein FliP | Chemotaxis and motility | -1.68 | 0 | 0 | 0 | 0 |
| *NA* | lmo0677 | similar to flagellar biosynthesis protein FliQ | Chemotaxis and motility | -1.66 | UP | 3.2 | 0 | 0.35 |
| *NA* | lmo1543 | similar to ribonuclease G | Cell division | -1.62 | 0 | 0 | 0 | -1.52 |
| *NA* | lmo1624 | similar to putative transporters | Adaptations to atypical conditions | -1.54 | 0 | 0 | 0 | 0.73 |
| *NA* | lmo0679 | similar to flagellar biosynthetic protein flhB | Chemotaxis and motility | -1.5 | 0 | 3.67 | 0 | 1.1 |
| *NA* | lmo2080 | unknown | Chemotaxis and motility | -1.5 | 0 | 0 | 0 | -3.13 |
| *NA* | lmo0197 | similar to B. subtilis SpoVG protein | Adaptations to atypical conditions | -1.49 | 0 | 0 | 0 | -1.01 |
| *NA* | lmo1708 | similar to aminoglycoside N3-acetyltransferases | Toxin production and resistance | -1.49 | 0 | 0 | 0 | 1.22 |
| *NA* | lmo1071 | similar to cell-division protein RodA and FtsW | Cell division | -1.44 | DOWN | 0 | 0 | -3.28 |
| *motB* | LMOf2365_0722 | chemotaxis protein MotB | Chemotaxis and motility | -1.44 | 0 | 0 | 0 | 0 |
| *menH* | lmo1931 | similar to 2-heptaprenyl-1,4-naphthoquinone methyltransferase | Other | -1.43 | 0 | 0 | 0 | -0.26 |
| *flaA* | lmo0690 | flagellin protein | Chemotaxis and motility | -1.39 | 0 | 2.47 | 0 | 0.58 |
| *divIVA* | lmo2020 | similar to cell-division initiation protein (septum placement) | Cell division | -1.36 | DOWN | 0 | 0 | -5.06 |
| *NA* | LMOh7858_1793 | adhesion lipoprotein | Cell adhesion | -1.36 | 0 | 0 | 0 | 0 |
| *ftsL* | lmo2040 | similar to cell-division protein FtsL | Cell division | -1.31 | 0 | 0 | 0 | -0.57 |
| *plcA* | lmo0201 | phosphatidylinositol-specific phospholipase c | Toxin production and resistance | 0 | UP | -8.34 | 0 | -1.13 |
| *inlA* | lmo0433 | Internalin A | Pathogenesis | 0 | UP | 0 | 2 | 0.24 |
| *inlB* | lmo0434 | Internalin B | Pathogenesis | 0 | UP | 0 | 2 | -0.1 |
| *inlH* | lmo0263 | internalin H | Pathogenesis | 0 | UP | 0 | 6.7 | -0.56 |
| *NA* | lmo0291 | conserved hypothetical protein similar to B. subtilis YycJ protein | Toxin production and resistance | 0 | UP | 0 | 2 | 1.52 |
| *NA* | lmo1300 | similar to arsenic efflux pump protein | Detoxification | 0 | UP | -2.42 | 0 | 1.95 |
| *comGB* | lmo1346 | similar to B. subtilis comG operon protein 2 | Toxin production and resistance | 0 | UP | 0 | 0 | 0.22 |
| *NA* | lmo1433 | similar to glutathione reductase | Detoxification | 0 | UP | 0 | 2.7 | -0.14 |
| *comEA* | lmo1484 | similar to integral membrane protein ComEA | DNA transformation | 0 | UP | 0 | 0 | -0.08 |
| *NA* | lmo1601 | similar to general stress protein | Adaptations to atypical conditions | 0 | UP | 0 | 2.5 | -0.47 |
| *NA* | lmo2084 | unknown | Toxin production and resistance | 0 | UP | 0 | 0 | 0.58 |
| *NA* | lmo2189 | similar to a putative competence protein from streptococcus pneumoniae | DNA transformation | 0 | UP | 0 | 0 | -1.95 |
| *NA* | lmo2232 | similar to unknown proteins | Toxin production and resistance | 0 | UP | 0 | 4.9 | -0.14 |
| *NA* | lmo2273 | protein gp30 [Bacteriophage A118] | Detoxification | 0 | UP | 0 | 0 | -0.88 |
| *NA* | lmo2274 | protein gp29 [Bacteriophage A118] | Detoxification | 0 | UP | 0 | 0 | -2.32 |
| *ltrC* | lmo2398 | low temperature requirement C protein, also similar to B. subtilis YutG protein | Adaptations to atypical conditions | 0 | UP | 0 | 7 | -0.96 |
| *NA* | lmo0186 | similar to B. subtilis YabE protein | Detoxification | 0 | DOWN | 0 | 0 | -1.27 |
| *ksgA* | lmo0188 | dimethyladenosine transferase (16S rRNA dimethylase) | Toxin production and resistance | 0 | DOWN | 0 | 0 | -1.25 |
| *cspL* | lmo1364 | similar to cold shock protein | Adaptations to atypical conditions | 0 | DOWN | 7.14 | -2 | 1.09 |
| *NA* | lmo1400 | similar to N-acetyltransferase | Toxin production and resistance | 0 | DOWN | 0 | 0 | 1.45 |
| *minD* | lmo1544 | highly similar to cell division inhibitor (septum placement) protein MinD | Cell division | 0 | DOWN | 0 | 0 | 0.92 |
| *minC* | lmo1545 | similar to cell-division inhibition (septum placement) protein MinC | Cell division | 0 | DOWN | 0 | 0 | 2 |
| *NA* | lmo1614 | similar to unknown proteins | Toxin production and resistance | 0 | DOWN | 0 | 0 | 0.33 |
| *NA* | lmo0321 | similar to unknown proteins | Toxin production and resistance | 0 | 0 | 3.28 | 3 | -0.51 |
| *motB* | lmo0686 | similar to motility protein (flagellar motor rotation) MotB | Chemotaxis and motility | 0 | 0 | 2.42 | 0 | -0.87 |
| *NA* | lmo1245 | unknown | Adaptations to atypical conditions | 0 | 0 | 4.71 | 0 | -0.43 |
| *plcB* | lmo0205 | phospholipase C | Toxin production and resistance | 0 | 0 | -5.32 | 0 | -0.93 |
| *NA* | lmo2064 | similar to large conductance mechanosensitive channel protein | Adaptations to atypical conditions | 0 | 0 | -2.08 | 2.2 | 0.36 |
| *cspD* | lmo1879 | similar to cold shock protein | Adaptations to atypical conditions | 0 | 0 | 0 | -2 | 0.15 |
| *NA* | lmo1694 | similar to CDP-abequose synthase | Cell division | 0 | 0 | 0 | 3.4 | 0.29 |
| *NA* | lmo0341 | unknown | Toxin production and resistance | 0 | 0 | 0 | -1.3 | 0.81 |
| *NA* | lmo1027 | similar to conserved hypothetical proteins (in particular B. subtilis YkqC) | Toxin production and resistance | 0 | 0 | 0 | 2.5 | -2.19 |
| *NA* | lmo0196 | similar to B. subtilis SpoVG protein | Adaptations to atypical conditions | 0 | 0 | 0 | 1.2 | -0.47 |
| *NA* | lmo0696 | similar to flagellar hook assembly protein | Chemotaxis and motility | 0 | 0 | 0 | -2.3 | 0.95 |
| *NA* | lmo0705 | similar to flagellar hook-associated protein FlgK | Chemotaxis and motility | 0 | 0 | 0 | -2.1 | -0.04 |
| *NA* | lmo0710 | similar to flagellar basal-body rod protein flgB | Chemotaxis and motility | 0 | 0 | 0 | -2.4 | 0.56 |
| *NA* | lmo0712 | similar to flagellar hook-basal body complex protein FliE | Chemotaxis and motility | 0 | 0 | 0 | -2.5 | -0.06 |
| *NA* | lmo0713 | similar to flagellar basal-body M-ring protein fliF | Chemotaxis and motility | 0 | 0 | 0 | -2.2 | 0.15 |
| *NA* | lmo0714 | similar to flagellar motor switch protein fliG | Chemotaxis and motility | 0 | 0 | 0 | -2.2 | -0.04 |
| *NA* | lmo0008 | similar to cardiolipin synthase | Toxin production and resistance | 0 | 0 | 0 | 0 | 0.37 |
| *NA* | lmo0028 | similar to E. coli microcin C7 self-immunity protein (MccF) | Toxin production and resistance | 0 | 0 | 0 | 0 | -0.76 |
| *NA* | lmo0061 | highly similar to B. subtilis YukA protein | Toxin production and resistance | 0 | 0 | 0 | 0 | -0.1 |
| *NA* | lmo0065 | unknown | Pathogenesis | 0 | 0 | 0 | 0 | 0.58 |
| *NA* | lmo0153 | similar to a probable high-affinity zinc ABC transporter (Zn(II)-binding lipoprotein) | Pathogenesis | 0 | 0 | 0 | 0 | -1.32 |
| *NA* | lmo0171 | similar to internalin proteins, putative peptidoglycan bound protein (LPXTG motif) | Pathogenesis | 0 | 0 | 0 | 0 | -1.83 |
| *NA* | lmo0217 | similar to B. subtilis DivIC protein | Cell division | 0 | 0 | 0 | 0 | -0.78 |
| *ftsH* | lmo0220 | highly similar to cell division protein ftsH | Cell division | 0 | 0 | 0 | 0 | 0.36 |
| *NA* | lmo0252 | similar to repressor (penicilinase repressor) | Toxin production and resistance | 0 | 0 | 0 | 0 | -0.29 |
| *inlG* | lmo0262 | internalin G | Pathogenesis | 0 | 0 | 0 | 0 | -0.56 |
| *inlE* | lmo0264 | internalin E | Pathogenesis | 0 | 0 | 0 | 0 | -1.36 |
| *NA* | lmo0275 | C-terminal part similar to B. subtilis ComEC protein | DNA transformation | 0 | 0 | 0 | 0 | 1.21 |
| *NA* | lmo0331 | similar to internalin, putative peptidoglycan bound protein (LPXTG motif) | Pathogenesis | 0 | 0 | 0 | 0 | -0.26 |
| *ltrA* | lmo0389 | low temperature requirement protein A | Adaptations to atypical conditions | 0 | 0 | 0 | 0 | 3.25 |
| *NA* | lmo0409 | similar to internalin, peptidoglycan bound protein (LPxTG motif) | Pathogenesis | 0 | 0 | 0 | 0 | 0.03 |
| *NA* | lmo0421 | similar to rod shape-determining protein RodA | Cell division | 0 | 0 | 0 | 0 | 0.04 |
| *NA* | lmo0529 | conserved hypothetical protein similar to putative glucosaminyltransferase | Toxin production and resistance | 0 | 0 | 0 | 0 | 0.28 |
| *NA* | lmo0601 | similar to cell surface protein | Pathogenesis | 0 | 0 | 0 | 0 | 1.29 |
| *cheY* | lmo0691 | Chemotaxis response regulator CheY | Chemotaxis and motility | 0 | 0 | 0 | 0 | 0.2 |
| *NA* | lmo0697 | similar to flagellar hook protein FlgE | Chemotaxis and motility | 0 | 0 | 0 | 0 | 1.07 |
| *NA* | lmo0699 | similar to flagellar switch protein FliM | Chemotaxis and motility | 0 | 0 | 0 | 0 | 0.79 |
| *NA* | lmo0700 | similar to flagellar motor switch protein fliY | Chemotaxis and motility | 0 | 0 | 0 | 0 | 0.67 |
| *NA* | lmo0707 | similar to flagellar hook-associated protein 2 FliD | Chemotaxis and motility | 0 | 0 | 0 | 0 | 0.74 |
| *NA* | lmo0708 | similar to hypothetical flagellar protein | Chemotaxis and motility | 0 | 0 | 0 | 0 | 0.27 |
| *NA* | lmo0801 | similar to internalin, putative peptidoglycan bound protein (LPXTG motif) | Pathogenesis | 0 | 0 | 0 | 0 | 0.35 |
| *NA* | lmo0839 | similar to Tetracycline resistance protein | Toxin production and resistance | 0 | 0 | 0 | 0 | -0.63 |
| *NA* | lmo0906 | similar to glutathione Reductase | Detoxification | 0 | 0 | 0 | 0 | -0.56 |
| *NA* | lmo0969 | similar to ribosomal large subunit pseudouridine synthetase | Toxin production and resistance | 0 | 0 | 0 | 0 | -1.73 |
| *NA* | lmo0983 | similar to glutathione peroxidase | Detoxification | 0 | 0 | 0 | 0 | 0.2 |
| *NA* | lmo1091 | similar to glysosyltransferases | Toxin production and resistance | 0 | 0 | 0 | 0 | -0.43 |
| *NA* | lmo1136 | similar to internalin, putative peptidoglycan bound protein (LPXTG motif) | Pathogenesis | 0 | 0 | 0 | 0 | -2.19 |
| *NA* | lmo1274 | similar to polypeptide deformylase, similar to B. subtilis Smf protein | DNA transformation | 0 | 0 | 0 | 0 | 1.14 |
| *NA* | lmo1288 | conserved hypothetical protein | Other | 0 | 0 | 0 | 0 | -0.04 |
| *NA* | lmo1297 | similar to aluminum resistance protein and to B. subtilis YnbB protein (hypothetical) | Toxin production and resistance | 0 | 0 | 0 | 0 | 0.02 |
| *NA* | lmo1309 | similar to E. coli YbdM protein | Cell division | 0 | 0 | 0 | 0 | -0.21 |
| *NA* | lmo1345 | similar to B. subtilis comG operon protein 3 | DNA transformation | 0 | 0 | 0 | 0 | 1.64 |
| *comGA* | lmo1347 | similar to B. subtilis comG operon protein 1 | DNA transformation | 0 | 0 | 0 | 0 | 0.37 |
| *NA* | lmo1386 | similar to DNA translocase | Adaptations to atypical conditions | 0 | 0 | 0 | 0 | 1.59 |
| *NA* | lmo1434 | similar to unknown proteins | Toxin production and resistance | 0 | 0 | 0 | 0 | 0.78 |
| *sod* | lmo1439 | superoxide dismutase | Detoxification | 0 | 0 | 0 | 0 | 0.13 |
| *NA* | lmo1451 | similar to E. coli LytB protein | Toxin production and resistance | 0 | 0 | 0 | 0 | -0.86 |
| *NA* | lmo1462 | similar to GTP binding proteins | Cell division | 0 | 0 | 0 | 0 | -0.76 |
| *comEC* | lmo1482 | similar to putative integral membrane protein ComEC specifically required for DNA uptake but not for binding | DNA transformation | 0 | 0 | 0 | 0 | 0.96 |
| *NA* | lmo1577 | similar to unknown proteins | Toxin production and resistance | 0 | 0 | 0 | 0 | 0.4 |
| *NA* | lmo1583 | similar to thiol peroxidases | Detoxification | 0 | 0 | 0 | 0 | 1.75 |
| *NA* | lmo1671 | similar to ABC transporter and adhesion proteins | Pathogenesis | 0 | 0 | 0 | 0 | 0.11 |
| *NA* | lmo1729 | similar to beta-glucosidases | Toxin production and resistance | 0 | 0 | 0 | 0 | -0.93 |
| *inlC* | lmo1786 | internalin C | Pathogenesis | 0 | 0 | 0 | 0 | 1.74 |
| *smc* | lmo1804 | similar to Smc protein essential for chromosome condensation and partition | Cell division | 0 | 0 | 0 | 0 | 0.77 |
| *NA* | lmo1888 | similar to hypothetical proteins | Cell division | 0 | 0 | 0 | 0 | 1.61 |
| *NA* | lmo1903 | similar to thioredoxin | Toxin production and resistance | 0 | 0 | 0 | 0 | 1.72 |
| *NA* | lmo1967 | similar to toxic ion resistance proteins | Toxin production and resistance | 0 | 0 | 0 | 0 | 2.06 |
| *NA* | lmo1977 | similar to unknown proteins | Toxin production and resistance | 0 | 0 | 0 | 0 | 0.56 |
| *ftsZ* | lmo2032 | highly similar to cell-division initiation protein FtsZ | Cell division | 0 | 0 | 0 | 0 | -5.63 |
| *ftsA* | lmo2033 | highly similar to cell-division protein FtsA | Cell division | 0 | 0 | 0 | 0 | -3.87 |
| *divIB* | lmo2034 | similar to cell-division initiation protein divIB | Cell division | 0 | 0 | 0 | 0 | -0.07 |
| *NA* | lmo2167 | similar to unknown proteins | Toxin production and resistance | 0 | 0 | 0 | 0 | -1.63 |
| *NA* | lmo2217 | similar to unknown protein | Adaptations to atypical conditions | 0 | 0 | 0 | 0 | -0.92 |
| *NA* | lmo2235 | similar to NADH oxidase | Detoxification | 0 | 0 | 0 | 0 | -1.16 |
| *NA* | lmo2244 | similar to putative ribosomal large subunit pseudouridine synthase | Toxin production and resistance | 0 | 0 | 0 | 0 | -1.13 |
| *comK* | lmo2270 | similar to competence transcription factor ComK, N terminal part | DNA transformation | 0 | 0 | 0 | 0 | -1.02 |
| *NA* | lmo2385 | similar to B. subtilis YuxO protein | DNA transformation | 0 | 0 | 0 | 0 | 0.4 |
| *NA* | lmo2399 | similar to conserved hypothetical proteins | Toxin production and resistance | 0 | 0 | 0 | 0 | 0.37 |
| *NA* | lmo2426 | conserved hypothetical proteins | Detoxification | 0 | 0 | 0 | 0 | -0.98 |
| *NA* | lmo2426 | conserved hypothetical proteins | Toxin production and resistance | 0 | 0 | 0 | 0 | -0.98 |
| *NA* | lmo2427 | similar to cell division proteins RodA, FtsW | Cell division | 0 | 0 | 0 | 0 | -2.86 |
| *NA* | lmo2428 | similar to cell division proteins RodA, FtsW | Cell division | 0 | 0 | 0 | 0 | -1.16 |
| *NA* | lmo2470 | similar to internalin proteins | Other | 0 | 0 | 0 | 0 | 1.53 |
| *NA* | lmo2503 | similar to cardiolipin synthase | Toxin production and resistance | 0 | 0 | 0 | 0 | -0.79 |
| *comFC* | lmo2512 | similar to late competence protein comFC | DNA transformation | 0 | 0 | 0 | 0 | -0.47 |
| *NA* | lmo2554 | similar to galactosyltransferase | Toxin production and resistance | 0 | 0 | 0 | 0 | 0.13 |
| *NA* | lmo2687 | similar to cell division protein FtsW | Cell division | 0 | 0 | 0 | 0 | -0.56 |
| *NA* | lmo2688 | similar to cell division protein FtsW | Cell division | 0 | 0 | 0 | 0 | -0.95 |
| *NA* | lmo2755 | similar to acylase and diesterase | Toxin production and resistance | 0 | 0 | 0 | 0 | -0.05 |
| *parB* | lmo2790 | Partition protein ParB homolg | Cell division | 0 | 0 | 0 | 0 | -0.5 |
| *NA* | lmo2794 | highly similar to B. subtilis DNA-binding protein Spo0J-like homolog YyaA | Cell division | 0 | 0 | 0 | 0 | -0.18 |
| *NA* | lmo2803 | unknown | Cell division | 0 | 0 | 0 | 0 | -2.02 |
| **Mobile and extrachromosomal element functions** | | |  |  |  |  |  |  |
| *NA* | lmo0650 | conserved membrane protein | Prophage functions | 2.61 | UP | 0 | 0 | 0.79 |
| *NA* | lmo0152 | similar to oligopeptide ABC transporter-binding protein | Plasmid functions | 2.06 | 0 | -6.2 | 0 | -1.81 |
| *NA* | lmo0114 | similar to putative repressor C1 from lactococcal bacteriophage Tuc2009 | Prophage functions | 1.87 | UP | 0 | 0 | -1.1 |
| *NA* | lmo1955 | similar to integrase/recombinase | Prophage functions | 1.28 | 0 | 0 | 0 | 1.13 |
| *NA* | lmo2196 | similar to pheromone ABC transporter (binding protein) | Plasmid functions | -3.23 | DOWN | -2.17 | 0 | -0.5 |
| *NA* | lmo2569 | similar to dipeptide ABC transporter (dipeptide-binding protein) | Plasmid functions | -2.51 | 0 | 0 | 0 | -0.34 |
| *codV* | lmo1277 | similar to integrase/recombinase | Prophage functions | -2.04 | 0 | 0 | 0 | -0.66 |
| *NA* | lmo2070 | similar to unknown proteins | Prophage functions | -1.63 | 0 | 0 | 0 | 0.82 |
| *NA* | lmo1295 | similar to host factor-1 protein | Prophage functions | 0 | UP | 0 | 2.5 | 0.44 |
| *NA* | lmo2360 | transmembrane protein | Prophage functions | 0 | UP | 0 | 0 | -0.05 |
| *NA* | lmo2485 | similar to B. subtilis yvlC protein | Prophage functions | 0 | UP | 0 | 4.4 | 0.63 |
| *NA* | lmo2290 | Portein gp13 [Bacteriophage A118] | Prophage functions | 0 | 0 | 0 | 2.1 | -0.14 |
| *NA* | lmo0174 | similar to transposase | Transposon functions | 0 | 0 | 0 | 0 | -1.6 |
| *NA* | lmo0329 | similar to transposase | Transposon functions | 0 | 0 | 0 | 0 | -0.93 |
| *NA* | lmo0330 | similar to transposase | Transposon functions | 0 | 0 | 0 | 0 | -0.1 |
| *NA* | lmo0444 | conserved hypothetical protein | Prophage functions | 0 | 0 | 0 | 0 | 2.74 |
| *NA* | lmo0464 | weakly similar to transposase | Transposon functions | 0 | 0 | 0 | 0 | -0.99 |
| *NA* | lmo0660 | similar to transposases | Transposon functions | 0 | 0 | 0 | 0 | 2.4 |
| *NA* | lmo0827 | similar to transposases | Transposon functions | 0 | 0 | 0 | 0 | -0.57 |
| *NA* | lmo0828 | similar to transposases | Transposon functions | 0 | 0 | 0 | 0 | -2.47 |
| *NA* | lmo0832 | similar to transposase | Transposon functions | 0 | 0 | 0 | 0 | -2.53 |
| *NA* | lmo1097 | similar to integrases | Prophage functions | 0 | 0 | 0 | 0 | 0 |
| *NA* | lmo2279 | holin [Bacteriophage A118] | Prophage functions | 0 | 0 | 0 | 0 | -1 |
| **Unknown function** | | |  |  |  |  |  |  |
| *NA* | LMOf6854_0867 | acetyltransferase, GNAT family | Enzymes of unknown specificity | 4.34 | 0 | 0 | 0 | 0 |
| *NA* | LMOf2365_2255 | Ser/Thr protein phosphatase family protein | Enzymes of unknown specificity | 3.89 | 0 | 0 | 0 | 0 |
| *NA* | LMOf2365_0839 | acetyltransferase, GNAT family | Enzymes of unknown specificity | 3.78 | 0 | 0 | 0 | 0 |
| *NA* | LMOf2365_2458 | PspC domain protein, truncated | General | 3.22 | 0 | 0 | 0 | 0 |
| *NA* | LMOf2365_1708 | D-isomer specific 2-hydroxyacid dehydrogenase family protein | Enzymes of unknown specificity | 3.17 | 0 | 0 | 0 | 0 |
| *NA* | lmo1975 | similar to E. coli DNA-damage-inducible protein dinP | General | 2.38 | UP | -3.64 | 0 | -0.68 |
| *NA* | LMOh7858_1807 | D-isomer specific 2-hydroxyacid dehydrogenase family protein | Enzymes of unknown specificity | 2.09 | 0 | 0 | 0 | 0 |
| *NA* | LMOf6854_0856 | HD domain protein | General | 1.93 | 0 | 0 | 0 | 0 |
| *NA* | lmo2412 | similar to conserved hypothetical proteins | General | 1.9 | 0 | 0 | 0 | 0.61 |
| *lemA* | lmo0962 | Listeria epitope LemA | General | 1.85 | 0 | 0 | 0 | -1.71 |
| *NA* | lmo1760 | similar to unknown protein | General | 1.82 | 0 | 0 | 0 | 0.71 |
| *NA* | lmo1382 | unknown | General | 1.79 | 0 | 0 | 0 | 2.65 |
| *NA* | lmo0790 | similar to transcription regulator (EbsC from Enterococcus faecalis) | General | 1.63 | 0 | 0 | 0 | 0.64 |
| *NA* | LMOf2365_2383 | NifU family protein | General | 1.6 | 0 | 0 | 0 | 0 |
| *NA* | LMOf6854_0747 | flagellar motor switch domain protein | General | 1.55 | 0 | 0 | 0 | 0 |
| *NA* | lmo1865 | similar to conserved hypothetical proteins | General | 1.54 | 0 | 0 | 0 | -0.22 |
| *NA* | LMOf2365_0306 | aminotransferase, class I | Enzymes of unknown specificity | -4.87 | 0 | 0 | 0 | 0 |
| *NA* | lmo1067 | similar to GTP-binding elongation factor | General | -4.37 | DOWN | 3.14 | 0 | 1.2 |
| *NA* | LMOf2365_0292 | hydrolase, haloacid dehalogenase-like family | Enzymes of unknown specificity | -3.57 | 0 | 0 | 0 | 0 |
| *NA* | lmo2127 | unknown | General | -3.47 | 0 | 0 | 0 | -1.27 |
| *NA* | LMOh7858_2654 | NLP/P60 family protein | General | -3.36 | 0 | 0 | 0 | 0 |
| *NA* | LMOf2365_2478 | NLP/P60 family protein | General | -2.96 | 0 | 0 | 0 | 0 |
| *NA* | LMOf6854_2752 | pyridine nucleotide-disulfide oxidoreductase family protein | Enzymes of unknown specificity | -2.8 | 0 | 0 | 0 | 0 |
| *NA* | LMOf6854_1997 | GTPase family protein | Enzymes of unknown specificity | -2.78 | 0 | 0 | 0 | 0 |
| *NA* | lmo1937 | similar to unknown protein | General | -2.77 | DOWN | 2.62 | -2 | -2.32 |
| *NA* | lmo0597 | similar to transcription regulator CRP/FNR family | General | -2.52 | 0 | 0 | 0 | -0.01 |
| *NA* | LMOf2365_2611 | pyridine nucleotide-disulfide oxidoreductase family protein | Enzymes of unknown specificity | -2.38 | 0 | 0 | 0 | 0 |
| *NA* | LMOf2365_0626 | cyclic nucleotide-binding protein | General | -2.26 | 0 | 0 | 0 | 0 |
| *lepA* | LMOf6854_1524 | GTP-binding protein LepA | General | -2.12 | 0 | 0 | 0 | 0 |
| *NA* | lmo1066 | similar to extragenic suppressor protein SuhB and to myo-inositol-1(or 4)-monophosphatase | General | -2.11 | 0 | 0 | 0 | 1.22 |
| *NA* | lmo1615 | similar to unknown proteins | Enzymes of unknown specificity | -2.09 | 0 | 0 | 0 | 1.25 |
| *gidB* | lmo2802 | GidB protein | General | -2.08 | 0 | 0 | 0 | 2.7 |
| *NA* | lmo1240 | conserved hypothetical protein, similar to B. subtilis YsnB protein | Enzymes of unknown specificity | -2.07 | DOWN | -1.6 | 0 | 0.51 |
| *ychF* | LMOf6854_2897 | GTP-binding protein YchF | General | -2.04 | 0 | 0 | 0 | 0 |
| *NA* | lmo1239 | conserved hypothetical protein, similar to B. subtilis YsnA protein | General | -1.98 | 0 | -1.11 | 0 | -0.32 |
| *NA* | LMOf6854_2131 | CAAX amino terminal protease family protein | General | -1.88 | 0 | 0 | 0 | 0 |
| *NA* | lmo1558 | similar to hypothetical GTP binding protein | General | -1.88 | DOWN | 0 | 0 | 0.96 |
| *NA* | LMOf2365_0287 | phosphoglycerate mutase family protein | Enzymes of unknown specificity | -1.87 | 0 | 0 | 0 | 0 |
| *lepA* | lmo1479 | highly similar to GTP-binding protein LepA | General | -1.77 | 0 | 0 | 0 | 0.05 |
| *NA* | lmo1441 | similar to putative peptidoglycan acetylation protein | General | -1.7 | 0 | 0 | 0 | -1.05 |
| *NA* | LMOf2365_1579 | GTP-binding protein | Enzymes of unknown specificity | -1.54 | 0 | 0 | 0 | 0 |
| *NA* | LMOf2365_1313 | GTP-binding domain protein | General | -1.52 | 0 | 0 | 0 | 0 |
| *NA* | lmo2829 | similar to yeast protein Frm2p involved in fatty acid signaling | Enzymes of unknown specificity | -1.51 | UP | 0 | 0 | -0.2 |
| *NA* | LMOf6854_1629 | metallo-beta-lactamase family protein | Enzymes of unknown specificity | -1.51 | 0 | 0 | 0 | 0 |
| *NA* | LMOf6854_2603 | modification methylase, HemK family | General | -1.5 | 0 | 0 | 0 | 0 |
| *NA* | lmo0414 | conserved membrane protein | General | -1.49 | 0 | 0 | 0 | 0.69 |
| *NA* | lmo1442 | similar to transport proteins | General | -1.48 | 0 | 0 | 0 | 0.84 |
| *NA* | LMOf6854_1628 | CBS domain protein | General | -1.48 | 0 | 0 | 0 | 0 |
| *NA* | LMOf6854_0639 | BioY family protein | General | -1.45 | 0 | 0 | 0 | 0 |
| *NA* | lmo2209 | unknown | Enzymes of unknown specificity | -1.44 | 0 | 0 | 0 | -1.21 |
| *NA* | LMOf6854_1627 | DHH subfamily 1 protein | General | -1.44 | 0 | 0 | 0 | 0 |
| *NA* | lmo1491 | similar to unknown proteins | General | -1.39 | DOWN | 0 | 0 | -0.3 |
| *NA* | lmo1515 | similar to unknown protein | General | -1.38 | 0 | 0 | 0 | -0.05 |
| *NA* | LMOf6854_0556 | maturase-related protein | General | -1.38 | 0 | 0 | 0 | 0 |
| *NA* | LMOf2365_1598 | CBS domain protein | General | -1.37 | 0 | 0 | 0 | 0 |
| *NA* | LMOh7858_0712 | acetyltransferase, GNAT family | Enzymes of unknown specificity | -1.34 | 0 | 0 | 0 | 0 |
| *NA* | lmo2369 | similar to B. subtilis general stress protein 13 containing a ribosomal S1 protein domain | General | -1.33 | 0 | 0 | 0 | -0.03 |
| *NA* | lmo1019 | similar to B. subtilis YitL protein | General | -1.3 | 0 | 0 | 0 | 1.09 |
| *NA* | lmo0998 | similar to hypothetical protein | General | -1.21 | 0 | 0 | 0 | -0.76 |
| *NA* | LMOf2365_2373 | CBS domain protein | General | -1.18 | 0 | 0 | 0 | 0 |
| *NA* | lmo1117 | unknown | General | -1.16 | 0 | 0 | 0 | -1.47 |
| *NA* | lmo0554 | similar to NADH-dependent butanol dehydrogenase | General | 0 | UP | 0 | 4 | 2.67 |
| *NA* | lmo0590 | similar to a fusion of two types of conserved hypothetical proteinconserved hypothetical | General | 0 | UP | 0 | 4.1 | 0.48 |
| *NA* | lmo0291 | conserved hypothetical protein similar to B. subtilis YycJ protein | General | 0 | UP | 0 | 2 | 1.52 |
| *NA* | lmo0602 | weakly similar to transcription regulator | General | 0 | UP | 0 | 5.5 | 0.74 |
| *NA* | lmo0816 | similar to B. subtilis regulatory protein PaiA | General | 0 | UP | 0 | 0 | -1.56 |
| *NA* | lmo0919 | similar to ABC transporter ATP-binding protein (antibiotic resistance) | General | 0 | UP | 0 | 0 | 0.83 |
| *NA* | lmo2109 | similar to hydrolase | Enzymes of unknown specificity | 0 | UP | 0 | 0 | -0.86 |
| *NA* | lmo2174 | similar to unknown proteins | General | 0 | UP | 0 | 0 | -0.63 |
| *NA* | lmo2361 | conserved hypothetical protein | General | 0 | UP | 0 | 0 | 0.87 |
| *NA* | lmo2589 | similar to transcription regulator TetR/AcrR family | General | 0 | UP | 0 | 0 | -0.35 |
| *NA* | lmo2796 | similar to transcription regulator | General | 0 | UP | 0 | 0 | 0.86 |
| *NA* | lmo0187 | similar to B. subtilis YabF protein | General | 0 | DOWN | 0 | 0 | -0.53 |
| *NA* | lmo0273 | unknown | Enzymes of unknown specificity | 0 | DOWN | 0 | 0 | 1.15 |
| *NA* | lmo0313 | conserved hypothetical protein | Enzymes of unknown specificity | 0 | DOWN | 0 | 0 | -0.13 |
| *NA* | lmo1486 | unknown | General | 0 | DOWN | 0 | 0 | 1.38 |
| *NA* | lmo1537 | conserved GTP binding protein | General | 0 | DOWN | 0 | 0 | 0.12 |
| *NA* | lmo1604 | similar to 2-cys peroxiredoxin | General | 0 | DOWN | -2.41 | -2.1 | 1.48 |
| *NA* | lmo1614 | similar to unknown proteins | General | 0 | DOWN | 0 | 0 | 0.33 |
| *NA* | lmo0624 | similar to unknown proteins | Enzymes of unknown specificity | 0 | 0 | 3.57 | 0 | 0.98 |
| *NA* | lmo1238 | similar to ribonuclease PH | General | 0 | 0 | -2.21 | 0 | 0.02 |
| *NA* | lmo1027 | similar to conserved hypothetical proteins (in particular B. subtilis YkqC) | General | 0 | 0 | 0 | 2.5 | -2.19 |
| *NA* | lmo1285 | conserved hypothetical protein, similar to B. subtilis YneT protein | General | 0 | 0 | 0 | -2.1 | -0.67 |
| *NA* | lmo1826 | unknown | General | 0 | 0 | 0 | -2.2 | 0.7 |
| *NA* | lmo0017 | similar to Bacillus anthracis CapA protein (polyglutamate capsule biosynthesis) | General | 0 | 0 | 0 | 0 | -0.92 |
| *NA* | lmo0042 | similar to E. coli DedA protein | General | 0 | 0 | 0 | 0 | -0.74 |
| *NA* | lmo0062 | unknown | General | 0 | 0 | 0 | 0 | 2.08 |
| *NA* | lmo0067 | similar to dinitrogenase reductase ADP-ribosylation system | General | 0 | 0 | 0 | 0 | -2.06 |
| *NA* | lmo0106 | similar to transcription regulator | General | 0 | 0 | 0 | 0 | -1.19 |
| *NA* | lmo0191 | similar to a putative phospho-beta-glucosidase | General | 0 | 0 | 0 | 0 | -0.82 |
| *NA* | lmo0212 | unknown | Enzymes of unknown specificity | 0 | 0 | 0 | 0 | -0.34 |
| *NA* | lmo0227 | conserved hypothetical protein | General | 0 | 0 | 0 | 0 | 1.36 |
| *NA* | lmo0320 | similar to surface protein (peptidoglycan bound, LPXTG motif) | General | 0 | 0 | 0 | 0 | 0.46 |
| *NA* | lmo0382 | similar to B. subtilis transcription repressor of myo-inositol catabolism operon IolR | General | 0 | 0 | 0 | 0 | 2 |
| *NA* | lmo0436 | similar to unknown proteins | General | 0 | 0 | 0 | 0 | 1.81 |
| *NA* | lmo0516 | similar to Bacillus anthracis encapsulation protein CapA | General | 0 | 0 | 0 | 0 | 1.65 |
| *NA* | lmo0636 | similar to unknown proteins | General | 0 | 0 | 0 | 0 | 0.19 |
| *NA* | lmo0694 | unknown | General | 0 | 0 | 0 | 0 | 0.11 |
| *NA* | lmo0762 | similar to ATP/GTP-binding protein | General | 0 | 0 | 0 | 0 | -1.58 |
| *NA* | lmo0776 | similar to transcription regulator (repressor) | General | 0 | 0 | 0 | 0 | 1.02 |
| *NA* | lmo0795 | conserved hypothetical protein | General | 0 | 0 | 0 | 0 | -2.56 |
| *NA* | lmo0857 | similar to carboxylesterase | Enzymes of unknown specificity | 0 | 0 | 0 | 0 | 0.17 |
| *NA* | lmo0898 | conserved hypothetical protein | General | 0 | 0 | 0 | 0 | 0.67 |
| *NA* | lmo0899 | similar to B. subtilis YdcK protein | General | 0 | 0 | 0 | 0 | 0.9 |
| *NA* | lmo0976 | similar to B. subtilis YjcF protein | General | 0 | 0 | 0 | 0 | -1.83 |
| *NA* | lmo1049 | similar to molybdopterin biosynthesis protein MoeB | General | 0 | 0 | 0 | 0 | 1.54 |
| *NA* | lmo1111 | highly similar to TN916 ORF20 | General | 0 | 0 | 0 | 0 | 0.32 |
| *NA* | lmo1129 | similar to unknown proteins | Enzymes of unknown specificity | 0 | 0 | 0 | 0 | -0.91 |
| *NA* | lmo1230 | similar to B. subtilis YshB protein | General | 0 | 0 | 0 | 0 | 0.02 |
| *NA* | lmo1272 | conserved hypothetical protein similar to B. subtilis YlqF protein | General | 0 | 0 | 0 | 0 | 0.75 |
| *gid* | lmo1276 | similar glucose inhibited division protein A | General | 0 | 0 | 0 | 0 | 1.12 |
| *NA* | lmo1296 | conserved hypothetical protein similar to B. subtilis YnbA protein | General | 0 | 0 | 0 | 0 | 1.83 |
| *NA* | lmo1434 | similar to unknown proteins | General | 0 | 0 | 0 | 0 | 0.78 |
| *NA* | lmo1500 | similar to unknown proteins | General | 0 | 0 | 0 | 0 | 0.67 |
| *NA* | lmo1549 | similar to DNA repair protein RadC | General | 0 | 0 | 0 | 0 | 1.09 |
| *NA* | lmo1569 | similar to unknown proteins | General | 0 | 0 | 0 | 0 | 0.16 |
| *NA* | lmo1576 | similar to unknown proteins | General | 0 | 0 | 0 | 0 | 0.02 |
| *NA* | lmo1577 | similar to unknown proteins | General | 0 | 0 | 0 | 0 | 0.4 |
| *pheT* | lmo1607 | similar phenylalanyl-tRNA synthetase (beta subunit) | General | 0 | 0 | 0 | 0 | -0.9 |
| *NA* | lmo1622 | similar to unknown proteins | General | 0 | 0 | 0 | 0 | -0.45 |
| *NA* | lmo1859 | similar to transcriptional regulator (PilB family) | General | 0 | 0 | 0 | 0 | 0.58 |
| *NA* | lmo1912 | similar to unknown proteins (hypothetical sensory transduction histidine kinase) | General | 0 | 0 | 0 | 0 | -0.16 |
| *NA* | lmo1977 | similar to unknown proteins | General | 0 | 0 | 0 | 0 | 0.56 |
| *NA* | lmo1982 | similar to unknown proteins | Enzymes of unknown specificity | 0 | 0 | 0 | 0 | 1.18 |
| *NA* | lmo2005 | similar to oxidoreductase | General | 0 | 0 | 0 | 0 | 1.85 |
| *NA* | lmo2053 | similar to unknown proteins | Enzymes of unknown specificity | 0 | 0 | 0 | 0 | -1.65 |
| *NA* | lmo2081 | similar to unknown protein | General | 0 | 0 | 0 | 0 | -1.5 |
| *NA* | lmo2082 | similar to unknown proetin | General | 0 | 0 | 0 | 0 | -1.74 |
| *NA* | lmo2165 | similar to transcription regulator CRP/FNR family | General | 0 | 0 | 0 | 0 | -4.34 |
| *NA* | lmo2167 | similar to unknown proteins | General | 0 | 0 | 0 | 0 | -1.63 |
| *NA* | lmo2216 | similar to histidine triad (HIT) protein | General | 0 | 0 | 0 | 0 | -1.39 |
| *NA* | lmo2260 | similar to unknown proteins | Enzymes of unknown specificity | 0 | 0 | 0 | 0 | -0.71 |
| *NA* | lmo2261 | similar to unknown proteins | General | 0 | 0 | 0 | 0 | -0.88 |
| *NA* | lmo2288 | Protein gp15 [Bacteriophage A118] | General | 0 | 0 | 0 | 0 | -0.87 |
| *NA* | lmo2397 | similar to NifU protein | General | 0 | 0 | 0 | 0 | -1.6 |
| *NA* | lmo2401 | similar to conserved hypothetical protein and to B. subtilis YutF protein | General | 0 | 0 | 0 | 0 | -0.01 |
| *NA* | lmo2649 | similar to hypothetical PTS enzyme IIC component | General | 0 | 0 | 0 | 0 | 1.68 |
| *NA* | lmo2723 | similar to unknown proteins | General | 0 | 0 | 0 | 0 | -0.02 |
| *NA* | lmo2779 | similar to probable GTP-binding protein | General | 0 | 0 | 0 | 0 | 0.88 |
| *NA* | lmo2789 | unknown | General | 0 | 0 | 0 | 0 | 0.28 |
| *gidA* | lmo2810 | highly similar to GidA protein | General | 0 | 0 | 0 | 0 | -0.78 |
| **Hypothetical proteins** | | |  |  |  |  |  |  |
| *NA* | LMOf2365_0838 | conserved hypothetical protein | Conserved | 5.56 | 0 | 0 | 0 | 0 |
| *NA* | lmo0647 | unknown | NA | 4.38 | UP | 2.68 | 4.8 | 0.13 |
| *NA* | LMOf2365_2819 | conserved hypothetical protein | Conserved | 3.67 | 0 | 0 | 0 | 0 |
| *NA* | lmo1690 | similar to hypothetical proteins | Conserved | 3.64 | 0 | 4.61 | 0 | 1.24 |
| *NA* | lmo0821 | unknown | NA | 3.63 | 0 | 0 | 0 | -2.79 |
| *NA* | LMOf2365_0975 | conserved hypothetical protein | Conserved | 3.55 | 0 | 0 | 0 | 0 |
| *NA* | lmo0819 | unknown | NA | 3.32 | UP | 0 | 0 | -0.9 |
| *NA* | lmo2487 | similar to B. subtilis YvlB protein | Conserved | 3.21 | UP | 0 | 0 | -0.28 |
| *NA* | lmo2828 | unknown | NA | 3.15 | UP | 0 | 0 | 1.44 |
| *NA* | lmo0654 | unknown | NA | 3.13 | UP | 0 | 2.2 | 0.57 |
| *NA* | lmo2486 | unknown | Conserved | 3.08 | UP | 0 | 0 | 1.12 |
| *NA* | lmo0911 | unknown | Conserved | 2.72 | UP | 0 | 3.8 | -0.57 |
| *NA* | LMOf2365_1322 | conserved hypothetical protein | Conserved | 2.68 | 0 | 0 | 0 | 0 |
| *NA* | lmo0954 | unknown | NA | 2.6 | UP | 2.79 | 0 | -1.03 |
| *NA* | lmo1640 | unknown | NA | 2.6 | 0 | 0 | 0 | 2.19 |
| *NA* | lmo0796 | conserved hypothetical protein | Conserved | 2.55 | UP | 0 | 6.7 | 0.44 |
| *NA* | LMOf2365_2152 | conserved hypothetical protein | Conserved | 2.44 | 0 | 0 | 0 | 0 |
| *NA* | lmo2567 | unknown | NA | 2.44 | 0 | 0 | 0 | 1.58 |
| *NA* | lmo0955 | unknown | Conserved | 2.41 | UP | 0 | 0 | -2.25 |
| *NA* | lmo1068 | unknown | NA | 2.36 | UP | 0 | 0 | -1 |
| *NA* | LMOf2365_1996 | conserved hypothetical protein | Conserved | 2.35 | 0 | 0 | 0 | 0 |
| *NA* | lmo2119 | similar to unknown proteins | Conserved | 2.24 | 0 | 0 | 0 | -0.92 |
| *NA* | lmo0932 | conserved hypothetical protein | Conserved | 2.23 | 0 | 0 | 0 | -1.06 |
| *NA* | lmo2339 | conserved hypothetical protein | Conserved | 2.22 | 0 | 0 | 0 | -1.04 |
| *NA* | lmo2191 | similar to unknown proteins | Conserved | 2.2 | UP | 2.51 | 3.5 | -1.79 |
| *NA* | lmo1304 | similar to B. subtilis YnzC protein | Conserved | 2.2 | 0 | 0 | 0 | 2 |
| *NA* | lmo2120 | similar to unknown proteins | Conserved | 2.04 | 0 | 0 | 0 | -1.15 |
| *NA* | lmo1568 | similar to unknown proteins | Conserved | 2.03 | 0 | 0 | 0 | -0.05 |
| *NA* | lmo1757 | similar to unknown protein | Conserved | 2.01 | 0 | 0 | 0 | 0.81 |
| *NA* | lmo2393 | similar to B. subtilis YuzD protein | Conserved | 1.9 | 0 | 0 | 0 | 0.13 |
| *NA* | lmo2265 | similar to unknown proteins | Conserved | 1.88 | 0 | 0 | 0 | -0.36 |
| *NA* | lmo0701 | unknown | NA | 1.88 | 0 | 0 | -2.1 | 0.87 |
| *NA* | lmo0950 | unknown | Conserved | 1.86 | 0 | 0 | 0 | -0.32 |
| *NA* | lmo0774 | conserved hypothetical protein | Conserved | 1.79 | 0 | 0 | 0 | 0.11 |
| *NA* | lmo2557 | conserved hypothetical protein | Conserved | 1.79 | UP | 0 | 0 | -1.78 |
| *NA* | LMOf2365_2463 | conserved hypothetical protein | Conserved | 1.78 | 0 | 0 | 0 | 0 |
| *NA* | lmo0419 | similar to unknown protein | NA | 1.76 | 0 | 0 | 0 | 0.71 |
| *NA* | lmo2239 | unknown | NA | 1.73 | 0 | 0 | 0 | 1.05 |
| *NA* | lmo2054 | similar to unknown proteins | Conserved | 1.69 | 0 | 0 | 0 | 1.17 |
| *NA* | lmo1358 | similar to B. subtilis YqhY protein | Conserved | 1.69 | 0 | 0 | 0 | 0.21 |
| *NA* | lmo2857 | hypothetical protein | NA | 1.68 | 0 | 0 | 0 | 0 |
| *NA* | lmo2062 | similar to copper export proteins | Conserved | 1.68 | 0 | 0 | 0 | -0.03 |
| *NA* | lmo0004 | similar to B. subtilis YaaA protein | Conserved | 1.67 | 0 | 0 | 0 | -0.13 |
| *NA* | lmo0964 | similar to B. subtilis YjbH protein | Conserved | 1.66 | 0 | 0 | 0 | -1.06 |
| *NA* | lmo1687 | similar to hypothetical proteins | Conserved | 1.66 | 0 | 0 | 0 | 0.55 |
| *NA* | lmo2441 | similar to transcription regulator | Conserved | 1.66 | 0 | 0 | 0 | -1.03 |
| *NA* | lmo1602 | similar to unknown proteins | Conserved | 1.66 | UP | 0 | 3.6 | 0.81 |
| *NA* | lmo0941 | unknown | Conserved | 1.64 | 0 | 0 | 0 | 0.75 |
| *NA* | lmo0231 | similar to arginine kinase | Conserved | 1.64 | UP | 0 | 2 | -1.63 |
| *NA* | lmo2074 | similar to unknown proteins | Conserved | 1.64 | 0 | 0 | 0 | 1.21 |
| *NA* | lmo0702 | unknown | NA | 1.63 | 0 | 0 | -2.2 | 0.39 |
| *NA* | lmo0726 | Hypothetical CDS | NA | 1.61 | 0 | 0 | 0 | -0.04 |
| *NA* | LMOf2365_2714 | conserved hypothetical protein | Conserved | 1.58 | 0 | 0 | 0 | 0 |
| *NA* | lmo2574 | unknown | NA | 1.58 | UP | 0 | 0 | -0.02 |
| *NA* | lmo0408 | unknown | Conserved | 1.56 | 0 | 0 | 0 | -1.43 |
| *NA* | lmo2578 | unknown | Conserved | 1.55 | 0 | 0 | 0 | 0.39 |
| *NA* | lmo1058 | similar to B. subtilis YktA protein | Conserved | 1.53 | 0 | 0 | 0 | 0.15 |
| *NA* | lmo2416 | unknown | NA | 1.49 | DOWN | 0 | 0 | 1.17 |
| *NA* | lmo2472 | conserved hypothetical protein | Conserved | 1.49 | UP | 0 | 0 | -0.11 |
| *NA* | lmo0622 | hypothetical | NA | 1.45 | 0 | 0 | 0 | -0.51 |
| *NA* | lmo1753 | similar to unknown protein | Conserved | 1.45 | 0 | 0 | 0 | 1.81 |
| *NA* | lmo1219 | unknown | NA | 1.42 | 0 | -2.52 | 0 | 0.59 |
| *NA* | lmo1020 | similar to B. subtilis YvqF protein | Conserved | 1.42 | UP | 0 | 0 | 0.19 |
| *NA* | lmo2527 | similar to B. subtilis YwzB protein | Conserved | 1.35 | UP | 0 | 0 | 1.64 |
| *NA* | lmo1670 | similar to conserved hypothetical proteins | Conserved | 1.34 | 0 | 2.46 | 0 | 0.09 |
| *NA* | lmo2223 | similar to unknown proteins | Conserved | 1.33 | DOWN | 0 | 0 | -2.6 |
| *NA* | lmo1752 | unknown | NA | 1.33 | 0 | 0 | 0 | -0.38 |
| *NA* | lmo2140 | similar to ABC transporter (membrane protein) | Conserved | 1.31 | 0 | 0 | 0 | -0.05 |
| *NA* | lmo0946 | unknown | Conserved | 1.3 | 0 | 0 | 0 | -0.09 |
| *NA* | LMOf2365_0393 | conserved hypothetical protein | Conserved | 1.29 | 0 | 0 | 0 | 0 |
| *NA* | lmo0939 | unknown | NA | 1.26 | 0 | 0 | 0 | -1.47 |
| *NA* | lmo1503 | unknown | Conserved | 1.23 | 0 | 0 | 0 | 1.08 |
| *NA* | lmo0332 | unknown | Conserved | 1.19 | 0 | 0 | 0 | 0.79 |
| *NA* | lmo1945 | similar to unknown protein | Conserved | 1.16 | 0 | 0 | -1.4 | 2.81 |
| *NA* | lmo0731 | unknown | NA | -4.73 | 0 | -2.59 | 0 | 3.41 |
| *NA* | lmo1025 | unknown | NA | -3.26 | DOWN | 0 | 0 | -1.23 |
| *NA* | LMOf2365_1046 | conserved hypothetical protein | Conserved | -3.21 | 0 | 0 | 0 | 0 |
| *NA* | LMOf2365_0737 | conserved hypothetical protein | Conserved | -3.05 | 0 | 0 | 0 | 0 |
| *NA* | lmo1334 | similar to B. subtilis YqzD protein | Conserved | -3.04 | 0 | 0 | 0 | 0.8 |
| *NA* | lmo0099 | unknown | Conserved | -3.01 | DOWN | -2.42 | 0 | 1.43 |
| *NA* | lmo1417 | highly similar to B. subtilis YxiO protein | Conserved | -3.01 | DOWN | 0 | 0 | 1.9 |
| *NA* | lmo2516 | similar to conserved hypothetical proteins | Conserved | -2.83 | 0 | 0 | 0 | 1.43 |
| *NA* | lmo1024 | unknown | NA | -2.8 | 0 | 0 | 0 | -1.3 |
| *NA* | lmo0269 | similar to transporter | NA | -2.77 | 0 | 0 | 0 | -0.66 |
| *NA* | lmo2248 | similar to unknown proteins | Conserved | -2.62 | DOWN | 0 | 0 | -1.44 |
| *NA* | LMOf2365_2053 | hypothetical protein | NA | -2.55 | 0 | 0 | 0 | 0 |
| *NA* | lmo0883 | similar to B. subtilis YbtB protein | Conserved | -2.55 | 0 | 0 | 0 | 0.07 |
| *NA* | lmo1918 | similar to unknown proteins | Conserved | -2.5 | 0 | 0 | 0 | 0.83 |
| *NA* | lmo1333 | similar to B. subtilis YqzC protein | Conserved | -2.46 | 0 | 0 | 0 | 1.63 |
| *NA* | LMOf2365_1685 | conserved hypothetical protein TIGR01212 | Conserved | -2.44 | 0 | 0 | 0 | 0 |
| *NA* | lmo2104 | unknown | Conserved | -2.44 | 0 | 0 | 0 | -1.88 |
| *NA* | lmo0777 | unknown | NA | -2.4 | 0 | 0 | 0 | 1.47 |
| *NA* | lmo0882 | similar to B. subtilis YdbS protein | Conserved | -2.36 | 0 | 0 | 0 | -0.54 |
| *NA* | lmo1810 | similar to unknown proteins | Conserved | -2.35 | 0 | 0 | 0 | 1.1 |
| *NA* | lmo0867 | unknown | Conserved | -2.28 | 0 | 0 | 0 | 0.6 |
| *NA* | lmo1211 | similar to unknown proteins | Conserved | -2.22 | 0 | 0 | 0 | 0.5 |
| *NA* | LMOf2365_2562 | hypothetical protein | NA | -2.22 | 0 | 0 | 0 | 0 |
| *NA* | lmo2204 | similar to unknown protein | Conserved | -2.22 | DOWN | 0 | 0 | -0.98 |
| *NA* | LMOf2365_0901 | conserved hypothetical protein | Conserved | -2.21 | 0 | 0 | 0 | 0 |
| *NA* | LMOf2365_1045 | conserved hypothetical protein | Conserved | -2.21 | 0 | 0 | 0 | 0 |
| *NA* | lmo2115 | similar to ABC transporter (permease) | Conserved | -2.2 | DOWN | 0 | 0 | -0.41 |
| *rpmG* | lmo1335 | ribosomal protein L33 | NA | -2.14 | 0 | 0 | 0 | 2.71 |
| *NA* | lmo0482 | similar to conserved hypothetical proteins, highly similar to B. subtilis YloN protein | Conserved | -2.13 | 0 | 0 | 0 | 0.21 |
| *NA* | lmo0484 | unknown | Conserved | -2.1 | DOWN | 0 | -2.6 | 3.2 |
| *NA* | lmo1236 | similar to B. subtilis YslB protein | Conserved | -2.09 | 0 | 0 | 0 | 0.28 |
| *NA* | lmo2063 | unknown | Conserved | -2.05 | 0 | 0 | 0 | 2.03 |
| *NA* | lmo0369 | conserved hypothetical protein, highly similar to B. subtilis YeeI protein | Conserved | -2.04 | 0 | 0 | 0 | 0.64 |
| *NA* | lmo1495 | similar to unknown proteins | Conserved | -2.01 | 0 | 0 | 0 | 0.87 |
| *NA* | LMOf2365_2691 | hypothetical protein | NA | -1.98 | 0 | 0 | 0 | 0 |
| *NA* | lmo1056 | unknown | NA | -1.95 | 0 | 2.03 | 0 | 0.48 |
| *NA* | lmo0666 | similar to unknown protein | Conserved | -1.94 | DOWN | 0 | 0 | 2.71 |
| *NA* | lmo0836 | similar to B. subtilis YrkR protein | Conserved | -1.9 | 0 | 0 | 0 | -0.53 |
| *NA* | lmo1582 | weakly similar to site specific DNA-methyltransferase | Conserved | -1.9 | 0 | 0 | 0 | 0.3 |
| *NA* | lmo2492 | unknown | NA | -1.89 | UP | 0 | 0 | 1.17 |
| *NA* | lmo1352 | unknown | NA | -1.87 | 0 | 0 | 0 | 1.57 |
| *NA* | lmo0831 | unknown | Conserved | -1.81 | 0 | 0 | 0 | -1.36 |
| *NA* | lmo1979 | similar to unknown proteins | Conserved | -1.81 | 0 | 0 | 0 | 1.27 |
| *NA* | LMOf2365_0509 | conserved hypothetical protein TIGR00048 | Conserved | -1.79 | 0 | 0 | 0 | 0 |
| *NA* | lmo0791 | unknown | Conserved | -1.78 | 0 | 0 | 0 | 0.33 |
| *NA* | lmo1188 | unknown | NA | -1.78 | 0 | 0 | 0 | -1.33 |
| *NA* | lmo1440 | similar to unknown proteins | Conserved | -1.77 | DOWN | 0 | 0 | 2.2 |
| *NA* | lmo1920 | similar to unknown proteins | Conserved | -1.76 | 0 | 0 | 0 | 1.09 |
| *NA* | lmo0100 | unknown | Conserved | -1.76 | 0 | 0 | 0 | 0.15 |
| *NA* | lmo1802 | similar to unknown proteins | Conserved | -1.75 | 0 | 0 | 0 | 0.67 |
| *NA* | lmo2148 | similar to unknown proteins | Conserved | -1.73 | DOWN | 0 | 0 | -1.53 |
| *NA* | LMOf2365_0385 | conserved hypothetical protein TIGR01033 | Conserved | -1.73 | 0 | 0 | 0 | 0 |
| *NA* | lmo1815 | similar to unknown protein | Conserved | -1.73 | 0 | 0 | 0 | -1.63 |
| *NA* | lmo1980 | unknown | NA | -1.72 | 0 | 0 | 0 | 0.58 |
| *NA* | LMOf2365_2757 | conserved hypothetical protein | Conserved | -1.71 | 0 | 0 | 0 | 0 |
| *NA* | lmo1410 | unknown | Conserved | -1.71 | 0 | 0 | 0 | 2.2 |
| *NA* | lmo1535 | similar to unknown proteins | Conserved | -1.7 | UP | 0 | 0 | -1.18 |
| *NA* | lmo1744 | similar to unknown proteins | Conserved | -1.7 | 0 | 0 | 0 | 1.82 |
| *NA* | lmo2405 | unknown | Conserved | -1.7 | DOWN | 0 | 0 | 0.51 |
| *NA* | lmo1092 | conserved hypothetical protein, similar to B. subtilis YueK protein | Conserved | -1.7 | 0 | 0 | 0 | -0.87 |
| *NA* | lmo1584 | similar to unknown proteins | Conserved | -1.69 | 0 | 0 | 0 | 0.3 |
| *NA* | lmo0665 | unknown | NA | -1.69 | DOWN | 0 | 0 | 2.39 |
| *NA* | lmo0247 | unknown | NA | -1.68 | 0 | 0 | 0 | -0.49 |
| *NA* | lmo2048 | similar to unknown proteins | Conserved | -1.66 | 0 | 0 | 0 | 0.93 |
| *NA* | lmo2218 | unknown | Conserved | -1.66 | DOWN | 0 | 0 | -0.58 |
| *NA* | lmo1950 | similar to unknown proteins | Conserved | -1.65 | 0 | 0 | 0 | -0.52 |
| *NA* | LMOf2365_2379 | conserved hypothetical protein | Conserved | -1.64 | 0 | 0 | 0 | 0 |
| *NA* | lmo0745 | unknown | NA | -1.64 | 0 | 0 | 0 | -0.43 |
| *NA* | lmo0531 | unknown | Conserved | -1.63 | 0 | 0 | 0 | 1.46 |
| *NA* | lmo1429 | similar to unknown proteins | Conserved | -1.6 | UP | 2.03 | 0 | 0.73 |
| *NA* | lmo1750 | similar to unknown protein | Conserved | -1.6 | 0 | 0 | 0 | 1.58 |
| *NA* | lmo1857 | similar to hypoyhetical protein | Conserved | -1.6 | 0 | 0 | 0 | 0.36 |
| *NA* | lmo1031 | similar to hypothetical proteins | Conserved | -1.58 | 0 | 0 | 0 | -0.35 |
| *NA* | lmo0623 | unknown | NA | -1.54 | 0 | 0 | 0 | 1.37 |
| *NA* | lmo0270 | unknown | NA | -1.53 | 0 | 0 | 0 | -0.15 |
| *NA* | lmo0720 | unknown | Conserved | -1.53 | UP | 0 | 0 | 1.23 |
| *NA* | lmo1626 | unknown | NA | -1.53 | DOWN | -1.24 | 0 | -0.74 |
| *NA* | lmo2151 | similar to unknown proteins | Conserved | -1.5 | 0 | 0 | 0 | 1 |
| *NA* | lmo1686 | similar to hypothetical proteins | Conserved | -1.48 | 0 | 0 | 0 | 1.1 |
| *NA* | lmo2767 | unknown | Conserved | -1.48 | 0 | 0 | 0 | -2.57 |
| *NA* | lmo1541 | similar to unknown protein | Conserved | -1.47 | 0 | 0 | 0 | -1.39 |
| *NA* | lmo2388 | similar to B. subtilis YwqG protein | Conserved | -1.46 | 0 | 0 | 0 | 0.34 |
| *NA* | lmo2169 | unknown | NA | -1.46 | 0 | 0 | 1.1 | -0.14 |
| *NA* | lmo1243 | unknown | Conserved | -1.45 | 0 | 0 | 0 | 0.16 |
| *NA* | LMOf2365_0510 | conserved hypothetical protein | Conserved | -1.43 | 0 | 0 | 0 | 0 |
| *NA* | lmo0349 | unknown | NA | -1.43 | 0 | 0 | 0 | 0.45 |
| *NA* | lmo1485 | similar to unknown proteins | Conserved | -1.42 | 0 | 0 | 0 | 1.24 |
| *NA* | LMOf2365_0252 | conserved hypothetical protein | Conserved | -1.41 | 0 | 0 | 0 | 0 |
| *NA* | LMOf2365_1108 | conserved hypothetical protein | Conserved | -1.41 | 0 | 0 | 0 | 0 |
| *NA* | lmo2707 | unknown | Conserved | -1.4 | 0 | 0 | 0 | 1.02 |
| *NA* | lmo0719 | similar to unknown protein | Conserved | -1.4 | UP | 0 | 0 | 0.78 |
| *NA* | lmo1951 | similar to unknown proteins | Conserved | -1.4 | 0 | 0 | 0 | 0.82 |
| *NA* | lmo1332 | similar to conserved hypothetical proteins | Conserved | -1.39 | 0 | 0 | 0 | 0.46 |
| *NA* | lmo0729 | unknown | NA | -1.39 | 0 | 0 | 0 | 0.85 |
| *NA* | lmo2177 | similar to unknown protein | Conserved | -1.38 | 0 | 0 | 0 | 0.51 |
| *NA* | lmo2562 | unknown | Conserved | -1.38 | 0 | 0 | 0 | 1.1 |
| *NA* | lmo2354 | conserved hypothetical protein | Conserved | -1.37 | 0 | 0 | 0 | -2.2 |
| *NA* | lmo1916 | similar to peptidase | Conserved | -1.37 | 0 | 0 | 0 | 0.37 |
| *NA* | LMOf2365_0301 | conserved hypothetical protein | Conserved | -1.36 | 0 | 0 | 0 | 0 |
| *NA* | lmo0413 | unknown | NA | -1.36 | 0 | 0 | 0 | 2.4 |
| *NA* | lmo2465 | unknown | NA | -1.36 | 0 | 0 | 0 | -1.52 |
| *NA* | lmo2078 | similar to unknown proteins | Conserved | -1.35 | 0 | 0 | 0 | 0.16 |
| *NA* | lmo0442 | unknown | NA | -1.33 | 0 | 0 | 0 | -0.87 |
| *NA* | lmo0403 | unknown | NA | -1.32 | 0 | 0 | 0 | -0.52 |
| *NA* | lmo2706 | unknown | NA | -1.31 | 0 | 0 | 0 | -1.45 |
| *NA* | lmo1344 | similar to comG operon protein 4 (comGD) | Conserved | -1.31 | 0 | 0 | 0 | -1.04 |
| *NA* | lmo1242 | similar to B. subtilis YdeI protein | Conserved | -1.3 | 0 | 0 | 0 | -0.64 |
| *NA* | lmo2705 | unknown | Conserved | -1.23 | 0 | 0 | 0 | -0.77 |
| *NA* | lmo0592 | unknown | Conserved | -1.2 | 0 | 3.39 | 2 | 2.61 |
| *NA* | lmo0019 | unknown | Conserved | 0 | UP | 2.08 | 5.1 | -1.27 |
| *NA* | lmo0025 | similar to phosphoheptose isomerase | Conserved | 0 | UP | 0 | 0 | -0.87 |
| *NA* | lmo0038 | conserved hypothetical protein | Conserved | 0 | UP | 0 | 0 | 2.48 |
| *NA* | lmo0047 | unknown | Conserved | 0 | UP | 2.76 | -2 | -1.4 |
| *NA* | lmo0080 | unknown | NA | 0 | UP | 0 | 0 | 1.89 |
| *NA* | lmo0081 | unknown | NA | 0 | UP | 0 | 0 | -2.22 |
| *NA* | lmo0082 | unknown | NA | 0 | UP | 0 | 0 | -0.4 |
| *NA* | lmo0094 | unknown | NA | 0 | UP | 0 | 0 | -0.5 |
| *NA* | lmo0133 | similar to E. coli YjdI protein | Conserved | 0 | UP | 0 | 2.4 | -0.74 |
| *NA* | lmo0149 | unknown | NA | 0 | UP | 0 | 0 | -0.55 |
| *NA* | lmo0170 | unknown | Conserved | 0 | UP | 0 | 4 | 0.35 |
| *NA* | lmo0439 | weakly similar to a module of peptide synthetase | Conserved | 0 | UP | 0 | 0 | 0.81 |
| *NA* | lmo0459 | similar to transcription regulator (VirR from Streptococcus pyogenes) | NA | 0 | UP | 0 | 0 | -1.28 |
| *NA* | lmo0496 | similar to B. subtilis YnzC protein | Conserved | 0 | UP | 0 | -2.1 | -0.39 |
| *NA* | lmo0576 | hypothetical cell wall associated protein | Conserved | 0 | UP | 0 | 0 | 0.96 |
| *NA* | lmo0579 | similar to unknown protein | Conserved | 0 | UP | 0 | 0 | -1.1 |
| *NA* | lmo0589 | unknown | Conserved | 0 | UP | 0 | 3.9 | 0.03 |
| *NA* | lmo0596 | similar to unknown proteins | Conserved | 0 | UP | 0 | 17.7 | 0.46 |
| *NA* | lmo0628 | unknown | NA | 0 | UP | 0 | 2.2 | -5.21 |
| *NA* | lmo0642 | unknown | Conserved | 0 | UP | 0 | 0 | 0.34 |
| *NA* | lmo0670 | unknown | Conserved | 0 | UP | 0 | 12.8 | 2.15 |
| *NA* | lmo0724 | similar to B. subtilis YvpB protein | Conserved | 0 | UP | 0 | -1.3 | 2.03 |
| *NA* | lmo0748 | unknown | NA | 0 | UP | 0 | 0 | -0.06 |
| *NA* | lmo0750 | unknown | NA | 0 | UP | 0 | 0 | 0.93 |
| *NA* | lmo0751 | unknown | NA | 0 | UP | 0 | 0 | 1.85 |
| *NA* | lmo0761 | similar to unknown proteins | Conserved | 0 | UP | 0 | 0 | 1.05 |
| *NA* | lmo0794 | similar to B. subtilis YwnB protein | Conserved | 0 | UP | 0 | 2.3 | 0.9 |
| *NA* | lmo0863 | unknown | NA | 0 | UP | 0 | 0 | -0.17 |
| *NA* | lmo0868 | unknown | NA | 0 | UP | 0 | 0 | 0.22 |
| *NA* | lmo0869 | unknown | Conserved | 0 | UP | 0 | 0 | 0.18 |
| *NA* | lmo0879 | unknown | Conserved | 0 | UP | 0 | 0 | -0.44 |
| *NA* | lmo0881 | unknown | NA | 0 | UP | 0 | 0 | -0.15 |
| *NA* | lmo0905 | unknown | NA | 0 | UP | 2.45 | 0 | -0.17 |
| *NA* | lmo0937 | unknown | NA | 0 | UP | 0 | 3.3 | 0.02 |
| *NA* | lmo0944 | similar to B. subtilis YneR protein | Conserved | 0 | UP | 0 | 0 | -1.7 |
| *NA* | lmo0953 | unknown | NA | 0 | UP | 0 | 3 | -1.1 |
| *NA* | lmo0994 | unknown | NA | 0 | UP | 0 | 7.6 | -0.24 |
| *NA* | lmo1127 | unknown | Conserved | 0 | UP | 0 | 0 | 0.7 |
| *NA* | lmo1137 | unknown | Conserved | 0 | UP | 0 | 0 | -3.07 |
| *NA* | lmo1139 | unknown | NA | 0 | UP | 0 | 0 | 0.75 |
| *NA* | lmo1140 | unknown | Conserved | 0 | UP | 0 | 4.5 | -0.04 |
| *NA* | lmo1241 | unknown | Conserved | 0 | UP | 0 | 2.3 | 0.2 |
| *NA* | lmo1264 | unknown | Conserved | 0 | UP | 0 | 0 | 0.81 |
| *NA* | lmo1432 | unknown | NA | 0 | UP | 0 | 1.8 | 1 |
| *NA* | lmo1526 | similar to unknown proteins | Conserved | 0 | UP | 0 | 2.6 | 0.92 |
| *NA* | lmo1637 | similar to membrane proteins | Conserved | 0 | UP | 0 | 0 | 0.61 |
| *NA* | lmo1972 | similar to pentitol PTS system enzyme II B component | Conserved | 0 | UP | 0 | 0 | 3.58 |
| *NA* | lmo2031 | similar to unknown proteins | Conserved | 0 | UP | 0 | 0 | -0.31 |
| *NA* | lmo2056 | similar to unknown proteins | Conserved | 0 | UP | 0 | 0 | -2.9 |
| *NA* | lmo2083 | unknown | Conserved | 0 | UP | 0 | 0 | -1.29 |
| *NA* | lmo2132 | unknown | NA | 0 | UP | 0 | 1.8 | -1.09 |
| *NA* | lmo2210 | unknown | NA | 0 | UP | 0 | 0 | -1.07 |
| *NA* | lmo2213 | similar to unknown protein | Conserved | 0 | UP | 0 | 14.1 | -1.24 |
| *NA* | lmo2258 | unknown | NA | 0 | UP | 0 | 0 | -0.07 |
| *NA* | lmo2276 | similar to an unknown bacteriophage protein | Conserved | 0 | UP | 0 | 0 | -1.72 |
| *NA* | lmo2302 | unknown | NA | 0 | UP | 0 | 0 | -1.17 |
| *NA* | lmo2305 | unknown | NA | 0 | UP | 0 | 0 | -0.05 |
| *NA* | lmo2309 | unknown | NA | 0 | UP | 0 | 0 | -0.52 |
| *NA* | lmo2313 | similar to a bacteriophage protein | Conserved | 0 | UP | 0 | 0 | 0.5 |
| *NA* | lmo2356 | unknown | NA | 0 | UP | 0 | 0 | 0.32 |
| *NA* | lmo2387 | conserved hypothetical protein | Conserved | 0 | UP | 0 | 1.2 | 1.97 |
| *NA* | lmo2391 | conserved hypothetical protein similar to B. subtilis YhfK protein | Conserved | 0 | UP | 0 | 5.7 | 0.41 |
| *NA* | lmo2432 | unknown | NA | 0 | UP | 0 | 0 | 0.42 |
| *NA* | lmo2454 | unknown | NA | 0 | UP | 0 | 1.6 | 1.97 |
| *NA* | lmo2568 | unknown | Conserved | 0 | UP | 0 | 0 | -0.12 |
| *NA* | lmo2570 | unknown | Conserved | 0 | UP | 0 | 3.1 | 0.02 |
| *NA* | lmo2572 | similar to Chain A, Dihydrofolate Reductase | Conserved | 0 | UP | 0 | 4.4 | 0.64 |
| *NA* | lmo2585 | similar to B. subtilis YrhD protein | Conserved | 0 | UP | 0 | 0 | 0.86 |
| *NA* | lmo2603 | unknown | Conserved | 0 | UP | 0 | 2.9 | -1.44 |
| *NA* | lmo2669 | unknown | Conserved | 0 | UP | 0 | 0 | 1.32 |
| *NA* | lmo2675 | unknown | Conserved | 0 | UP | -2.55 | 0 | 1.21 |
| *NA* | lmo2748 | similar to B. subtilis stress protein YdaG | Conserved | 0 | UP | 0 | 15.6 | 1.41 |
| *NA* | lmo0049 | unknown | NA | 0 | DOWN | 0 | 0 | -2.28 |
| *NA* | lmo0449 | unknown | Conserved | 0 | DOWN | 0 | 0 | -0.43 |
| *NA* | lmo1401 | conserved hypothetical protein | Conserved | 0 | DOWN | 0 | 0 | -0.37 |
| *NA* | lmo1402 | similar to B. subtilis YmcA protein | Conserved | 0 | DOWN | 0 | 0 | 1.53 |
| *NA* | lmo1461 | unknown | NA | 0 | DOWN | 0 | 0 | -0.79 |
| *NA* | lmo1488 | similar to unknown proteins | Conserved | 0 | DOWN | 0 | 0 | 3 |
| *NA* | lmo1492 | similar to unknown proteins | Conserved | 0 | DOWN | 0 | 0 | -1.15 |
| *NA* | lmo1597 | unknown | NA | 0 | DOWN | 0 | 0 | 1.37 |
| *NA* | lmo1662 | similar to conserved hypothetical protein | Conserved | 0 | DOWN | 0 | 0 | -0.33 |
| *NA* | lmo1828 | similar to conserved hypothetical protein | Conserved | 0 | DOWN | 0 | 0 | -1.49 |
| *NA* | lmo1943 | similar to unknown proteins | Conserved | 0 | DOWN | 0 | 0 | 0.28 |
| *NA* | lmo2079 | unknown | NA | 0 | DOWN | 0 | 0 | 0.09 |
| *NA* | lmo2129 | unknown | NA | 0 | DOWN | 0 | 0 | -0.16 |
| *NA* | lmo2149 | similar to unknown proteins | Conserved | 0 | DOWN | 0 | 0 | -1.16 |
| *NA* | lmo2156 | unknown | NA | 0 | DOWN | 0 | 0 | -1.75 |
| *NA* | lmo2197 | unknown | NA | 0 | DOWN | 0 | 0 | -0.55 |
| *NA* | lmo2277 | unknown | NA | 0 | DOWN | 0 | 0 | -0.2 |
| *NA* | lmo2409 | unknown | NA | 0 | DOWN | 0 | 0 | -0.52 |
| *NA* | lmo2806 | hypothetical secreted protein | Conserved | 0 | DOWN | 0 | 0 | -1.42 |
| *NA* | lmo0119 | unknown | Conserved | 0 | 0 | 1.22 | 0 | -1.5 |
| *NA* | lmo0120 | unknown | Conserved | 0 | 0 | 2.18 | 0 | -1.67 |
| *NA* | lmo0121 | similar to bacteriophage minor tail proteins | Conserved | 0 | 0 | 1.07 | 0 | 0.79 |
| *NA* | lmo0123 | similar to protein gp18 from Bacteriophage A118 | Conserved | 0 | 0 | 3.04 | 0 | -1.48 |
| *NA* | lmo0124 | unknown | NA | 0 | 0 | 2.95 | 0 | 0.08 |
| *NA* | lmo0125 | unknown | NA | 0 | 0 | 3.08 | 0 | -0.83 |
| *NA* | lmo0126 | unknown | Conserved | 0 | 0 | 2.93 | 0 | 0.88 |
| *NA* | lmo0127 | weakly similar to protein gp20 from Bacteriophage A118 | NA | 0 | 0 | 2.97 | 0 | -1.2 |
| *NA* | lmo0128 | similar to a protein from Bacteriophage phi-105 (ORF 45) | Conserved | 0 | 0 | 1.87 | 0 | -1.36 |
| *NA* | lmo0391 | unknown | NA | 0 | 0 | 3.78 | 0 | 3.86 |
| *NA* | lmo0392 | highly similar to B. subtilis YqfA protein | Conserved | 0 | 0 | 3.05 | 0 | 2.79 |
| *NA* | lmo0393 | unknown | NA | 0 | 0 | 3.66 | 0 | 1.89 |
| *NA* | lmo0525 | unknown | Conserved | 0 | 0 | 2.54 | 0 | -2.17 |
| *NA* | lmo0581 | conserved hypothetical protein | Conserved | 0 | 0 | 4.71 | 0 | 1.72 |
| *NA* | lmo0604 | similar to B. subtilis YvlA protein | Conserved | 0 | 0 | 2.83 | 0 | 1.35 |
| *NA* | lmo0675 | unknown | NA | 0 | 0 | 1.44 | 1.8 | 0.47 |
| *NA* | lmo0684 | unknown | NA | 0 | 0 | 3.19 | -2 | 3.12 |
| *NA* | lmo0687 | unknown | NA | 0 | 0 | 2.46 | 0 | 1.23 |
| *NA* | lmo0850 | hypothetical protein | NA | 0 | 0 | 4.48 | 0 | -1.72 |
| *NA* | lmo0984 | weakly similar to two-component response regulator | Conserved | 0 | 0 | 2.27 | 0 | -0.63 |
| *NA* | lmo0985 | unknown | NA | 0 | 0 | 3.56 | 0 | -1.08 |
| *NA* | lmo1416 | unknown | Conserved | 0 | 0 | 3.01 | 0 | -0.32 |
| *NA* | lmo1487 | similar to unknown proteins | Conserved | 0 | 0 | 2.34 | 0 | 0.92 |
| *NA* | lmo2375 | unknown | NA | 0 | 0 | 2.41 | 0 | -1.68 |
| *NA* | lmo2852 | unknown | Conserved | 0 | 0 | 3.74 | 0 | 0 |
| *NA* | lmo0029 | unknown | Conserved | 0 | 0 | -2.25 | 0 | 0.18 |
| *NA* | lmo0104 | unknown | NA | 0 | 0 | -2.6 | 0 | -0.56 |
| *NA* | lmo0903 | conserved hypothetical protein | Conserved | 0 | 0 | -2.62 | 0 | -2.1 |
| *NA* | lmo0653 | unknown | Conserved | 0 | 0 | 0 | 2.3 | 0.64 |
| *NA* | lmo0293 | conserved hypothetical protein, highly similar to B. subtilis YydA proteinYyd | Conserved | 0 | 0 | 0 | 2.4 | -1.89 |
| *NA* | lmo0995 | similar to B. subtilis YkrP protein | Conserved | 0 | 0 | 0 | 3.9 | -0.97 |
| *NA* | lmo1028 | similar to B. subtilis YkzG protein | Conserved | 0 | 0 | 0 | 1.7 | -2.24 |
| *NA* | lmo1261 | unknown | Conserved | 0 | 0 | 0 | 1.9 | -0.08 |
| *NA* | lmo1830 | similar to conserved hypotheticl proteins | Conserved | 0 | 0 | 0 | 2 | 0.74 |
| *NA* | lmo2160 | similar to unknown proteins | Conserved | 0 | 0 | 0 | 1 | -1.21 |
| *NA* | lmo2161 | unknown | Conserved | 0 | 0 | 0 | -2 | -0.25 |
| *NA* | lmo0715 | unknown | NA | 0 | 0 | 0 | -2.1 | -0.57 |
| *NA* | lmo0166 | similar to B. subtilis YazA protein | Conserved | 0 | 0 | 0 | -1.6 | -1.13 |
| *NA* | lmo0620 | unknown | Conserved | 0 | 0 | 0 | -2 | -2.98 |
| *NA* | lmo0703 | unknown | NA | 0 | 0 | 0 | -2.8 | 0.02 |
| *NA* | lmo0704 | unknown | NA | 0 | 0 | 0 | -2.7 | 0.23 |
| *NA* | lmo0718 | unknown | NA | 0 | 0 | 0 | -2.6 | 0.46 |
| *NA* | lmo1007 | unknown | NA | 0 | 0 | 0 | -2.5 | -1.29 |
| *NA* | lmo1257 | unknown | NA | 0 | 0 | 0 | -1.4 | 2.11 |
| *NA* | lmo1306 | highly similar to B. subtilis YneF protein | NA | 0 | 0 | 0 | -2.7 | 1.05 |
| *NA* | lmo2181 | similar to unknown protein | Conserved | 0 | 0 | 0 | -3.8 | 1.02 |
| *NA* | lmo0040 | conserved hypothetical protein | Conserved | 0 | 0 | 0 | 0 | 0.06 |
| *NA* | lmo0056 | similar to a small heat shock protein of Clostridium acetobutylicum | Conserved | 0 | 0 | 0 | 0 | 0.93 |
| *NA* | lmo0057 | similar to B. subtilis YueB protein | Conserved | 0 | 0 | 0 | 0 | -0.42 |
| *NA* | lmo0058 | unknown | NA | 0 | 0 | 0 | 0 | -0.82 |
| *NA* | lmo0059 | similar to B. subtilis YukD protein | Conserved | 0 | 0 | 0 | 0 | -0.18 |
| *NA* | lmo0060 | similar to B. subtilis YukC protein | Conserved | 0 | 0 | 0 | 0 | -0.85 |
| *NA* | lmo0063 | unknown | Conserved | 0 | 0 | 0 | 0 | 1.81 |
| *NA* | lmo0064 | unknown | NA | 0 | 0 | 0 | 0 | 0.37 |
| *NA* | lmo0066 | similar to toxin components | Conserved | 0 | 0 | 0 | 0 | 1.17 |
| *NA* | lmo0068 | unknown | NA | 0 | 0 | 0 | 0 | -1.85 |
| *NA* | lmo0069 | unknown | NA | 0 | 0 | 0 | 0 | 0.99 |
| *NA* | lmo0070 | unknown | NA | 0 | 0 | 0 | 0 | 0.16 |
| *NA* | lmo0071 | unknown | NA | 0 | 0 | 0 | 0 | -1.09 |
| *NA* | lmo0072 | Hypothetical | NA | 0 | 0 | 0 | 0 | 1.41 |
| *NA* | lmo0073 | unknown | NA | 0 | 0 | 0 | 0 | -1.57 |
| *NA* | lmo0074 | unknown | NA | 0 | 0 | 0 | 0 | -2.39 |
| *NA* | lmo0075 | similar to carboxyphosphonoenolpyruvate phosphonomutase | Conserved | 0 | 0 | 0 | 0 | -1.04 |
| *NA* | lmo0079 | unknown | NA | 0 | 0 | 0 | 0 | 1.72 |
| *NA* | lmo0085 | unknown | NA | 0 | 0 | 0 | 0 | -1.31 |
| *NA* | lmo0086 | unknown | NA | 0 | 0 | 0 | 0 | 1.54 |
| *NA* | lmo0087 | unknown | NA | 0 | 0 | 0 | 0 | -0.47 |
| *NA* | lmo0095 | unknown | NA | 0 | 0 | 0 | 0 | -0.85 |
| *NA* | lmo0112 | weakly similar to transcription regulators, Fnr/Crp family | NA | 0 | 0 | 0 | 0 | -0.78 |
| *NA* | lmo0138 | unknown | NA | 0 | 0 | 0 | 0 | -0.22 |
| *NA* | lmo0140 | unknown | NA | 0 | 0 | 0 | 0 | -1.88 |
| *NA* | lmo0141 | unknown | NA | 0 | 0 | 0 | 0 | 0.49 |
| *NA* | lmo0142 | unknown | NA | 0 | 0 | 0 | 0 | 1.14 |
| *NA* | lmo0143 | unknown | NA | 0 | 0 | 0 | 0 | 0.08 |
| *NA* | lmo0144 | unknown | NA | 0 | 0 | 0 | 0 | -1.07 |
| *NA* | lmo0145 | hypothetical protein | NA | 0 | 0 | 0 | 0 | -1.42 |
| *NA* | lmo0146 | hypothetical protein | NA | 0 | 0 | 0 | 0 | -0.22 |
| *NA* | lmo0147 | unknown | NA | 0 | 0 | 0 | 0 | -0.68 |
| *NA* | lmo0148 | unknown | NA | 0 | 0 | 0 | 0 | -0.01 |
| *NA* | lmo0150 | unknown | NA | 0 | 0 | 0 | 0 | -1.01 |
| *NA* | lmo0151 | unknown | NA | 0 | 0 | 0 | 0 | -1.54 |
| *NA* | lmo0156 | unknown | NA | 0 | 0 | 0 | 0 | -1.34 |
| *NA* | lmo0164 | similar to B. subtilis YabA protein | Conserved | 0 | 0 | 0 | 0 | -0.73 |
| *NA* | lmo0165 | conserved hypothetical protein | Conserved | 0 | 0 | 0 | 0 | -1.66 |
| *NA* | lmo0167 | conserved hypothetical protein | Conserved | 0 | 0 | 0 | 0 | -0.97 |
| *NA* | lmo0193 | unknown | Conserved | 0 | 0 | 0 | 0 | -1.41 |
| *NA* | lmo0206 | unknown | Conserved | 0 | 0 | 0 | 0 | -0.32 |
| *NA* | lmo0209 | unknown | Conserved | 0 | 0 | 0 | 0 | -0.01 |
| *NA* | lmo0229 | highly similar to transcription repressor of class III stress genes (CtsR) | Conserved | 0 | 0 | 0 | 0 | 0.68 |
| *NA* | lmo0242 | similar to B. subtilis Yacp protein | Conserved | 0 | 0 | 0 | 0 | -1.41 |
| *NA* | lmo0254 | unknown | NA | 0 | 0 | 0 | 0 | -1.97 |
| *NA* | lmo0256 | conserved hypothetical protein | Conserved | 0 | 0 | 0 | 0 | -0.59 |
| *NA* | lmo0260 | similar to unknown proteins | Conserved | 0 | 0 | 0 | 0 | -1.83 |
| *NA* | lmo0274 | unknown | NA | 0 | 0 | 0 | 0 | -0.89 |
| *NA* | lmo0281 | unknown | NA | 0 | 0 | 0 | 0 | 1.58 |
| *NA* | lmo0296 | unknown | Conserved | 0 | 0 | 0 | 0 | -1.77 |
| *NA* | lmo0302 | unknown | NA | 0 | 0 | 0 | 0 | -2.41 |
| *NA* | lmo0303 | putaive secreted, lysin rich protein | NA | 0 | 0 | 0 | 0 | -1.02 |
| *NA* | lmo0304 | unknown | Conserved | 0 | 0 | 0 | 0 | 0.27 |
| *NA* | lmo0306 | unknown | Conserved | 0 | 0 | 0 | 0 | -0.9 |
| *NA* | lmo0307 | unknown | NA | 0 | 0 | 0 | 0 | 0.49 |
| *NA* | lmo0309 | similar to unknown protein | Conserved | 0 | 0 | 0 | 0 | -0.53 |
| *NA* | lmo0310 | unknown | NA | 0 | 0 | 0 | 0 | -0.19 |
| *NA* | lmo0311 | unknown | NA | 0 | 0 | 0 | 0 | 1.35 |
| *NA* | lmo0312 | similar to unknown proteins | Conserved | 0 | 0 | 0 | 0 | -0.01 |
| *NA* | lmo0322 | similar to unknown proteins | Conserved | 0 | 0 | 0 | 0 | 1.04 |
| *NA* | lmo0323 | similar to unknown proteins | Conserved | 0 | 0 | 0 | 0 | -0.01 |
| *NA* | lmo0324 | unknown | NA | 0 | 0 | 0 | 0 | -1.1 |
| *NA* | lmo0328 | unknown | NA | 0 | 0 | 0 | 0 | -0.46 |
| *NA* | lmo0334 | unknown | NA | 0 | 0 | 0 | 0 | 0.8 |
| *NA* | lmo0335 | unknown | NA | 0 | 0 | 0 | 0 | 0.04 |
| *NA* | lmo0337 | unknown | NA | 0 | 0 | 0 | 0 | -0.16 |
| *NA* | lmo0338 | unknown | NA | 0 | 0 | 0 | 0 | -1.93 |
| *NA* | lmo0340 | unknown | Conserved | 0 | 0 | 0 | 0 | 1.32 |
| *NA* | lmo0350 | unknown | NA | 0 | 0 | 0 | 0 | -0.04 |
| *NA* | lmo0351 | similar to unknown proteins | Conserved | 0 | 0 | 0 | 0 | 1.32 |
| *NA* | lmo0363 | similar to Salmonella typhimurium peptidase E | Conserved | 0 | 0 | 0 | 0 | -0.28 |
| *NA* | lmo0364 | similar to transcription regulator | Conserved | 0 | 0 | 0 | 0 | -0.54 |
| *NA* | lmo0365 | similar to conserved hypothetical protein | Conserved | 0 | 0 | 0 | 0 | 0.97 |
| *NA* | lmo0375 | unknown | NA | 0 | 0 | 0 | 0 | 0.65 |
| *NA* | lmo0376 | similar to putative transcription regulator | Conserved | 0 | 0 | 0 | 0 | 2.24 |
| *NA* | lmo0377 | unknown | NA | 0 | 0 | 0 | 0 | -0.36 |
| *NA* | lmo0378 | unknown | NA | 0 | 0 | 0 | 0 | 1.72 |
| *NA* | lmo0379 | unknown | NA | 0 | 0 | 0 | 0 | 2.09 |
| *NA* | lmo0380 | unknown | NA | 0 | 0 | 0 | 0 | 0.56 |
| *NA* | lmo0381 | unknown | NA | 0 | 0 | 0 | 0 | 0.44 |
| *NA* | lmo0388 | unknown | NA | 0 | 0 | 0 | 0 | 0.6 |
| *NA* | lmo0397 | similar to unknown proteins | Conserved | 0 | 0 | 0 | 0 | -0.22 |
| *NA* | lmo0404 | unknown | NA | 0 | 0 | 0 | 0 | 0.06 |
| *NA* | lmo0407 | unknown | Conserved | 0 | 0 | 0 | 0 | -0.41 |
| *NA* | lmo0412 | unknown | NA | 0 | 0 | 0 | 0 | 0.24 |
| *NA* | lmo0417 | unknown | NA | 0 | 0 | 0 | 0 | 2.09 |
| *NA* | lmo0418 | unknown | NA | 0 | 0 | 0 | 0 | 0.28 |
| *NA* | lmo0422 | similar to unknown protein | Conserved | 0 | 0 | 0 | 0 | 2.11 |
| *NA* | lmo0437 | conserved hypothetical protein | Conserved | 0 | 0 | 0 | 0 | 1.9 |
| *NA* | lmo0438 | unknown | NA | 0 | 0 | 0 | 0 | -0.52 |
| *NA* | lmo0440 | unknown | NA | 0 | 0 | 0 | 0 | -0.5 |
| *NA* | lmo0450 | similar to unknown proteins | Conserved | 0 | 0 | 0 | 0 | -0.01 |
| *NA* | lmo0451 | unknown | NA | 0 | 0 | 0 | 0 | -0.86 |
| *NA* | lmo0455 | similar to unknown proteins | Conserved | 0 | 0 | 0 | 0 | -0.01 |
| *NA* | lmo0457 | similar to unknown proteins | Conserved | 0 | 0 | 0 | 0 | 2.31 |
| *NA* | lmo0460 | putative membrane associated lipoprotein | Conserved | 0 | 0 | 0 | 0 | 4.71 |
| *NA* | lmo0461 | unknown | NA | 0 | 0 | 0 | 0 | 3.72 |
| *NA* | lmo0462 | unknown | NA | 0 | 0 | 0 | 0 | 1.54 |
| *NA* | lmo0465 | Hypothetical orf | NA | 0 | 0 | 0 | 0 | 0.65 |
| *NA* | lmo0467 | unknown | Conserved | 0 | 0 | 0 | 0 | 0.16 |
| *NA* | lmo0468 | unknown | NA | 0 | 0 | 0 | 0 | -0.21 |
| *NA* | lmo0469 | unknown | NA | 0 | 0 | 0 | 0 | -2.92 |
| *NA* | lmo0471 | unknown | NA | 0 | 0 | 0 | 0 | -0.35 |
| *NA* | lmo0472 | unknown | NA | 0 | 0 | 0 | 0 | -0.47 |
| *NA* | lmo0474 | unknown | NA | 0 | 0 | 0 | 0 | -0.66 |
| *NA* | lmo0475 | unknown | NA | 0 | 0 | 0 | 0 | -0.65 |
| *NA* | lmo0477 | putative secreted protein | Conserved | 0 | 0 | 0 | 0 | 1.53 |
| *NA* | lmo0478 | putative secreted protein | NA | 0 | 0 | 0 | 0 | 0.27 |
| *NA* | lmo0479 | putative secreted protein | Conserved | 0 | 0 | 0 | 0 | 0.34 |
| *NA* | lmo0504 | unknown | NA | 0 | 0 | 0 | 0 | 0.81 |
| *NA* | lmo0510 | unknown | NA | 0 | 0 | 0 | 0 | 1.78 |
| *NA* | lmo0523 | similar to B. subtilis YybC protein | Conserved | 0 | 0 | 0 | 0 | 0.51 |
| *NA* | lmo0527 | transmembrane protein | Conserved | 0 | 0 | 0 | 0 | -0.92 |
| *NA* | lmo0528 | hypothetical secreted protein | Conserved | 0 | 0 | 0 | 0 | 1.83 |
| *NA* | lmo0530 | unknown | Conserved | 0 | 0 | 0 | 0 | 1.14 |
| *NA* | lmo0532 | unknown | NA | 0 | 0 | 0 | 0 | 0.35 |
| *NA* | lmo0534 | similar to unknown proteins | Conserved | 0 | 0 | 0 | 0 | 0.67 |
| *NA* | lmo0545 | unknown | Conserved | 0 | 0 | 0 | 0 | 1.35 |
| *NA* | lmo0546 | similar to putative NAD(P)-dependent oxidoreductase | Conserved | 0 | 0 | 0 | 0 | 2.4 |
| *NA* | lmo0548 | unknown | NA | 0 | 0 | 0 | 0 | 0.82 |
| *NA* | lmo0551 | unknown | Conserved | 0 | 0 | 0 | 0 | 1.33 |
| *NA* | lmo0552 | similar to unknown protein | Conserved | 0 | 0 | 0 | 0 | -1.38 |
| *NA* | lmo0558 | conserved hypothetical protein | Conserved | 0 | 0 | 0 | 0 | 2.65 |
| *NA* | lmo0572 | unknown | Conserved | 0 | 0 | 0 | 0 | 1.44 |
| *NA* | lmo0578 | putative conserved membrane protein | Conserved | 0 | 0 | 0 | 0 | -0.26 |
| *NA* | lmo0585 | putative secreted protein | Conserved | 0 | 0 | 0 | 0 | 0.01 |
| *NA* | lmo0587 | putative secreted protein | NA | 0 | 0 | 0 | 0 | 0.17 |
| *NA* | lmo0599 | conserved hypothetical protein | Conserved | 0 | 0 | 0 | 0 | 2.37 |
| *NA* | lmo0600 | unknown | Conserved | 0 | 0 | 0 | 0 | -0.07 |
| *NA* | lmo0615 | unknown | NA | 0 | 0 | 0 | 0 | 0.26 |
| *NA* | lmo0617 | unknown | NA | 0 | 0 | 0 | 0 | 0.51 |
| *NA* | lmo0618 | similar to protein kinase | Conserved | 0 | 0 | 0 | 0 | -0.23 |
| *NA* | lmo0619 | unknown | NA | 0 | 0 | 0 | 0 | -1.08 |
| *NA* | lmo0626 | similar to unknown protein | Conserved | 0 | 0 | 0 | 0 | 0.01 |
| *NA* | lmo0634 | similar to an E. coli putative tagatose 6-phosphate kinase | NA | 0 | 0 | 0 | 0 | 1.11 |
| *NA* | lmo0638 | unknown | NA | 0 | 0 | 0 | 0 | 0.79 |
| *NA* | lmo0656 | conserved hypothetical protein | Conserved | 0 | 0 | 0 | 0 | -1.63 |
| *NA* | lmo0657 | unknown | NA | 0 | 0 | 0 | 0 | -1.17 |
| *NA* | lmo0671 | unknown | Conserved | 0 | 0 | 0 | 0 | -2.53 |
| *NA* | lmo0672 | similar to unknown protein | Conserved | 0 | 0 | 0 | 0 | -1.59 |
| *NA* | lmo0673 | unknown | NA | 0 | 0 | 0 | 0 | -1.66 |
| *NA* | lmo0709 | unknown | Conserved | 0 | 0 | 0 | 0 | 0.38 |
| *NA* | lmo0730 | unknown | Conserved | 0 | 0 | 0 | 0 | 1.2 |
| *NA* | lmo0737 | unknown | Conserved | 0 | 0 | 0 | 0 | 0.25 |
| *NA* | lmo0743 | unknown | NA | 0 | 0 | 0 | 0 | 0.62 |
| *NA* | lmo0746 | hypothetical | NA | 0 | 0 | 0 | 0 | 1.67 |
| *NA* | lmo0765 | unknown | Conserved | 0 | 0 | 0 | 0 | 1.65 |
| *NA* | lmo0771 | unknown | NA | 0 | 0 | 0 | 0 | -0.36 |
| *NA* | lmo0775 | unknown | NA | 0 | 0 | 0 | 0 | 0.19 |
| *NA* | lmo0778 | unknown | NA | 0 | 0 | 0 | 0 | -0.09 |
| *NA* | lmo0779 | unknown | Conserved | 0 | 0 | 0 | 0 | 0.36 |
| *NA* | lmo0780 | unknown | NA | 0 | 0 | 0 | 0 | -1.89 |
| *NA* | lmo0793 | similar to conserved hypothetical protein | Conserved | 0 | 0 | 0 | 0 | 1.25 |
| *NA* | lmo0804 | unknown | NA | 0 | 0 | 0 | 0 | 0.59 |
| *NA* | lmo0805 | unknown | NA | 0 | 0 | 0 | 0 | 0.87 |
| *NA* | lmo0824 | unknown | NA | 0 | 0 | 0 | 0 | -2.79 |
| *NA* | lmo0834 | unknown | NA | 0 | 0 | 0 | 0 | 0.41 |
| *NA* | lmo0843 | similar to B. subtilis protein YsdA | Conserved | 0 | 0 | 0 | 0 | -0.56 |
| *NA* | lmo0851 | unknown | Conserved | 0 | 0 | 0 | 0 | -1.27 |
| *NA* | lmo0864 | unknown | Conserved | 0 | 0 | 0 | 0 | -1.08 |
| *NA* | lmo0870 | unknown | Conserved | 0 | 0 | 0 | 0 | 1.83 |
| *NA* | lmo0900 | unknown | Conserved | 0 | 0 | 0 | 0 | -1.7 |
| *NA* | lmo0910 | unknown | Conserved | 0 | 0 | 0 | 0 | -4.04 |
| *NA* | lmo0920 | similar to B. subtilis YcgR protein | Conserved | 0 | 0 | 0 | 0 | -1.31 |
| *NA* | lmo0921 | similar to B. subtilis YcgQ protein | Conserved | 0 | 0 | 0 | 0 | -0.72 |
| *NA* | lmo0940 | unknown | Conserved | 0 | 0 | 0 | 0 | 1.25 |
| *NA* | lmo0951 | unknown | Conserved | 0 | 0 | 0 | 0 | -1.2 |
| *NA* | lmo0952 | unknown | NA | 0 | 0 | 0 | 0 | -0.35 |
| *NA* | lmo0965 | similar to B. subtilis YjbK protein | Conserved | 0 | 0 | 0 | 0 | -0.21 |
| *NA* | lmo0977 | similar to B. subtilis YjcH protein | Conserved | 0 | 0 | 0 | 0 | -1.77 |
| *NA* | lmo0982 | similar to glucanase and peptidase | Conserved | 0 | 0 | 0 | 0 | 1.64 |
| *NA* | lmo0999 | unknown | NA | 0 | 0 | 0 | 0 | -0.04 |
| *NA* | lmo1008 | similar to B. subtilis YkuJ protein | Conserved | 0 | 0 | 0 | 0 | -0.42 |
| *NA* | lmo1036 | unknown | NA | 0 | 0 | 0 | 0 | -0.61 |
| *NA* | lmo1037 | highly similar to B. subtilis YoaT protein | Conserved | 0 | 0 | 0 | 0 | 0.25 |
| *NA* | lmo1059 | unknown | Conserved | 0 | 0 | 0 | 0 | 0.79 |
| *NA* | lmo1065 | similar to B. subtilis YktB protein | Conserved | 0 | 0 | 0 | 0 | 0.65 |
| *NA* | lmo1069 | similar to B. subtilis YlaI protein | Conserved | 0 | 0 | 0 | 0 | -2.27 |
| *NA* | lmo1070 | similar to B. subtilis YlaN protein | Conserved | 0 | 0 | 0 | 0 | 2.59 |
| *NA* | lmo1094 | unknown | Conserved | 0 | 0 | 0 | 0 | -2.02 |
| *NA* | lmo1098 | highly similar to TN916 ORF8 | Conserved | 0 | 0 | 0 | 0 | 0.53 |
| *NA* | lmo1099 | similar to a protein encoded by Tn916 | Conserved | 0 | 0 | 0 | 0 | -0.04 |
| *NA* | lmo1103 | highly similar to TN916 ORF13 | Conserved | 0 | 0 | 0 | 0 | -2.52 |
| *NA* | lmo1105 | highly similar to TN916 ORF15 | Conserved | 0 | 0 | 0 | 0 | -0.62 |
| *NA* | lmo1106 | highly similar to TN916 ORF16 | Conserved | 0 | 0 | 0 | 0 | -0.36 |
| *NA* | lmo1107 | highly similar to TN916 ORF17 | Conserved | 0 | 0 | 0 | 0 | -0.31 |
| *NA* | lmo1108 | highly similar to TN916 ORF18 | Conserved | 0 | 0 | 0 | 0 | 0.44 |
| *NA* | lmo1109 | highly similar to TN916 ORF19 | Conserved | 0 | 0 | 0 | 0 | -0.8 |
| *NA* | lmo1110 | similar to unknown proteins | NA | 0 | 0 | 0 | 0 | 0.86 |
| *NA* | lmo1113 | highly similar to TN916 ORF22 | Conserved | 0 | 0 | 0 | 0 | -0.78 |
| *NA* | lmo1114 | highly similar to TN916 ORF23 | Conserved | 0 | 0 | 0 | 0 | -1.73 |
| *NA* | lmo1118 | unknown | NA | 0 | 0 | 0 | 0 | 0.13 |
| *NA* | lmo1120 | unknown | NA | 0 | 0 | 0 | 0 | -2.05 |
| *NA* | lmo1121 | unknown | NA | 0 | 0 | 0 | 0 | 1.13 |
| *NA* | lmo1122 | unknown | NA | 0 | 0 | 0 | 0 | -0.85 |
| *NA* | lmo1123 | unknown | NA | 0 | 0 | 0 | 0 | -1.03 |
| *NA* | lmo1124 | unknown | Conserved | 0 | 0 | 0 | 0 | 0.23 |
| *NA* | lmo1125 | unknown | Conserved | 0 | 0 | 0 | 0 | 0.57 |
| *NA* | lmo1128 | unknown | NA | 0 | 0 | 0 | 0 | -0.68 |
| *NA* | lmo1130 | similar to transcription regulators | NA | 0 | 0 | 0 | 0 | -1.11 |
| *NA* | lmo1133 | similar to B. subtilis YjcS protein | Conserved | 0 | 0 | 0 | 0 | -0.54 |
| *NA* | lmo1135 | unknown | NA | 0 | 0 | 0 | 0 | 1.11 |
| *NA* | lmo1146 | unknown | Conserved | 0 | 0 | 0 | 0 | -0.35 |
| *NA* | lmo1181 | similar to cobalamin adenosyl transferase | Conserved | 0 | 0 | 0 | 0 | -0.29 |
| *NA* | lmo1182 | similar to Salmonella enterica PduL protein | Conserved | 0 | 0 | 0 | 0 | 1.6 |
| *NA* | lmo1183 | unknown | Conserved | 0 | 0 | 0 | 0 | 0.59 |
| *NA* | lmo1187 | similar to ethanolamine utilization protein EutQ | Conserved | 0 | 0 | 0 | 0 | 0.58 |
| *NA* | lmo1190 | unknown | NA | 0 | 0 | 0 | 0 | -1.04 |
| *NA* | lmo1210 | similar to unknown proteins | Conserved | 0 | 0 | 0 | 0 | -0.34 |
| *NA* | lmo1212 | similar to unknown proteins | Conserved | 0 | 0 | 0 | 0 | 0.22 |
| *NA* | lmo1213 | similar to unknown protein | Conserved | 0 | 0 | 0 | 0 | -1.47 |
| *NA* | lmo1214 | unknown | NA | 0 | 0 | 0 | 0 | 1 |
| *NA* | lmo1229 | similar to B. subtilis YshA protein | Conserved | 0 | 0 | 0 | 0 | 0.31 |
| *NA* | lmo1247 | unknown | NA | 0 | 0 | 0 | 0 | 0.17 |
| *NA* | lmo1249 | unknown | NA | 0 | 0 | 0 | 0 | -0.38 |
| *NA* | lmo1265 | weakly similar to oligopeptide ABC transporter AppA (binding protein) | Conserved | 0 | 0 | 0 | 0 | 2.5 |
| *NA* | lmo1266 | unknown | Conserved | 0 | 0 | 0 | 0 | 1.31 |
| *NA* | lmo1281 | similar to B. subtilis YneP protein | Conserved | 0 | 0 | 0 | 0 | 0.49 |
| *NA* | lmo1282 | similar to B. subtilis YneQ protein | Conserved | 0 | 0 | 0 | 0 | 0.88 |
| *NA* | lmo1284 | conserved hypothetical protein, similar to B. subtilis YneS protein | Conserved | 0 | 0 | 0 | 0 | -0.29 |
| *NA* | lmo1307 | unknown | NA | 0 | 0 | 0 | 0 | 0.52 |
| *NA* | lmo1310 | similar to E. coli YbdN protein | Conserved | 0 | 0 | 0 | 0 | 0.13 |
| *NA* | lmo1312 | unknown | NA | 0 | 0 | 0 | 0 | 0.79 |
| *NA* | lmo1323 | similar to B. subtilis YlxR protein | Conserved | 0 | 0 | 0 | 0 | 3.16 |
| *NA* | lmo1326 | conserved hypothetical protein similar to B. subtilis YlxP protein | Conserved | 0 | 0 | 0 | 0 | 1.13 |
| *NA* | lmo1338 | similar to B. subtilis yqgQ | Conserved | 0 | 0 | 0 | 0 | 0.09 |
| *NA* | lmo1342 | similar to B. subtilis comG operon protein 6 | Conserved | 0 | 0 | 0 | 0 | -1.21 |
| *NA* | lmo1343 | similar to comG operon protein 5 (comGE) | Conserved | 0 | 0 | 0 | 0 | 1.24 |
| *NA* | lmo1353 | similar to B. subtilis YqhQ protein | Conserved | 0 | 0 | 0 | 0 | 1.91 |
| *NA* | lmo1380 | unknown | NA | 0 | 0 | 0 | 0 | 0.6 |
| *NA* | lmo1385 | similar to unknown protein | Conserved | 0 | 0 | 0 | 0 | 0.77 |
| *NA* | lmo1392 | similar to putative proteases | Conserved | 0 | 0 | 0 | 0 | 0.45 |
| *NA* | lmo1395 | similar to unknown protein | Conserved | 0 | 0 | 0 | 0 | -1.8 |
| *NA* | lmo1408 | similar to unknown proteins | Conserved | 0 | 0 | 0 | 0 | 2.03 |
| *NA* | lmo1423 | unknown | NA | 0 | 0 | 0 | 0 | 1.48 |
| *NA* | lmo1452 | conserved hypothetical protein | Conserved | 0 | 0 | 0 | 0 | 1.44 |
| *NA* | lmo1453 | conserved hypothetical protein | Conserved | 0 | 0 | 0 | 0 | 2.55 |
| *NA* | lmo1457 | similar to unknown protein | Conserved | 0 | 0 | 0 | 0 | -3.72 |
| *NA* | lmo1465 | similar to unknown proteins | Conserved | 0 | 0 | 0 | 0 | 0.47 |
| *NA* | lmo1470 | similar to unknown proteins | Conserved | 0 | 0 | 0 | 0 | -0.08 |
| *NA* | lmo1481 | similar to unknown protein | Conserved | 0 | 0 | 0 | 0 | -2.23 |
| *NA* | lmo1489 | similar to unknown proteins | Conserved | 0 | 0 | 0 | 0 | 0.42 |
| *NA* | lmo1499 | similar to unknown proteins | Conserved | 0 | 0 | 0 | 0 | -0.11 |
| *NA* | lmo1501 | similar to unknown proteins | Conserved | 0 | 0 | 0 | 0 | 0.12 |
| *NA* | lmo1502 | similar to unknown proteins | Conserved | 0 | 0 | 0 | 0 | 0.11 |
| *NA* | lmo1518 | unknown | Conserved | 0 | 0 | 0 | 0 | -0.1 |
| *NA* | lmo1522 | similar to unknown proteins | Conserved | 0 | 0 | 0 | 0 | 0.24 |
| *NA* | lmo1528 | similar to unknown proteins | Conserved | 0 | 0 | 0 | 0 | -1.15 |
| *NA* | lmo1562 | similar to unknown protein | Conserved | 0 | 0 | 0 | 0 | 1.35 |
| *NA* | lmo1594 | similar to B. subtilis negative regulator of FtsZ ring formation (EzrA) | Conserved | 0 | 0 | 0 | 0 | 0.34 |
| *NA* | lmo1608 | similar to unknown proteins | Conserved | 0 | 0 | 0 | 0 | 2.01 |
| *NA* | lmo1610 | hypothetical gene | NA | 0 | 0 | 0 | 0 | 0.45 |
| *NA* | lmo1612 | similar to unknown proteins | Conserved | 0 | 0 | 0 | 0 | 2.17 |
| *NA* | lmo1616 | similar to unknown proteins | Conserved | 0 | 0 | 0 | 0 | -0.02 |
| *NA* | lmo1635 | similar to unknown proteins | Conserved | 0 | 0 | 0 | 0 | 0.5 |
| *NA* | lmo1643 | unknown | NA | 0 | 0 | 0 | 0 | 0.25 |
| *NA* | lmo1648 | unknown | NA | 0 | 0 | 0 | 0 | 1.04 |
| *NA* | lmo1649 | unknown | NA | 0 | 0 | 0 | 0 | 0.36 |
| *NA* | lmo1650 | similar to hypothetical proteins | Conserved | 0 | 0 | 0 | 0 | 1.3 |
| *NA* | lmo1653 | putative cellsurface protein | NA | 0 | 0 | 0 | 0 | -0.72 |
| *NA* | lmo1654 | putative cellsurface protein | NA | 0 | 0 | 0 | 0 | 0.82 |
| *NA* | lmo1656 | unknown | NA | 0 | 0 | 0 | 0 | 0.78 |
| *NA* | lmo1659 | unknown | NA | 0 | 0 | 0 | 0 | 1.62 |
| *NA* | lmo1661 | similar to conserved hypothetical proteins | Conserved | 0 | 0 | 0 | 0 | -0.43 |
| *NA* | lmo1665 | unknown | NA | 0 | 0 | 0 | 0 | 1.01 |
| *NA* | lmo1668 | some similarity to hypothetical proteins | Conserved | 0 | 0 | 0 | 0 | 3.09 |
| *NA* | lmo1692 | unknown | Conserved | 0 | 0 | 0 | 0 | 0.83 |
| *NA* | lmo1696 | similar to unknown proteins | NA | 0 | 0 | 0 | 0 | 2.31 |
| *NA* | lmo1701 | unknown | NA | 0 | 0 | 0 | 0 | 1.11 |
| *NA* | lmo1704 | similar to conserved hypothetical proteins | Conserved | 0 | 0 | 0 | 0 | -0.24 |
| *NA* | lmo1707 | unknown | Conserved | 0 | 0 | 0 | 0 | 2.07 |
| *NA* | lmo1715 | similar to hypothetical proteins | Conserved | 0 | 0 | 0 | 0 | 0.79 |
| *NA* | lmo1717 | similar to hypothetical proteins | Conserved | 0 | 0 | 0 | 0 | 1.54 |
| *NA* | lmo1718 | similar to putative outer surface protein | Conserved | 0 | 0 | 0 | 0 | -0.25 |
| *NA* | lmo1723 | unknown | NA | 0 | 0 | 0 | 0 | 0.93 |
| *NA* | lmo1728 | some similarities to cellobiose-phosphorylase | Conserved | 0 | 0 | 0 | 0 | 0.27 |
| *NA* | lmo1743 | unknown | NA | 0 | 0 | 0 | 0 | 0.93 |
| *NA* | lmo1748 | unknown | NA | 0 | 0 | 0 | 0 | 1.36 |
| *NA* | lmo1762 | unknown | NA | 0 | 0 | 0 | 0 | 1.33 |
| *NA* | lmo1763 | similar to unknown protein | Conserved | 0 | 0 | 0 | 0 | 1.23 |
| *NA* | lmo1771 | similar to unknown protein | Conserved | 0 | 0 | 0 | 0 | 0.89 |
| *NA* | lmo1776 | similar to unknown protein | Conserved | 0 | 0 | 0 | 0 | -0.88 |
| *NA* | lmo1779 | unknown | NA | 0 | 0 | 0 | 0 | 0.37 |
| *NA* | lmo1781 | unknown | Conserved | 0 | 0 | 0 | 0 | -0.95 |
| *NA* | lmo1791 | unknown | NA | 0 | 0 | 0 | 0 | 1.05 |
| *NA* | lmo1794 | similar to unknown proteins | Conserved | 0 | 0 | 0 | 0 | 0.91 |
| *NA* | lmo1798 | similar to unknown protein | Conserved | 0 | 0 | 0 | 0 | 0.09 |
| *NA* | lmo1817 | weakly similar to thiamin pyrophosphokinase | Conserved | 0 | 0 | 0 | 0 | 0.17 |
| *NA* | lmo1819 | similar to unknown proteins | Conserved | 0 | 0 | 0 | 0 | 2.46 |
| *NA* | lmo1841 | unknown | NA | 0 | 0 | 0 | 0 | 0.38 |
| *NA* | lmo1861 | similar to hypothetical proteins | Conserved | 0 | 0 | 0 | 0 | 0.89 |
| *NA* | lmo1866 | similar to conserved hypothetical proteins | Conserved | 0 | 0 | 0 | 0 | 1.05 |
| *NA* | lmo1869 | similar to conserved hypothetical proteins, putative integral membrane protein | Conserved | 0 | 0 | 0 | 0 | -0.44 |
| *NA* | lmo1890 | similar to conserved hypothetical proteins | Conserved | 0 | 0 | 0 | 0 | -1.62 |
| *NA* | lmo1893 | unknown | Conserved | 0 | 0 | 0 | 0 | 2.29 |
| *NA* | lmo1898 | similar to hypothetical proteins | Conserved | 0 | 0 | 0 | 0 | 2.68 |
| *NA* | lmo1908 | similar to unknown proteins | Conserved | 0 | 0 | 0 | 0 | 0.64 |
| *NA* | lmo1919 | similar to unknown proteins | Conserved | 0 | 0 | 0 | 0 | -1.34 |
| *NA* | lmo1921 | similar to unknown proteins | Conserved | 0 | 0 | 0 | 0 | 1.74 |
| *NA* | lmo1965 | similar to unknown proteins | Conserved | 0 | 0 | 0 | 0 | 0.62 |
| *NA* | lmo1981 | similar to unknown proteins | Conserved | 0 | 0 | 0 | 0 | 0.19 |
| *NA* | lmo2012 | similar to unknown proteins | Conserved | 0 | 0 | 0 | 0 | -1.36 |
| *NA* | lmo2013 | similar to unknown proteins | Conserved | 0 | 0 | 0 | 0 | 0.14 |
| *NA* | lmo2014 | similar to putative sugar hydrolases | Conserved | 0 | 0 | 0 | 0 | 1.2 |
| *NA* | lmo2041 | similar to unknown proteins | Conserved | 0 | 0 | 0 | 0 | -1.74 |
| *NA* | lmo2042 | similar to unknown proteins | Conserved | 0 | 0 | 0 | 0 | -0.21 |
| *NA* | lmo2045 | unknown | NA | 0 | 0 | 0 | 0 | 0.53 |
| *NA* | lmo2049 | similar to unknown proteins | Conserved | 0 | 0 | 0 | 0 | -1.43 |
| *NA* | lmo2055 | similar to unknown proteins | Conserved | 0 | 0 | 0 | 0 | 0.57 |
| *NA* | lmo2060 | similar to unknown protein | Conserved | 0 | 0 | 0 | 0 | -2.42 |
| *NA* | lmo2061 | similar to unknown protein | Conserved | 0 | 0 | 0 | 0 | 0.92 |
| *NA* | lmo2065 | unknown | NA | 0 | 0 | 0 | 0 | -2.38 |
| *NA* | lmo2066 | unknown | NA | 0 | 0 | 0 | 0 | 1.9 |
| *NA* | lmo2071 | unknown | NA | 0 | 0 | 0 | 0 | 0.02 |
| *NA* | lmo2086 | weakly similar to transcription regulators | NA | 0 | 0 | 0 | 0 | -2.57 |
| *NA* | lmo2093 | unknown | NA | 0 | 0 | 0 | 0 | 0.49 |
| *NA* | lmo2106 | similar to unknown proteins | Conserved | 0 | 0 | 0 | 0 | -0.23 |
| *NA* | lmo2113 | similar to unknown proteins | Conserved | 0 | 0 | 0 | 0 | -0.56 |
| *NA* | lmo2116 | unknown | NA | 0 | 0 | 0 | 0 | -1.01 |
| *NA* | lmo2131 | unknown | NA | 0 | 0 | 0 | 0 | -0.82 |
| *NA* | lmo2150 | unknown | NA | 0 | 0 | 0 | 0 | -2.03 |
| *NA* | lmo2162 | similar to unknown proteins | Conserved | 0 | 0 | 0 | 0 | -1.01 |
| *NA* | lmo2166 | unknown | NA | 0 | 0 | 0 | 0 | 0.11 |
| *NA* | lmo2187 | unknown | Conserved | 0 | 0 | 0 | 0 | 0.91 |
| *NA* | lmo2221 | similar to unknown proteins | Conserved | 0 | 0 | 0 | 0 | -1.24 |
| *NA* | lmo2224 | similar to unknown proteins | Conserved | 0 | 0 | 0 | 0 | -1.62 |
| *NA* | lmo2226 | similar to unknown proteins | Conserved | 0 | 0 | 0 | 0 | -2.39 |
| *NA* | lmo2228 | similar to unknown protein | NA | 0 | 0 | 0 | 0 | 1.49 |
| *NA* | lmo2234 | similar to unknown proteins | Conserved | 0 | 0 | 0 | 0 | -1.22 |
| *NA* | lmo2255 | unknown | NA | 0 | 0 | 0 | 0 | -1.99 |
| *NA* | lmo2257 | hypothetical CDS | NA | 0 | 0 | 0 | 0 | -0.71 |
| *NA* | lmo2262 | similar to unknown proteins | Conserved | 0 | 0 | 0 | 0 | -1.15 |
| *NA* | lmo2271 | unknown | NA | 0 | 0 | 0 | 0 | -0.42 |
| *NA* | lmo2272 | unknown | Conserved | 0 | 0 | 0 | 0 | -0.52 |
| *NA* | lmo2301 | similar to putative terminase small subunit from Bacteriophage A118 | Conserved | 0 | 0 | 0 | 0 | -0.4 |
| *NA* | lmo2306 | similar to phage protein | Conserved | 0 | 0 | 0 | 0 | 0.71 |
| *NA* | lmo2307 | Hypothetical protein | NA | 0 | 0 | 0 | 0 | 0.22 |
| *NA* | lmo2310 | unknown | NA | 0 | 0 | 0 | 0 | 0.44 |
| *NA* | lmo2311 | unknown | NA | 0 | 0 | 0 | 0 | 0.24 |
| *NA* | lmo2314 | unknown | NA | 0 | 0 | 0 | 0 | 0.19 |
| *NA* | lmo2318 | unknown | Conserved | 0 | 0 | 0 | 0 | 0.37 |
| *NA* | lmo2319 | similar to bacteriophage proteins | Conserved | 0 | 0 | 0 | 0 | -0.51 |
| *NA* | lmo2320 | unknown | NA | 0 | 0 | 0 | 0 | -1.21 |
| *NA* | lmo2325 | unknown | NA | 0 | 0 | 0 | 0 | -0.59 |
| *NA* | lmo2327 | unknown | NA | 0 | 0 | 0 | 0 | -1.62 |
| *NA* | lmo2331 | weakly similar to gp32_Bacteriophage A118 protein | NA | 0 | 0 | 0 | 0 | 0.45 |
| *NA* | lmo2340 | similar to Erwinia chrysanthemi IndA protein | Conserved | 0 | 0 | 0 | 0 | -0.26 |
| *NA* | lmo2343 | similar to nitrilotriacetate monooxygenase | Conserved | 0 | 0 | 0 | 0 | -1.37 |
| *NA* | lmo2344 | similar to B. subtilis YtnI protein | Conserved | 0 | 0 | 0 | 0 | -1.3 |
| *NA* | lmo2351 | similar to NADH-dependent FMN reductase | Conserved | 0 | 0 | 0 | 0 | -2.06 |
| *NA* | lmo2357 | similar to unknown protein | Conserved | 0 | 0 | 0 | 0 | -0.2 |
| *NA* | lmo2364 | Hypothetical protein | NA | 0 | 0 | 0 | 0 | 1.09 |
| *NA* | lmo2392 | similar to B. subtilis YuzB protein | Conserved | 0 | 0 | 0 | 0 | -0.4 |
| *NA* | lmo2394 | hypothetical CDS | NA | 0 | 0 | 0 | 0 | -0.03 |
| *NA* | lmo2395 | unknown | NA | 0 | 0 | 0 | 0 | 0.96 |
| *NA* | lmo2402 | similar to B. subtilis YutD protein | Conserved | 0 | 0 | 0 | 0 | 0.51 |
| *NA* | lmo2407 | unknown | NA | 0 | 0 | 0 | 0 | -0.26 |
| *NA* | lmo2410 | unknown | Conserved | 0 | 0 | 0 | 0 | -0.07 |
| *NA* | lmo2420 | unknown | NA | 0 | 0 | 0 | 0 | 0.02 |
| *NA* | lmo2435 | similar to B. subtilis YfhL protein | Conserved | 0 | 0 | 0 | 0 | -1.95 |
| *NA* | lmo2438 | unknown | Conserved | 0 | 0 | 0 | 0 | 0.14 |
| *NA* | lmo2440 | unknown | Conserved | 0 | 0 | 0 | 0 | 1.6 |
| *NA* | lmo2442 | unknown | Conserved | 0 | 0 | 0 | 0 | 0.71 |
| *NA* | lmo2443 | unknown | NA | 0 | 0 | 0 | 0 | -0.15 |
| *NA* | lmo2466 | unknown | Conserved | 0 | 0 | 0 | 0 | 0.32 |
| *NA* | lmo2474 | conserved hypothetical protein | Conserved | 0 | 0 | 0 | 0 | 0.82 |
| *NA* | lmo2490 | similar to B. subtilis CsbA protein | Conserved | 0 | 0 | 0 | 0 | -0.65 |
| *NA* | lmo2502 | unknown | Conserved | 0 | 0 | 0 | 0 | -0.43 |
| *NA* | lmo2553 | conserved hypothetical protein | Conserved | 0 | 0 | 0 | 0 | 1.87 |
| *NA* | lmo2563 | conserved hypothetical protein | Conserved | 0 | 0 | 0 | 0 | 0.7 |
| *NA* | lmo2594 | unknown | NA | 0 | 0 | 0 | 0 | 1.39 |
| *NA* | lmo2595 | similar to unknown proteins | NA | 0 | 0 | 0 | 0 | 0.58 |
| *NA* | lmo2604 | unknown | Conserved | 0 | 0 | 0 | 0 | 1.83 |
| *NA* | lmo2639 | unknown | Conserved | 0 | 0 | 0 | 0 | 1.68 |
| *NA* | lmo2642 | unknown | NA | 0 | 0 | 0 | 0 | -5.05 |
| *NA* | lmo2643 | unknown | Conserved | 0 | 0 | 0 | 0 | 0.83 |
| *NA* | lmo2644 | unknown | Conserved | 0 | 0 | 0 | 0 | 1.16 |
| *NA* | lmo2646 | unknown | Conserved | 0 | 0 | 0 | 0 | 0.33 |
| *NA* | lmo2686 | unknown | NA | 0 | 0 | 0 | 0 | -1.43 |
| *NA* | lmo2692 | unknown | Conserved | 0 | 0 | 0 | 0 | 0.28 |
| *NA* | lmo2697 | unknown | Conserved | 0 | 0 | 0 | 0 | -1.87 |
| *NA* | lmo2701 | similar to B. subtilis YaaL protein | Conserved | 0 | 0 | 0 | 0 | -1.44 |
| *NA* | lmo2703 | highly similar to B. subtilis YaaK protein | Conserved | 0 | 0 | 0 | 0 | 0.47 |
| *NA* | lmo2709 | unknown | NA | 0 | 0 | 0 | 0 | -0.86 |
| *NA* | lmo2710 | unknown | NA | 0 | 0 | 0 | 0 | 1.11 |
| *NA* | lmo2711 | similar to hypothetical proteins | NA | 0 | 0 | 0 | 0 | -0.75 |
| *NA* | lmo2729 | similar to unknown proteins | Conserved | 0 | 0 | 0 | 0 | 1.09 |
| *NA* | lmo2731 | similar to transcription regulator (RpiR family) | NA | 0 | 0 | 0 | 0 | 0.02 |
| *NA* | lmo2734 | weakly similar to sugar hydrolase | Conserved | 0 | 0 | 0 | 0 | -1.6 |
| *NA* | lmo2736 | conserved hypothetical protein | Conserved | 0 | 0 | 0 | 0 | 1.21 |
| *NA* | lmo2746 | unknown | Conserved | 0 | 0 | 0 | 0 | 0.75 |
| *NA* | lmo2753 | unknown | Conserved | 0 | 0 | 0 | 0 | 0.71 |
| *NA* | lmo2768 | hypothetical membrane protein | Conserved | 0 | 0 | 0 | 0 | -2.71 |
| *NA* | lmo2775 | hypothetical membrane protein | Conserved | 0 | 0 | 0 | 0 | 1.03 |
| *NA* | lmo2776 | unknown | NA | 0 | 0 | 0 | 0 | 1.95 |
| *NA* | lmo2778 | unknown | NA | 0 | 0 | 0 | 0 | 0.27 |
| *NA* | lmo2793 | unknown | NA | 0 | 0 | 0 | 0 | -2.14 |
| *NA* | lmo2804 | unknown | Conserved | 0 | 0 | 0 | 0 | 1.04 |
| *NA* | lmo2805 | hypothetical secreted protein | NA | 0 | 0 | 0 | 0 | 3.06 |
| *NA* | lmo2807 | hypothetical secreted protein | NA | 0 | 0 | 0 | 0 | -0.19 |
| *NA* | lmo2808 | hypothetical secreted protein | NA | 0 | 0 | 0 | 0 | 0.71 |
| *NA* | lmo2809 | hypothetical secreted protein | NA | 0 | 0 | 0 | 0 | 4.53 |
| *NA* | lmo2813 | unknown | NA | 0 | 0 | 0 | 0 | -0.12 |
| *NA* | lmo2822 | unknown | NA | 0 | 0 | 0 | 0 | 1.5 |
| *NA* | lmo2823 | similar to an hypothetical protein from Thermotoga maritima | Conserved | 0 | 0 | 0 | 0 | 3.44 |
| *NA* | lmo2832 | similar to unknown proteins | Conserved | 0 | 0 | 0 | 0 | -0.08 |
| *NA* | lmo2835 | highly similar to an E. coli protein | Conserved | 0 | 0 | 0 | 0 | 1.18 |
| *NA* | lmo2843 | unknown | NA | 0 | 0 | 0 | 0 | -2.79 |
| *NA* | lmo2846 | highly similar to B. subtilis YulD protein | Conserved | 0 | 0 | 0 | 0 | -0.87 |
| **Disrupted reading frame** | | |  |  |  |  |  |  |
| *NA* | LMOf2365_2458 | PspC domain protein, truncated | NA | 3.22 | 0 | 0 | 0 | 0 |
| *comK* | lmo2270 | similar to competence transcription factor ComK, N terminal part | NA | 0 | 0 | 0 | 0 | -1.02 |

a This study

b Differentially expressed genes of *L. monocytogenes* EGD-e at designated time-points after exposure to 48 ºC in comparison with time-zero

expression at 37 ºC. Hain et al. 2007. Microbiology **153** : 3593-3607.

c Differentially expressed genes of *L. monocytogenes* at 4°C during logarithmic-phase. Chan et al. 2007. [Appl Environ Microbiol.](javascript:AL_get(this, 'jour', 'Appl Environ Microbiol.');) **73:**6484-98.

d Summary of genes identified by microarray analyses as positively regulated by σB in L. monocytogenes salt stress. Raengpradub et al., 2008. Appl. Environ. Microbiol. **74:** 158-171.

e Differential gene expression (log2 ratio) of *Listeria monocytogenes* during high hydrostatic pressure processing. Bowman et al. 2007 GEO series accession [number GSE9179](http://www.ncbi.nlm.nih.gov/geo/query/acc.cgi?acc=GSE9179)**.**
